# On the Role of Hydrogen Migrations in the Taxadiene System

**DOI:** 10.1002/anie.202422788

**Published:** 2025-01-10

**Authors:** Heng Li, Bernd Goldfuss, Jeroen S. Dickschat

**Affiliations:** ^1^ Kekulé Institute of Organic Chemistry and Biochemistry University of Bonn Gerhard-Domagk-Straße 1 53121 Bonn Germany; ^2^ Department for Chemistry University of Cologne Greinstrasse 4 50939 Cologne Germany

**Keywords:** biosynthesis, enzymes, natural products, substrate analogs, terpenoids

## Abstract

Taxa‐4,11‐diene is made by the taxa‐4,11‐diene synthase (TxS) from *Taxus brevifolia*. The unique reactivity of the taxane system is characterised by long distance hydrogen migrations in the biosynthesis. This study demonstrates that selective long range hydrogen migrations also play a role in the high energy process of the EI‐MS fragmentation of taxa‐4,11‐diene. A TxS enzyme variant was generated that produces cyclophomactene, a compound that is formed through a concerted process involving a long range proton shift and a ring closure that can also be described as the addition of a methylcarbinyl cation to an olefin. Based on a previous computational study the cyclisation mechanism towards taxa‐4,11‐diene was suggested to involve two long distance proton migrations instead of one direct transfer. A substrate analog with a shifted double bond was converted with TxS to obtain experimental evidence for this proposal.

## Graphical abstract

Long range proton migrations are known to play a role in the biosynthesis of taxa‐4,11‐diene. This study demonstrates the involvement of long distance hydrogen transfers also in the mass spectrometric fragmentation, and combined approaches through site‐directed mutagenesis and enzymatic conversion of designed substrate analogs give detailed insights into their involvement in the biosynthesis of taxa‐4,11‐diene and related compounds.

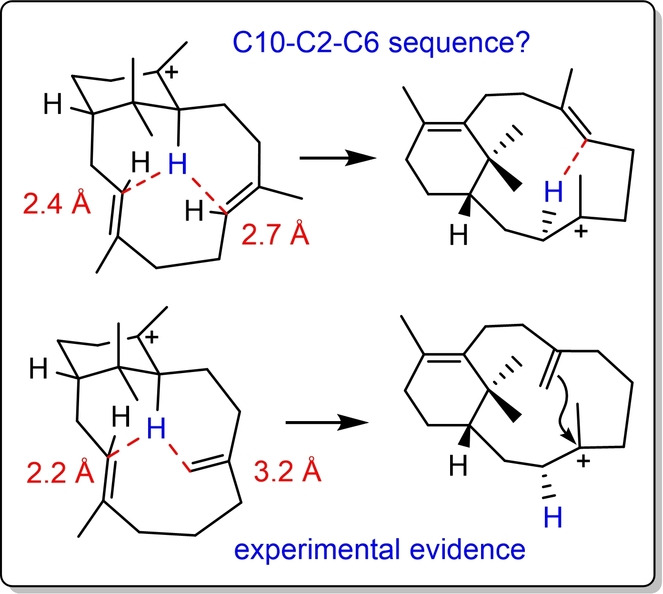

## Introduction

Taxol was first isolated from the stem bark of Pacific yew (*Taxus brevifolia*).^[1]^ The compound exhibits a pronounced cytotoxicity by enhancing the polymerisation of tubulin^[2]^ and is in clinical use for the treatment of various types of cancer. The low content in the bark and the slow growth of the tree lead to a supply problem that is addressed through genetic engineering of microbial strains,[^3^, ^4^] which requires profound knowledge of the biosynthetic genes and enzymes involved in its biosynthesis.[^5^, ^6^, ^7^, ^8^, ^9^, ^10^, ^11^, ^12^, ^13^, ^14^]

**Scheme 1:**
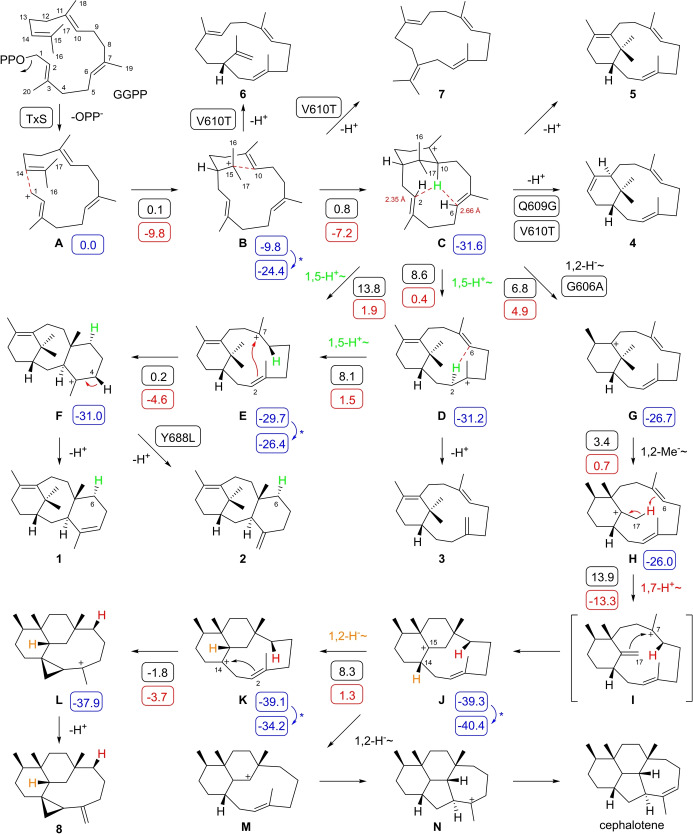
Taxa‐4,11‐diene biosynthesis. A) Mechanism for the cyclisation of GGPP to **1** and biosynthetically related products by TxS and its enzyme variants. For isolated products the source enzymes are given in boxes. The numbers in boxes are computed energies (in kcal/mol) of intermediates relative to **A** (set to 0.0 kcal/mol, blue), reaction barriers (Gibbs free energies of activation at 298.15 K, black) and Gibbs free reaction energies (red) relative to each preceeding intermediate. All structures were computed using the mPW1PW91/6‐311+G(d,p)//B97D3/6‐31 G(d,p) method (298 K). Asterisks indicate conformational changes needed between the product of one transformation and the starting structure of the next step. All carbon numbers follow GGPP numbering, which is different to the accepted carbon numbering of taxanes.^[15]^

The first committed step is catalysed by taxa‐4,11‐diene (**1**) synthase (TxS, Scheme 1) that has originally been purified from *Taxus brevifolia*,^[15]^ followed by cloning and heterologous expression of its coding nucleotide sequence and functional characterisation of the enzyme.^[16]^ The crystal structure of TxS has been reported^[17]^ and the enzyme has extensively been investigated through site‐directed mutagenesis.[^18^, ^19^] Initially suggested mechanisms[^20^, ^21^] for the formation of **1** start with the abstraction of diphosphate from the diterpene precursor geranylgeranyl diphosphate (GGPP) to yield cation **A**. A subsequent 1,14‐cyclisation to **B** and a 10,15‐cyclisation result in **C** that were suggested to undergo a deprotonation from C10 to a neutral intermediate (verticillene) and its reprotonation to **E**.^[20]^ However, incubation experiments with (10‐^2^H)GGPP revealed a retainment of deuterium with incorporation into H6α of **1**, leading to the suggestion that **C** may react in a direct 1,5‐proton transfer from C10 to C6 to yield **E**.^[21]^ The ultimate 2,7‐cyclisation to **F** and deprotonation from C4 would then result in **1**.

Also several stereochemical details of the cyclisation process have been investigated. The stereospecificity of the terminal deprotonation step was investigated using (*R*)‐(4‐^2^H)GGPP, showing selective removal of a proton from the *Re* face at C4.^[21]^ Incubation experiments with stereoselectively deuterated (*R*)‐ and (*S*)‐(1‐^2^H)GGPP revealed the inversion of configuration at C1 in the initial 1,14‐cyclisation, and a selective deuterium labelling at Me16, realised in the substrate (16,16,16‐^2^H_3_)GGPP, established a stereochemical course for the 10,15‐cyclisation from **B** to **C** with attack at C15 from the *Re* face (for unlabelled GGPP, this is identical to the *Si* face of GGPP deuterated at C16).^[22]^

DFT calculations using the B3LYP/6‐31+G(d,p) method reveiled that the situation for the intramolecular proton migrations is more complex than initially anticipated. Besides the direct conversion of **C** to **E** also two sequential 1,5‐proton transfers with translocation of a proton from C10 to C2 (**C** to **D**) and then from C2 to C6 (**D** to **E**) are possible.[^23^, ^24^] Interestingly, the energy barriers for the two‐step process from **C** to **E** were lower than for the direct proton shift.[^23^, ^24^] The existence of cation **D** was also supported by a docking study,^[19]^ while QM/MM calculations in the environment of the enzyme by Major and co‐workers suggested a slight energetic preference for the direct pathway.[^25^, ^26^] In contrast, QM/MM calculations by Thiel and co‐workers were in favour of the **C**‐**D**‐**E** transformation.[^27^, ^28^]

**Scheme 2:**
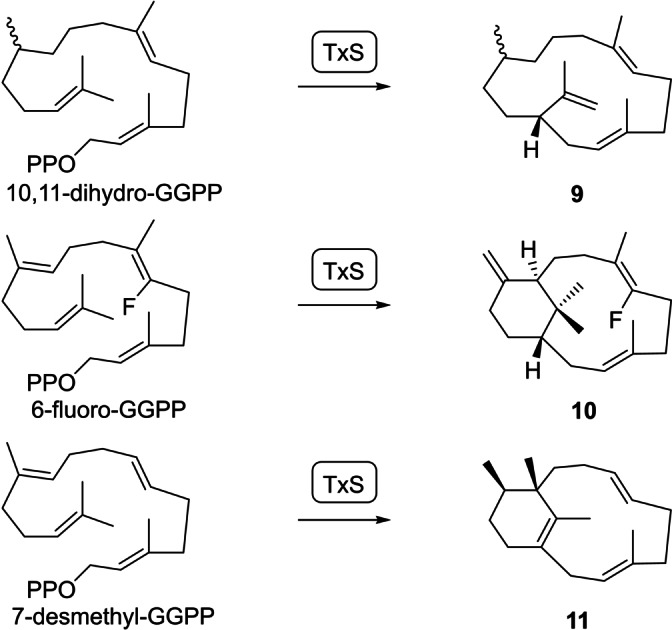
Products obtained with TxS from GGPP derivatives.

The cyclisation cascade was further interrogated using substrate analogs with a reduced reactivity (Scheme 2). The enzymatic conversion of (*R*)‐ and (*S*)‐10,11‐dihydro‐GGPP resulted in the corresponding stereoisomers of **9**,^[29]^ while 6‐fluoro‐GGPP with an electronically buffered reactivity in the C6=C7 double bond yielded several fluorinated diterpenes including the main product **10**.^[30]^ Also 7‐desmethyl‐GGPP has a reduced reactivity in the C6=C7 double bond, because its participation in proton transfers or cyclisation reactions would lead to a secondary cation. Consequently, the bicyclic hydrocarbon **11** was isolated from this substrate.^[31]^ While **9** and **10** represent the deprotonation products of the analogs of intermediates **B** and **C**, compound **11** arises from a **C** analog by hydride and methyl migrations and deprotonation.

Here we report on advanced isotopic labelling experiments to investigate the cyclisation cascade towards **1** and its EI‐MS fragmentation mechanism. Site‐directed mutagenesis and incubation experiments using a substrate analog with a shifted double bond gave additional insights into the unusual long‐range proton shifts in the biosynthesis of **1** and related molecules.

## Results and Discussion

All experiments performed in this study are based on a truncated and His‐tagged taxadiene synthase lacking 78 amino acids at the N‐terminus, further referred to as “TxS” in this study. This enzyme is known to give high yields of soluble protein in the expression in *Escherichia coli* (Figure S1) and exhibits a high activity in the in vitro conversion of GGPP into taxa‐4,11‐diene (**1**, 87 %) and its double bond isomer taxa‐4(20),11‐diene (**2**) (13 %).^[32]^

The isotopic labelling experiments performed in this study are summarised in Table S1. For interpretation of the results **1** and **2** were isolated from a preparative scale incubation of GGPP (Table S2), followed by a complete NMR assignment (Tables S3 and S4, Figures S2–S10). Compound **2** has initially been identified as a TxS product by GC/MS^[33]^ in comparison to a synthetic authentic standard,^[34]^ but has never been isolated before from an enzymatic reaction with TxS. For an unambiguous assignment of all diastereotopic hydrogen atoms incubation experiments with (*R*)‐ and (*S*)‐(1‐^13^C,1‐^2^H)isopentenyl diphosphate (IPP),^[35]^ and with dimethylallyl diphosphate (DMAPP) plus (*E*)‐ or (*Z*)‐(4‐^13^C,4‐^2^H)IPP^[36]^ were performed (Figures S11 and S12). These stereoselectively deuterated precursors were enzymatically converted into **1** with *E. coli* isopentenyl diphosphate isomerase (IDI,^[37]^ only used for the substrates labelled at C1), *Streptomyces cyaneofuscatus* GGPP synthase (GGPPS)^[38]^ and TxS, followed by HSQC analysis of the product, showing vanished crosspeaks for the hydrogens substituted by deuterium. As a result, a correction for the previous hydrogen assignment at C9 of **1** ^[20]^ is necessary (Table S3). These experiments require knowledge about the stereochemical course of the oligomerisation of the terpene precursor monomers^[39]^ and about the absolute configuration of **1** that can be inferred from the absolute configuration of taxol^[1]^ and has been established through enantioselective synthesis.^[40]^

**Scheme 3:**
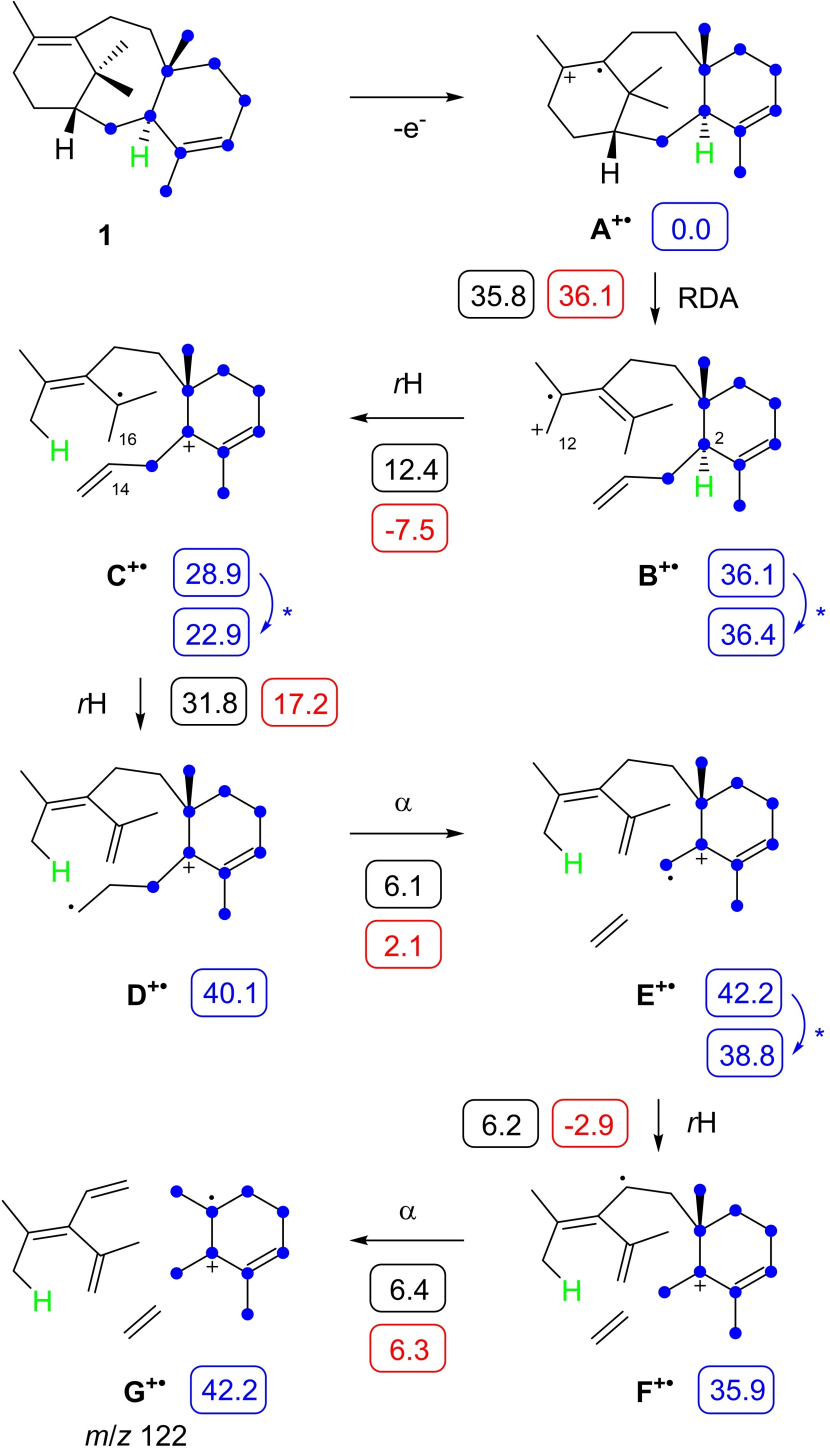
EIMS fragmentation mechanism for the base peak ion *m*/*z* 122 of **1**. Blue dots indicate carbons for which a substitution with ^13^C leads to an increase of the base peak ion to *m*/*z* 123. The numbers in boxes are computed energies (in kcal/mol) of intermediates relative to **A** (set to 0.0 kcal/mol, blue), reaction barriers (Gibbs free energies of activation at 298.15 K, black) and Gibbs free reaction energies (red) relative to each preceeding intermediate. All structures were computed using the mPW1PW91/6‐311+G(d,p)//B97D3/6‐31 G(d,p) method (298 K).

The conversion of all twenty isotopomers of (^13^C)GGPP, obtained through chemical synthesis[^38^, ^41^] or by enzymatic preparation from its correspondingly labelled prenyl diphosphate precursors,[^38^, ^42^, ^43^, ^44^] with TxS confirmed the general carbon skeleton assambly including the precise stereochemical course for the geminal Me groups C16 and C17 (Figures S13 and S14).^[22]^ The EI mass spectrum of **1** exhibits a base peak ion at *m*/*z* 122 (Figure S15), and inspection of the mass spectra of its singly ^13^C‐labelled isotopomers showed an increase to *m*/*z* 123, if one of the carbons marked by the blue dots in Scheme 3 is substituted with ^13^C, indicating that this fragment ion arises from the nine carbons in the eastern portion of **1** (Figures S16 and S17). Mechanistically, this is explainable by the ionisation of **1** to **A^+^** ⋅, which preferentially takes place at the highest substituted (most electron rich) double bond, followed by a retro‐Diels–Alder (RDA) fragmentation to **B^+^** ⋅ and a hydrogen migration to **C^+^** ⋅. A subsequent hydrogen migration to **D^+^** ⋅ induces the neutral loss of ethylene by α‐fragmentation to **E^+^** ⋅ that can undergo another hydrogen rearrangement to **F^+^** ⋅ and α‐cleavage to **G^+^** ⋅ (*m*/*z* 122) (Scheme 3).

This fragmentation mechanism was further investigated through DFT calculations (Table S5, Figure S18), revealing the higest activation barrier (+35.8 kcal/mol) and a strongly positive Gibbs free energy (+36.1 kcal/mol) for the RDA, which can be overcome by the high ionisation energy used (70 eV≈1614 kcal/mol) that leaves the molecule after ionisation in a highly excited state. **B^+^** ⋅ is obtained in a conformation that enables the next hydrogen transfer to **C^+^** ⋅ through a distance of 3.4 Å between H2 and C12 (Figure S19A) with a low reaction barrier (12.4 kcal/mol). Also **C^+^** ⋅ is formed with a short distance of 2.6 Å between C14 and H16 for the next hydrogen transfer to **D^+^** ⋅ (Figure S19B). In this case the barrier is much higher (31.8 kcal/mol), because the reaction leads to a primary radical cation. After α‐cleavage of ethylene to **E^+^** ⋅ a conformational change is required to allow for the hydrogen migration to **F^+^** ⋅ (Figure S19C). The reaction barriers for both steps and the terminal α‐fragmentation are with ca. 6 kcal/mol in all three cases low. A key step of this mechanism is the long range hydrogen migration from C2 to C12 (**B^+^** ⋅ to **C^+^** ⋅) that was investigated through incubation of (2‐^2^H)GGPP^[45]^ with TxS and analysis of the product **1** by GC/MS (Figure S20). Observation of the base peak at *m*/*z* 122 confirmed the loss of deuterium from the corresponding fragment ion and thus the long distance hydrogen migration during the fragmentation reaction.

TxS has been extensively studied through site‐directed mutagenesis.[^17^, ^19^] All previously reported enzyme variants have been designed based on the structure of the enzyme and have targeted active site residues. The results revealed that TxS is highly sensitive to reshaping of the active site, as many of the tested enzyme variants were inactive or exhibited a strongly reduced production (Tables S6–S8). Frequently observed products include, besides **1** and **2**, the hydrocarbons verticilla‐4(20),7,11‐triene (**3**),^[46]^ verticilla‐3,7,12‐triene (**4**),^[19]^ and cembrene A (**6**)^[47]^ that can be explained as the deprotonation products of intermediates **B**, **C**, and **D**, respectively (Scheme 1). Also verticilla‐3,7,11‐triene (**5**) has tentatively been identified from wildtype TxS and various enzyme variants,[^18^, ^19^] but has never been isolated and properly structurally characterised. Moreover, its isomer **3** has been isolated with NMR‐based structure elucidation from various plant sources,[^46^, ^48^] but its structure was confused with that of **5** in a later publication,^[49]^ resulting in the assignment of a CAS number and a confusion about the identity of **5** as a TxS product. We tried to isolate this material from a large scale enzyme incubation, but unfortunately the production of **5** was too low for an unambiguous structure elucidation. Based on GC/MS data we can confirm the observation of the same compound as reported by Brück and co‐workers,^[19]^ but its identity remains at the end unclear. Notable discoveries are the selective formation of **4** by the Q609G variant and the good production of **2** by the Y688L variant.^[18]^

**Figure 1:**
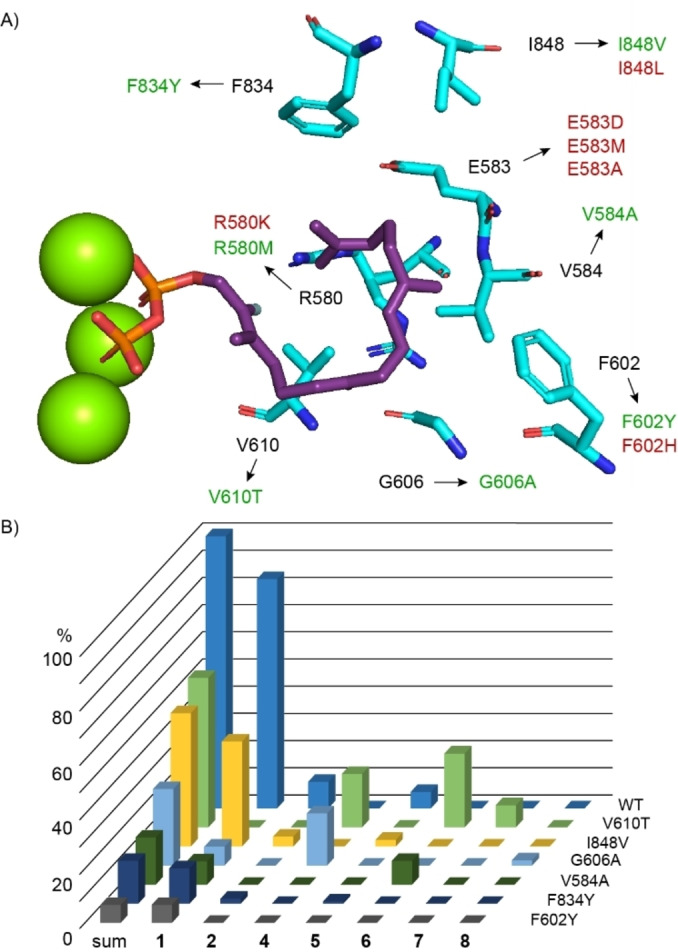
Site‐directed mutagenesis of TxS. A) Active site residues targeted by site‐directed mutagenesis in this study (based on the X‐ray structure of TxS, PDB 3P5R). Enzyme variants shown in green were catalytically active, those shown in red were inactive. B) Relative production of enzyme variants. The sum of compounds produced by the wild‐type (WT) was set to 100 %. Bars represent means from triplicates. For standard deviations cf. Table S8.

This work was expanded through the investigation of additional enzyme variants, addressing the active site residues shown in Figure 1A (the results are summarised in Figure 1B, Table S8 and Figure S21). Several of these residues have not been tested before, including E583, G606 and I848. All three enzyme variants E583D, E583M and E583A were inactive, demonstrating that amino acid exchanges in this position even against the structurally similar Asp are critical. A structural analysis revealed a hydrogen bond of E583 to a cluster of four water molecules at the enzyme surface (Figure S22), suggesting that E583 serves as a gatekeeper that blocks the entrance of water to the active site. The G606A variant showed a reduced productivity, but a good selectivity for **4**, giving access to an enzyme for the selective production of this compound. This variant also formed a side product that was isolated and structurally characterised as the new compound cyclophomactene (**8**) (Table S9, Figures S23–S30). The exchange of I848 against other hydrophobic residues gave surprising results: While the I848V variant only showed a moderately reduced activity, the I848L variant was completely inactive. A comparison of the X‐ray structure with an AlphaFold2 model of the I848L variant reveals a strong disturbance of F834, a residue presumably involved in a cation‐π stabilisation of Intermediate **B**, causing a rotation of F834 by ca. 90° and explaining the loss of acivity, while only a minor disturbance is observed in the I848V variant (Figure S31).

Additional enzyme variants targeted active site positions that were investigated before,[^18^, ^19^] but with exchange to different residues than in the previous experiments. R580 adopts two alternative conformations. Its exchange against another basic residue in the R580K variant or against a large hydrophobic residue (R580M) resulted in inactivity. Aromatic active site residues are potentially involved in the stabilisation of cationic intermediates. The exchange of F602 and F834 against Tyr gave in both cases a lower productivity, while the F602H variant was inactive. The V584A variant resulted in a substantially lower production of **1**, with additional formation of **6**. The most interesting results were obtained with the V610T variant that completely lost the ability to produce **1**. Instead, compounds **4** and **6** in addition to another diterpene hydrocarbon (**7**) that could not be identified by GC/MS were obtained. Compound **7** was isolated and identified as a known macrocyclic diterpene hydrocarbon for which we propose the name cembrene D (Figure S32, Table S10). The same hydrocarbon **7** was previously isolated from the soft coral *Sarcophyton glaucum* ^[50]^ and is a side product of the diterpene synthase DtcycA from *Streptomyces* sp. SANK 60404.^[51]^

**Scheme 4:**
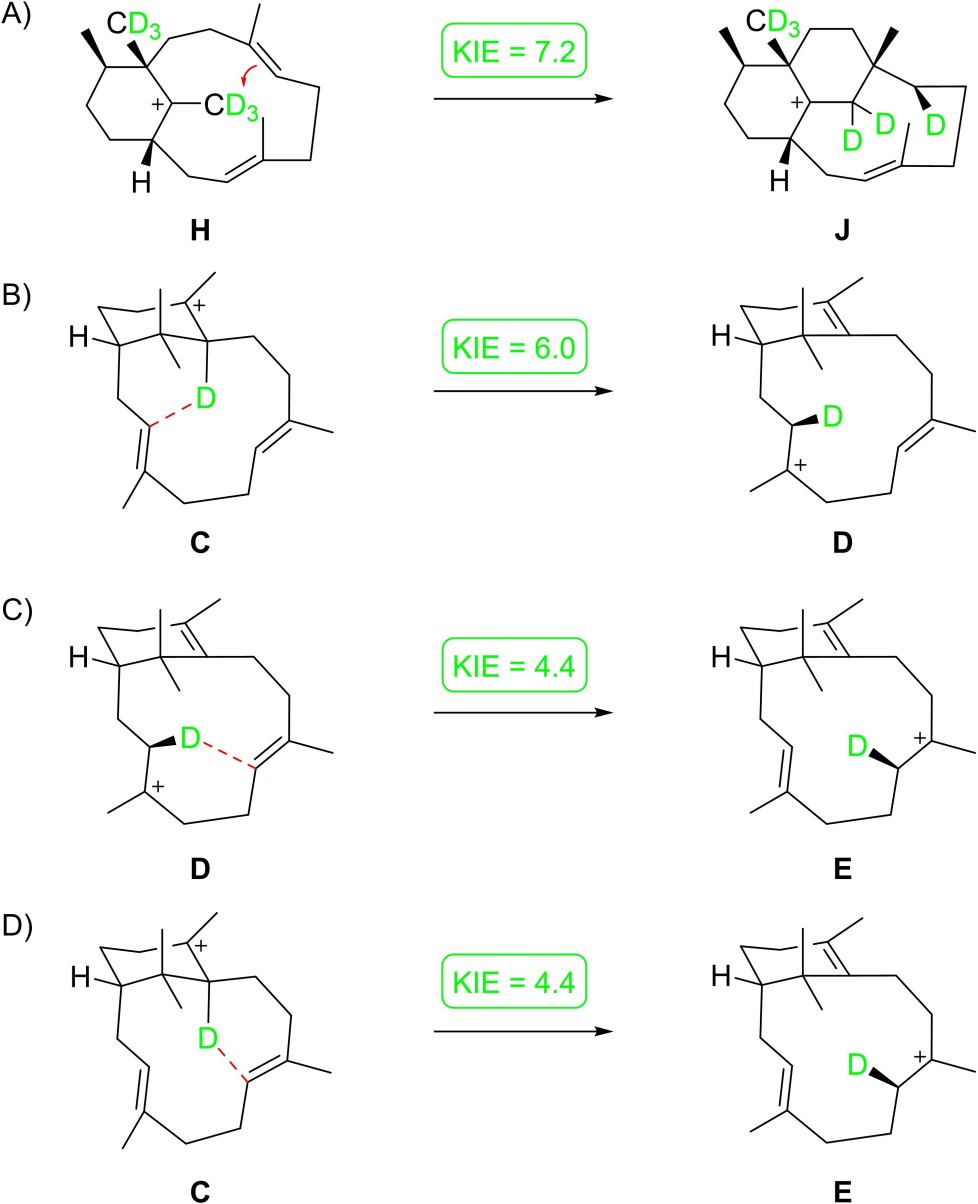
Computed kinetic isotope effects (ratio of rate constants *k*_H_/*k*_D_) for long distance hydrogen migrations in the biosynthesis of **1** and **8**, determined with H/D exchange of only the migrating hydrogen. Kinetic isotope effects for A) the unusual cyclisation reaction in the biosynthesis of **8** and B–D) the 1,5‐proton shifts in the biosynthesis of **1**.

The formation of the new compound **8** was investigated through DFT calculations using the advanced mPW1PW91/6‐311+G(d,p)//B97D3/6‐31G(d,p) method. These calculations showed similar results as previously obtained with three different methods (including B3LYP/6‐31+G(d,p), mPW1PW91/6‐31+G(d,p)//B3LYP/6‐31+G(d,p) and MPWB1K/6‐31+G(d,p)//B3LYP/6‐31+G(d,p)) for the reactions from intermediate **A** to **F**[^23^, ^24^] (Scheme 1, Table S11, Figure S33). In particular, the sequence of two 1,5‐proton shifts (**C**‐**D**‐**E**) was favoured over the direct 1,5‐proton shift from **C** to **E** by the lower reaction barriers. The pathway to **8** branches out from **C** by a 1,2‐hydride shift to **G** and a 1,2‐methyl group migration to **H**, the precursor of the fungal compound phomacta‐1(14),3,7‐triene for which recently a type I terpene synthase was discovered.[^52^, ^53^] An intramolecular 1,7‐proton transfer to **I** may then be followed by an unusual 7,17‐cyclisation to **J**, a 1,2‐hydride shift to **K**, a 2,14‐cyclisation to **L** and deprotonation to yield **8**. The DFT calculations revealed an overall smooth energy profile for this biosynthetic process, but also uncovered a surprising direct transformation from **H** to **J** with skipping of the intermediate **I**, which shows with 13.9 kcal/mol the highest activation barrier and is thus the rate‐limiting step towards **8**. The conversion of **H** into **J** can be understood as a direct addition of a C−H bond of Me17 to the olefinic C6=C7 bond. This unusual step was further investigated through an isotopic labelling experiment. For this purpose, (4,4,4,5,5,5‐^2^H_6_)DMAPP was synthesised from (^2^H_6_)acetone (Scheme S1) and incubated with (2‐^13^C)IPP and GGPPS to obtain (2,6‐^13^C_2_,16,16,16,17,17,17‐^2^H_6_)GGPP. Its conversion with TxS‐G606A resulted in a strongly reduced production of labelled **8** in comparison to the production from unlabelled GGPP (Figure S34), which is explained by the large computed kinetic isotope effect of *k*_H_/*k*_D_=7.2 (Scheme 4A). As a consequence, the expected triplet signal in the ^13^C NMR (as a result of ^13^C‐^2^H spin coupling) for the ^13^C^2^H^1^H group (C6) of **8** formed by the deuterium migration from C17 to C6 was not observed. However, GC/MS analysis of the product revealed that all six deuterium atoms of the substrate remained in the product **8** (Figure S34), giving experimental support for the C17‐to‐C6 proton transfer. The pronounced kinetic isotope effect for the **H**‐to‐**J** transformation in the biosynthesis of **8** compares to smaller computed values for the 1,5‐proton shifts in the biosynthesis of **1** (Scheme 4B–D).

Based on isotopic labelling experiments, a similar 1,7‐proton shift has also been suggested for the biosynthesis of cephalotene, the product of the diterpene synthase CsCTS from *Cephalotaxus sinensis*, whose biosynthesis also proceeds through intermediate **J**, then branching out to **M** and **N** (Scheme 1).^[54]^ The results from the labelling experiments provided in this previous and in the present study support analogous long range proton migrations in the biosynthesis of **8** and cephalotene, while our DFT calculations clarify the unique mechanism for the formation of **J**.

**Scheme 5:**
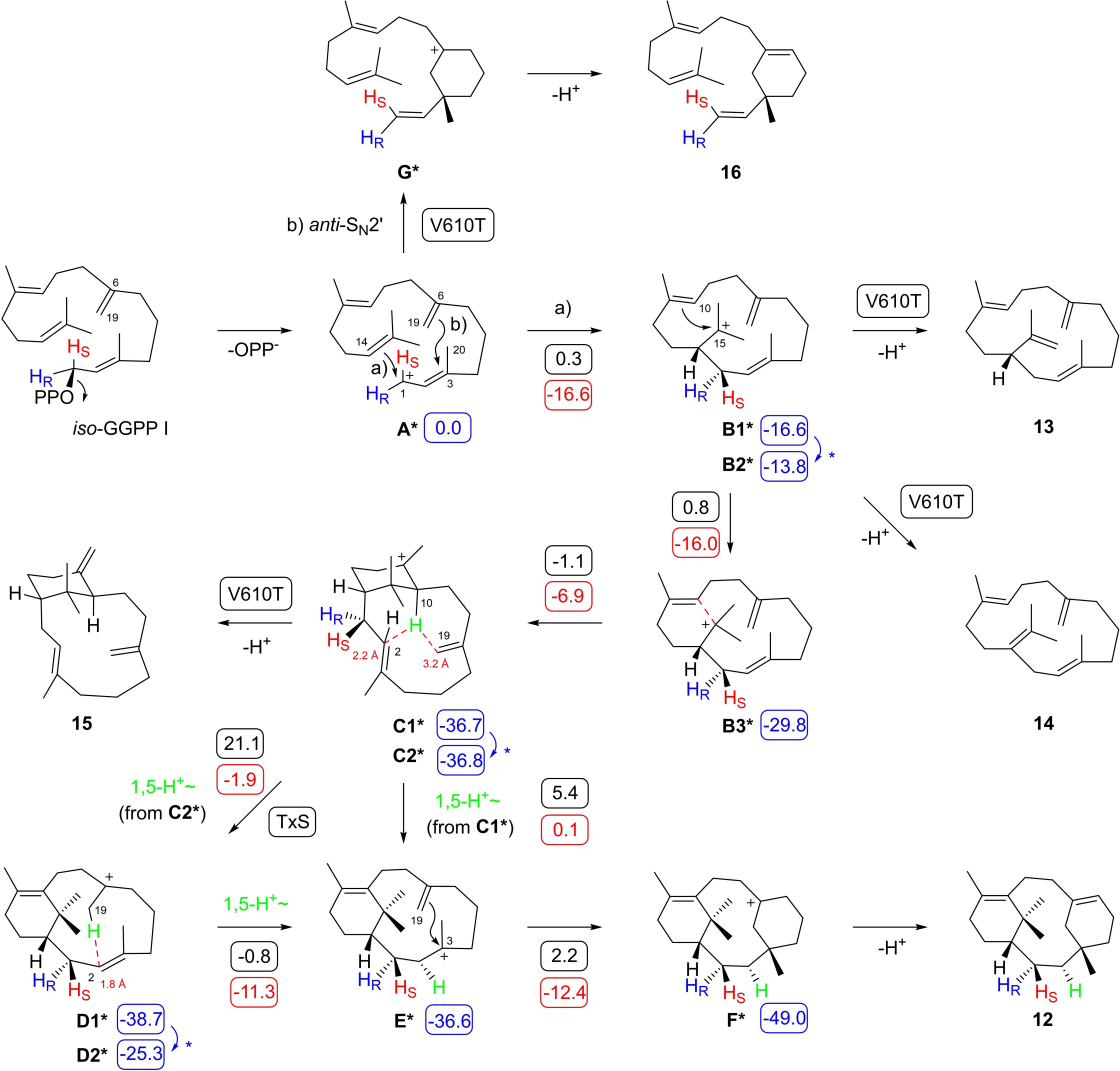
Mechanism for the cyclisation of *iso*‐GGPP I to **12** by TxS. The numbers in boxes are computed energies (in kcal/mol) of intermediates relative to A* (set to 0.0 kcal/mol, blue), reaction barriers (Gibbs free energies of activation at 298.15 K, black) and Gibbs free reaction energies (red) relative to each preceeding intermediate. All structures were computed using the mPW1PW91/6‐311+G(d,p)//B97D3/6‐31 G(d,p) method (298 K). Asterisks indicate conformational changes needed between the product of one transformation and the starting structure of the next step. All carbon numbers follow GGPP numbering (Scheme 1), which is different to the accepted carbon numbering of taxanes.^[15]^

We have recently demonstrated the efficient conversion of *iso*‐GGPP I, an isomer of GGPP with the C6=C7 double bond shifted into the C7=C19 position, with a large variety of diterpene synthases. The minor structural changes in *iso*‐GGPP I have resulted in unexpected reactivities leading to the formation of many new compounds with previously unknown skeletons.[^55^, ^56^, ^57^] The conversion of *iso*‐GGPP I with TxS yielded the novel compound taxaxenene (**12**) as the major product (Figure S35) that was isolated and structurally characterised by NMR spectroscopy (Table S12, Figures S36–S43). Xenenes are a family of compounds from *iso*‐GGPP I formed through a cyclisation mechanism with participation of the C6=C19 double bond, generally leading to compounds that are foreign to the enzyme used for their preparation (gr. ξϵνοσ=foreign). The formation of **12** from *iso*‐GGPP I requires substrate ionisation to A*, followed by 1,14‐ and 10,15‐cyclisations to B* and C* (Scheme 5). At this stage a direct 1,5‐proton shift can lead to D* that can undergo 3,19‐cyclisation to F* and deprotonation to **12**. Similar to the situation in the biosynthesis of **1**, the direct 1,5‐proton transfer from C* to D* may be substituted by two sequential 1,5‐proton shifts mediated through E*.

The biosynthesis of **12** was investigated through isotopic labelling experiments. The incubation of *iso*‐GGPP I with TxS in a D_2_O buffer showed no incorporation of deuterium (Figure S44), suggesting that the cyclisation cascade does not proceed through a neutral intermediate. To investigate the intramolecular proton shifts, (10‐^2^H)‐*iso*‐GGPP I was synthesised (Scheme S2) and converted with TxS, followed by the isolation of labelled **12**. The deuterium in the substrate may migrate directly to C2 in which case one of the signals for the hydrogens at C2 (H2α) should be vanished in the HSQC spectrum. On the contrary, if deuterium would first migrate to C19, a distribution of the three homotopic hydrogens in the Me group would be expected and a kinetic isotope effect should result in the preferential migration of a proton from C19 to C2. In this case crosspeaks for both hydrogens at C2 would be expected in the HSQC spectrum. For the unlabelled compound in C_6_D_6_ at 298 K one of the C2 crosspeaks overlaps with a strong signal for Me19, but after a change of the solvent and measuring the spectra at 238 K the signals were resolved. Analysis of the sample obtained with TxS from (10‐^2^H)‐*iso*‐GGPP revealed a vanished crosspeak for H2α (Figure S45), giving first experimental evidence for the proton transfer from C10 to C2 in a system that is structurally closely related to the natural taxadiene system. Most computational studies have favoured a two‐step proton transfer from C10 to C6 using C2 as an intermediate springboard in the biosynthesis of **1**, but conclusive experimental evidence for this mechanistic hypothesis as provided here is difficult to obtain.

These experimental findings are also in line with computational data for the conversion of *iso*‐GGPP I into **12** (Scheme 5, Table S13 and Figure S46). DFT calculations revealed a low reaction barrier of 5.5 kcal/mol for the direct conversion of C1* into E*, while the step from C2*, a conformer of C1*, to D* exhibits a reaction barrier of 21.1 kcal/mol. All other transformations proceed through low reaction barriers and the overall cyclisation cascade is with −49.0 kcal/mol highly exergonic.

The V610T variant of TxS was selected for further study with *iso*‐GGPP I as substrate, because this variant showed the best diterpene production of all variants created in this study. In addition, the V610T variant has a completely altered product profile in comparison to wild‐type TxS, suggesting that the formation of products different to **12** may be expected from this variant. The incubation of *iso*‐GGPP I with TxS‐V610T resulted in the formation of four main compounds besides several side products (Figure S47). The main compounds were isolated and structurally characterised as isocembrene A2 (**13**), isocembrene C (**14**), verticilla‐3,8(19),12(18)‐triene (**15**) and taxasimplene (**16**) that are all new diterpenoid hydrocarbons (Tables S14–S17, Figures S48–S79). Compounds **13–15** are explainable as the deprotonation products of the cationic intermediates B1* and C1*, while **16** requires a change in the initial cyclisation mode from the usually observed 1,14‐ to a 3,19‐cyclisation, leading to the direct precursor G*.

The absolute configurations of terpenes can be determined through chemical correlation using stereoselectively deuterated terpene precursors. To investigate the absolute configurations of **12**, **15** and **16**, the stereoselectively deuterated compounds (*R*)‐ and (*S*)‐(1‐^13^C,1‐^2^H)‐*iso*‐GGPP I^[56]^ were converted with TxS‐V610T. In these reactions stereogenic centers of known configuration are introduced at the labelled carbons, allowing to conclude on the absolute configuration of the products by determination of the relative orientation. For **12** and **15** this approach uncovered the absolute configurations as shown in Scheme 5 (Figures S80 and S81). For compound **16** the argumentation is more complex: Starting from the usual conformational fold of *iso*‐GGPP I that is analogous to the fold of GGPP leading to taxa‐4,11‐diene, diphosphate will leave to the front side to allow a 1,14‐cyclisation of A* to B1* with inversion of configuration at C1 (S_N_2 reaction), or a 3,19‐cyclisation to G* with attack at C3 from the *Re* face (back side, *anti*‐S_N_2’ reaction). This process necessarily turns the 1‐*pro*‐*R* hydrogen at C1 of *iso*‐GGPP I into the (*E*)‐position of the vinyl group of **16** and the 1‐*pro*‐*S* hydrogen into the (*Z*)‐position. For a substrate analog a conformational change may be possible, but for the minor structural differences between GGPP and *iso*‐GGPP I the overall conformational fold should be similar. Eventually, a conformational change in the C1‐C3 portion with Me20 pointing down may be possible (Scheme S3). In this case C3 would be attacked from the *Si* face, leading to the enantiomer of **16**, but such a cyclisation must proceed with the 1‐*pro*‐*R* hydrogen at C1 of *iso*‐GGPP I turning into the (*Z*)‐position of the vinyl group of **16** and the 1‐*pro*‐*S* hydrogen into the (*E*)‐position. This analysis demonstrates that the stereochemical fate of the hydrogens at C1 of *iso*‐GGPP I can be taken as an indicator for the absolute configuration of **16**, and the experiment demonstrates that **16** has the absolute configuration as shown in Scheme 5 (Figure S82).

## Conclusions

Long range proton shifts in the biosynthesis of taxa‐4,11‐diene (**1**) have extensively been discussed in the literature, first by suggesting a direct transfer from C10 to C6,^[21]^ and later, based on computational data, a two step migration from C10 to C2 and then further to C6 was raised.[^23^, ^24^] In the present study we have experimentally and computationally uncovered that similar long range hydrogen migrations are also relevant in the high energy process of the mass spectrometric fragmentation reaction towards the base peak ion of **1**, demonstrating that long distance hydrogen shifts are part of the intrinsic reactivity of the unique taxane skeleton. Site‐directed mutagenesis experiments resulted in the discovery of a novel product from the G606A variant that was named cyclophomactene (**8**). This compound is formed through an unprecedented concerted process combining a 1,7‐proton shift and ring closure that can also be described as the addition of a methylcarbinyl cation to an olefin, again revealing the relevance of long range proton transfers in taxanes and closely related systems. Further insights were obtained through the enzymatic conversion of *iso*‐GGPP I with a double bond shifted from C6=C7 to C7=C19. In this substrate analog C6 is unreactive and thus gave first experimental evidence for a migration of H10 to C2, supported through the labelling experiments in conjunction with DFT calculations performed in this study. The downstream steps ultimately lead to taxaxenene (**12**), a compound with a novel skeleton that cannot be formed naturally from GGPP. The V610T substitution in TxS strongly interferes with long distance proton migrations, as not only with GGPP the cyclisation cascade was interrupted at intermediate **C**, resulting only in cembrane and verticillane diterpenes, but – despite the formation of several interesting compounds – also with *iso*‐GGPP I no products beyond the analogous intermediate C* were obtained.

Long distance proton migrations are not limited to taxa‐4,11‐diene biosynthesis, but were also invoked for the biosynthesis of trichodiene^[58]^ and asperfumene.^[59]^ Based on DFT calculations two sequential proton shifts have also been proposed for the biosynthesis of fusicocca‐2,19(14)‐diene.^[60]^ While the results of computational studies are generally trustable, experimental evidence is lacking for fusicocca‐2,19(14)‐diene biosynthesis, but may be obtained through a similar approach as described here.

## Conflict of Interests

The authors declare no conflict of interest.

1

## Supporting information

As a service to our authors and readers, this journal provides supporting information supplied by the authors. Such materials are peer reviewed and may be re‐organized for online delivery, but are not copy‐edited or typeset. Technical support issues arising from supporting information (other than missing files) should be addressed to the authors.

Supporting Information

## Data Availability

The data that support the findings of this study are available in the supplementary material of this article.
