# Supplementary material for: On the Role of Hydrogen Migrations in the Taxadiene System
Source: Angew Chem Int Ed Engl. 2025 Jan 10;64(13):e202422788. doi: 10.1002/anie.202422788 (PMC11933516; doi:10.1002/anie.202422788)
Supplement: Supplementary file 1 — Supporting Information [file ANIE-64-e202422788-s001.pdf]

Angewandte Chemie
Eine Zeitschrift der Gesellschaft Deutscher Chemiker

Supporting Information

On the Role of Hydrogen Migrations in the Taxadiene System

*H. Li, B. Goldfuss, J. S. Dickschat**

Table of Contents

Analytical methods	2
Buffers, gene cloning and expression, protein purification	3
Incubation experiments with labelled substrates	4
Spectroscopic and physical data of isolated compounds	5
Comparison of measured and previously published NMR data of **1**	7
NMR spectra of **1**	8
NMR data of **2**	15
Stereoselective deuteration experiments for **1**	16
Individual carbon labelling experiments for **1** and **2**	18
EI mass spectra of unlabelled and labelled **1**	20
Computational methods	23
Computational data for the EI-MS fragmentation of **1**	24
The long range hydrogen migration in the formation of the base peak ion of **1**	27
Site-directed mutagenesis of TxS	28
GC/MS analysis of products from TxS and its variants	32
Structural interpretation of the results obtained with the E583 enzyme variants	33
Structure elucidation and NMR data of **8**	34
Structural interpretation of the results obtained with the I848 enzyme variants	42
NMR data of **7**	43
Computational data for the cyclisation mechanism to **8**	44
Synthesis of (4,4,4,5,5,5-$^{2}H_{6}$)DMAPP	46
The 1,7-proton shift from **H** to **I** in the biosynthesis of **8**	48
GC/MS analysis of the products obtained with TxS from *iso*-GGPP I	49
Structure elucidation and NMR data of **12**	50
Biosynthesis of **12** in D_2O buffer	58
Synthesis of (10-^{2}H)-*iso*-GGPP I	59
Proton shift in the biosynthesis of **12**	63
Computational data for the cyclisation mechanism to **12** – **15**	64
GC/MS analysis of products from incubation of *iso*-GGPP I and TxS-V610T	66
Structure elucidation and NMR data of **13**	67
Structure elucidation and NMR data of **14**	75
Structure elucidation and NMR data of **15**	83
Structure elucidation and NMR data of **16**	91
The absolute configurations of **12**, **15** and **16**	99
Cartesian coordinates of computed structures	103
References	180

General methods

Chemicals were purchased from Sigma Aldrich Chemie GmbH (Steinheim, Germany), Carbolution Chemicals GmbH (St. Ingbert, Germany), or Carl Roth (Karlsruhe, Germany) and used without purification. Solvents for column chromatography were purchased in p.a. grade and purified by distillation. Thin-layer chromatography (TLC) was performed with 0.2 mm precoated plastic sheets Polygram Sil G/UV254 purchased from Machery-Nagel (Düren, Germany). Column chromatography was performed using silica gel 60 purchased from Merck (Darmstadt, Germany). For the activation of TLC with $AgNO_3$, it was soaked in a solution of $AgNO_3$ (2.5 g) in methanol (150 mL) 10 min, followed by rigorous evaporation of the solvent.

GC/MS

GC/MS analyses were performed on a 5977A GC/MSD system (Agilent, Santa Clara, CA, USA) with a 7890B GC and a 5977A mass selective detector. The GC was equipped with a HP5-MS fused silica capillary column (30 m, 0.25 mm i. d., 0.50 μm film). Specific GC settings were 1) inlet pressure: 77.1 kPa, He at 23.3 mL min^{-1}, 2) injection volume: 1 μL, 3) temperature program: 5 min at 50 °C increasing at 10 °C min^{-1} to 320 °C, 4) 60 s valve time, and 5) carrier gas: He at 1.2 mL min^{-1}. MS settings were 1) source: 230 °C, 2) transfer line: 250 °C, 3) quadrupole: 150 °C and 4) electron energy: 70 eV. Retention indices (*I*) were determined from retention times in comparison to the retention times of *n*-alkanes (C_7-C_{40}).

HRMS

High resolution mass spectra using APCI were recorded on an Orbitrap XL instrument (Thermo Fisher Scientific, Waltham, MA, USA) or using a 7890B/7200 series gas chromatography/accurate mass Q-ToF detector system (Agilent). The GC was equipped with a HP5-MS fused silica capillary column (30 m, 0.25 mm i. d., 0.50 μm film). GC settings were 1) injection volume: 1 μL, 2) temperature program: 5 min at 50 °C, increasing 10 °C min^{-1} to 320 °C, 3) split ratio: 5:1, 60 s valve time and 4) carrier gas flow: He at 1 mL min^{-1}. MS settings were 1) inlet pressure: 83.2 kPa, He flow at 24.6 mL min^{-1}, 2) transfer line temperature: 250 °C, 3) ionization energy: 70 eV.

NMR spectroscopy

NMR spectra were recorded on a Bruker (Billerica, MA, USA) Avance I (300 MHz), Avance I (400 MHz), Avance I (500 MHz), Avance III HD Prodigy (500 MHz) or an Avance III HD Cryo (700 MHz) NMR spectrometer. Spectra were measured in C_6D_6 and referenced against solvent signals (^{1}H-NMR, residual proton signal: δ = 7.16; ^{13}C-NMR: δ = 128.06).[1]

IR spectroscopy

IR spectra were recorded on a Bruker α infrared spectrometer with a diamond ATR probehead. Peak intensities are given as s (strong), m (medium), w (weak) and br (broad).

Optical rotations

Optical rotations were recorded on a Modular Compact Polarimeter MCP 100 (Anton Paar, Graz, Austria). The temperature setting was 25 °C; the wavelength of the light used was 589 nm (sodium D line); the path-length was 10 cm; the compound concentrations *c* are given in g 100 mL^{-1}.

Buffers

The buffers used for protein purification and incubation experiments were: binding buffer (20 mM Na_2HPO_4, 500 mM NaCl, 20 mM imidazole, 1 mM $MgCl_2$, pH = 7.4), washing buffer (20 mM Na_2HPO_4, 500 mM NaCl, 100 mM imidazole, 1 mM $MgCl_2$, pH = 7.4), elution buffer (20 mM Na_2HPO_4, 500 mM NaCl, 500 mM imidazole, 1 mM $MgCl_2$, pH = 7.4), incubation buffer (50 mM Tris/HCl, 10 mM $MgCl_2$, 10% glycerol, 20 mM β-cyclodextrin, pH = 8.2) and substrate buffer (25 mM aq. NH_4HCO_3).

Gene expression and purification of TxS and variants

A small scale culture of *Escherichia coli* BL21 (DE3) transformed with the plasmid pYE-TxS was grown in LB medium (10 mL) containing kanamycin sulfate (50 mg L^{-1}) overnight with shaking at 37 °C. A large scale expression culture in LB medium (1 L) containing kanamycin sulfate was inoculated with the grown overnight culture (2%). Culturing was continued with shaking at 37 °C until an OD_{600} = 0.4 – 0.6 was reached. After cooling the culture to 18 °C, enzyme expression was induced by the addition of IPTG solution (100 mM, 1 ‰). The cultures were shaken at 18 °C for 18 h. Cells were harvested via centrifugation (1,500 g, 40 min, 4 °C), resuspended in binding buffer (30 mL, 4 °C) and lysed by ultrasonication (10x 1 min) on ice. The cell debris was removed by centrifugation (14,600 g, 7 min, 4 °C) and the supernatant was loaded on a Ni^{2+}-NTA superflow affinity chromatography column (Qiagen, Venlo, Netherlands) equilibrated with binding buffer. The column was washed with binding buffer (2 column volumes, CV, 4 °C) and washing buffer (2 CV, 4 °C). The desired protein was eluted with elution buffer (1 CV, 4 °C). TxS variants for which the construction is detailed below, were expressed and purified through the same procedure. Protein purity was checked by SDS-PAGE analysis (Figure S1) and protein concentrations were determined through Bradford assay.[2] All protein concentrations were adjusted to 1.0 mg mL^{-1} through dilution with incubation buffer for incubation experiments.

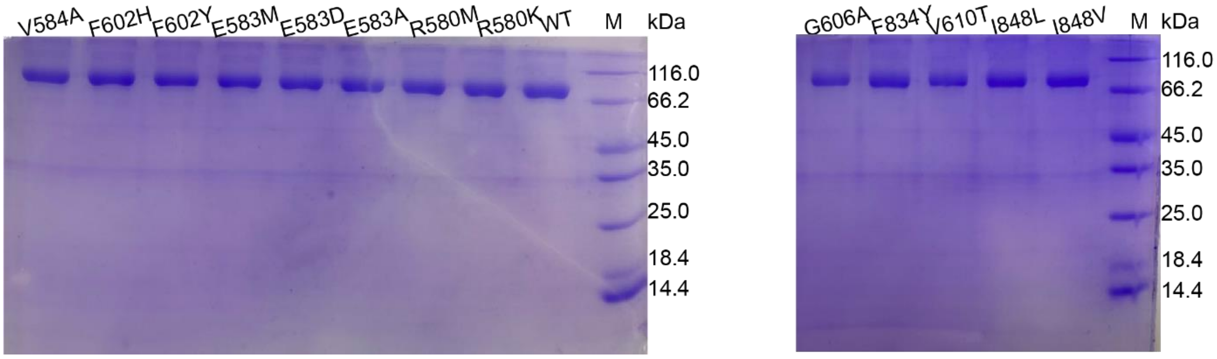

Figure S1. SDS-PAGE analysis of purified recombinant TxS and its enzyme variants.

Incubation experiments with labelled substrates
Isotopic labelling experiments were performed with the precursors of labelled GGPP (ca. 1 mg in 1 mL 25 mM NH_4HCO_3), incubation buffer (5 mL) and preparations of purified enzymes (each 1 mL) as listed in Table S1. After incubation at 28 °C overnight, the products were extracted with C_6D_6 (600 µL + 300 µL) or *n*-hexane (500 µL), then the extracts were dried with $MgSO_4$ and analysed by NMR and/or GC/MS.

Table S1. Labelling experiments performed with TxS in this study.

entry	substrates	enzymes	results shown in
1	(*R*)-(1-^{13}C,1-^{2}H)IPP[3]	TxS,[4] IDI,[5] GGPPS[6]	Figure S11
2	(*S*)-(1-^{13}C,1-^{2}H)IPP[3]	TxS,[4] IDI,[5] GGPPS[6]	Figure S11
3	DMAPP, (*E*)-(4-^{13}C,4-^{2}H)IPP[7]	TxS,[4] GGPPS[6]	Figures S12 and S15
4	DMAPP, (*Z*)-(4-^{13}C,4-^{2}H)IPP[7]	TxS,[4] GGPPS[6]	Figures S12 and S15
5	(1-^{13}C)GGPP[8]	TxS[4]	Figures S13 and S16
6	(2-^{13}C)GGPP[6]	TxS[4]	Figures S13 and S16
7	(3-^{13}C)GGPP[8]	TxS[4]	Figures S13 and S16
8	(4-^{13}C)GGPP[8]	TxS[4]	Figures S13 and S16
9	(1-^{13}C)FPP,[9] IPP	TxS,[4] GGPPS[6]	Figures S13 and S16
10	(2-^{13}C)FPP,[9] IPP	TxS,[4] GGPPS[6]	Figures S13 and S16
11	(3-^{13}C)FPP,[9] IPP	TxS,[4] GGPPS[6]	Figures S13 and S16
12	(4-^{13}C)FPP,[9] IPP	TxS,[4] GGPPS[6]	Figures S13 and S16
13	(1-^{13}C)GPP,[7] IPP	TxS,[4] GGPPS[6]	Figures S13 and S16
14	(6-^{13}C)FPP,[9] IPP	TxS,[4] GGPPS[6]	Figures S13 and S16
15	(7-^{13}C)FPP,[9] IPP	TxS,[4] GGPPS[6]	Figures S14 and S17
16	(8-^{13}C)FPP,[9] IPP	TxS,[4] GGPPS[6]	Figures S14 and S17
17	(9-^{13}C)FPP,[9] IPP	TxS,[4] GGPPS[6]	Figures S14 and S17
18	(10-^{13}C)FPP,[9] IPP	TxS,[4] GGPPS[6]	Figures S14 and S17
19	(11-^{13}C)FPP,[9] IPP	TxS,[4] GGPPS[6]	Figures S14 and S17
20	(12-^{13}C)FPP,[9] IPP	TxS,[4] GGPPS[6]	Figures S14 and S17
21	(9-^{13}C)GPP,[10] IPP	TxS,[4] GGPPS[6]	Figures S14 and S17
22	(14-^{13}C)FPP,[9] IPP	TxS,[4] GGPPS[6]	Figures S14 and S17
23	(15-^{13}C)FPP,[10] IPP	TxS,[4] GGPPS[6]	Figures S14 and S17
24	(20-^{13}C)GGPP[6]	TxS[4]	Figures S14 and S17
25	(2-^{2}H)GGPP[11]	TxS[4]	Figure S20
26	*iso*-GGPP I[12] in D_2O	TxS[4]	Figure S44
27	(10-^{2}H)-*iso*-GGPP I	TxS[4]	Figure S45
28	(*R*)-(1-^{13}C,1-^{2}H)-*iso*-GGPP I[13]	TxS[4]	Figure S80
29	(*S*)-(1-^{13}C,1-^{2}H)-*iso*-GGPP I[13]	TxS[4]	Figure S80
30	(*R*)-(1-^{13}C,1-^{2}H)-*iso*-GGPP I[13]	TxS-V610T	Figures S81 and S82
31	(*S*)-(1-^{13}C,1-^{2}H)-*iso*-GGPP I[13]	TxS-V610T	Figures S81 and S82

Table S2. Preparative scale conversions with TxS and enzyme variants.

enzymes	substrate	scale	isolated compounds
TxS	GGPP	80 mg (160 μmol)	**1**, **2**
TxS	*iso*-GGPP I	80 mg (160 μmol)	**12**
TxS-G606A	GGPP	80 mg (160 μmol)	**4**, **8**
TxS-V610T	GGPP	80 mg (160 μmol)	**4**, **6**, **7**
TxS-V610T	*iso*-GGPP I	80 mg (160 μmol)	**13**, **14**, **15**, **16**

Preparative scale incubations with TxS and its variants and compound isolations
For preparative scale enzymatic conversions (Table S2), a solution of the trisammonium salts of GGPP or *iso*-GGPP I (80 mg, 160 μmol) in substrate buffer (20 mL) was added to incubation buffer (130 mL) containing enzymes (0.3 mg L^{-1}), followed by incubation for 16 h at 30 °C. The reaction mixtures were extracted with n-hexane, the extracts were dried with $MgSO_4$ and the solvent was evaporated, followed by compound isolation through column chromatography on silica gel or on silica gel activated with $AgNO_3$. For the activation of silica gel with $AgNO_3$, 50 g of silica gel were suspended in a solution of $AgNO_3$ (2.5 g) in methanol (150 mL) overnight, followed by rigorous evaporation of the solvent.

Taxa-4,11-diene (**1**).[14] Yield: 2.0 mg (7.3 μmol, 4.6%), TLC (pentane): R_f = 0.83. GC (HP-5): *I* = 2065. IR (diamond ATR): $\tilde{\nu}$ / cm^{-1} = 3396 (br), 2956 (s), 2920 (s), 2853 (s), 1718 (w), 1456 (m), 1364 (m), 1259 (w), 1085 (m), 1015 (m), 798 (w). HR-MS (APCI): calc. for $[C_{20}H_{33}]^+$ *m*/*z* = 273.2577; found: *m*/*z* = 273. 2574. Optical rotation: $[\alpha]_D^{25}$ = +197.8 (*c* 0.09, CH_2Cl_2). lit. for (+)-**1**: $[\alpha]_D$ = +170.[15] NMR data given in Table S3.

Taxa-4(20),11-diene (**2**). Yield: 0.5 mg (1.84 μmol, 0.5%), TLC (pentane): R_f = 0.85. GC (HP-5): *I* = 2013. IR (diamond ATR): $\tilde{\nu}$ / cm^{-1} = 3363 (br), 3011 (m), 2981 (s), 2927 (s), 2851 (s), 1643 (w), 1460 (m), 1441 (m), 1376 (w), 1285 (w), 1088 (w), 880 (m), 805 (w). HR-MS (EI): calc. for $[C_{20}H_{32}]^+$ *m*/*z* = 272.2499; found: *m*/*z* = 272. 2503. Optical rotation: $[\alpha]_D^{25}$ = +142.5 (*c* 0.09, CH_2Cl_2). NMR data given in Table S4.

Verticilla-3,7,12-triene (**4**).[16] Yield: 0.2 mg (0.7 μmol, 0.4%), TLC (pentane): R_f = 0.52. GC (HP-5): *I* = 2062. IR (diamond ATR): $\tilde{\nu}$ / cm^{-1} = 3364 (w), 3196 (w), 2961 (m), 2922 (s), 2870 (m), 2852 (s), 2058 (w), 1991 (w), 1733 (w), 1659 (w), 1632 (w), 1459 (w), 1438 (w), 1260 (w), 1098 (w), 1079 (w), 1025 (w), 805 (w). HR-MS (APCI): calc. for $[C_{20}H_{33}]^+$ *m*/*z* = 273.2577; found: *m*/*z* = 273.2570. Optical rotation: $[\alpha]_D^{25}$ = +70.0 (*c* 0.02, acetone).

Cembrene A (**6**). Yield: 0.7 mg (2.6 μmol, 1.6%), TLC ($AgNO_3$ activated TLC plate, pentane:Et_2O = 8:1): R_f = 0.38. GC (HP-5): *I* = 1986. IR (diamond ATR): $\tilde{\nu}$ / cm^{-1} = 2923 (s), 2853 (m), 1663 (w), 1643 (w), 1444 (m), 1374 (w), 887 (m), 847 (w), 807 (w). HR-MS (APCI): calc. for $[C_{20}H_{33}]^+$ *m*/*z* = 273.2577; found: *m*/*z* = 273.2580. Optical rotation: $[\alpha]_D^{25}$ = +5.0 (*c* 0.02, acetone), lit. for (*S*)-(+)-**6**: $[\alpha]_D$ = +19.5.[17]

Cembrene D (**7**).[18] Yield: 0.2 mg (0.7 μmol, 0.4%), TLC ($AgNO_3$ activated TLC plate, pentane:Et_2O = 8:1): R_f = 0.10. GC (HP-5): *I* = 2049. IR (diamond ATR): $\tilde{\nu}$ / cm^{-1} = 2955 (s), 2924 (s), 2852 (s), 1731 (m), 1466 (w), 1366 (w), 1140 (w), 775 (w). HR-MS (APCI): calc. for $[C_{20}H_{33}]^+$ *m*/*z* = 273.2577; found: *m*/*z* = 273.2574. NMR data given in Table S10.

Cyclophomactene (**8**). Yield: 0.2 mg (0.7 μmol, 0.4%), TLC (pentane): R_f = 0.86. GC (HP-5): *I* = 2077. IR (diamond ATR): $\tilde{\nu}$ / cm^{-1} = 2956 (m), 2924 (s), 2854 (m), 1657 (w), 1635 (w), 1467 (w), 1375 (w), 1259 (w), 1015 (w), 880 (w). HR-MS (EI): calc. for $[C_{20}H_{32}]^+$ *m*/*z* = 272.2499; found: *m*/*z* = 272.2501. Optical rotation: $[\alpha]_D^{25}$ = +85.0 (*c* 0.02, CH_2Cl_2). NMR data given in Table S9.

Taxaxenene (**12**). Yield: 0.8 mg (2.9 μmol, 1.8%), TLC (pentane): R_f = 0.74. GC (HP-5): *I* = 2047. IR (diamond ATR): $\tilde{\nu}$ / cm^{-1} = 2944 (s), 2910 (s), 2865 (s), 2832 (m), 1737 (w), 1699 (w), 1459 (m), 1385 (w), 1373 (w), 1360 (w), 1087 (w), 857 (w). HR-MS (EI): calc. for $[C_{20}H_{32}]^+$ *m*/*z* = 272.2499; found: *m*/*z* = 272.2505. Optical rotation: $[\alpha]_D^{25}$ = +61.0 (*c* 0.2, acetone). NMR data given in Table S12.

Isocembrene A2 (**13**). Yield: 1.4 mg (5.1 μmol, 3.2%), TLC (pentane): R_f = 0.48. GC (HP-5): *I* = 1991. IR (diamond ATR): $\tilde{\nu}$ / cm^{-1} = 3074 (w), 2927 (s), 2854 (m), 2163 (w), 2005 (w), 1641 (w), 1448 (m), 1380 (w), 1261 (w), 886 (m). HR-MS (EI): calc. for $[C_{20}H_{32}]^+$ *m*/*z* = 272.2499; found: *m*/*z* = 272.2498. Optical rotation: $[\alpha]_D^{25}$ = –0.7 (*c* 0.14, acetone). NMR data given in Table S14.

Isocembrene C (**14**). Yield: 1.3 mg (4.8 μmol, 3%), TLC (pentane): R_f = 0.59. GC (HP-5): *I* = 2062. IR (diamond ATR): $\tilde{\nu}$ / cm^{-1} = 3069 (w), 2924 (s), 2855 (m), 1735 (m), 1641 (w), 1460 (m), 1380 (w), 1180 (w), 885 (m), 848 (w). HR-MS (EI): calc. for $[C_{20}H_{32}]^+$ *m*/*z* = 272.2499; found: *m*/*z* = 272.2500. NMR data given in Table S15.

Verticilla-3,8(19),12(18)-triene (**15**). Yield: 0.7 mg (2.6 μmol, 1.6%), TLC (pentane): R_f = 0.72. GC (HP-5): *I* = 2068. IR (diamond ATR): $\tilde{\nu}$ / cm^{-1} = 3078 (w), 2924 (s), 2867 (m), 1741 (w), 1644 (w), 1443 (m), 1382 (w), 1099 (w), 884 (m). HR-MS (APCI): calc. for $[C_{20}H_{33}]^+$ *m*/*z* = 273.2577; found: *m*/*z* = 273.2580. Optical rotation: $[\alpha]_D^{25}$ = +1.0 (*c* 0.1, acetone). NMR data given in Table S16.

Taxasimplene (**16**). Yield: 0.6 mg (2.2 μmol, 1.4%), TLC (pentane): R_f = 0.40. GC (HP-5): *I* = 1906. IR (diamond ATR): $\tilde{\nu}$ / cm^{-1} = 3358 (w), 3080 (w), 2962 (m), 2921 (s), 2851 (m), 1659 (w), 1634 (w), 1448 (w), 1374 (w), 1261 (w), 1100 (w), 1036 (w), 807 (w), 724 (w). HR-MS (APCI): calc. for $[C_{20}H_{33}]^+$ *m*/*z* = 273.2577; found: *m*/*z* = 273.2569. Optical rotation: $[\alpha]_D^{25}$ = –3.3 (*c* 0.06, acetone). NMR data given in Table S17.

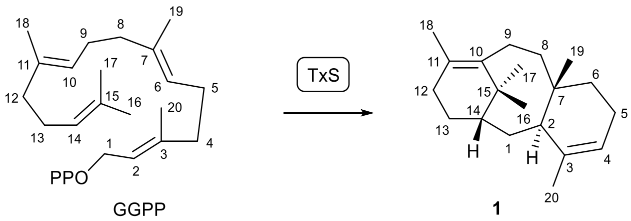

Figure S2. Carbon numbering of taxa-4,11-diene (**1**), indicating the origin of each carbon from GGPP by same number.

Table S3. NMR data of taxa-4,11-diene (**1**) in C_6D_6 recorded at 298 K.

C[a]	type	^{13}C[b]	^{1}H[b]	^{13}C[c]	^{1}H[c]
1	CH_2	28.79	1.72 (m, H_β)<br>1.63 (m, H_α)	28.4	1.72 (H_β)<br>1.62 (H_α)
2	CH	40.20	2.63 (m)	39.8	2.62
3	C_q	138.43	–	138.1	–
4	CH	121.69	5.39 (br s)	121.3	5.38
5	CH_2	23.66	2.12 (m, H_β)<br>1.95 (m, H_α)	22.7	2.11 (H_β)<br>1.94 (H_α)
6	CH_2	38.91	1.76 (m, H_α)<br>1.19 (ddt, J = 6.2, 12.5, 1.0, H_β)	38.5	1.76 (H_α)<br>1.19 (H_β)
7	C_q	37.68	–	37.3	–
8	CH_2	41.82	1.82 (m, H_β)<br>1.40 (m, H_α)	41.4	1.81 (H_β)<br>1.39 (H_α)
9	CH_2	24.89	2.64 (m, H_α)<br>2.17 (m, H_β)	24.5	2.63 (H_β)*<br>2.15 (H_α)*
10	C_q	137.91	–	137.5	–
11	C_q	129.68	–	129.3	–
12	CH_2	30.06	2.26 (m, H_β)<br>1.81 (m, H_α)	29.6	2.24 (H_β)<br>1.79 (H_α)
13	CH_2	23.10	2.04 (m, H_β)<br>1.60 (m, H_α)	23.3	2.02 (H_β)<br>1.58 (H_α)
14	CH	44.65	1.67 (m)	44.2	1.66
15	C_q	39.33	–	38.9	–
16	CH_3	31.03	1.10 (s)	30.6	1.09
17	CH_3	26.53	1.33 (s)	26.1	1.32
18	CH_3	21.57	1.62 (br s)	21.2	1.60
19	CH_3	21.95	0.93 (br s)	21.5	0.93
20	CH_3	24.30	1.73 (s)	23.9	1.73

[a] Carbon numbering as shown in Figure S2. [b] Chemical shifts δ in ppm, multiplicity: s = singlet, d = doublet, m = multiplet, coupling constants J are given in Hertz. [c] Literature data for **1** in C_6D_6 (for spin multiplicities and coupling constants cf. the original report).[19] The ^{13}C data deviate by ca. 0.4 ppm because of inaccurate peak referencing. ^{1}H NMR data that need a reassignment are indicated by asterisks.

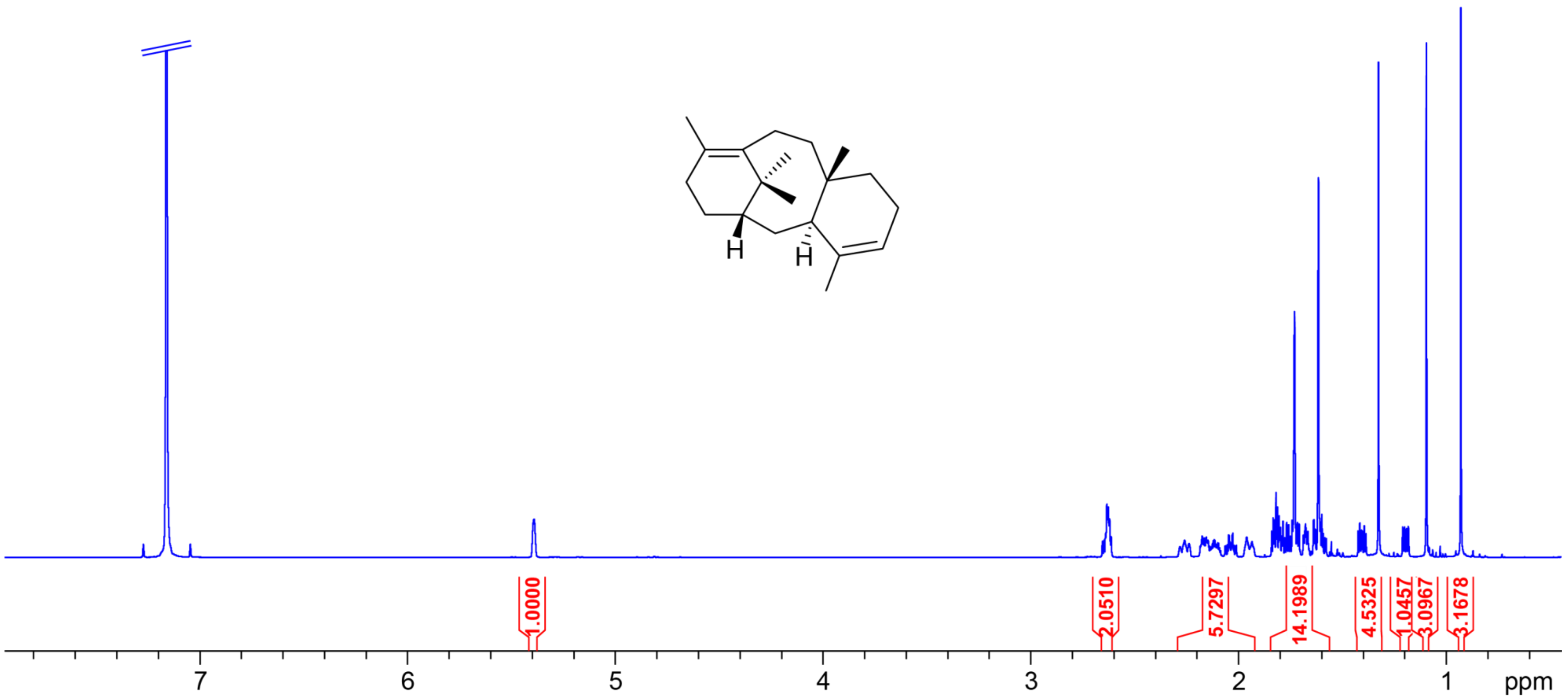

Figure S3. ^{1}H-NMR spectrum of **1** (700 MHz, C_6D_6).

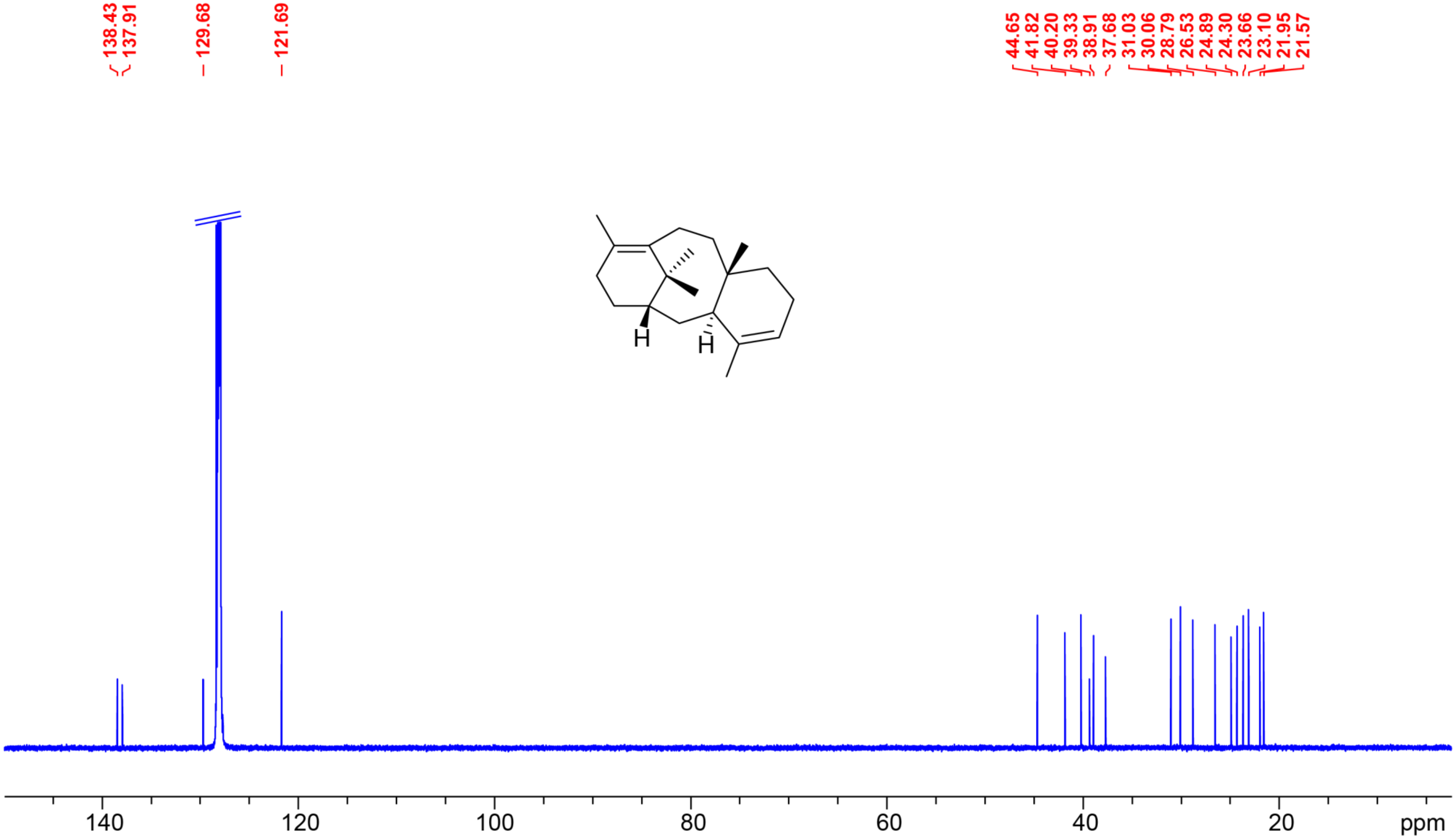

Figure S4. ^{13}C-NMR spectrum of **1** (176 MHz, C_6D_6).

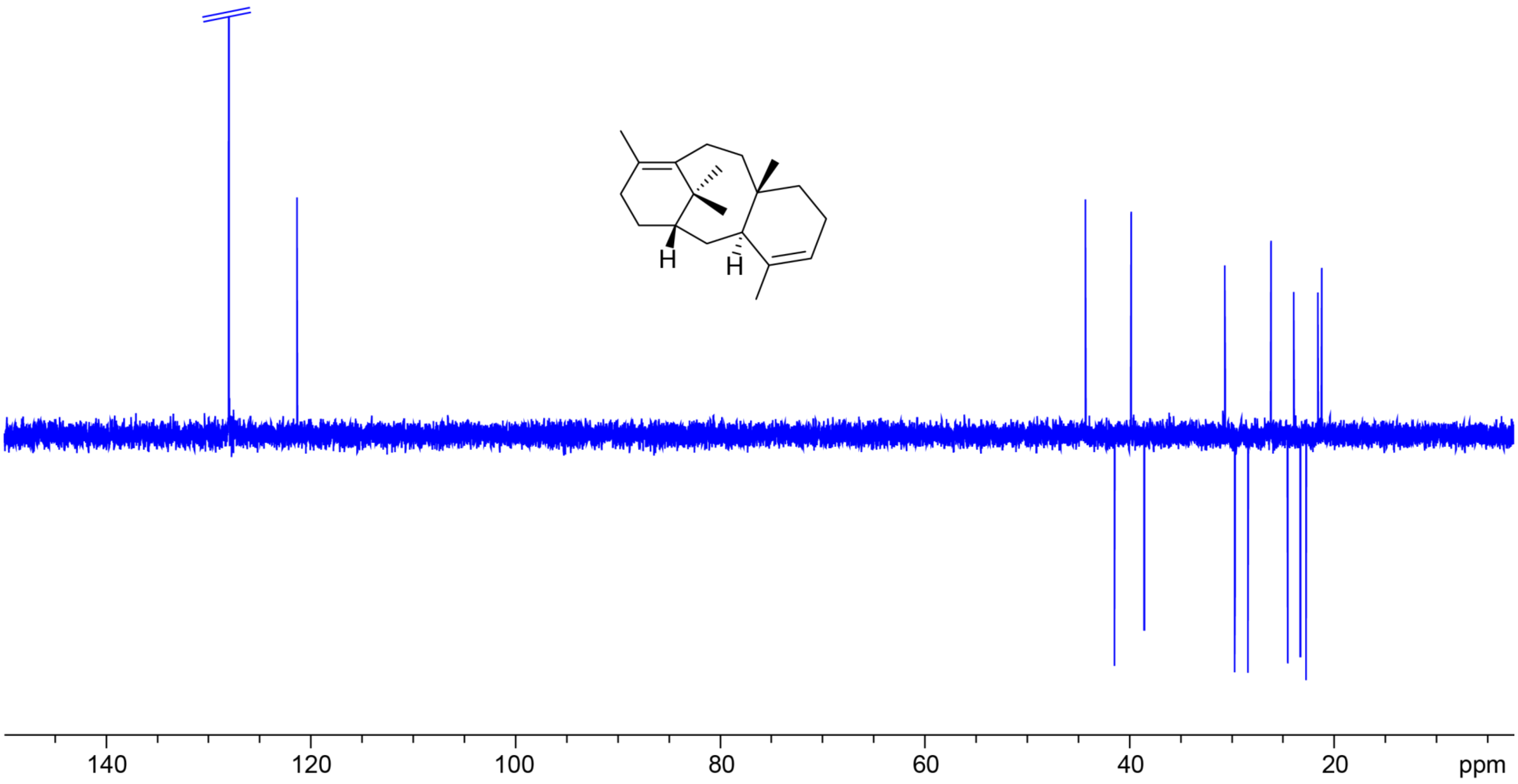

Figure S5. ^{13}C-DEPT135 spectrum of **1** (176 MHz, C_6D_6).

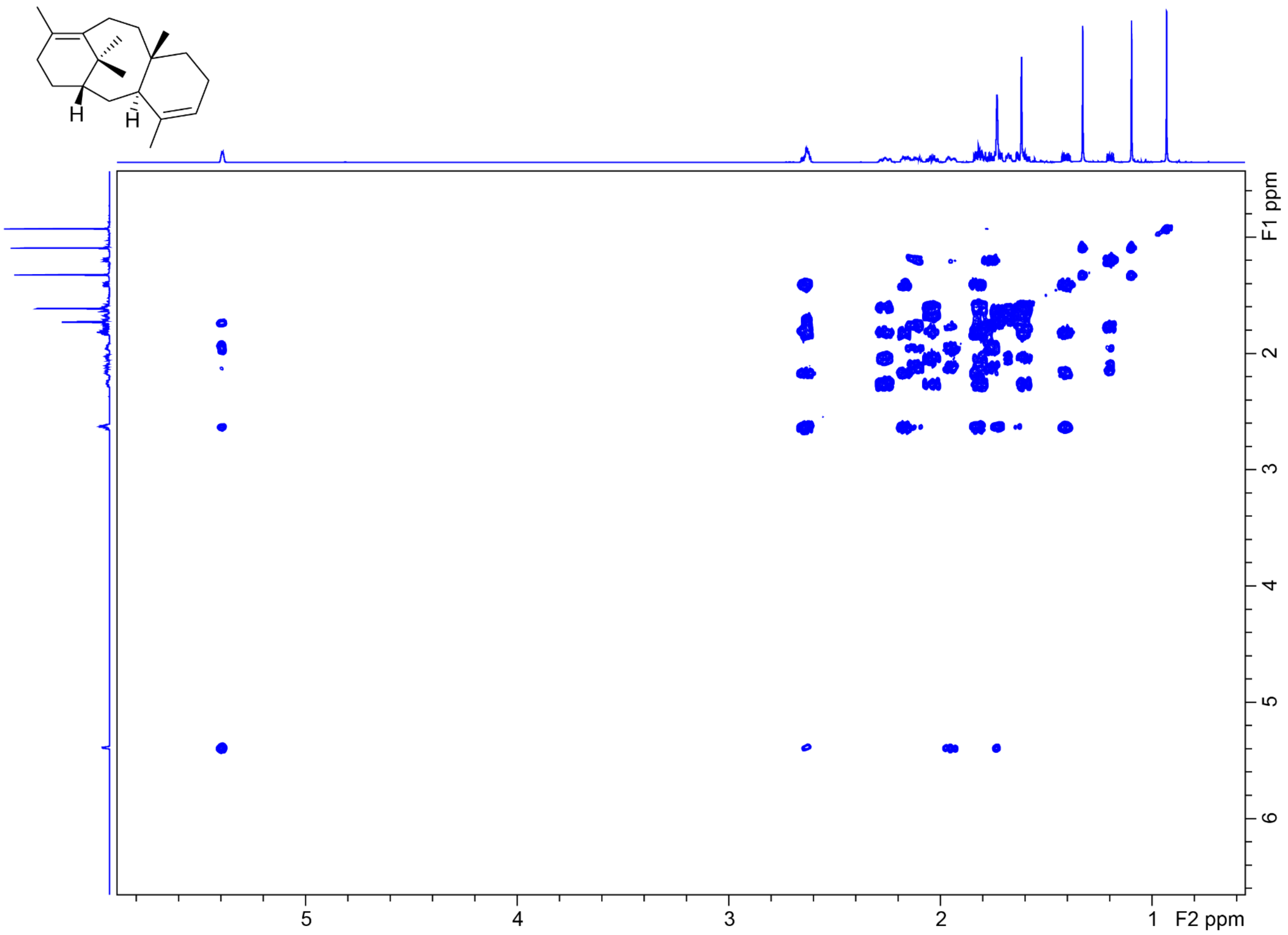

Figure S6. ^{1}H-^{1}H-COSY spectrum (C_6D_6) of **1**.

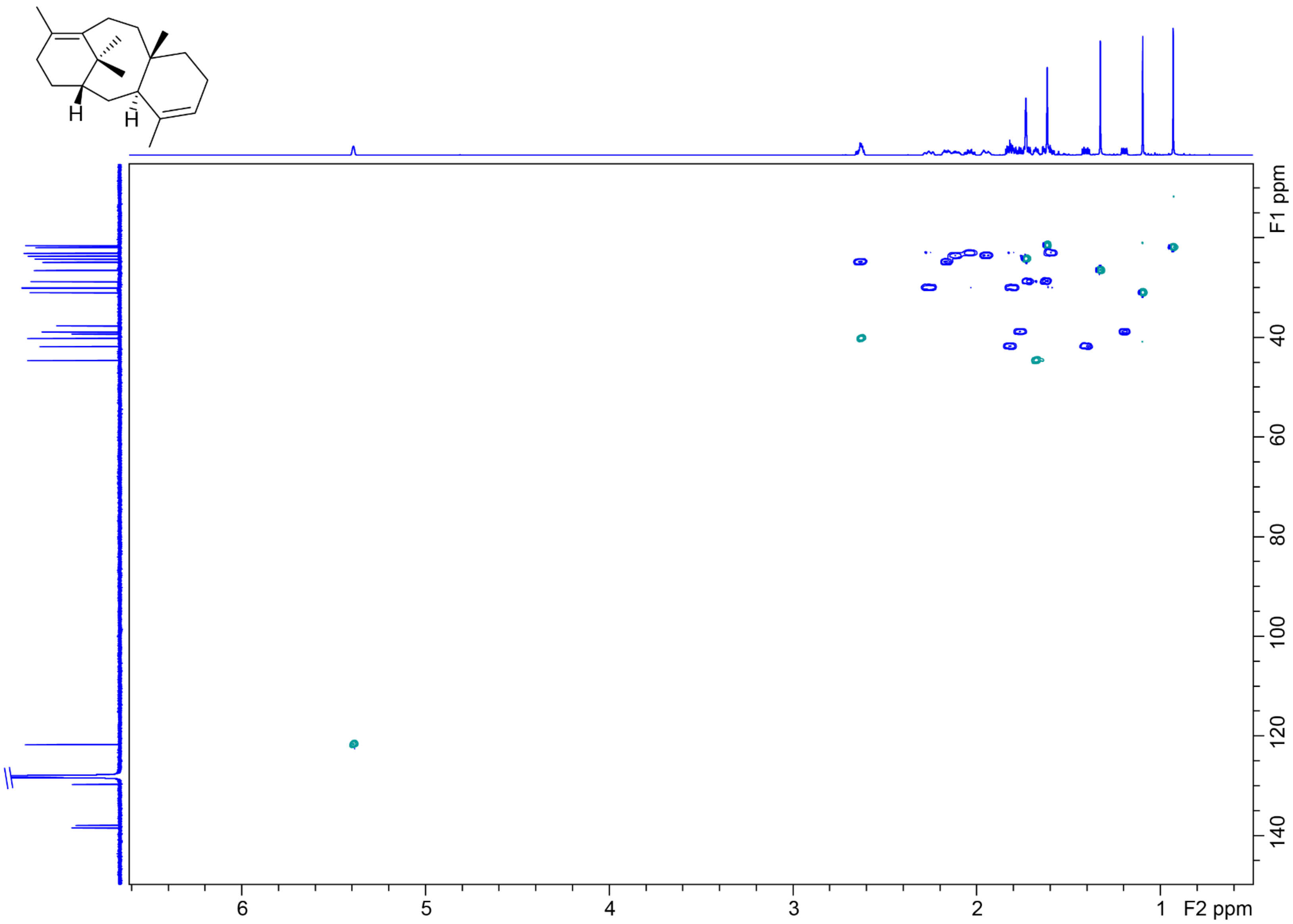

Figure S7. HSQC spectrum (C_6D_6) of **1**.

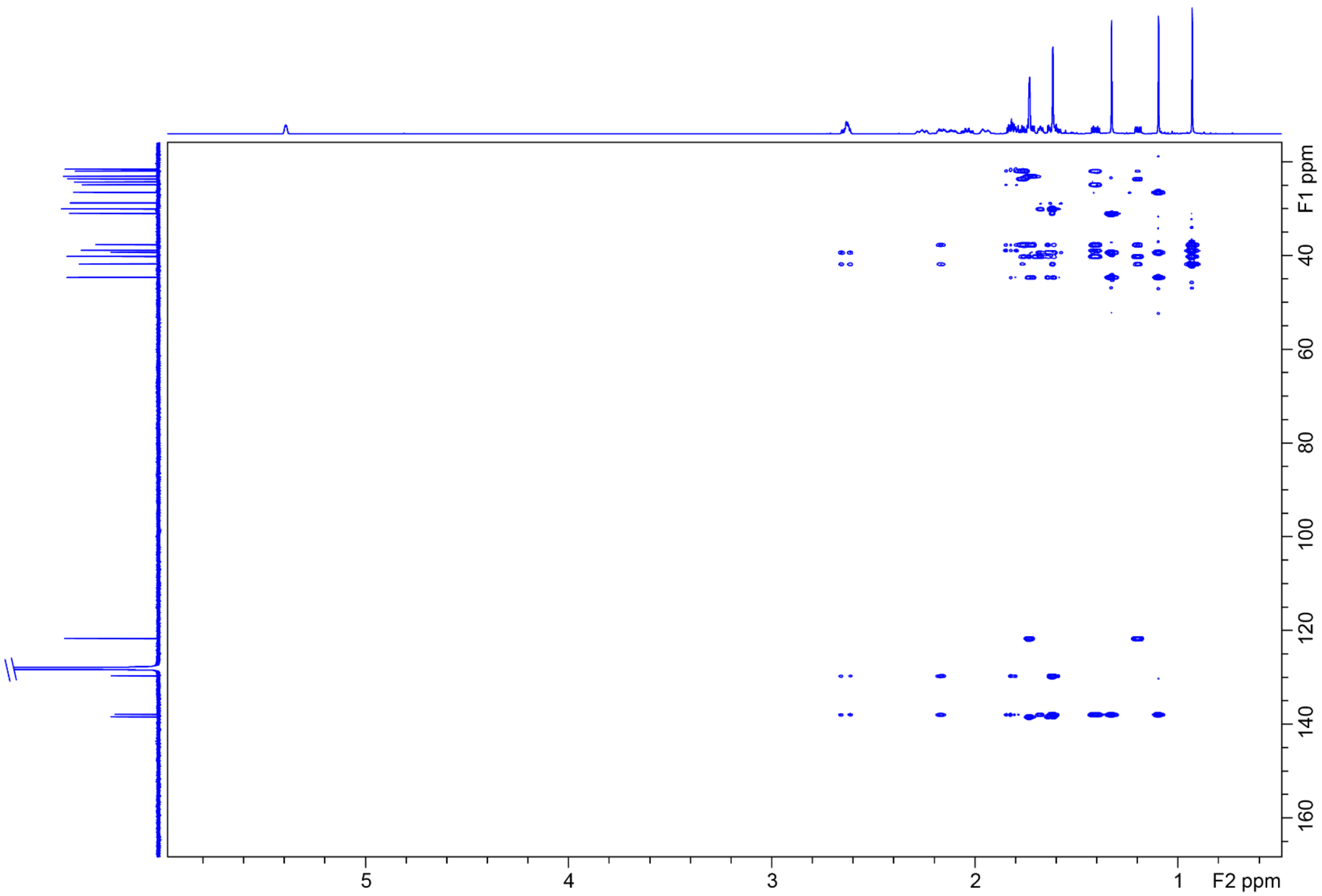

Figure S8. HMBC spectrum (C_6D_6) of **1**.

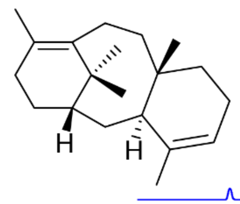

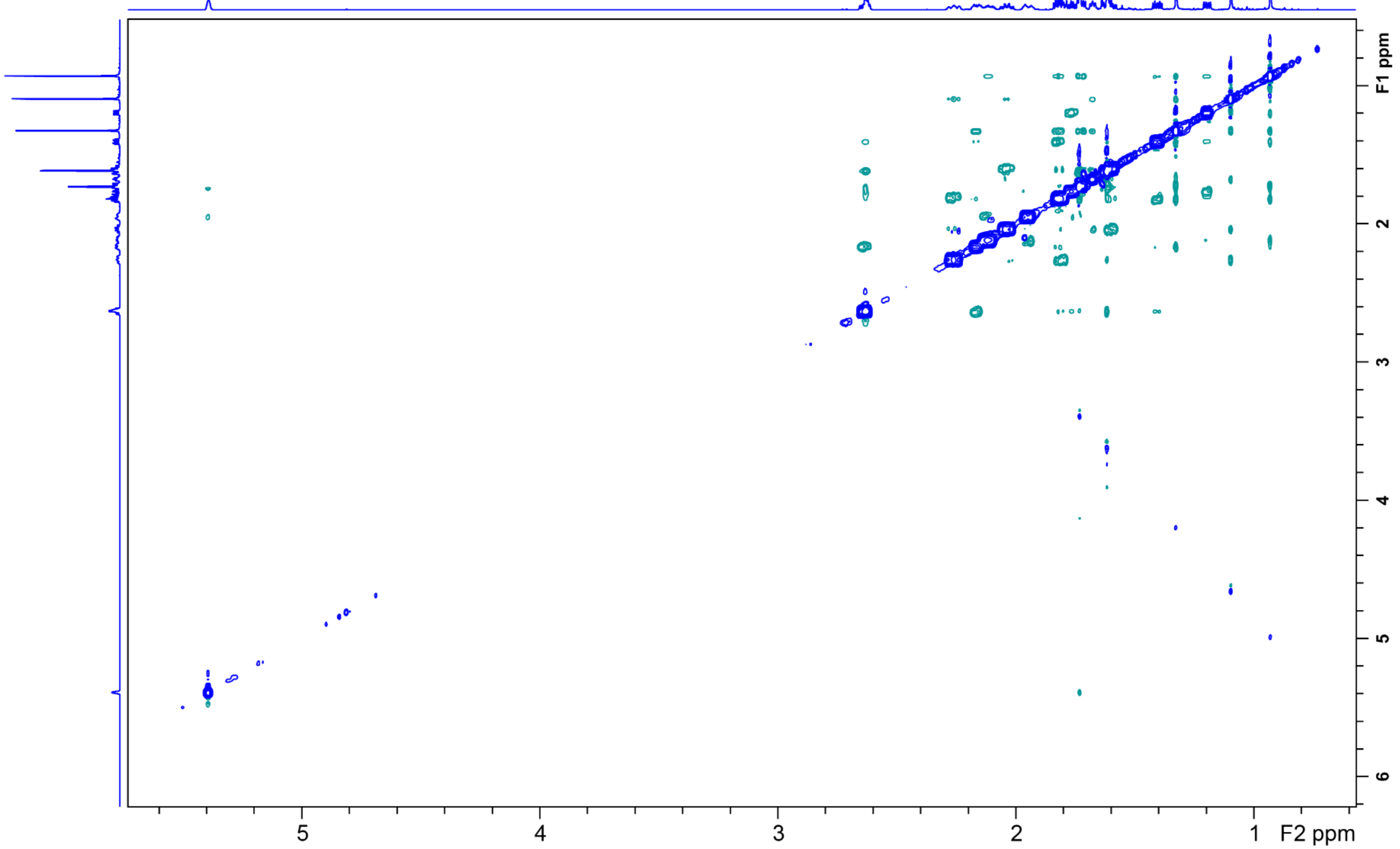

Figure S9. NOESY spectrum (C_6D_6) of **1**.

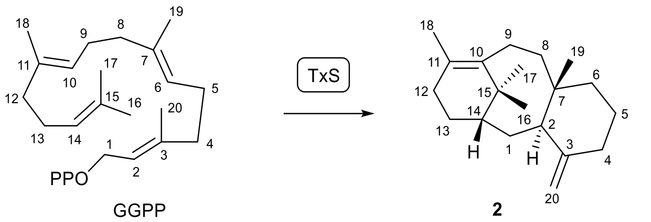

Figure S10. Carbon numbering of taxa-4(20),11-diene (**2**), indicating the origin of each carbon from GGPP by same number.

Table S4. NMR data of taxa-4(20),11-diene (**2**) in C_6D_6 recorded at 298 K.

C[a]	type	^{13}C[b]	^{1}H[b]
1	CH_2	29.32	1.69 (m, H_α)
			1.65 (m, H_β)
2	CH	43.05	2.70 (br d, *J* = 5.8)
3	C_q	153.65	–
4	CH_2	38.88	2.30 (dddd, *J* = 12.5, 3.8, 1.8, 1.8, H_β)
			1.93 (m, H_α)
5	CH_2	24.55	1.61 (m, H_α)
			1.58 (m, H_β)
6	CH_2	38.42	1.88 (ddd, *J* = 13.1, 13.1, 5.0, H_α)
			1.12 (dddd, *J* = 13.3, 3.8, 1.8, 1.8, H_β)
7	C_q	40.49	–
8	CH_2	40.68	2.00 (ddd, *J* = 15.0, 13.4, 5.2, H_β)
			1.15 (ddd, *J* = 15.1, 5.1, 2.5, H_α)
9	CH_2	25.16	2.78 (ddd, *J* = 13.6, 13.6, 5.3, H_α)
			2.07 (m, H_β)
10	C_q	138.09	–
11	C_q	129.77	–
12	CH_2	30.50	2.27 (m, H_β)
			1.75 (ddd, *J* = 18.3, 10.3, 2.8, H_α)
13	CH_2	23.14	1.97 (m, H_β)
			1.35 (ddd, *J* = 14.8, 10.3, 4.8, H_α)
14	CH	44.06	1.70 (m)
15	C_q	39.70	–
16	CH_3	31.09	1.10 (s)
17	CH_3	25.72	1.32 (s)
18	CH_3	22.14	1.71 (s)
19	CH_3	23.27	0.73 (s)
20	CH_2	105.88	4.89 (m, H_E)
			4.68 (m, H_Z)

[a] Carbon numbering as shown in Figure S10. [b] Chemical shifts δ in ppm, multiplicity: s = singlet, d = doublet, m = multiplet, coupling constants *J* are given in Hertz.

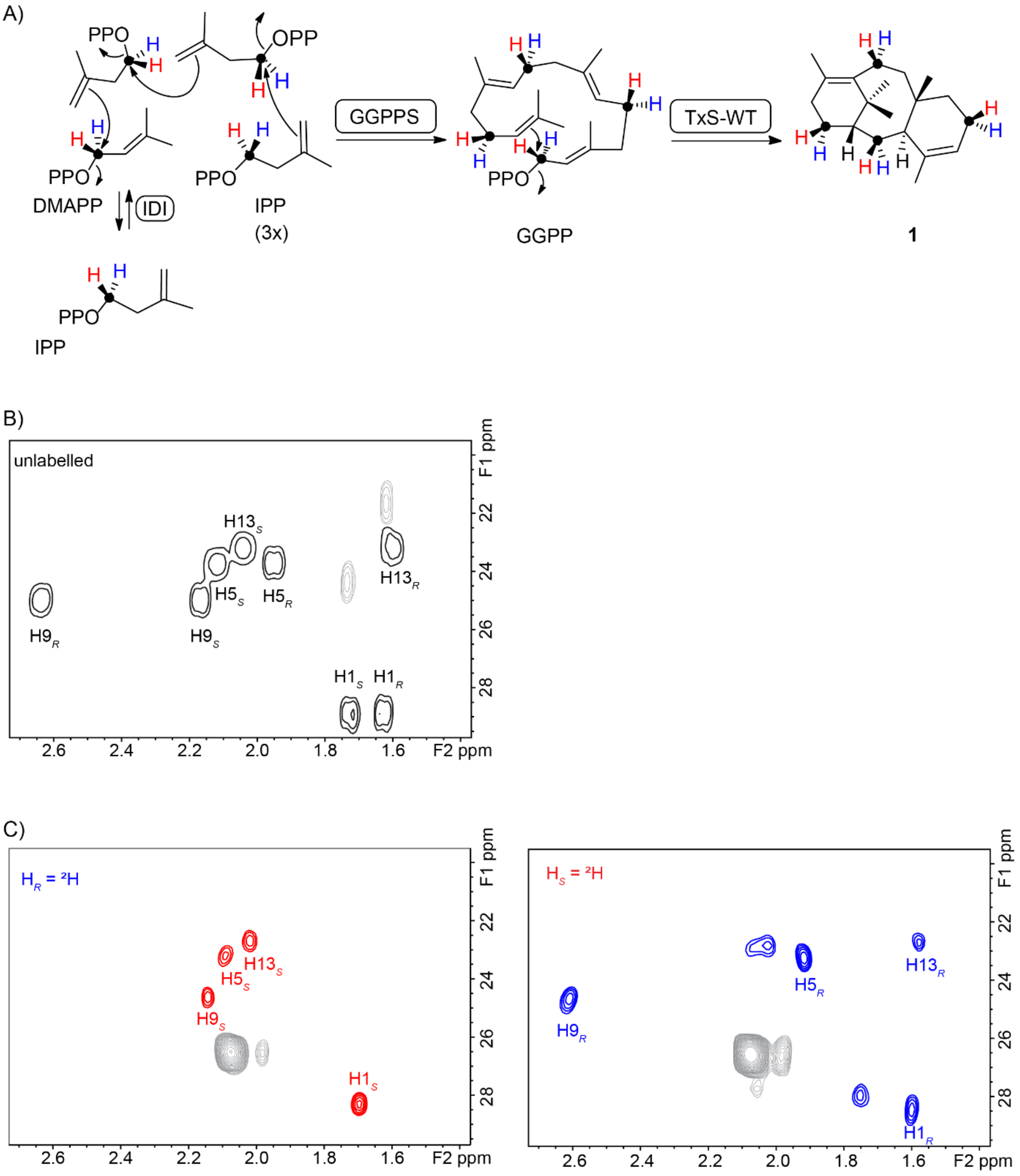

Figure S11. NMR assigment for the diastereotopic hydrogen atoms at C1, C5, C9 and C13. A) Enzymatic conversion of (*R*)- (blue hydrogen substituted with deuterium) and (*S*)-(1-^{13}C,1-^{2}H)IPP (red hydrogen substituted with deuterium) with IDI, GGPPS and TxS into labelled **1**. HSQC spectra of B) unlabelled **1** and C) labelled **1** obtained from (*R*)- (left) and (*S*)-(1-^{13}C,1-^{2}H)IPP (right). Black dots indicate ^{13}C-labelled carbons.

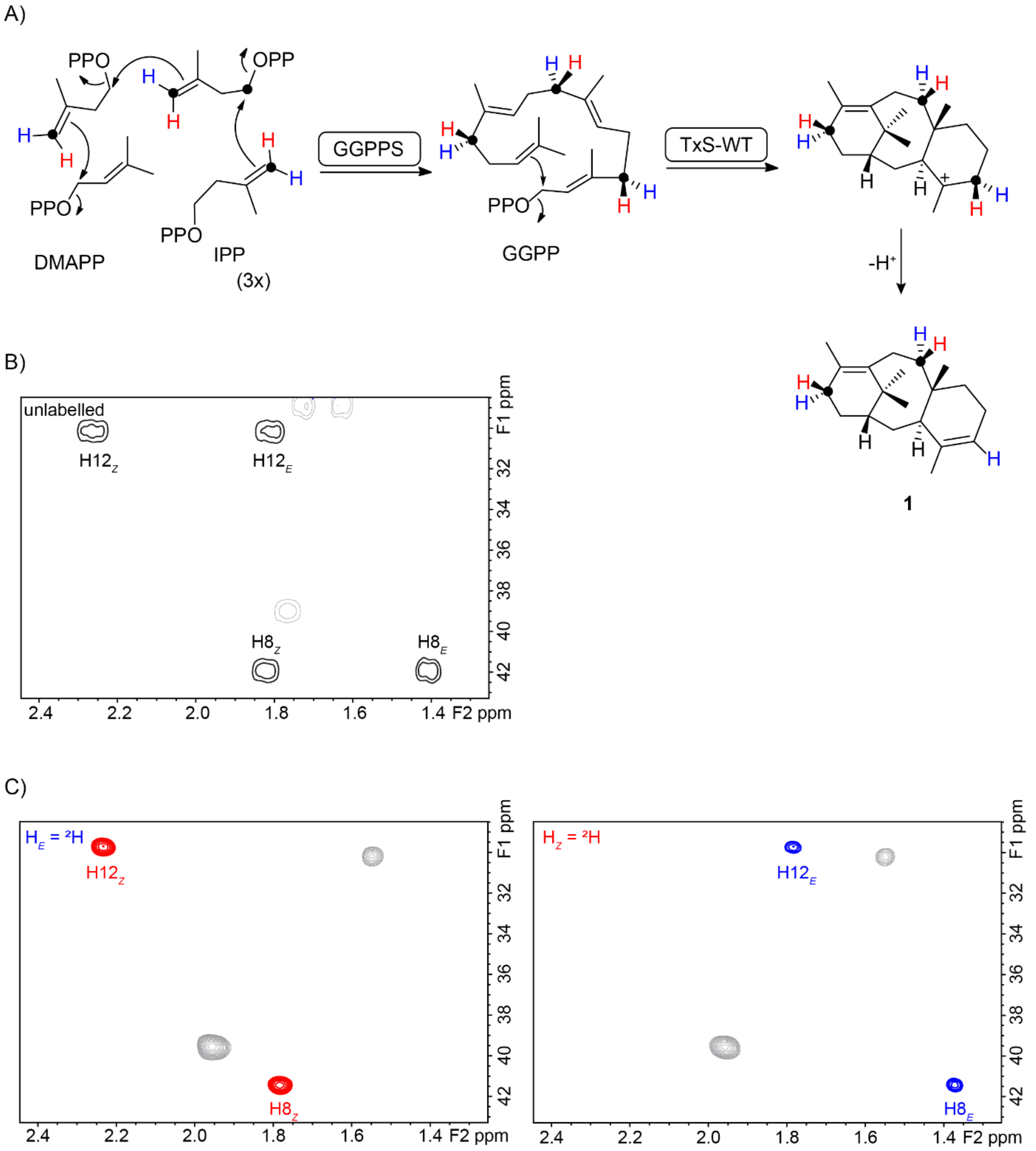

Figure S12. NMR assigment for the diastereotopic hydrogen atoms at C8 and C12 and confirmation of the reported stereochemical course for the terminal deprotonation step. A) Enzymatic conversion of DMAPP and (*E*)- (blue hydrogen substituted with deuterium) or (*Z*)-(4-^{13}C,4-^{2}H)IPP (red hydrogen substituted with deuterium) with GGPPS and TxS into labelled **1**. HSQC spectra of B) unlabelled **1** and C) labelled **1** obtained from (*E*)- (left) and (*Z*)-(4-^{13}C,4-^{2}H)IPP (right). GC/MS analysis of the products revealed the specific loss of deuterium from (*Z*)-(4-^{13}C,4-^{2}H)IPP in the terminal deprotonation step (Figure S15). Black dots indicate ^{13}C-labelled carbons.

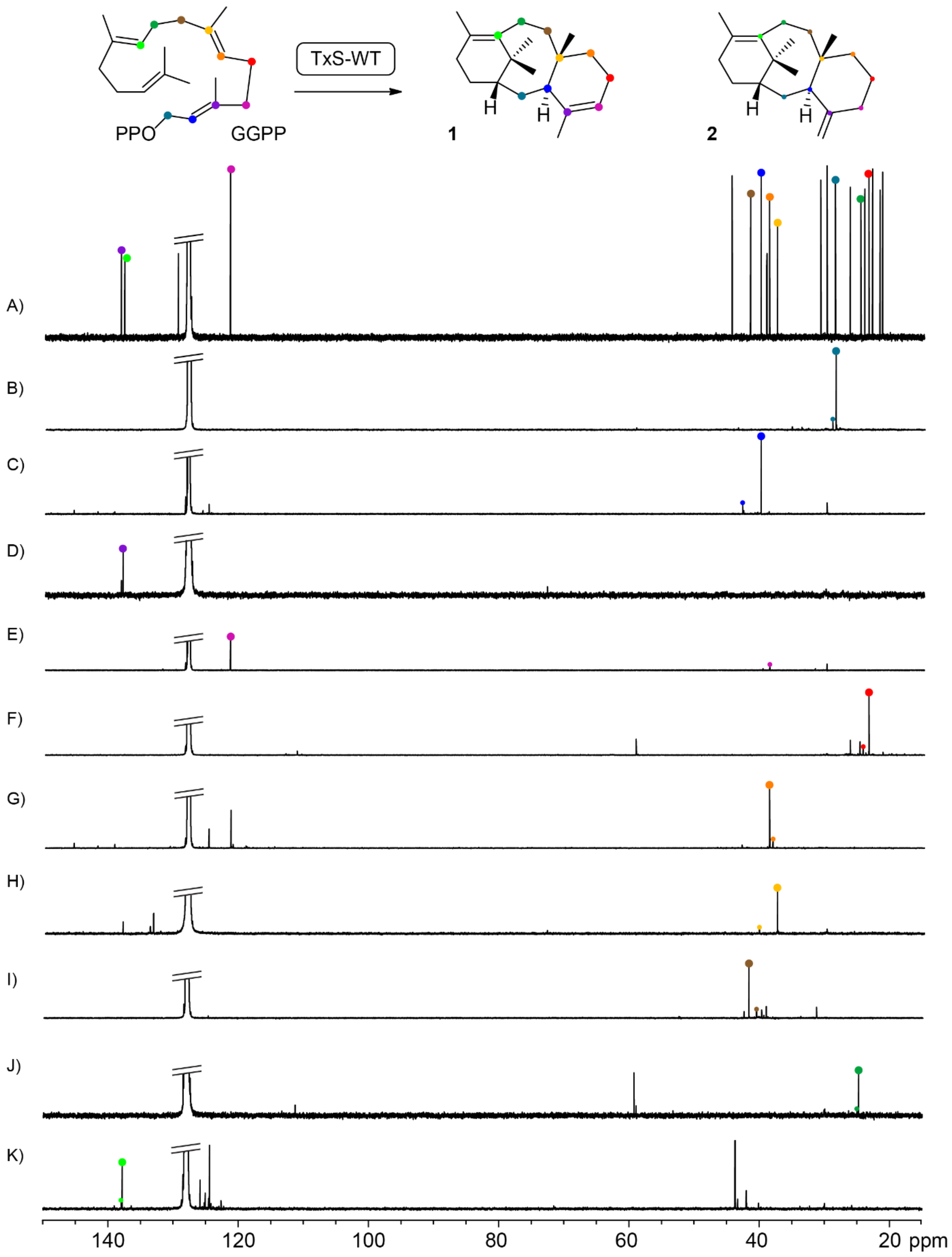

Figure S13. ^{13}C-NMR spectra of A) unlabelled **1** and B) – K) the mixture of labelled products obtained with TxS from (1-^{13}C)GGPP – (10-^{13}C)GGPP. Labelled substrates were prepared by chemical and enzymatic synthesis (cf. Table S1). The coloured dots highlight the sites of incorporation into **1** (large dots) and into **2** (small dots) and the corresponding signals in the ^{13}C-NMR spectra.

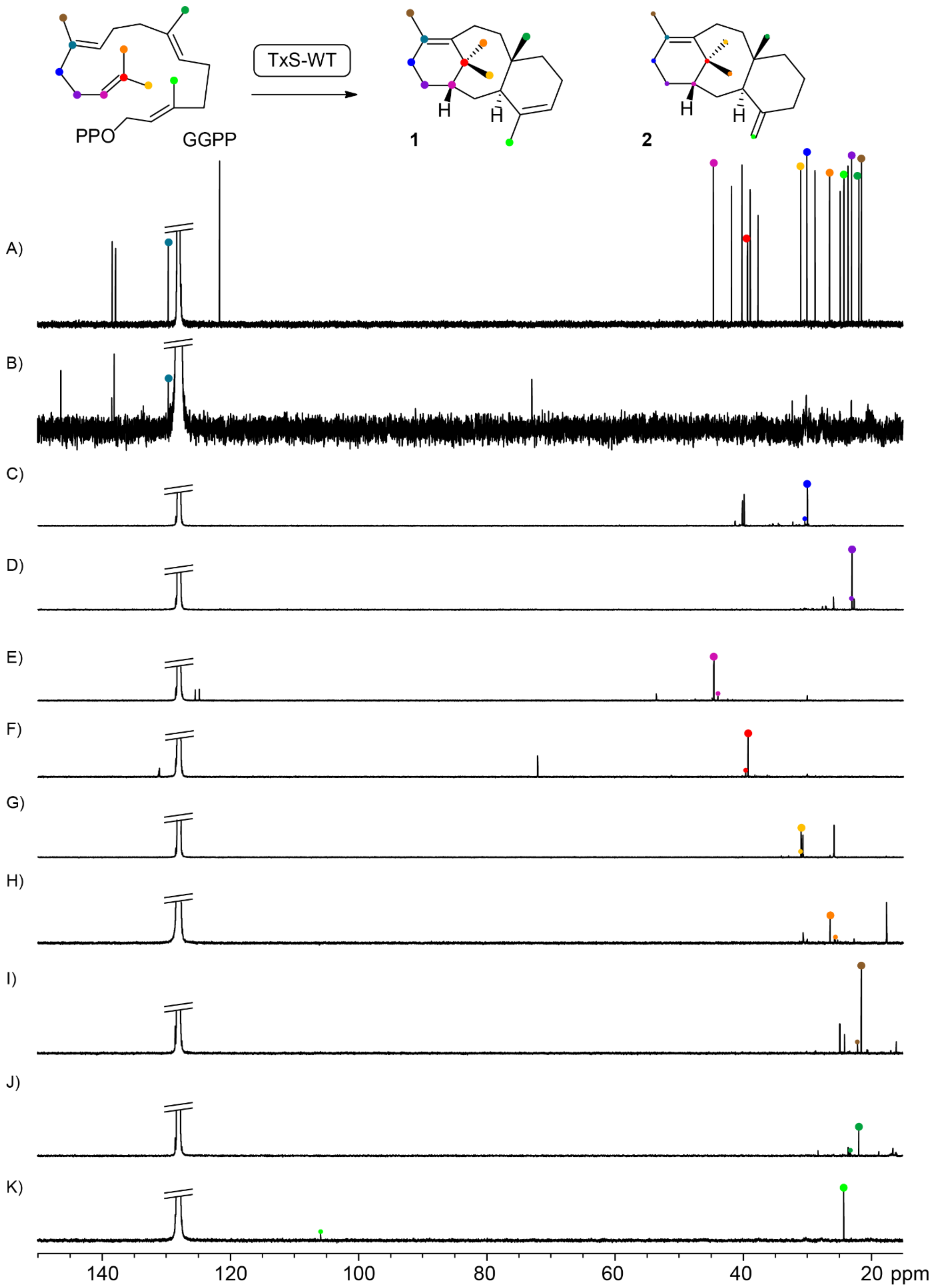

Figure S14. ^{13}C-NMR spectra of A) unlabelled **1** and B) – K) the mixture of labelled products obtained with TxS from (11-^{13}C)GGPP – (20-^{13}C)GGPP. Labelled substrates were prepared by chemical and enzymatic synthesis (cf. Table S1). The coloured dots highlight the sites of incorporation into **1** (large dots) and into **2** (small dots) and the corresponding signals in the ^{13}C-NMR spectra.

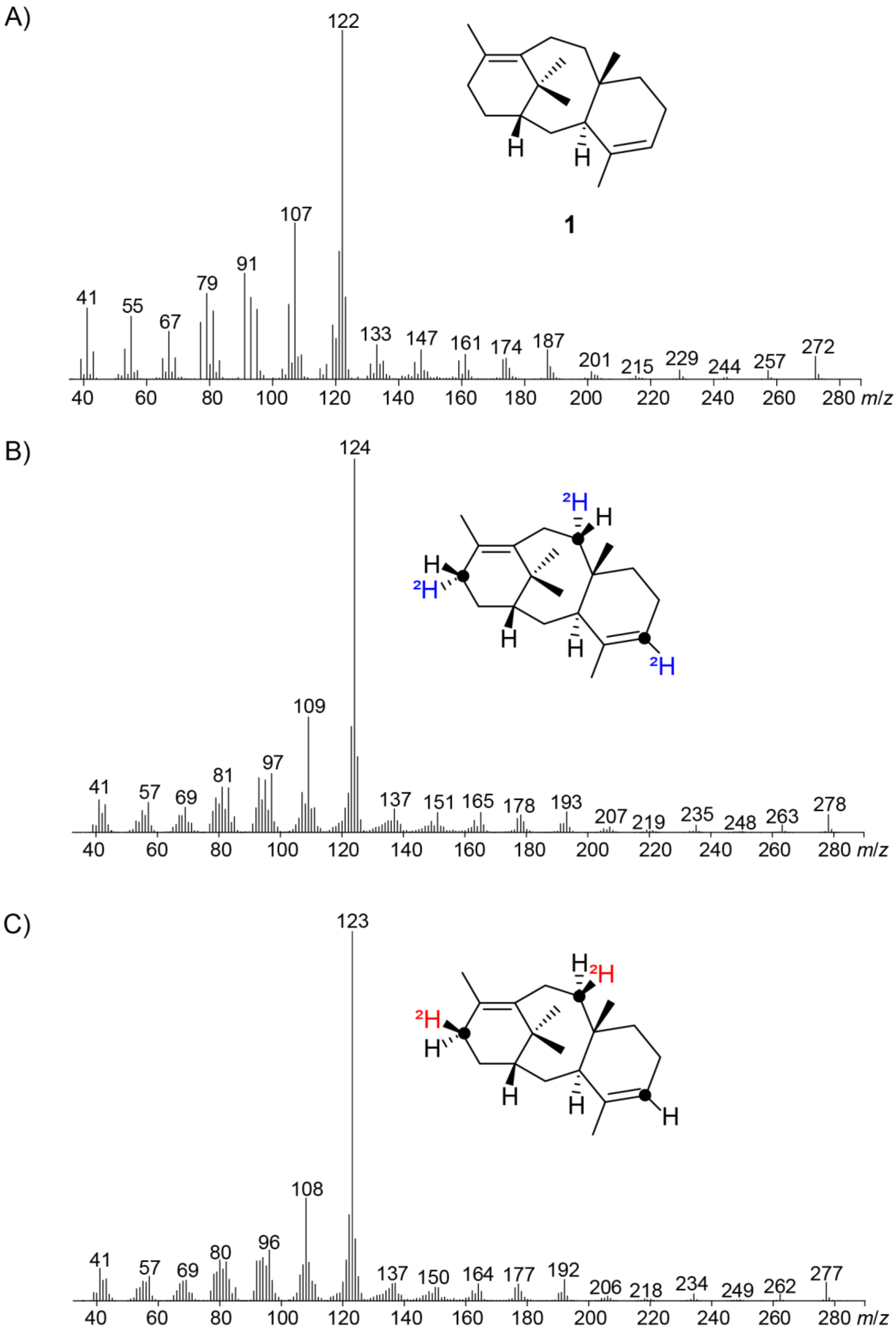

Figure S15. EI mass spectra of A) unlabelled **1**, B) and C) labelled **1** obtained from an enzymatic conversion of DMAPP and (*E*)- or (*Z*)-(4-^{13}C,4-^{2}H)IPP with GGPPS and TxS. The molecular ion in B) at *m*/*z* 278 shows retainment of three deuterium atoms, while the molecular ion in C) at *m*/*z* 277 indicates the incorporation of only two deuterium atoms and thus the loss of one deuterium in the terminal deprotonation.

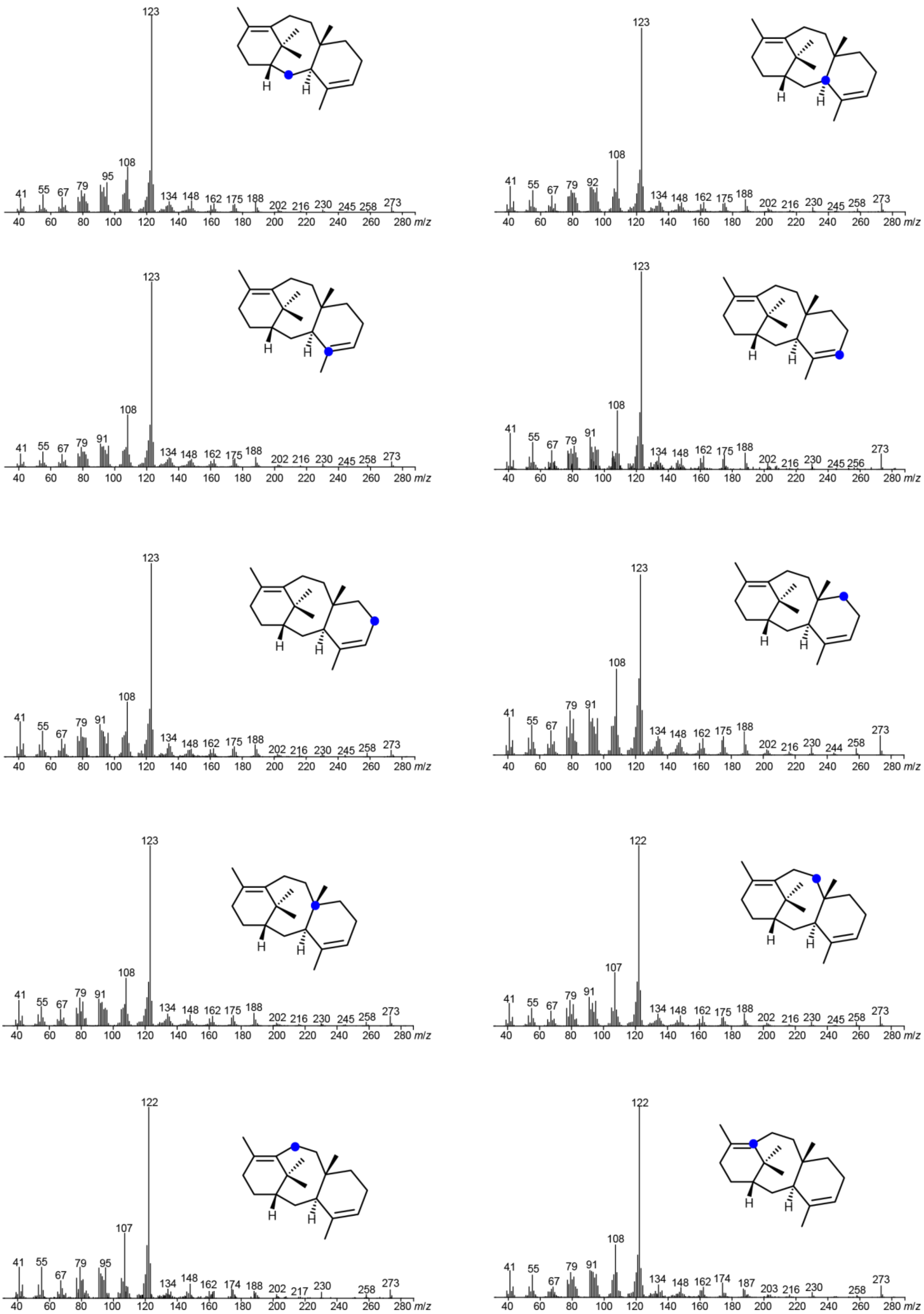

Figure S16. EI mass spectra of labelled **1** obtained with TxS from (1-^{13}C)GGPP – (10-^{13}C)GGPP. Blue dots indicate ^{13}C-labelled carbons.

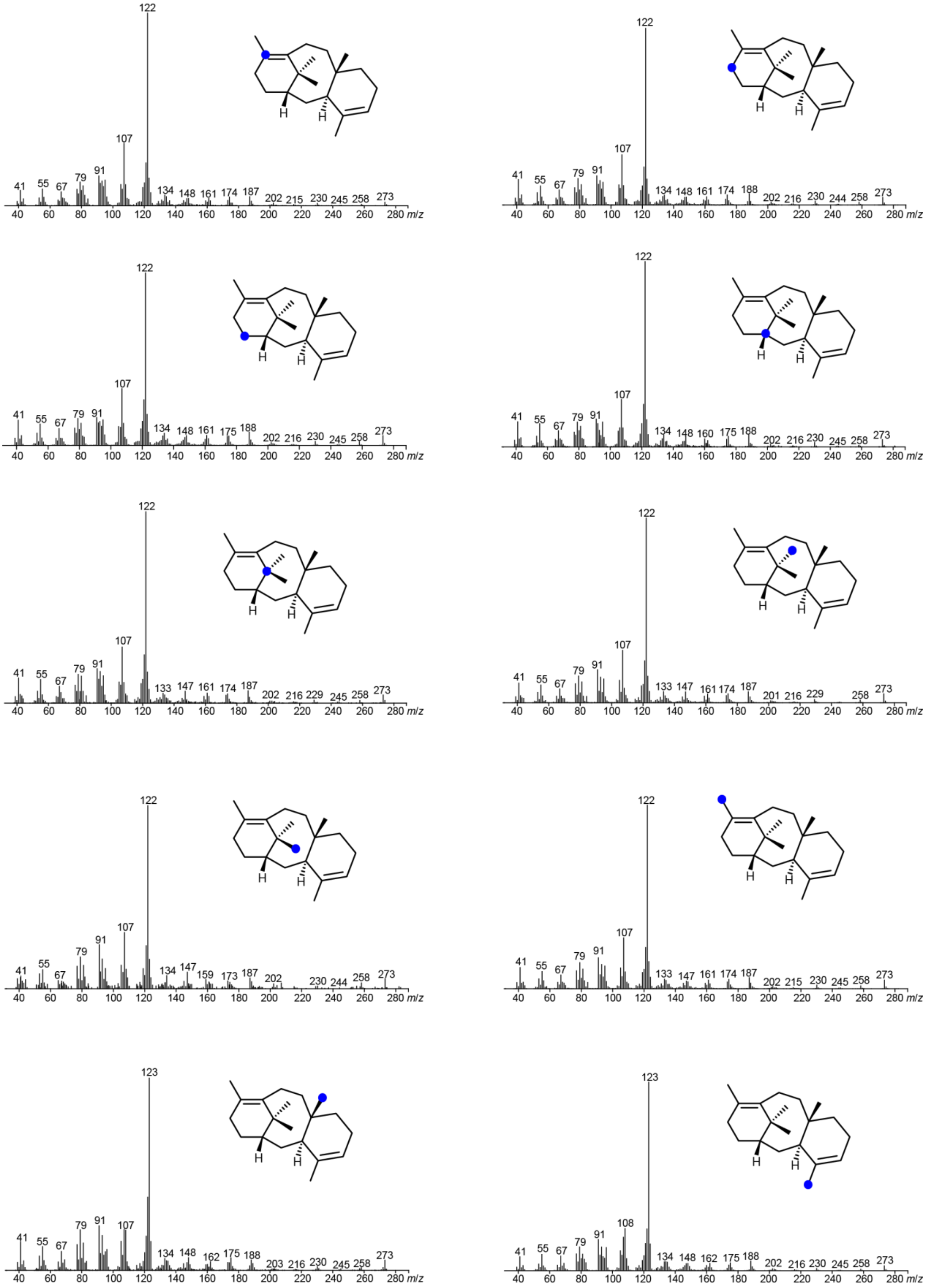

Figure S17. EI mass spectra of labelled **1** obtained with TxS from (11-^{13}C)GGPP – (20-^{13}C)GGPP. Blue dots indicate ^{13}C-labelled carbons.

Computational methods

All computed structures were geometry optimized without restrictions and were characterized as minima or as transition state structures by frequency analyses using the B97D3/6-31g(d,p) method with the density fitting approximation for s- and p-functions, including Grimme's empirical D3-dispersion correction[20] in Gaussian16.[21] Frequency computations also provided Gibbs corrections, which include Grimme's quasi-RRHO approach with a frequency cut-off value of 100.0 wave numbers using GoodVibes.[22,23] For single point energies, the mPW1PW91 functional was applied with the 6-311+G(d,p) basis set without density fitting and the ultra-fine integration grid, as this method was shown to be very reliable for examining carbocation cyclization and rearrangement reactions.[24-28] Conformational analyses were performed with xTB-GFN2 in the CREST 2.12 program (github.com/crest-lab), developed by the Grimme group.[29-33]

Kinetic isotope effects (KIE) were computed with the program kinisot.py of the Paton group (github.com/patonlab/Kinisot), based on B97D3/6-31g(d,p) frequencies at 298.15 K. Kinisot.py diagonalizes the mass-weighted Hessian matricies to obtain harmonic vibrational frequencies and Bigeleisen-Mayer reduced isotopic partition function ratios.[34]

Table S5. Results of DFT calculations for the EI-MS fragmentation of **1** (Scheme 3 of main text).

Structure	Gibbs energy (298.15K) in Hartree	energy relative to $A^{+\bullet}$ in kcal/mol	reaction barrier in kcal/mol	Gibbs free energy in kcal/mol
**$A^{+\bullet}$**	–781.109516	0.00		
**$A^{+\bullet}$-$B^{+\bullet}$-TS**	–781.052547	35.75	35.75	
**$B^{+\bullet}$**	–781.052066	36.05		36.05
**$B^{+\bullet}$**	–781.051526	36.39		
**$B^{+\bullet}$-$C^{+\bullet}$-TS**	–781.031695	48.83	12.44	
**$C^{+\bullet}$**	–781.063400	28.94		–7.45
**$C^{+\bullet}$**	–781.072985	22.92		
**$C^{+\bullet}$-$D^{+\bullet}$-TS**	–781.022278	54.74	31.82	
**$D^{+\bullet}$**	–781.045626	40.09		17.17
**$D^{+\bullet}$**	–781.045624	40.09		
**$D^{+\bullet}$-$E1^{+\bullet}$-TS**	–781.035988	46.14	6.05	
**$E1^{+\bullet}$**	–781.042329	42.16		2.07
**$E1^{+\bullet}$**	–781.045729	40.03		
**$E1^{+\bullet}$-$E2^{+\bullet}$-TS**	–781.037931	44.92	4.89	
**$E2^{+\bullet}$**	–781.049131	37.89		–2.13
**$E2^{+\bullet}$**	–781.047669	38.81		
**$E2^{+\bullet}$-$F^{+\bullet}$-TS**	–781.037719	45.05	6.24	
**$F^{+\bullet}$**	–781.052350	35.87		–2.94
**$F^{+\bullet}$**	–781.052453	35.81		
**$F^{+\bullet}$-$G^{+\bullet}$-TS**	–781.042215	42.23	6.42	
**$G^{+\bullet}$**	–781.042248	42.21		6.40

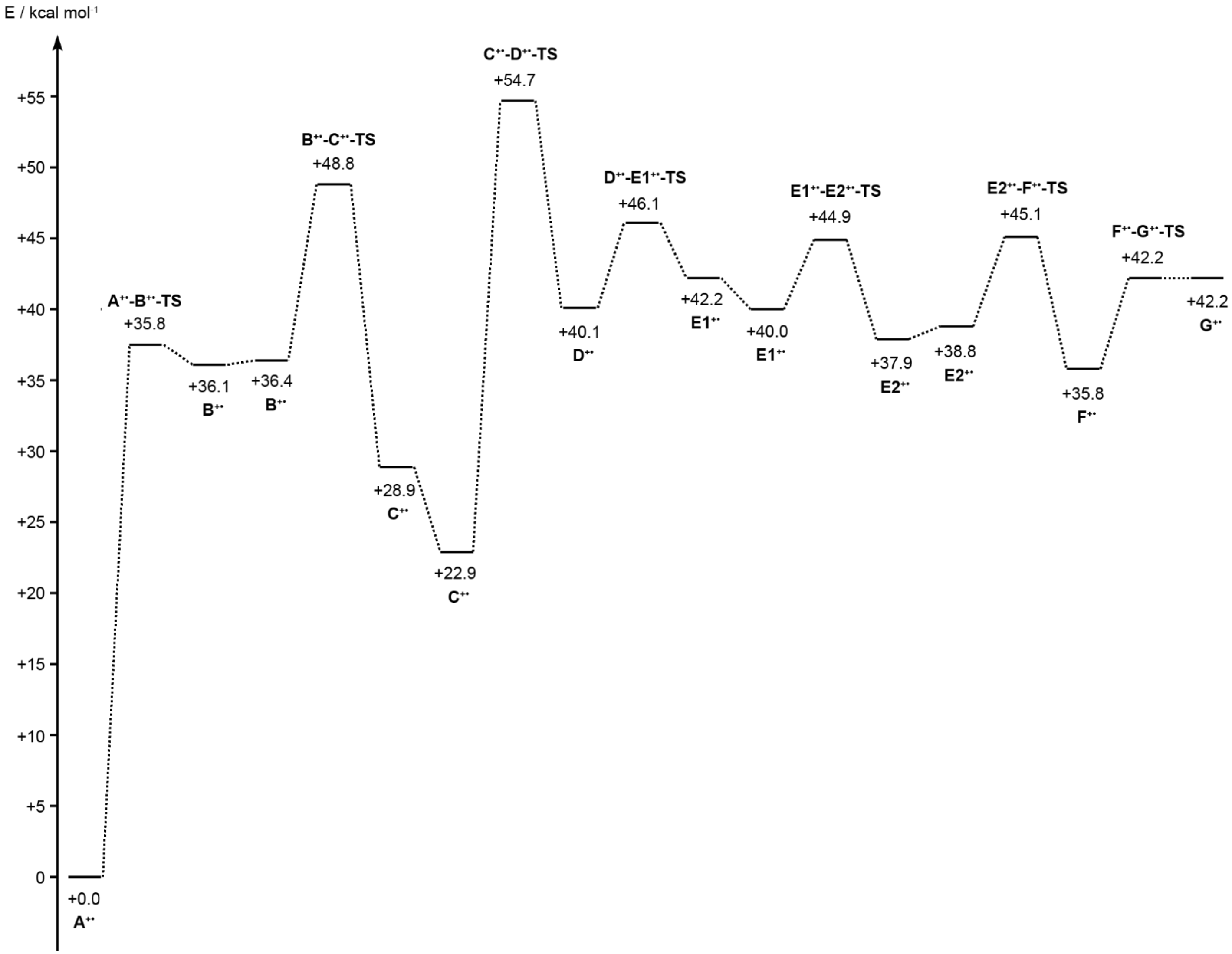

Figure S18. Computed energy profile for the transformations of **A+•** to **F+•** (Scheme 3 of main text, mPW1PW91/6-311+G(d,p)//B97D3/6-31g(d,p), 298 K).

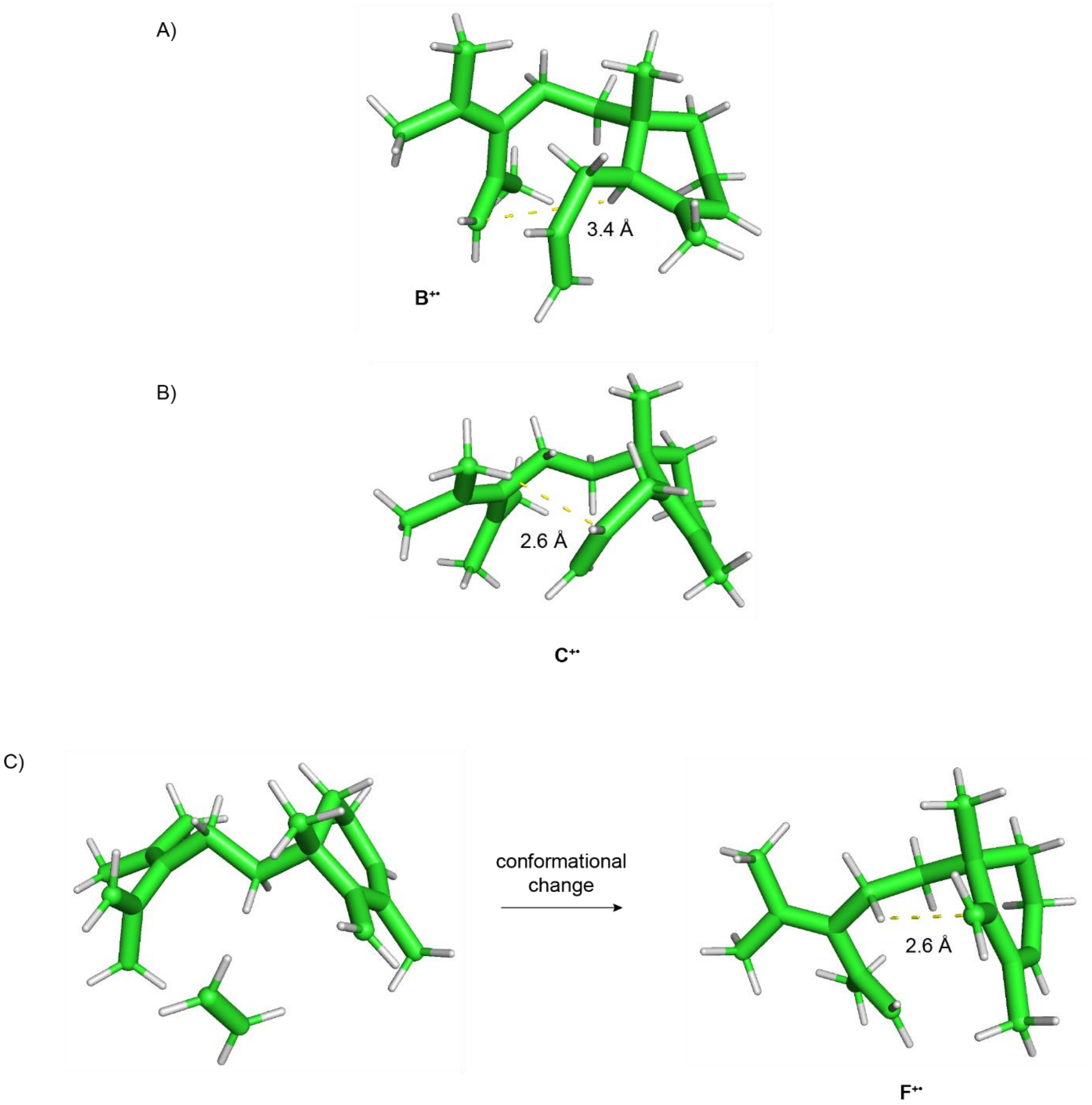

Figure S19. Computed structures of intermediates and distances for hydrogen migrations in the EI-MS fragmentation mechanism of **1**.

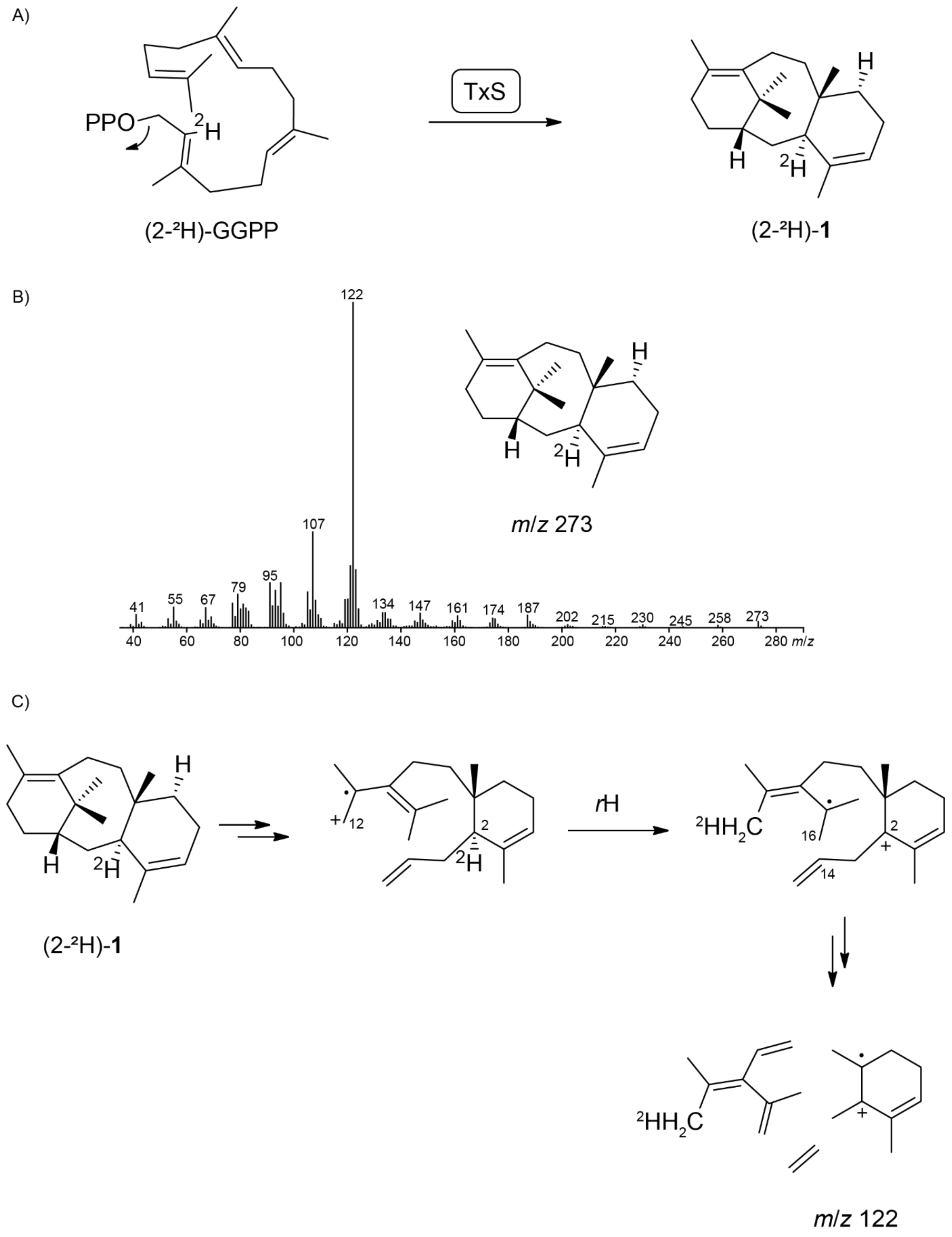

Figure S20. The long distance hydrogen migration from $B^{+•}$ to $C^{+•}$ in the formation of the base peak ion (*m*/*z* 122) of **1**. A) Formation of (2-^{2}H)-**1** through incubation of (2-^{2}H)GGPP with TxS (cf. Scheme 1 of main text for mechanistic details). B) EI mass spectrum of (2-^{2}H)-**1** showing a base peak ion at *m*/*z* 122 as observed for unlabelled **1** (Figure S15A), indicating the loss of deuterium from the corresponding fragment ion. C) Formation of the fragment ion *m*/*z* 122 from (2-^{2}H)-**1** (cf. Scheme 3 of main text for mechanistic details).

Table S6. Summary of previous site-directed mutagenesis experiments with TxS.

position	enzyme variants created	results	reference
Y89	Y89F, Y89F	inactive	[35]
	Y89E	reduced activity, **1**	[35]
R580	R580H, R580A, R580E	inactive	[35]
V584	V584M, V584K, V584L	retained activity, taxadienes and verticillatrienes	[35]
	V584N, V584S, V584P, V584R	inactive	[35]
S587	S587D, S587Y, S587K, S587L, S587G	inactive	[35]
	S587A	reduced activity, **1** and verticillatrienes	[35]
	saturation mutagenesis	all variants have a lower activity or are inactive	[16]
F602	F602W, F602A	reduced activity, **1**	[35]
	F602I	reduced activity, taxadienes	[35]
Q609	saturation mutagenesis	all variants have a lower activity or are inactive, Q609G selectively produces verticilla-3,7,12-triene	[16]
V610	V610H, V610S, V610F, V610A	inactive	[35]
Y684	saturation mutagenesis	several variants retain activity, other variants have a lower activity, some variants produce verticilla-3,7,12-triene	[16]
Y688	saturation mutagenesis	all variants have a lower activity or are inactive, some variants produce verticilla-3,7,12-triene, Y688L shows a good production of taxa-4(20),11-diene	[16]
S713	S713T	retained activity, taxadienes, traces of verticillatrienes	[35]
	S713A, S713L	inacvtive	[35]
	saturation mutagenesis	no detailed results reported	[16]
V714	V714T, V714A, V714I	reduced activity, taxadienes	[35]
	V714G, V714P	inactive	[35]
	saturation mutagenesis	no detailed results reported	[16]
G715	G715A, G715S	inactive	[35]
	saturation mutagenesis	no detailed results reported	[16]
C719	C719A	reduced activity, products: **1**, **5**	[16]
W753	W753H	reduced activity, cembrene A	[35]
	W753L, W753V, W753E, W753C, W753A	inactive	[35]
	saturation mutagenesis	no detailed results reported	[16]

R754	R754H, R754A, R754E	inactive	[35]
R768	R768H, R768A, R768E	inactive	[35]
C830	C830A, C830S	retained activity, taxadienes and traces of verticillatrienes	[35]
	saturation mutagenesis	all variants have a lower activity or are inactive	[16]
V831	saturation mutagenesis	no detailed results reported	[16]
F834	F834A, F834G	reduced activity, taxadienes and verticillatrienes	[35]
	F834H	inactive	[35]
Y835	Y835F	reduced activity, **1**	[35]
	Y835W, Y835A	inactive	
	saturation mutagenesis	no detailed results reported	[16]
Y841	Y841A, Y841T	inactive	[35]
	Y841F	reduced activity, cembrene A, verticilla-3,7,12-triene	[35]

Site-directed mutagenesis

Plasmids for the expression of the desired enzyme variants of TxS were obtained by PCR with Q5-High-Fidelity DNA polymerase (New England Biolabs, USA), freshly isolated plasmid DNA (pYE-TxS) as a template and corresponding primers (Table S7). PCR conditions were: initial denaturation at 98 °C for 1 min, 30 cycles of denaturation at 98 °C for 10 s, annealing at 63 °C for 30 s and elongation at 72 °C for 6 min, and final elongation at 72 °C for 7 min. DpnI (1 μL, New England Biolabs, USA) and the rCutSmart buffer (5 μL, New England Biolabs, USA) were added to the PCR products and the mixtures were incubated at 37 °C for 1 h to digest the original template DNA. After purification with the PCR Clean-Up kit (Macherey-Nagel, Germany), the corresponding PCR products were used for electroporation of *Escherichia coli* BL21 (DE3) electrocompentent cells. The transformed *E. coli* cells were grown overnight at 37 °C on LB agar plates containing kanamycin sulfate (50 mg L^{-1}). Single colonies were selected to inoculate LB medium (10 mL) with kanamycin sulfate, followed by cultivation at 37 °C for 8 h. The plasmid DNA was extracted with the NucleoSpin plasmid extraction kit (Macherey-Nagel, Germany) and checked by sequencing.

Table S7. Primers used for site-directed mutagenesis.

Primer	Nucleotide sequence (5'→3')[a]
R580K-F	CGCGCCAT**AAA**GTGGCGGAAGTGTAC
R580K-R	TTCCGCCAC**TTT**ATGGCGCGTAAAGTTAAT
R580M-F	CGCGCCAT**ATG**GTGGCGGAAGTGTAC
R580M-R	TTCCGCCAC**CAT**ATGGCGCGTAAAGTTAAT
E583A-F	CGTGTG**GCG**GCGGTGTACTTTAGCAGTGC
E583A-R	AAAGTACAC**CGC**CGCCACACGATGGCGCG
E583D-F	CGTGTGGCG**GAT**GTGTACTTTAGCAGTGC
E583D-R	AAAGTACAC**ATC**CGCCACACGATGGCGCG
E583M-F	CGTGTGGCG**ATG**GTGTACTTTAGCAGTGC
E583M-R	AAAGTACAC**CAT**CGCCACACGATGGCGCG
V584A-F	GTGGCGGAA**GCG**TACTTTAGCAGTGCTAC
V584A-R	GCTAAAGTA**CGC**TTCCGCCACACGATGGCG
F602Y-F	CGTATTGCC**TAT**ACTAAGATTGGCTGTCTG
F602Y-R	ATCTTAGT**ATA**GGCAATACGCGTCGCTG
F602H-F	CGTATTGCC**CAT**ACTAAGATTGGCTGTCTG
F602H-R	ATCTTAGT**ATG**GGCAATACGCGTCGCTG
G606A-F	CTAAGATT**GCG**TGTCTGCAAGTGCTGTTTG
G606A-R	TGCAGACA**CGC**AATCTTAGTGAAGGCAAT
V610T-F	GTCTGCAA**ACC**CTGTTTGATGATATGGC
V610T-R	CAAACAG**GGT**TTGCAGACAGCCAATCTT
F834Y-F	GTACAGATC**TAC**TATAAATTTATCGATGG
F834Y-R	AAATTTATA**GTA**GATCTGTACGCACAGACG
I848L-F	ACGAAGAG**CTG**AAGGACTATATCCGGAAAG
I848L-R	TAGTCCTT**CAG**CTCTTCGTTCGCAATGCC
I848V-F	ACGAAGAG**GTG**AAGGACTATATCCGGAAAG
I848V-R	TAGTCCTT**CAC**CTCTTCGTTCGCAATGCC

[a] Modified codon triplets are highlighted in bold.

Table S8. Results obtained with TxS enzyme variants in this study.

variant	**total**[a]	**1**	**2**	**3**	**4**	**5**	**6**	**7**	**8**
wildtype	100±8.6	84.2±7.4	9.7±0.7	0.0±0.0	0.0±0.0	6.0±0.5	0.0±0.0	0.0±0.0	0.0±0.0
R580M	0.0±0.0	0.0±0.0	0.0±0.0	0.0±0.0	0.0±0.0	0.0±0.0	0.0±0.0	0.0±0.0	0.0±0.0
V584A	17.4±1.3	8.6±0.7	0.0±0.0	0.0±0.0	0.0±0.0	0.0±0.0	8.8±0.7	0.0±0.0	0.0±0.0
F602Y	6.6±0.7	6.6±0.7	0.0±0.0	0.0±0.0	0.0±0.0	0.0±0.0	0.0±0.0	0.0±0.0	0.0±0.0
G606A	28.1±0.2	7.0±0.9	0.0±0.0	0.0±0.0	19.2±1.0	0.0±0.0	0.0±0.0	0.0±0.0	2.0±0.2
V610T	55±3.5	0.0±0.0	0.0±0.0	0.0±0.0	19.7±1.3	0.0±0.0	27.2±1.7	8.2±0.7	0.0±0.0
F834Y	15.8±2.1	13.9±1.9	1.9±0.2	0.0±0.0	0.0±0.0	0.8±0.1	0.0±0.0	0.0±0.0	0.0±0.0
I848V	49.0±7.5	38.6±0.8	3.7±0.1	0.0±0.0	0.0±0.0	2.5±0.1	0.0±0.0	0.0±0.0	0.0±0.0

[a] Relative production with the total production by the wildtype set to 100%. The data represent mean ± standard deviation from triplicates.

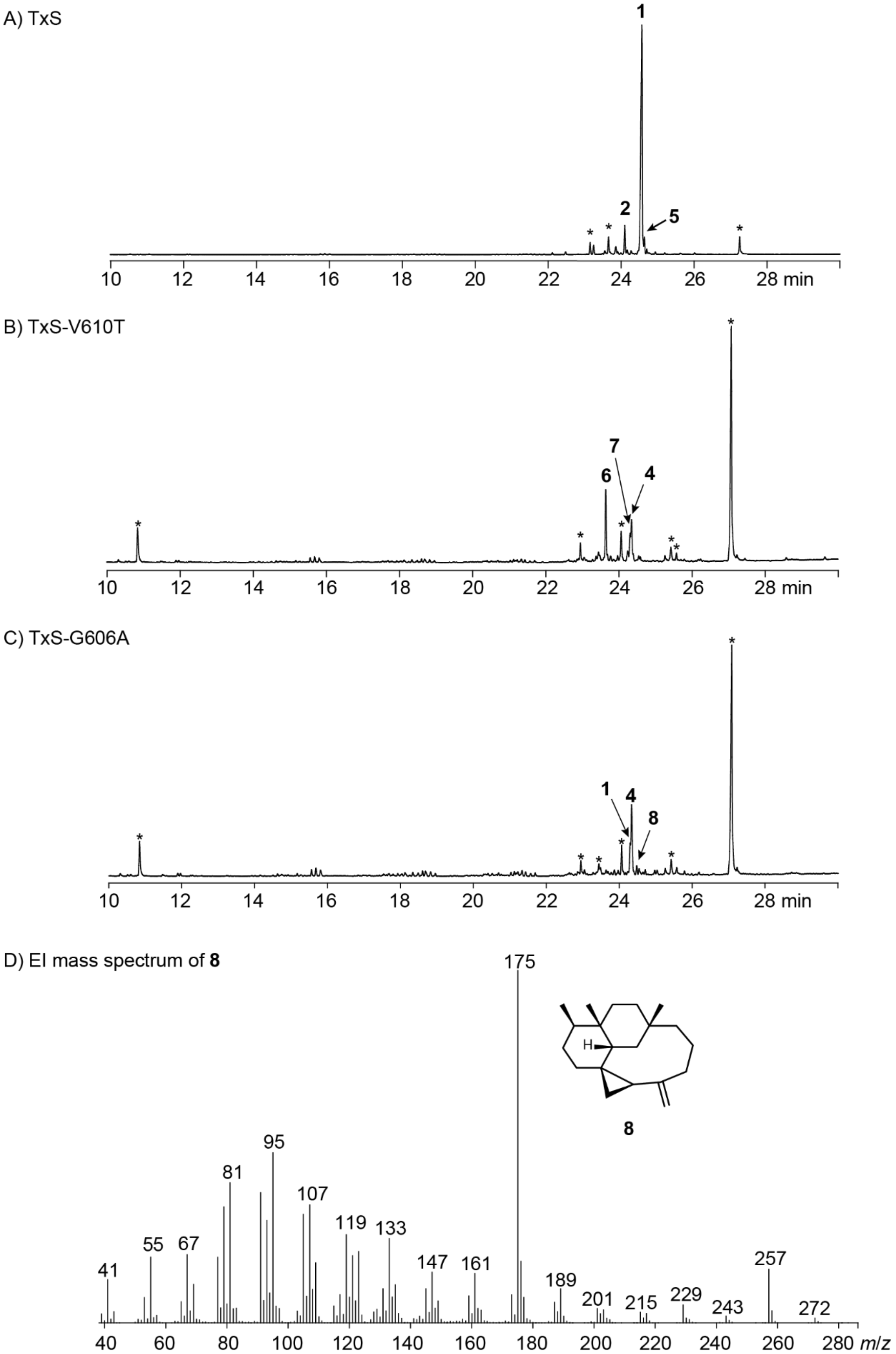

Figure S21. GC/MS analysis of the products obtained from incubations of GGPP with TxS and its enzyme variants. Total ion chromatograms of the products from A) wildtype TxS, B) the TxS-G606A variant, and C) the TxS-G606A variant. Asterisks indicate spontaneous lysis and hydrolysis products of GGPP and contaminants such as plasticisers. D) EI mass spectrum of cyclophomactene (**8**).

A)

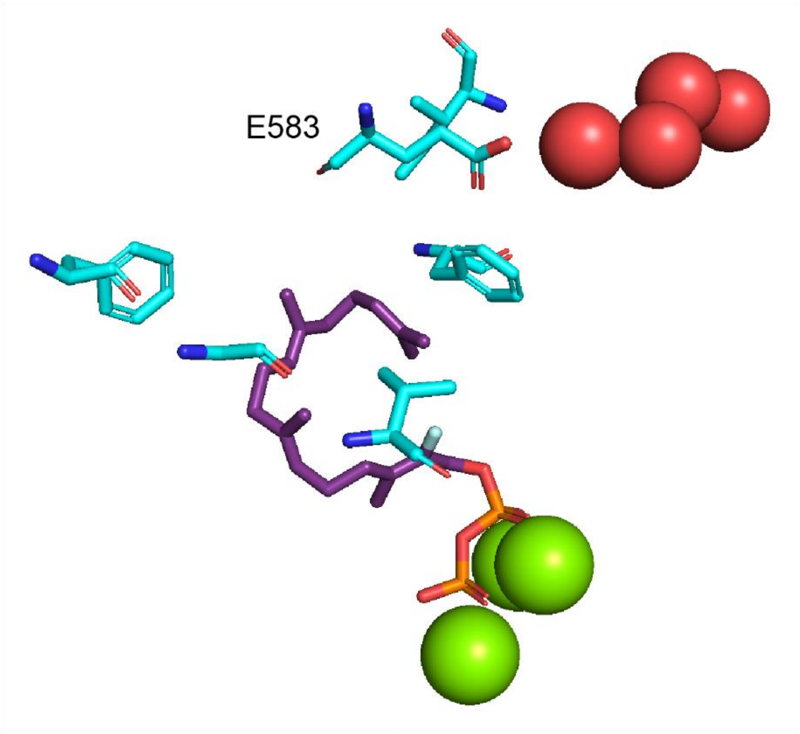

B)

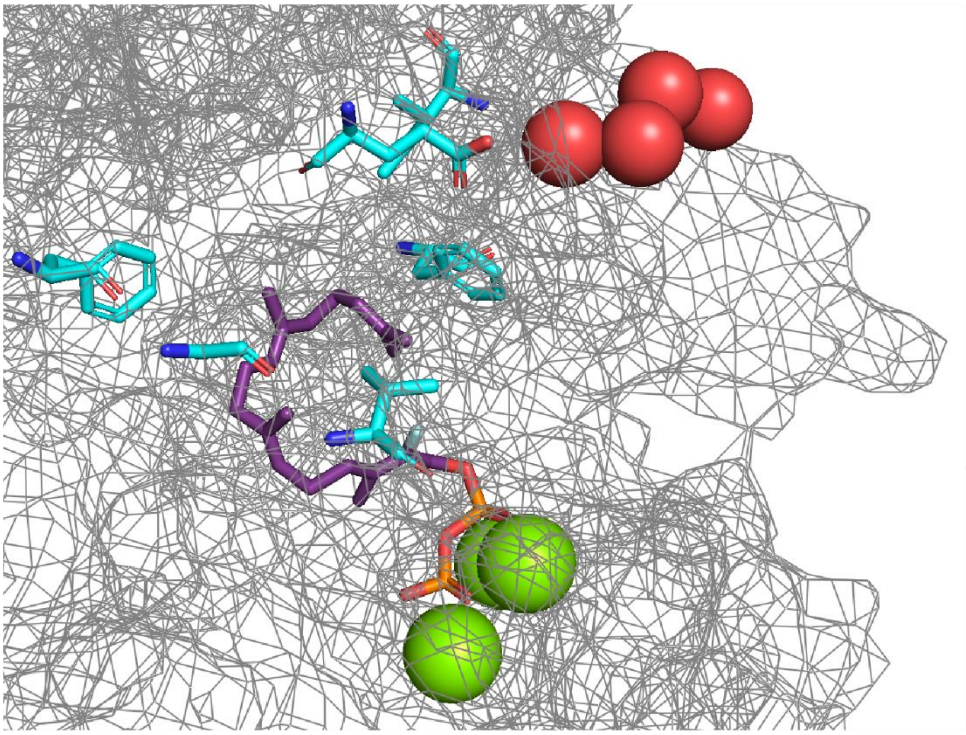

Figure S22. Structural interpretation of the results obtained with the E583D, E583M and E583A enzyme variants. A) The active site residue E583 shows hydrogen bonding to a cluster of four water molecules. B) These water molecules are located at the enzyme surface of TxS (grey meshes), suggesting that E583 serves as a gatekeeper that blocks entering of water to the active site. The figure is based on the crystal structure of TxS (PDB 3P5R).[36]

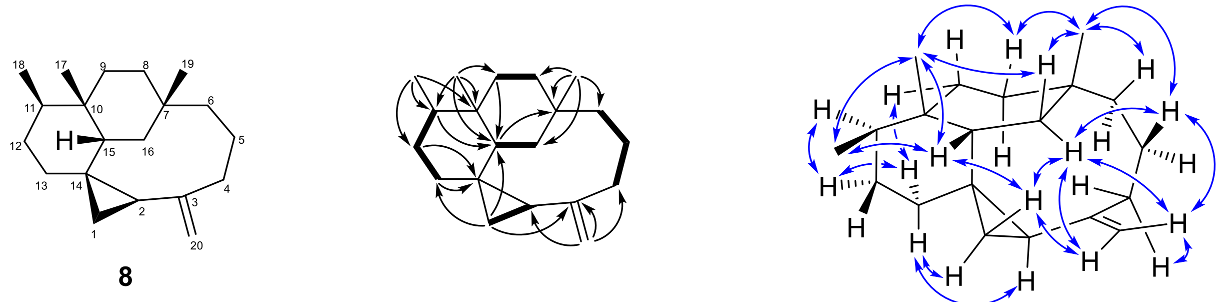

8

Figure S23. Structure elucidation of cyclophomactene (**8**). Bold: ^{1}H,^{1}H-COSY, single headed arrows: key HMBC, and blue double headed arrows: NOESY correlations. Carbon numbering follows GGPP numbering to indicate the origin of each carbon.

Table S9. NMR data of cyclophomactene (**8**) in C_6D_6 recorded at 298 K.

C[a]	type	^{13}C[b]	^{1}H[b]
1	CH_2	26.20	0.77 (dd, *J* = 3.8, 4.3)<br>0.66 (m)
2	CH	37.55	0.90 (m)
3	C_q	150.40	–
4	CH_2	40.76	2.54 (dt, *J* = 12.8, 4.2)<br>1.98 (dd, *J* = 13.6, 4.5)
5	CH_2	26.09	1.78 (m)<br>1.51 (m)
6	CH_2	36.06	2.02 (m)<br>1.17 (dt, *J* = 2.4, 2.4)
7	C_q	31.84	–
8	CH_2	36.79	1.63 (d, *J* = 5.4)<br>1.30 (m)
9	CH_2	30.81	1.96 (m)<br>0.90 (m)
10	C_q	36.53	–
11	CH	38.38	1.37 (m)
12	CH_2	26.38	1.89 (m)<br>1.32 (m)
13	CH_2	35.13	2.41 (m)<br>0.49 (ddd, *J* = 1.6, 10.4, 14.7)
14	C_q	25.28	–
15	CH	41.54	1.76 (m)
16	CH_2	29.24	2.26 (dt, *J* = 15.0, 2.5)<br>0.93 (m)
17	CH_3	25.37	0.92 (s)
18	CH_3	19.19	1.12 (d, *J* = 7.2)
19	CH_3	31.54	0.86 (s)
20	CH_2	110.07	4.90 (br s)<br>4.86 (m)

[a] Carbon numbering as shown in Figure S23. [b] Chemical shifts δ in ppm, multiplicity: s = singlet, m = multiplet, br = broad peak, d = doublet, t = triplet, coupling constants *J* are given in Hertz.

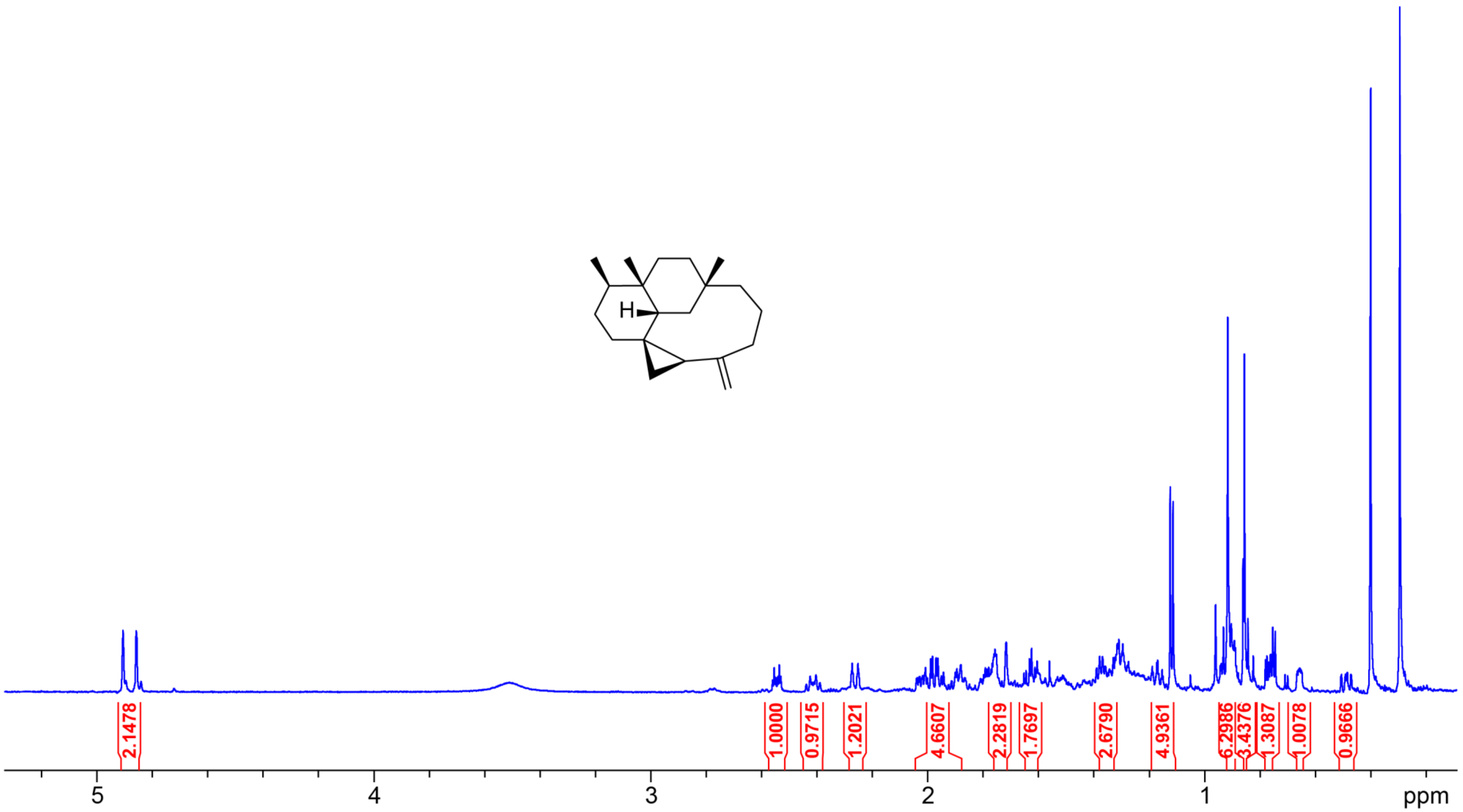

Figure S24. ^{1}H-NMR spectrum of **8** (700 MHz, C_6D_6).

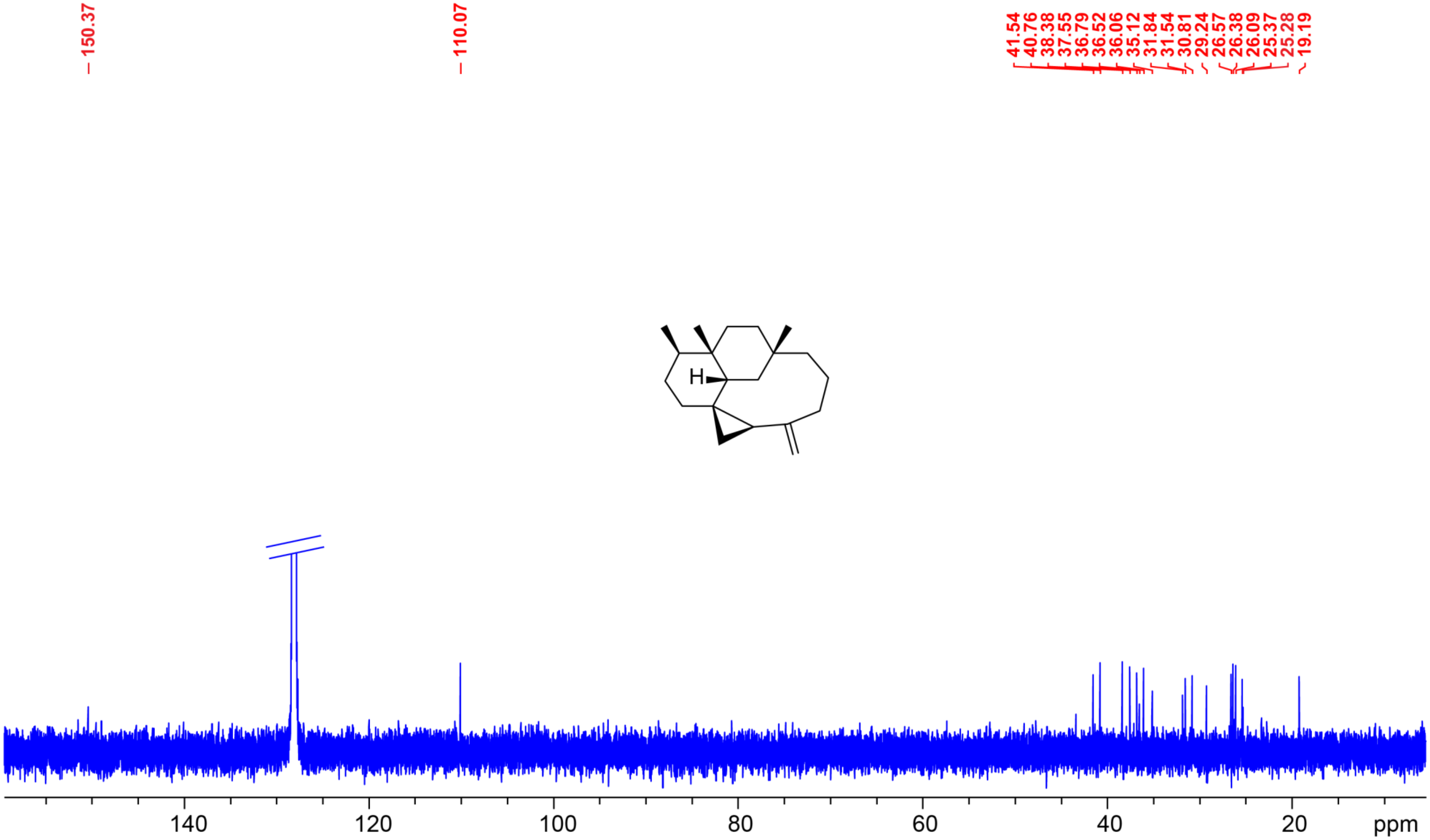

Figure S25. ^{13}C-NMR spectrum of **8** (176 MHz, C_6D_6).

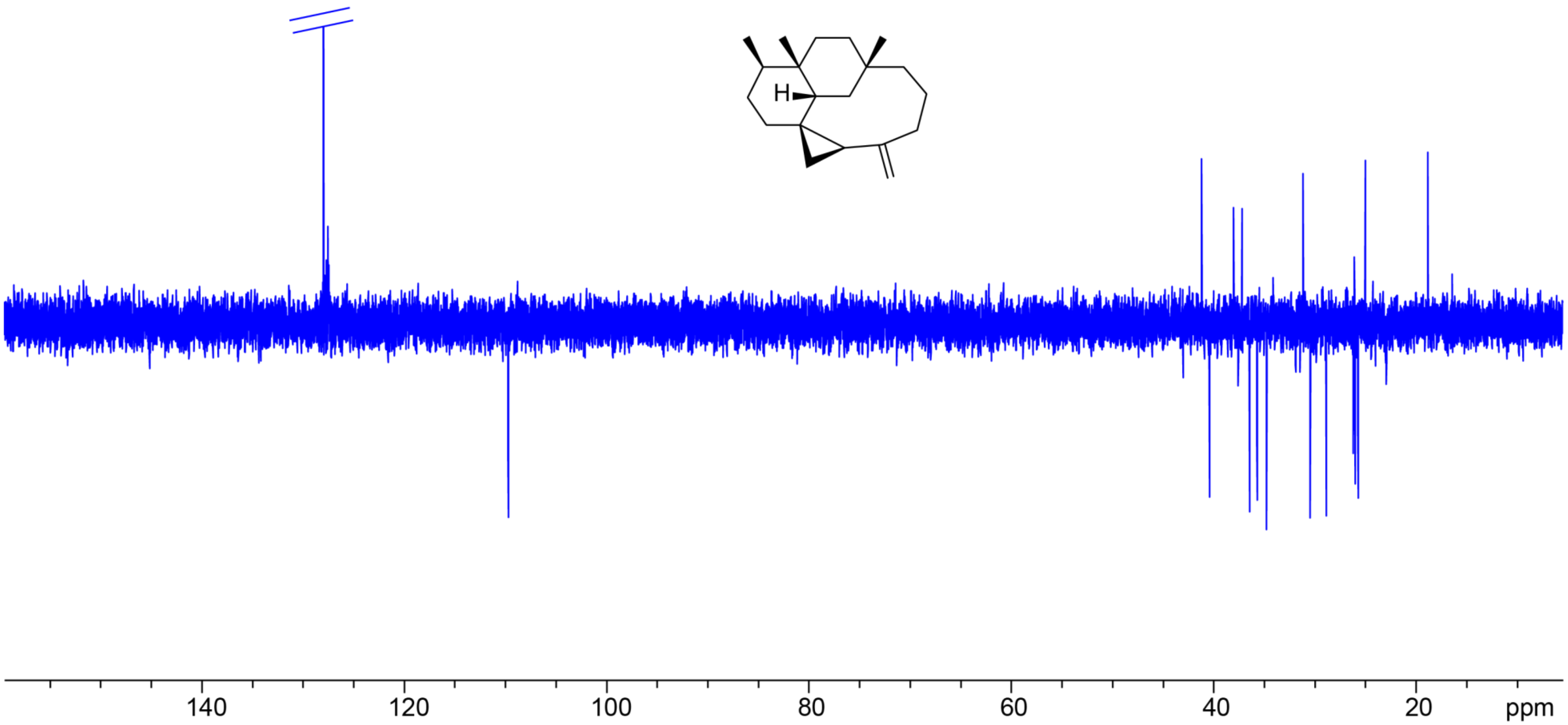

Figure S26. ^{13}C-DEPT135 spectrum of **8** (176 MHz, C_6D_6).

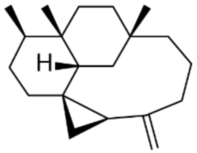

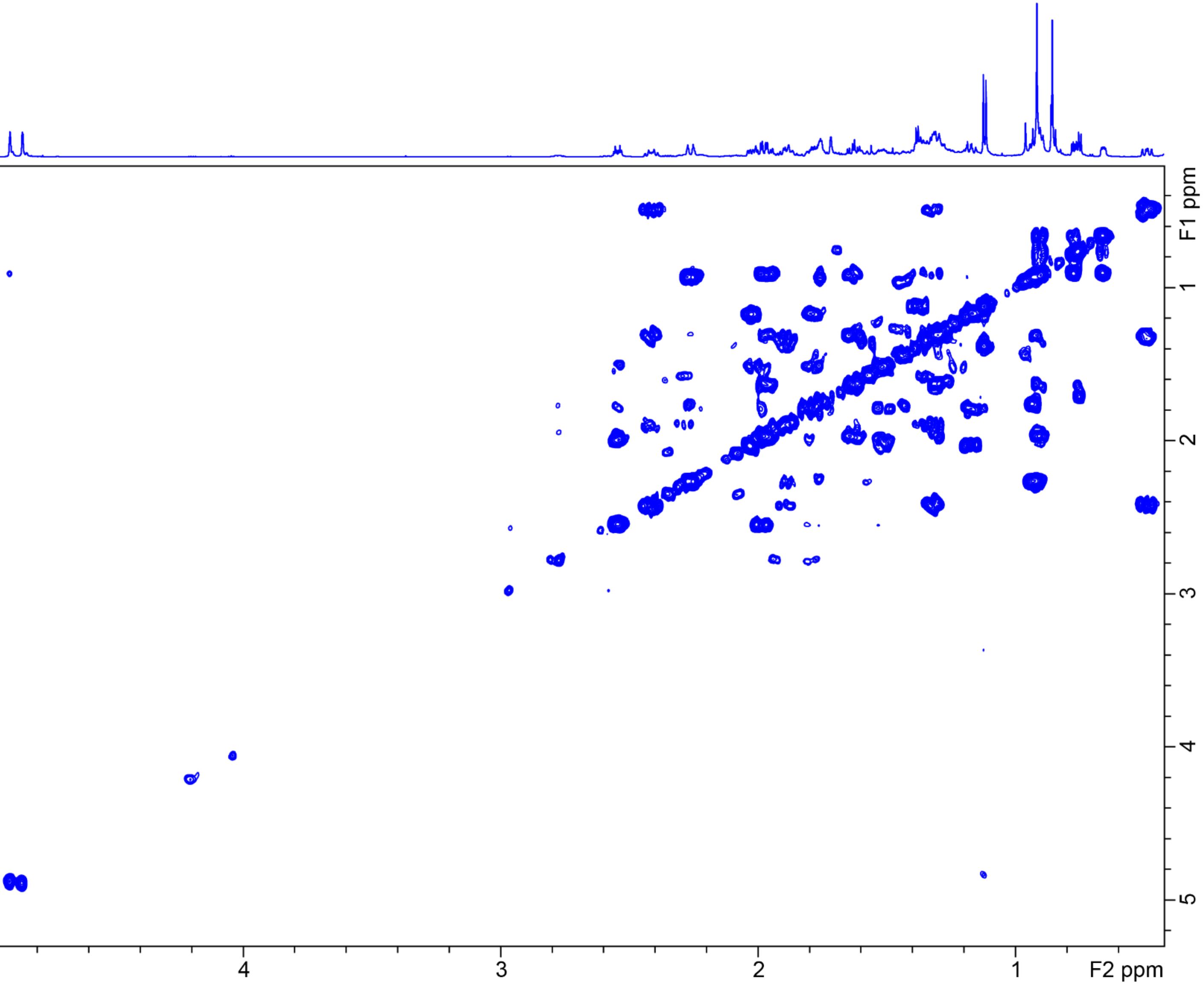

Figure S27. ^{1}H-^{1}H-COSY spectrum (C_6D_6) of **8**.

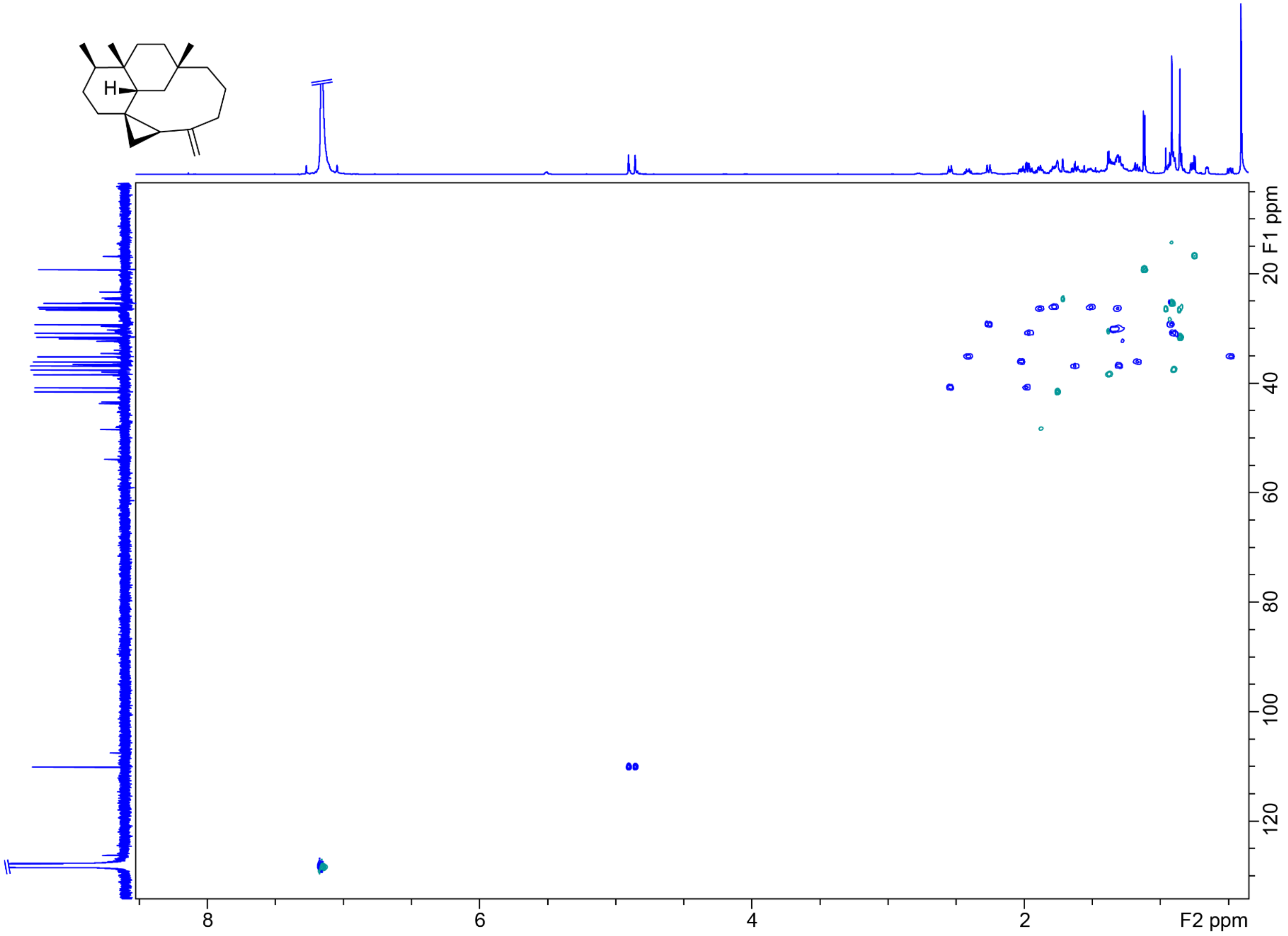

Figure S28. HSQC spectrum (C_6D_6) of **8**.

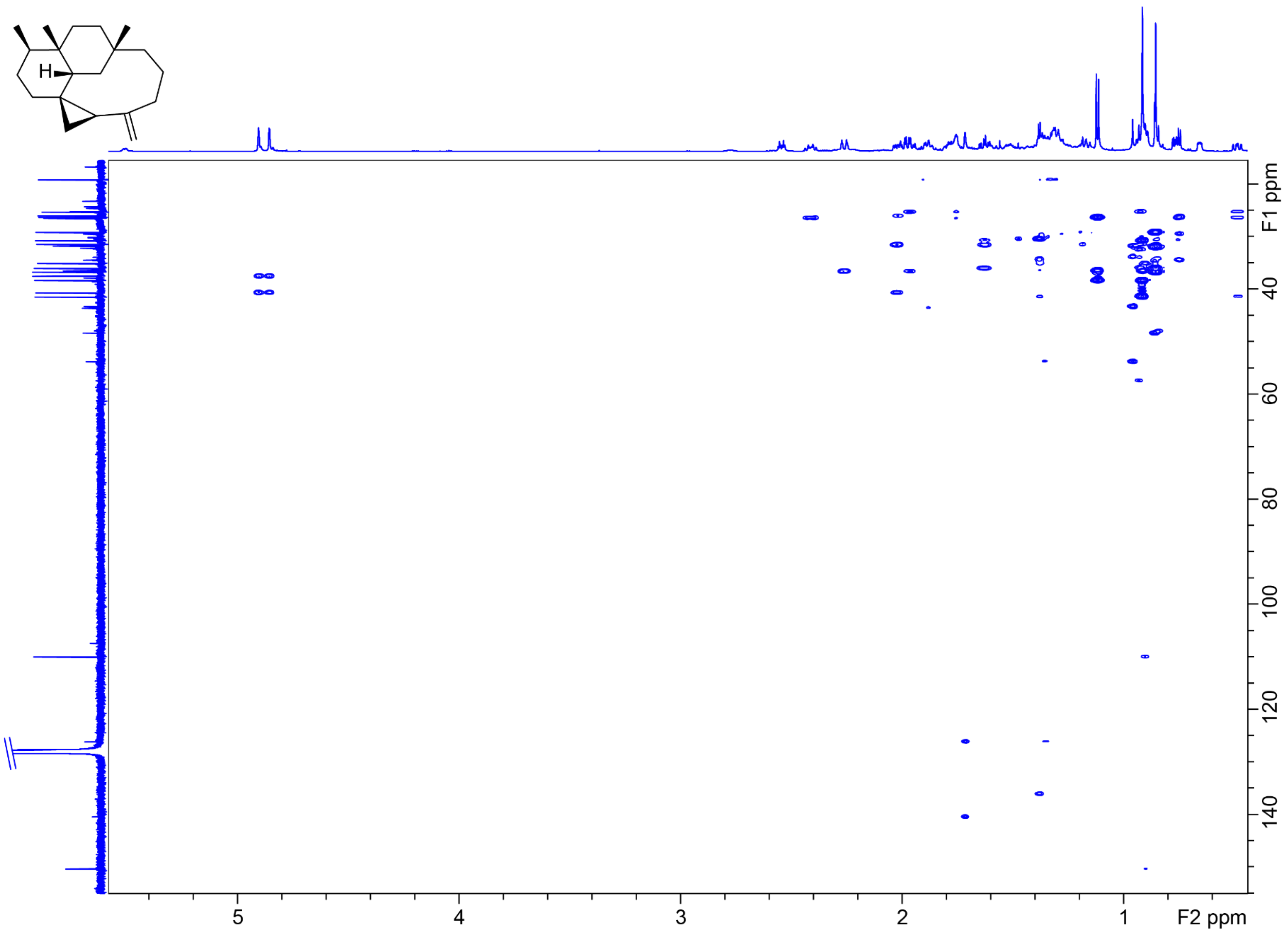

Figure S29. HMBC spectrum (C_6D_6) of **8**.

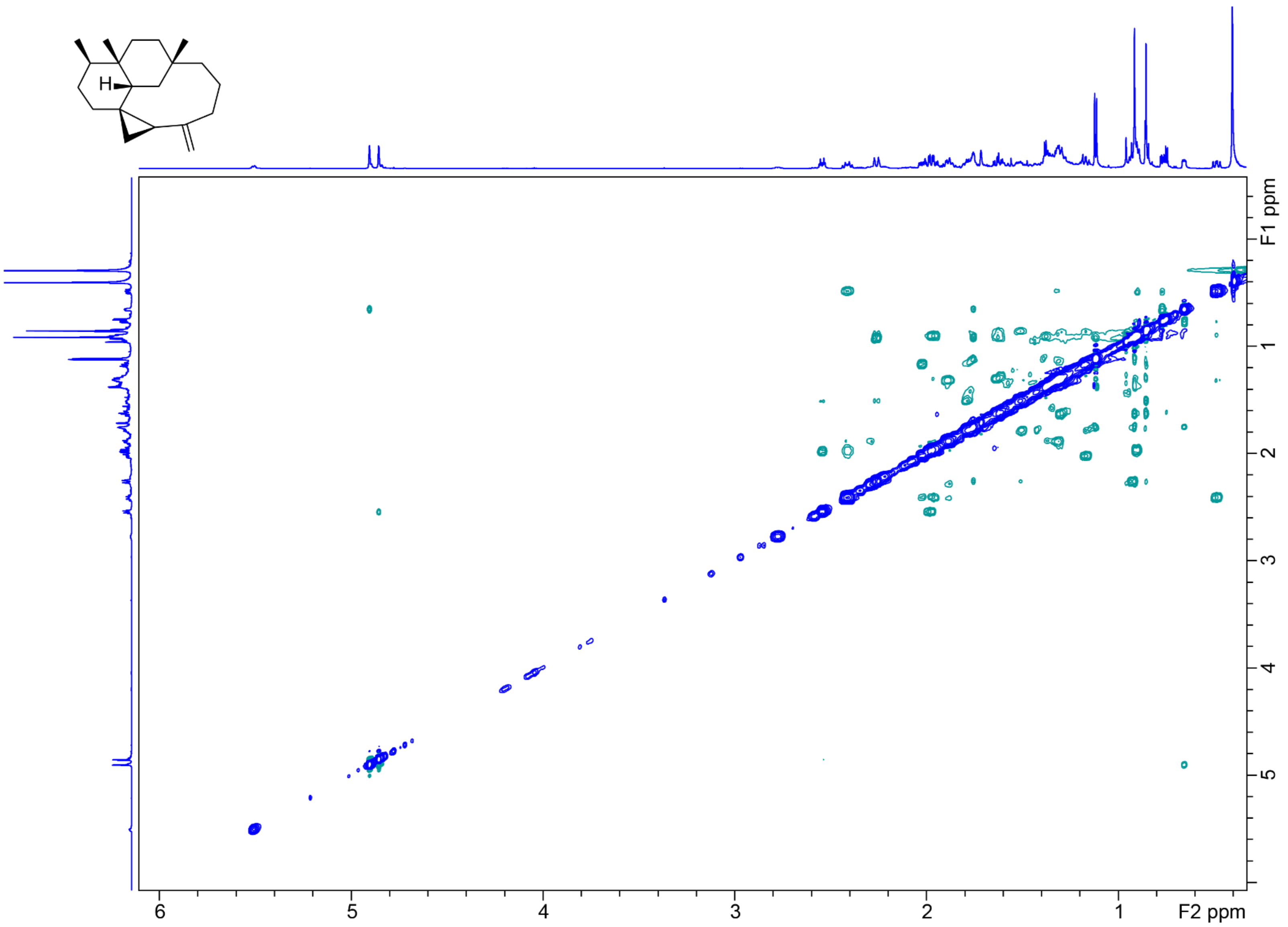

Figure S30. NOESY spectrum (C_6D_6) of **8**.

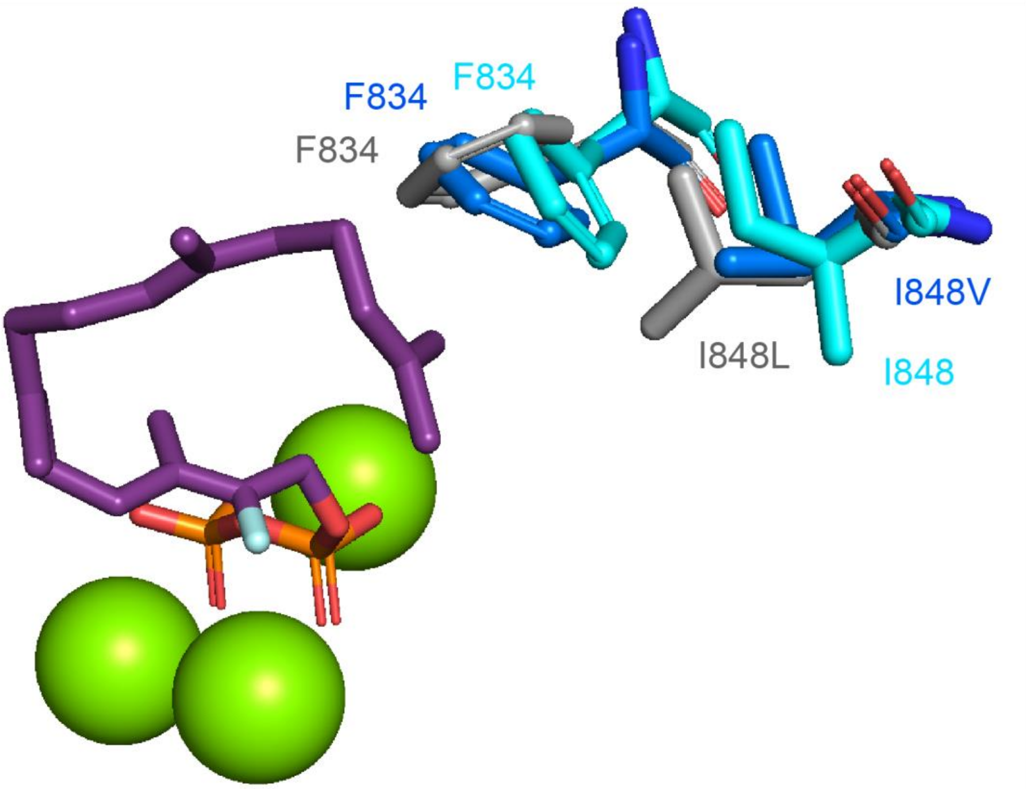

Figure S31. Structural interpretation of the results obtained with the I848L and I848V enzyme variants. Interaction between the active site residues F834 and I848 of TxS. For the wildtype (turquoise) is oriented to accept a cation-π interaction with the substrate after the initial 1,15-cyclisation. In the I848V variant (blue) the Val residue disturbs the F834 residue slightly, explaining its reduced productivity, and in the I848L variant (grey) F834 is strongly disturbed by Leu and is rotated by ca. 90°, explaining the loss of activity of this variant. The substrate analog 2-fluoro-GGPP is shown in purple and green spheres represent Mg^{2+}. The figure is based on the crystal structure of TxS (PDB 3P5R)[9] and on AlphaFold2 models of the I848V and I848L variants.

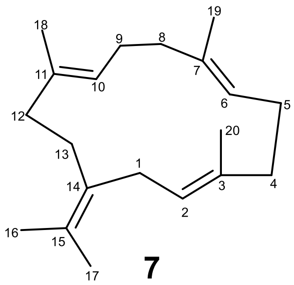

7

Figure S32. Carbon numbering of cembrene D (**7**).

Table S10. NMR data of cembrene D[18] (**7**) recorded in C_6D_6 at 298 K.

C[a]	type	^{13}C[b]	^{1}H[b]
1	CH_2	31.06	2.91 (m, 2H)
2	CH	125.34	5.18 (m)
3	C_q	133.70	–
4	CH_2	39.32	2.13 (m, 2H)
5	CH_2	25.35	2.17 (m, 2H)
6	CH	126.81	5.05 (m)
7	C_q	133.06	–
8	CH_2	40.36	2.07 (m, 2H)
9	CH_2	24.30	2.10 (m, 2H)
10	CH	124.67	5.16 (m)
11	C_q	134.84	–
12	CH_2	37.94	2.09 (m, 2H)
13	CH_2	31.94	2.30 (m, 2H)
14	C_q	131.70	–
15	C_q	124.74	–
16	CH_3	20.86	1.70 (br s)
17	CH_3	20.69	1.67 (br s)
18	CH_3	16.04	1.60 (br s)
19	CH_3	15.09	1.52 (br s)
20	CH_3	15.45	1.55 (br s)

[a] Carbon numbering as shown in Figure S32. [b] Chemical shifts δ in ppm, multiplicity: s = singlet, m = multiplet, br = broad, coupling constants J are given in Hertz.

Table S11. Results of DFT calculations for the cyclisation mechanism from GGPP to **1 – 8** by TxS and its enzyme variants (Scheme 1 of main text).

Structure	Gibbs energy (298.15K) in Hartree	energy relative to A in kcal/mol	reaction barrier in kcal/mol	Gibbs free energy in kcal/mol
**A**	–781.676118	0.00		
**A-B1-TS**	–781.675917	0.13	0.13	
**B1**	–781.691726	–9.79		–9.79
**B2**	–781.714954	–24.37		
**B2-C-TS**	–781.713618	–23.53	0.84	
**C**	–781.726425	–31.57		–7.20
**C**	–781.726430	–31.57		
**C-D-TS**	–781.712686	–22.95	8.62	
**D**	–781.725740	–31.14		0.43
**D**	–781.725746	–31.14		
**D-E1-TS**	–781.712802	–23.02	8.12	
**E1**	–781.723491	–29.73		1.42
**C**	–781.726424	–31.57		
**C-E1-TS**	–781.704421	–17.76	13.81	
**E1**	–781.723487	–29.72		1.84
**E2**	–781.718190	–26.40		
**E2-F-TS**	–781.717813	–26.16	0.24	
**F**	–781.725497	–30.99		–4.59
**C**	–781.726433	–31.57		
**C-G-TS**	–781.715535	–24.73	6.84	
**G**	–781.718708	–26.73		4.85
**G**	–781.718913	–26.85		
**G-H-TS**	–781.713750	–23.61	3.24	
**H**	–781.717552	–26.00		0.85
**H**	–781.717611	–26.04		
**H-J1-TS**	–781.695492	–12.16	13.88	
**J1**	–781.738751	–39.30		–13.27
**J2**	–781.740568	–40.44		
**J2-K1-TS**	–781.727272	–32.10	8.34	
**K1**	–781.738444	–39.11		1.33
**K2**	–781.730615	–34.20		
**K2-L-TS**	–781.733502	–36.01	–1.81	
**L**	–781.736541	–37.92		–3.72

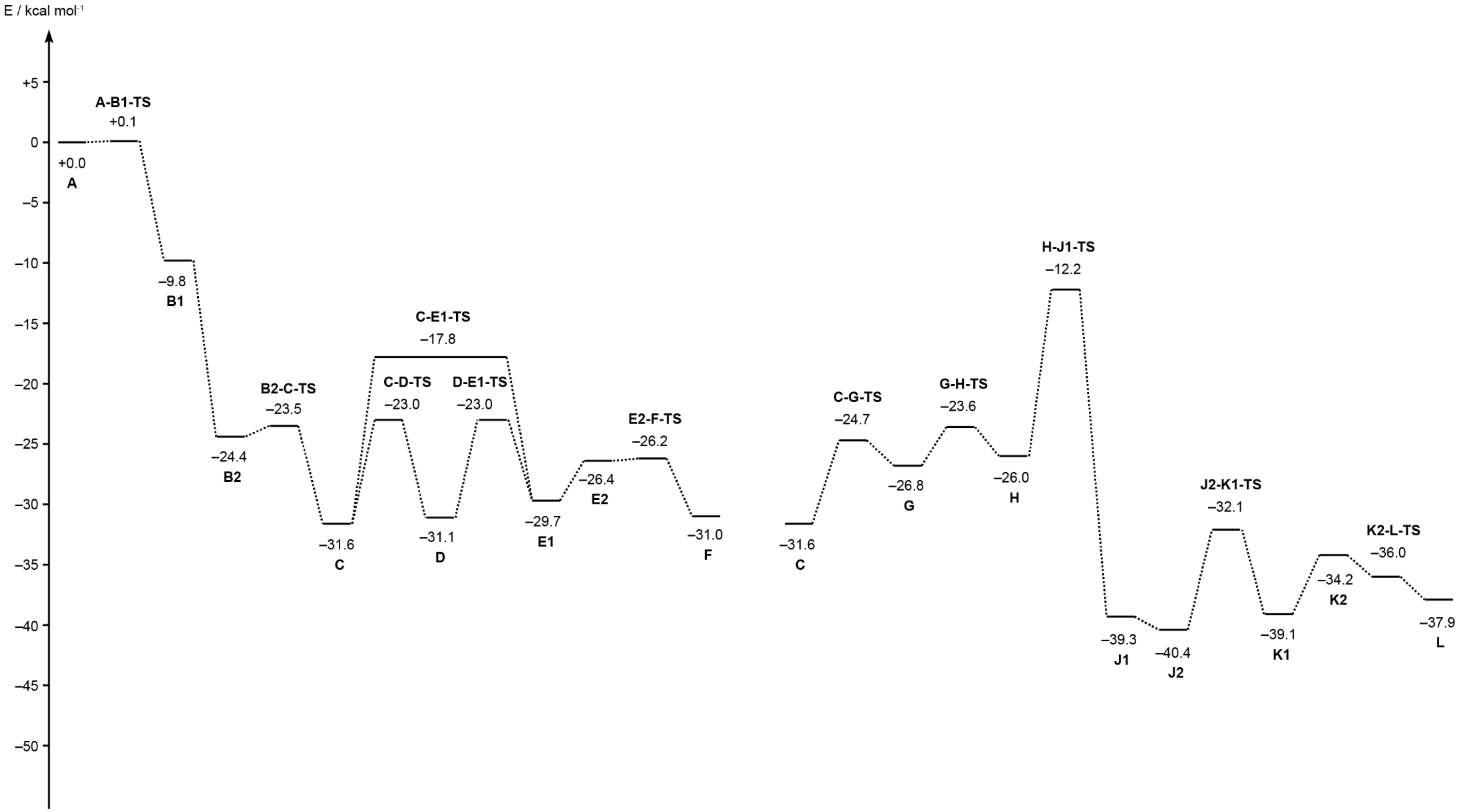

Figure S33. Computed energy profile for the cyclisation mechanism from GGPP to **1 – 8** by TxS and its enzyme variants (Scheme 1 of main text, mPW1PW91/6-311+G(d,p)//B97D3/6-31g(d,p), 298 K).

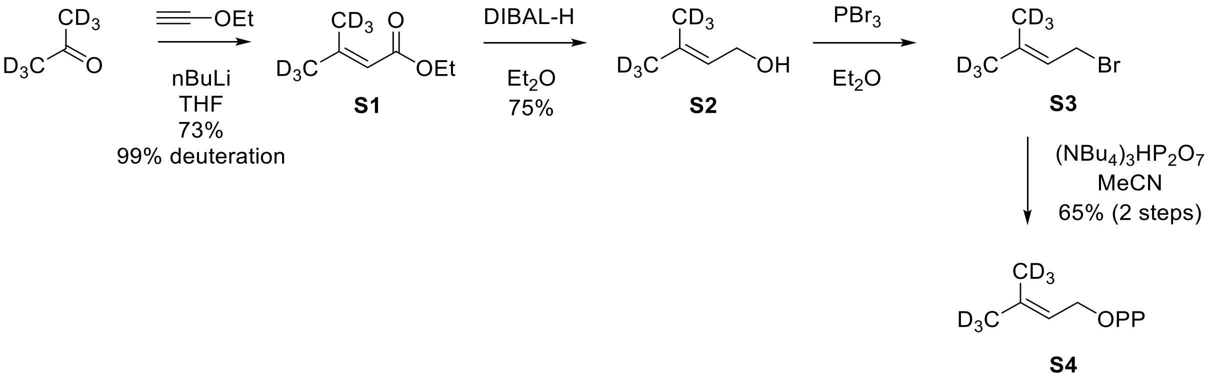

Scheme S1. Synthetic route to (4,4,4,5,5,5-$^2H_6$)DMAPP.

Synthesis of ethyl 3-(methyl-d3)but-2-enoate-4,4,4-d3 (S1)
To a cooled (–78 °C) solution of ethoxyethyne (5.00 g, 34.0 mmol, 40% wt in hexane, 2.50 eq) in dry THF (30 mL) was added *n*BuLi (21 mL, 1.6 M in hexane, 2.50 eq) slowly. The reaction mixture was stirred for 2 h at –78 °C, followed by the addition of acetone-d6 (800 mg, 13.8 mmol, 1.00 eq) at –40 °C. After stirring overnight at –40 °C, the reaction mixture was warmed to –10 °C. Then D_2SO_4 (10% in D_2O, 15 mL, 27.6 mmol, 2.00 eq) was added slowly, and the reaction mixture was stirred at room temperature for 4 h. Sat. $NaHCO_3$ (20 mL) was added to quench the reaction and the mixture was extracted with Et_2O (3x 100 mL). The extract was dried over $MgSO_4$ and concentrated by evaporation of solvents. The product was purified by column chromatography (petroleum ether:Et_2O = 8:1) to yield **S1** as pale yellow oil. TLC (petroleum ether: Et_2O = 8:1): R_f = 0.50. GC (HP-5): *I* = 922. ^{1}H-NMR (C_6D_6, 500 MHz): 5.72 (s, 1H), 4.04 (q, 2H, *J* = 7.2 Hz), 1.01 (t, 3H, *J* = 7.2 Hz) ppm. ^{13}C-NMR (C_6D_6, 125 MHz): 166.31 (C_q), 155.77 (C_q), 116.85 (CH), 59.32 (CH_2), 26.00 (heptet, CD_3, $^1J_{C,D}$ = 19.5), 19.18 (heptet, CD_3, $^1J_{C,D}$ = 19.3), 14.44 (CH_3) ppm.

Synthesis of 3-(methyl-d3)but-2-en-4,4,4-d3-1-ol (S2)
To a cooled (0 °C) solution of the ester **S1** (1.34 g, 10.0 mmol, 1.00 eq) in Et_2O (10 mL) was added DIBAL-H (624 mL, 24.0 mmol, 1.0 M in hexane, 2.40 eq) and the reaction mixture was stirred for 1 h at room temperature. The mixture was cooled to 0 °C and a saturated solution of Na-K-tartrate (20 mL) was added. The resulting slurry was stirred for 1 h to dissolve the precipitate and the aqueous phase was extracted with Et_2O (3x 50 mL). The organic layers were dried with $MgSO_4$ and concentrated under reduced pressure. The residue was purified by column chromatography (petroleum ether/Et_2O, 2:1) to yield the alcohol **S2** (694 mg, 7.54 mmol, 75%) as a colorless oil. TLC (petroleum ether: Et_2O = 2:1): R_f = 0.44. ^{1}H-NMR (C_6D_6, 500 MHz): 5.39 (d, 1H, *J* = 2.6 Hz), 4.61 (br d, 2H, *J* = 6.7 Hz) ppm. ^{13}C-NMR (C_6D_6, 125 MHz): 133.43 (C_q), 125.22 (CH), 59.26 (CH_2), 24.78 (heptet, CD_3, $^1J_{C,D}$ = 19.4 Hz), 16.88 (heptet, CD_3, $^1J_{C,D}$ = 19.4 Hz) ppm.

Synthesis of (4,4,4,5,5,5-$^2H_6$)DMAPP (S4)
To a cooled (0 °C) solution of **S2** (694 mg, 7.54 mmol, 1.00 eq) in CH_2Cl_2 (10 mL) PBr_3 (812 mg, 3.0 mmol, 0.40 eq) was added dropwise. The mixture was stirred for 1 h at 0 °C and then poured onto an ice/water mixture. The aqueous layer was extracted with Et_2O (3x 20 mL), the organic layers were dried with $MgSO_4$ and concentrated under reduced pressure. The bromide **S3** (ca. 400 mg) was directly used for the next reaction without purification.
To a solution of tris(tetra-n-butylammonium)hydrogen diphosphate (5.0 g, 5.57 mmol, 0.74 eq) in acetonitrile (10 mL) a solution of the allyl bromide **S3** was added and the mixture was stirred at room temperature overnight. Acetonitrile was removed under reduced pressure. The residue was dissolved in aqueous NH_4HCO_3 solution (1 mL, 0.25 M) and loaded onto a DOWEX 50WX8 ion-exchange column (NH_4^+ form, pH 7.0). The column was flushed slowly with 1.5

column volumes of NH_4HCO_3 buffer (25 mM, 5% iPrOH) and the eluate was lyophilised to yield the diphosphate **S4** as a colourless hygroscopic powder (1.47 g, 4.88 mmol, 65%). ^{1}H-NMR (D_2O, 500 MHz): 5.22 (qd, 1H, J = 7.7, 2.1 Hz), 4.78 (m, 1H, overlapped with D_2O), 4.23 (1H, 7.0, J = 7.0 Hz) ppm. ^{13}C-NMR (D_2O, 125 MHz): 139.86 (C_q), 119.32 (CH, d, $^3J_{C,P}$ = 7.6 Hz), 62.56 (CH_2, d, $^2J_{C,P}$ = 5.4 Hz), 23.97 (heptet, CD_3, $^1J_{C,D}$ = 19.9 Hz), 16.37 (heptet, CD_3, $^1J_{C,D}$ = 19.1 Hz) ppm. ^{31}P-NMR (D_2O, 203 MHz): –8.35 (d, $^2J_{P,P}$ = 20.7 Hz), –10.43 (d, $^2J_{P,P}$ = 20.5 Hz) ppm.

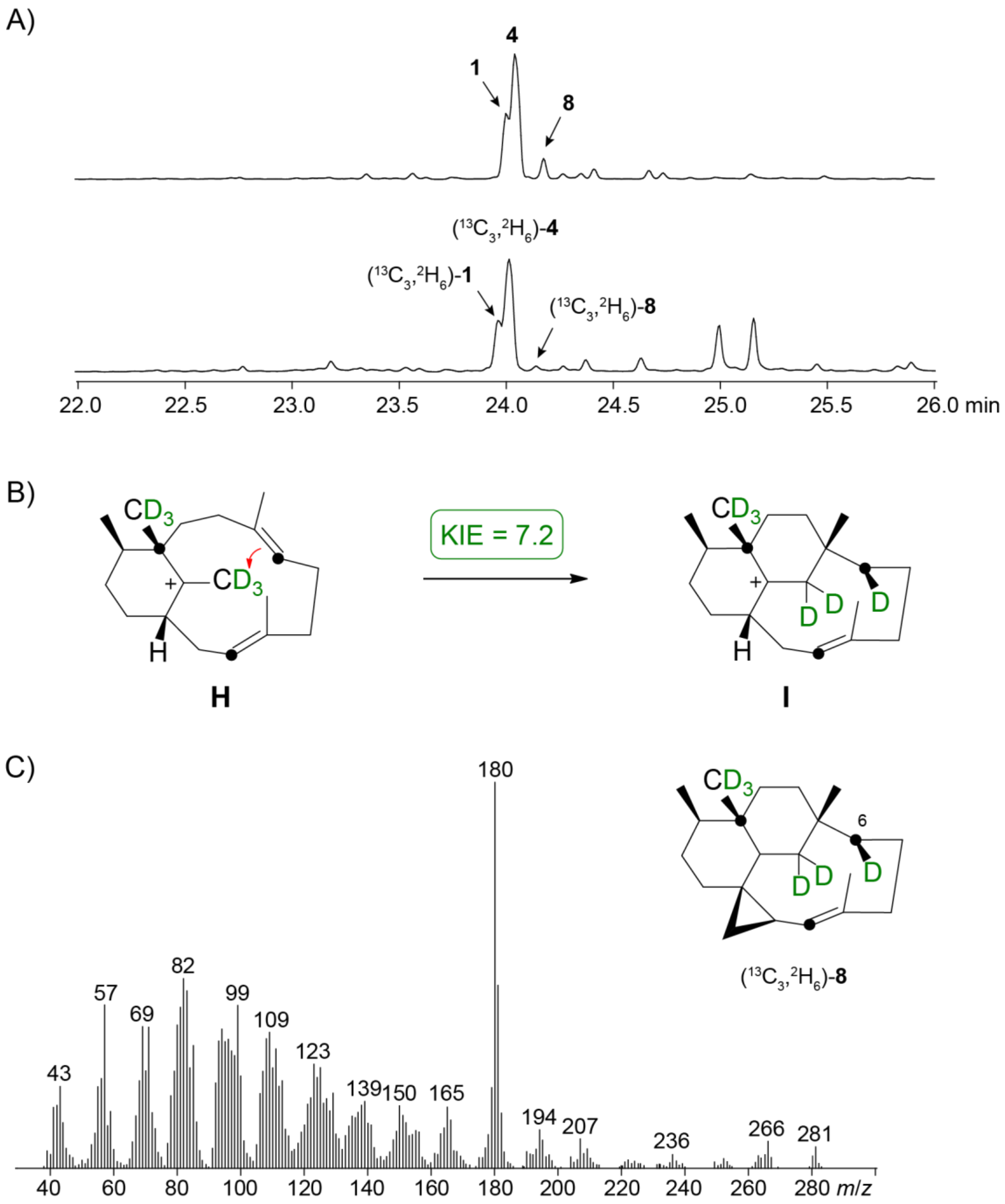

Figure S34. The 1,7-proton shift from **H** to **I** in the biosynthesis of **8**. A) Total ion chromatograms of the products obtained with TxS-G606A and GGPPS from DMAPP and IPP (top) and from (4,4,4,5,5,5-$^{2}H_{6}$)DMAPP and (2-^{13}C)IPP, showing a strongly reduced production of labelled **8** as a result of a kinetic isotope effect. B) The proton migration from **H** to **I** explains the strong kinetic isotope effect in the formation of **8** (for full cyclisation mechanism cf. Scheme 1 of main text). C) The reduced production of labelled **8** prevented the detection of a triplet signal in the ^{13}C-NMR spectrum for C6 of **8**, but the EI mass spectrum of ($^{13}C_{3}$,$^{2}H_{6}$)-**8** obtained from (4,4,4,5,5,5-$^{2}H_{6}$)DMAPP and (^{13}C)IPP shows retainment of all six deuterium atoms in the product by the molecular ion at *m*/*z* 281 (unlabelled: *m*/*z* 272), in agreement with the cyclisation mechanism of Scheme 1 of main text.

A)

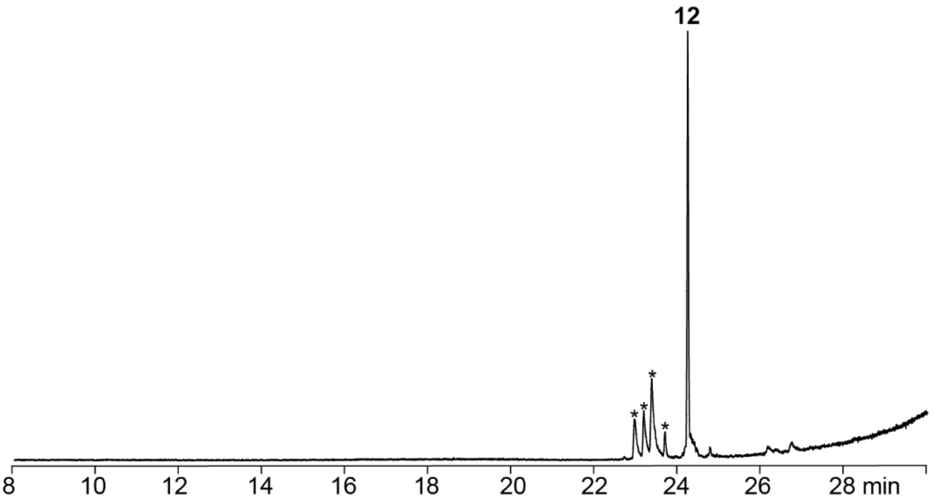

B)

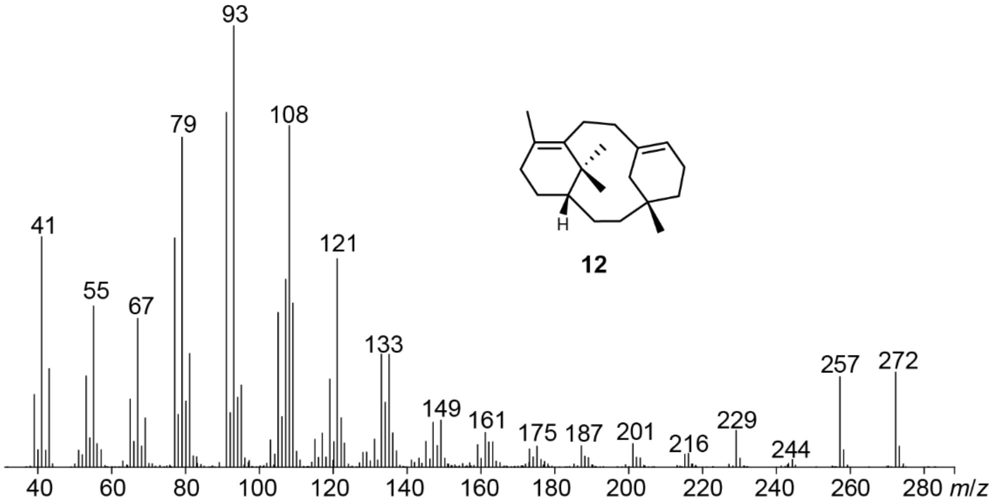

Figure S35. GC/MS analysis of the products obtained with TxS from *iso*-GGPP I. A) Total ion chromatogram of a crude extract from an enzyme incubation of *iso*-GGPP I with TxS, B) EI mass spectrum of taxaxenene (**12**).

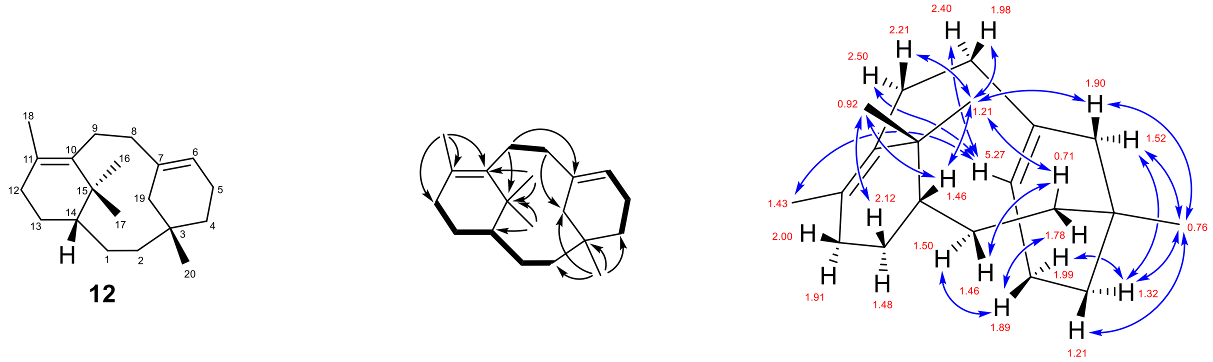

Figure S36. Structure elucidation of taxaxenene (**12**). Bold: ^{1}H,^{1}H-COSY, single headed arrows: key HMBC, and double headed arrows: NOESY correlations. Carbon numbering follows GGPP numbering to indicate the origin of each carbon.

Table S12. NMR data of taxaxenene (**12**) in $CDCl_3$ recorded at 238 K.

C[a]	type	^{13}C[b]	^{1}H[b]
1	CH_2	26.07	1.50 (m, H_{α})
			1.46 (m, H_{β})
2	CH_2	27.19	1.78 (m, H_{α})
			0.71 (m, H_{β})
3	C_q	33.24	–
4	CH_2	36.03	1.32 (m, H_{β})
			1.21 (m, H_{α})
5	CH_2	23.29	1.99 (m, H_{β})
			1.89 (m, H_{α})
6	CH	121.45	5.27 (m)
7	C_q	135.14	–
8	CH_2	34.61	2.40 (m, H_{α})
			1.98 (m, H_{β})
9	CH_2	25.06	2.50 (m, H_{α})
			2.21 (m, H_{β})
10	C_q	133.74	–
11	C_q	129.51	–
12	CH_2	29.69	2.00 (m, H_{β})
			1.91 (m, H_{α})
13	CH_2	22.28	2.12 (m, H_{β})
			1.48 (m, H_{α})
14	CH	42.45	1.46 (m)
15	C_q	37.53	–
16	CH_3	34.02	0.92 (s)
17	CH_3	26.80	1.21 (s)
18	CH_3	21.90	1.43 (s)
19	CH_2	38.12	1.90 (m, H_{α})
			1.52 (m, H_{β})
20	CH_3	27.29	0.76 (s)

[a] Carbon numbering as shown in Figure S36. [b] Chemical shifts δ in ppm, multiplicity: s = singlet, m = multiplet, coupling constants J are given in Hertz.

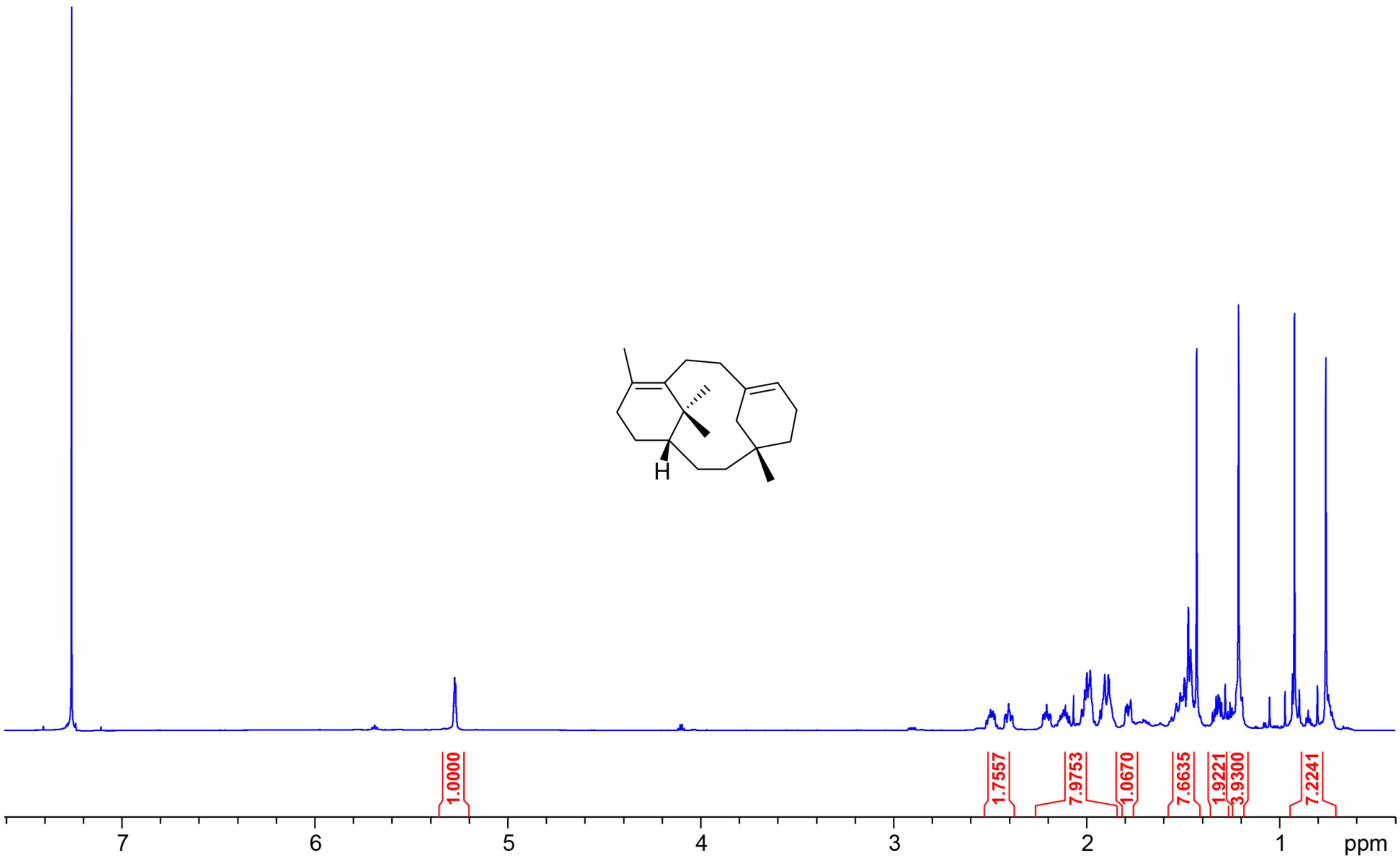

Figure S37. ^{1}H-NMR spectrum of **12** (700 MHz, $CDCl_3$).

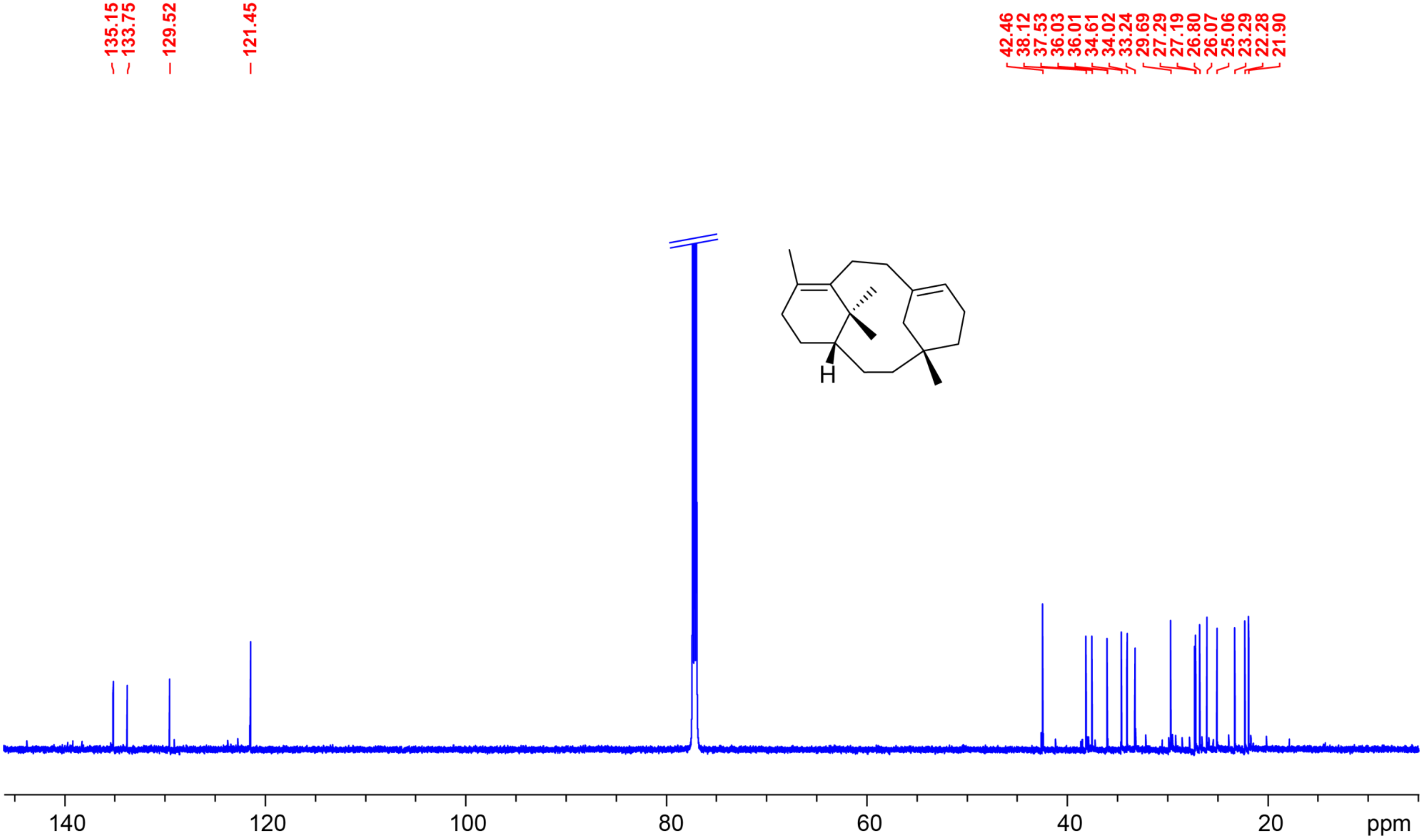

Figure S38. ^{13}C-NMR spectrum of **12** (176 MHz, $CDCl_3$).

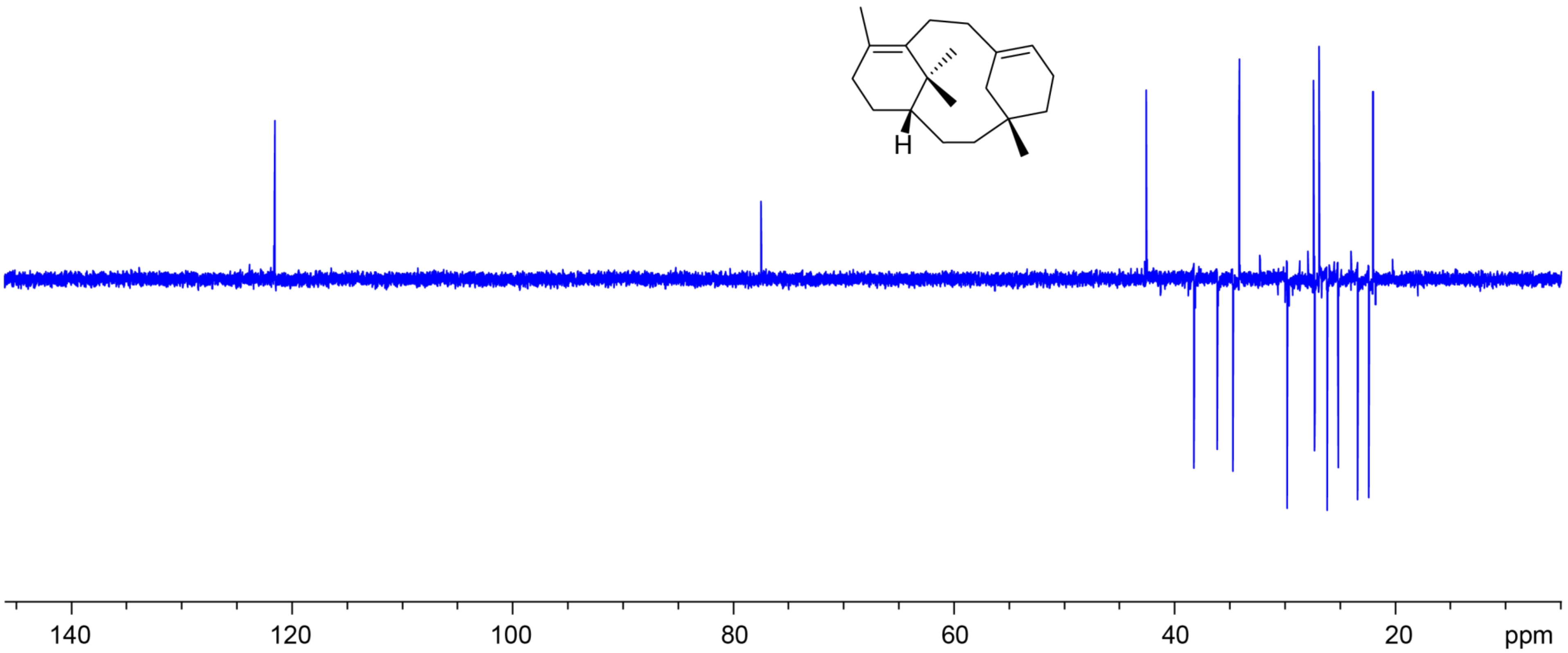

Figure S39. ^{13}C-DEPT135 spectrum of **12** (176 MHz, $CDCl_3$).

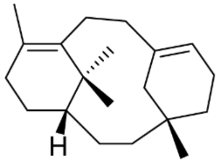

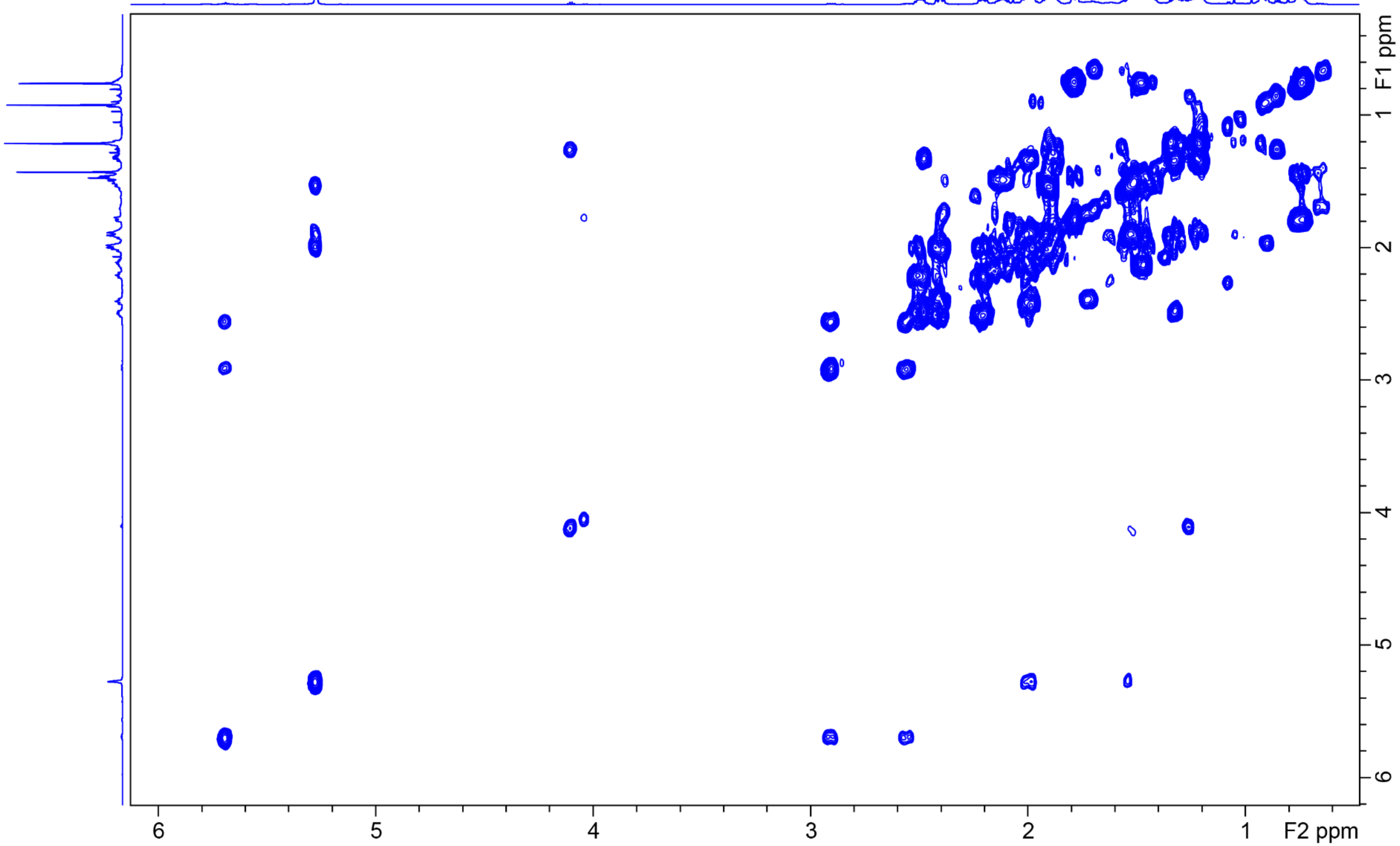

Figure S40. ^{1}H-^{1}H-COSY spectrum ($CDCl_3$) of **12**.

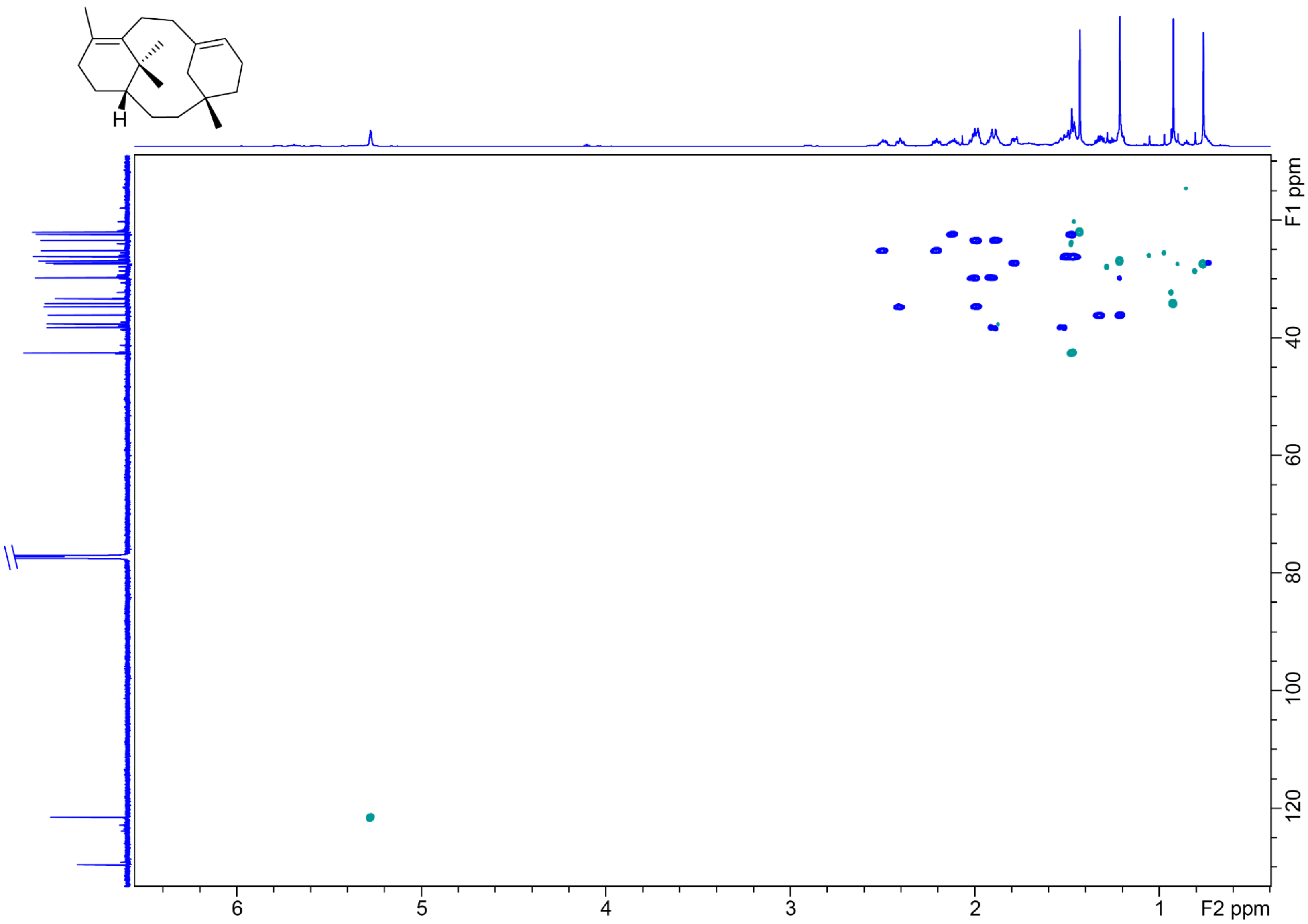

Figure S41. HSQC spectrum ($CDCl_3$) of **12**.

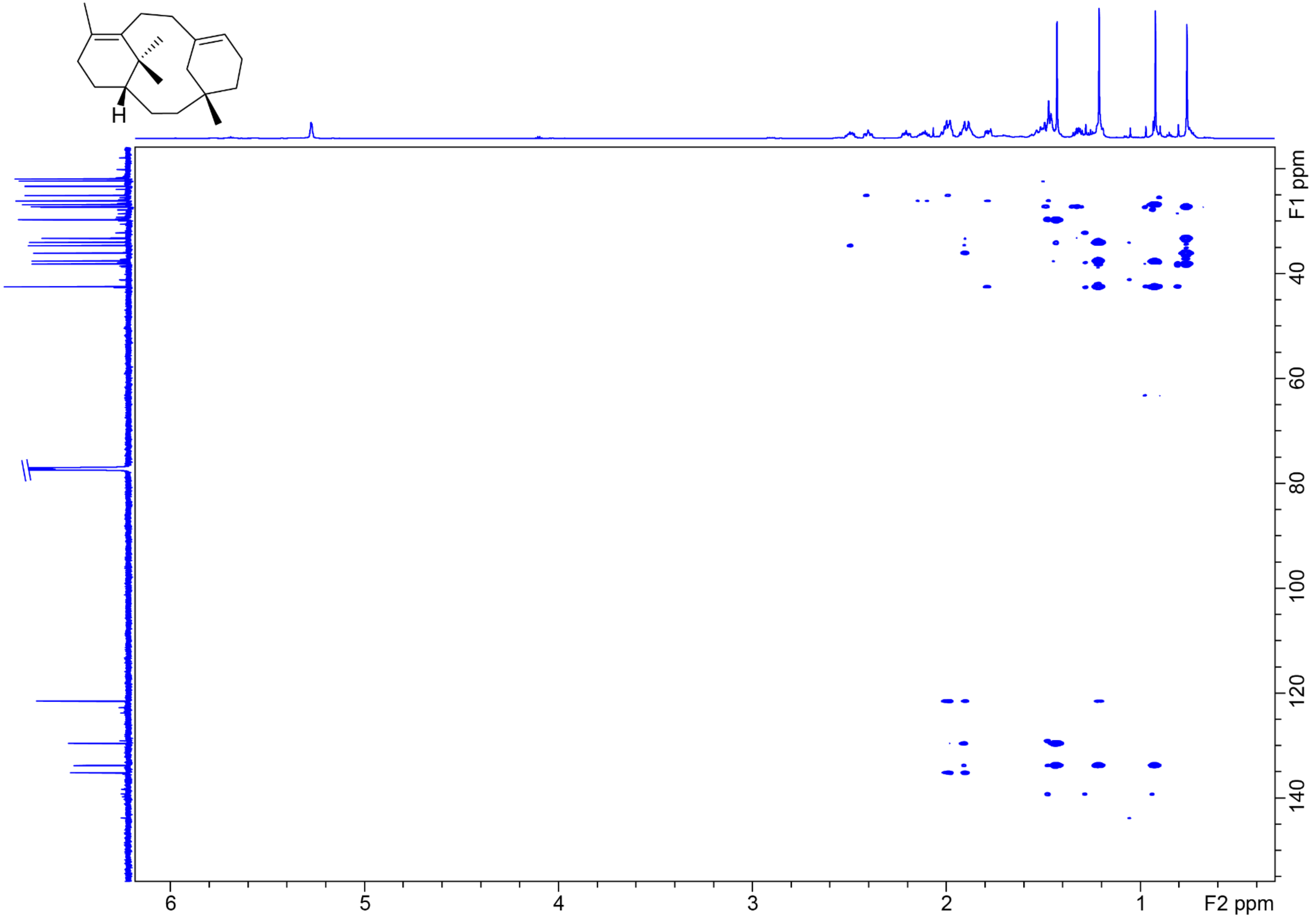

Figure S42. HMBC spectrum ($CDCl_3$) of **12**.

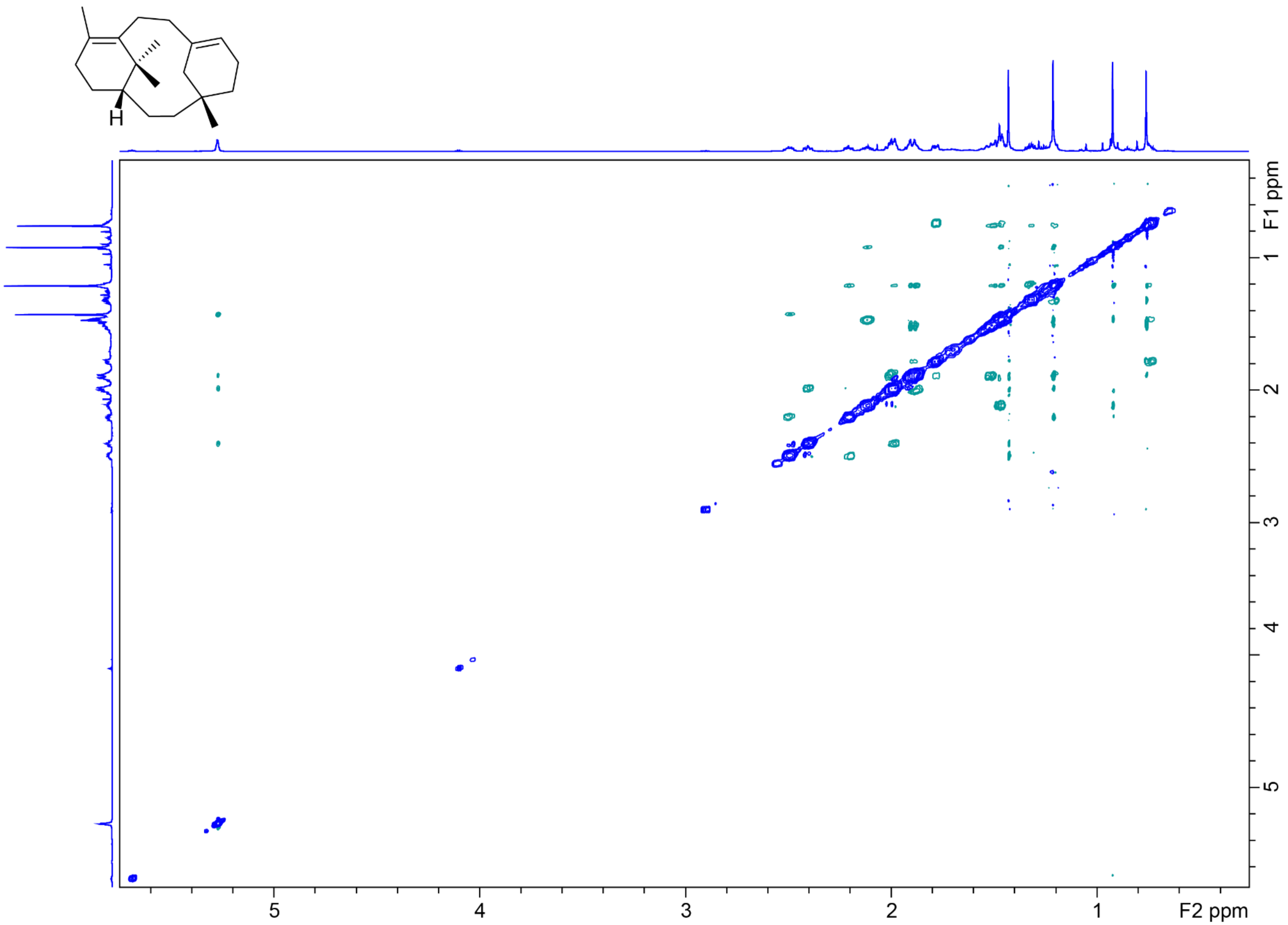

Figure S43. NOESY spectrum ($CDCl_3$) of **12**.

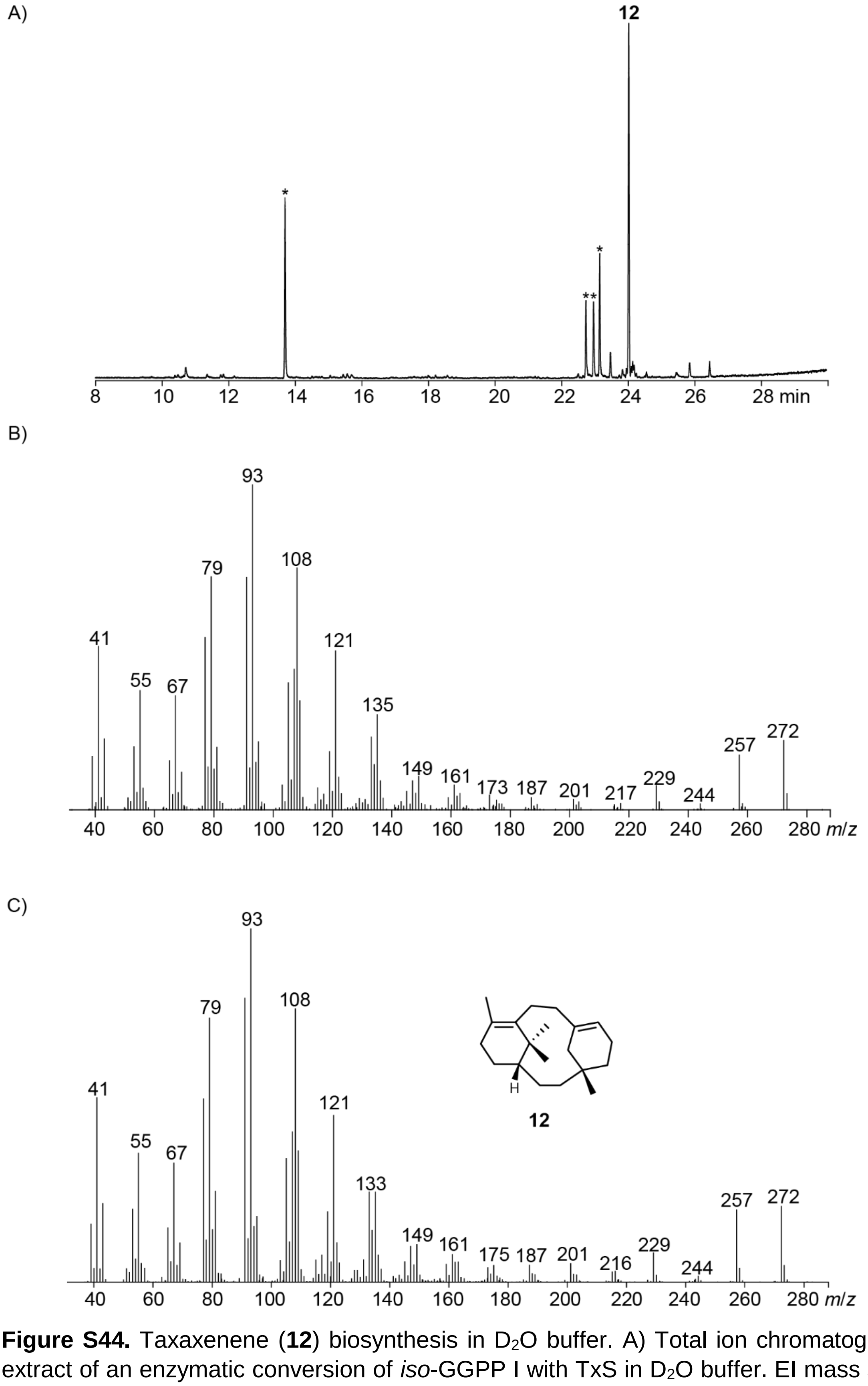

Figure S44. Taxaxenene (**12**) biosynthesis in D_2O buffer. A) Total ion chromatogram of an extract of an enzymatic conversion of *iso*-GGPP I with TxS in D_2O buffer. EI mass spectra of **12** obtained from an incubation in B) D_2O buffer and C) unlabelled buffer. The highly similar mass spectra with molecular ions at *m*/*z* 272 in both cases show no uptake of deuterium from D_2O buffer and thus support a cyclisation mechanism with no reprotonation of a neutral intermediate.

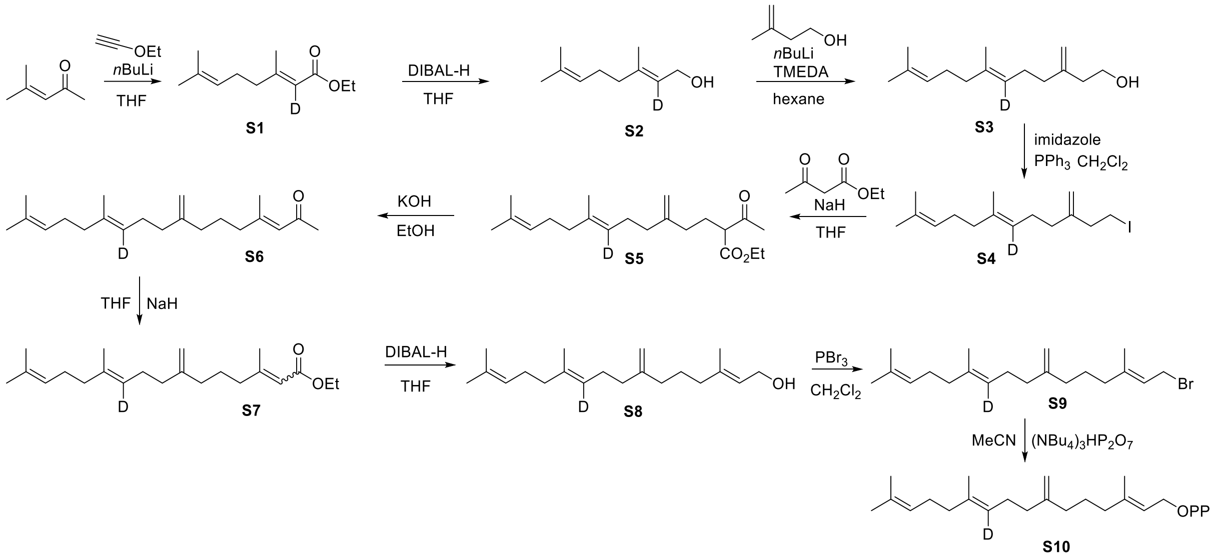

Scheme S2. Synthesis of (10-^{2}H)-*iso*-GGPP I.

Synthesis of (10-^{2}H)-*iso*-GGPP I

Synthesis of ethyl (*E*)-3,7-(2-^{2}H)dimethylocta-2,6-dienoate (S1)

To a solution of ethoxyethyne (4.50 g, 64.0 mmol, 2.50 eq) in cold (–78 °C) and dry THF (100 mL) was added *n*BuLi slowly. The reaction mixture was stirred for 2 h at –78 °C, followed by the addition of 6-methylhept-5-en-2-one (3.24 g, 25.7 mmol, 1.00 eq) at –40 °C. After stirring overnight at –40 °C, the reaction mixture was warmed to –10 °C. Then D_2SO_4 (10% in D_2O, 51 mL, 2.00 eq) was added slowly, and the reaction mixture was stirred at room temperature for 2 h. Sat. $NaHCO_3$ (20 mL) was added to quench the reaction and the mixture was extracted with Et_2O (150 mL, 3 times). The extract was dried over $MgSO_4$ and concentrated by evaporation of solvents. The product was purified by column chromatography (petroleum ether:Et_2O = 8:1) to yield (*E*)-**S1** and (*Z*)-**S1** as pale yellow oils.

(*E*)-**S1**: 1.33 g (6.75 mmol, 26%, 97% deuteration). TLC (petroleum ether:Et_2O = 8:1): R_f = 0.78. GC (HP-5): *I* = 1397. ^{1}H-NMR (C_6D_6, 500 MHz): 5.02 (tq, 1H, *J* = 7.1, 1.4 Hz), 4.04 (qd, 2H, *J* = 7.1, 0.9 Hz), 2.20 (s, 3H), 1.98 (m, 2H), 1.90 (m, 2H), 1.60 (br s, 3H), 1.45 (br s, 3H), 1.01 (t, 3H, *J* = 7.0 Hz) ppm. ^{13}C-NMR (C_6D_6, 125 MHz): 166.48 (C_q), 159.29 (C_q), 132.14 (C_q), 123.65 (CH), 116.35 (CH, t, $^1J_{C,D}$ = 39.8 Hz), 59.37 (CH_2), 40.94 (CH_2), 26.23 (CH_2), 25.74 (CH_3), 18.75 (CH_3), 17.65 (CH_3), 14.44 (CH_3) ppm.

(*Z*)-**S1**: 1.05 g (5.18 mmol, 20%, 97% deuteration) TLC (petroleum ether:Et_2O = 8:1): R_f = 0.74. GC (HP-5): *I* = 1355. ^{1}H-NMR (C_6D_6, 500 MHz): 5.27 (tq, 1H, *J* = 7.2, 1.4 Hz), 4.03 (q, 2H, *J* = 7.1 Hz), 2.79 (m, 2H), 2.22 (m, 2H), 1.64 (m, 3H), 1.60 (m, 3H), 1.54 (s, 3H), 1.00 (t, 3H, *J* = 7.1 Hz) ppm. ^{13}C-NMR (C_6D_6, 125 MHz): 166.02 (C_q), 159.81 (C_q), 132.03 (C_q), 124.41 (CH), 116.61 (CH, t, $^1J_{C,D}$ = 23.9 Hz), 59.34 (CH_2), 33.71 (CH_2), 27.53 (CH_2), 25.83 (CH_3), 25.01 (CH_3), 17.69 (CH_3), 14.43 (CH_3) ppm.

Synthesis of (*E*)-3,7-(2-^{2}H)dimethylocta-2,6-dien-1-ol (S2)

To a cooled (0 °C) solution of the ester (*E*)-**S1** (1.33 g, 6.75 mmol, 1.00 eq) in THF (20 mL) was added DIBAL-H (9.21 g, 16.2 mmol, 25 wt-% in toluene, 2.40 eq) and the reaction mixture was stirred for 1 h at room temperature. The mixture was cooled to 0 °C and a saturated solution of Na-K-tartrate (20 mL) was added. The resulting slurry was stirred for 2 h to dissolve the precipitate and the aqueous phase was extracted three times with Et_2O (100 mL). The organic layers were dried with $MgSO_4$ and concentrated under reduced pressure. The residue was purified by column chromatography (petroleum ether:Et_2O = 3:1) to yield the alcohol **S2** (1.0 g, 6.5 mmol, 96%) as a colourless oil. TLC (petroleum ether:Et_2O = 2:1): R_f = 0.25. GC (HP-5): *I* = 1264. ^{1}H-NMR (C_6D_6, 500 MHz): 5.17 (m, 1H), 3.97 (s, 2H), 2.10 (m, 2H), 1.99 (m, 2H), 1.66 (m, 3H), 1.53 (m, 3H), 1.46

(s, 3H) ppm. ^{13}C-NMR (C_6D_6, 125 MHz): 138.02 (C_q), 131.45 (C_q), 124.59 (CH), 124.76 (CH, t, $^1J_{C,D}$ = 23.46 Hz), 59.30 (CH_2), 39.83 (CH_2), 26.83 (CH_2), 25.85 (CH_3), 17.74 (CH_3), 16.16 (CH_3) ppm.

Synthesis of (*E*)-7,11-(6-^{2}H)dimethyl-3-methylenedodeca-6,10-dien-1-ol (S3)

To a cooled (0 °C) solution of **S2** (1.02 g, 6.45 mmol, 1.00 eq) in Et_2O (20mL) PBr_3 (697 mg, 2.58 mmol, 0.40 eq) was added dropwise. The mixture was stirred for 1 h at 0 °C and was transferred directly to an ice/water mixture. The aqueous layer was extracted with Et_2O (50 mL, three times), the organic layers were dried with $MgSO_4$ and concentrated under reduced pressure. The bromide (ca. 1.0 g) was directly used for the next reaction without purification.

A solution of *n*BuLi (10.5 mL, 1.6 M in hexane, 2.60 eq) was diluted with hexane (12 mL) and cooled to –15 °C. TMEDA (1.95 g, 16.8 mmol, 2.60 eq) was added and the mixture was stirred for 0.5 h at –23 °C. The mixture was warmed to room temperature and 3-methylbut-3-en-1-ol (0.72 g, 8.39 mmol, 1.00 eq) was added dropwise. After 6 h stirring, HMPA (3 mL) and a solution of the crude bromide (ca. 1.0 g) were added and the mixture was stirred at –78 °C for 2 h. The reaction was quenched by the addition of 1 M HCl (20 mL) and extracted with Et_2O (100 mL, three times). The combined organic layers were dried with $MgSO_4$ and the solvent was evaporated under reduce pressure. The residue was subjected to column chromatography (pentane/Et2O, 2:1) to yield the alcohol **S3** (940 mg, 4.22 mmol, 64%) as a colourless oil. TLC (petroleum ether: Et_2O = 2:1): R_f = 0.25. GC (HP-5): *I* = 1694. ^{1}H-NMR (C_6D_6, 500 MHz): 5.23 (ddq, 1H, *J* = 8.4, 5.8, 1.4 Hz), 4.83 (m, 1H), 4.78 (m, 1H), 3.48 (t, 2H, *J* = 6.6 Hz), 2.12 (m, 6H), 1.98 (m, 2H), 1.68 (m, 3H,), 1.57 (m, 6H) ppm. ^{13}C-NMR (C_6D_6, 125 MHz): 146.46 (C_q), 135.32 (C_q), 131.22 (C_q), 124.91 (CH), 124.10 (CH, t, $^1J_{C,D}$ = 22.90 Hz), 111.50 (CH_2), 60.76 (CH_2), 40.11 (CH_2), 39.74 (CH_2), 36.33 (CH_2), 27.17 (CH_2), 26.58 (CH_2), 25.87 (CH_3), 17.77 (CH_3), 16.10 (CH_3) ppm.

Synthesis of (*E*)-12-iodo-(6-^{2}H)-2,6-dimethyl-10-methylenedodeca-2,6-diene (S4)

To a solution of imidazole (338 mg, 4.97 mmol, 1.20 eq) and PPh_3 (1.3 g, 4.97 mmol, 1.20 eq) in CH_2Cl_2 (20 mL) were added **I**$_2$ (1.26 g, 4.97 mmol, 1.20 eq) in one portion and then **S3** (940 mg, 4.14 mmol, 1.00 eq) dropwise. After stirring for 1 h at room temperature, the reaction mixture was quenched by the addition of sat. aqueous NH_4Cl (20 mL) and then extracted three times with Et_2O (100 mL). The combined organic layers were dried with $MgSO_4$ and concentrated under reduced pressure. The residue was taken up in pentane, the precipitate was filtered off, and pentane was removed in vacuo. The product was purified by column chromatography (petroleum ether) to yield the **S4** as a pink oil (1.30 g, 3.92 mmol, 94%). TLC (petroleum ether): R_f = 0.64. GC (HP-5): *I* = 1886. ^{1}H-NMR (C_6D_6, 500 MHz): 5.22 (dddd, 1H, *J* = 7.0, 5.7, 2.9, 1.5 Hz), 4.79 (br s, 1H), 4.63 (br s, 1H), 2.83 (t, 2H, *J* = 7.7 Hz), 2.30 (t, 2H, *J* = 7.7 Hz), 2.16 (m, 2H), 2.05 (m, 4H), 1.85 (ddd, 2H, *J* = 8.7, 6.6, 1.5 Hz), 1.68 (br s, 3H), 1.57 (br s, 3H), 1.54 (br s, 3H) ppm. ^{13}C-NMR (C_6D_6, 125 MHz): 147.92 (C_q), 135.40 (C_q), 131.25 (C_q), 124.89 (CH), 123.89 (CH, t, $^1J_{C,D}$ = 23.2 Hz), 111.38 (CH_2), 40.69 (CH_2), 40.09 (CH_2), 35.60 (CH_2), 27.18 (CH_2), 26.41 (CH_2), 25.87 (CH_3), 17.78 (CH_3), 16.10 (CH_3), 3.23 (CH_2) ppm.

Synthesis of (3*E*,11*E*)-(11-^{2}H)-4,12,16-trimethyl-8-methyleneheptadeca-3,11,15-trien-2-one (S6)

To a cooled solution of ethyl acetoacetate (1.02 g, 7.84 mmol, 2.00 eq) in THF (20 mL) was added NaH (310 mg, 7.84 mmol, 2.00 eq, 60% in mineral oil) in small portions. The reaction mixture was allowed to reach room temperature and stirred for 1 h. The iodide **S4** was added dropwise and the reaction mixture was refluxed overnight, followed by cooling to room temperature. The cooled mixture was quenched by the addition of saturated aqueous NH_4Cl (20 mL) solution and then extracted with Et_2O (100 mL, 3 times). The combined organic layers were dried with $MgSO_4$ and concentrated under reduced pressure. The crude product **S5** was used for the next step without purification.

The solution of the crude β-keto ester **S5** (ca. 800 mg) in EtOH (15 mL) was mixed with an aqueous solution of KOH (655 mg, 11.7 mmol, 3.00 eq; 2 M) and the reaction mixture was refluxed for 3 h before cooling to room temperature. The reaction mixture was slowly acidified with 2 N HCl solution, leading to the development of CO_2. After CO_2 formation had stopped, the resulting suspension was

extracted with Et_2O (50 mL, three times). The combined layers were dried with $MgSO_4$ and concentrated under reduced pressure. The residue was subjected to column chromatography (petroleum ether /Et_2O, 10:1) to yield pure **S6** (346 mg, 1.32 mmol, 34%) as a colourless oil. TLC (petroleum ether /Et2O, 8:1): R_f = 0.46. GC (HP-5): *I* = 1911. ^{1}H-NMR (C_6D_6, 500 MHz): 5.24 (dddd, 1H, *J* = 7.0, 5.6, 2.9, 1.5 Hz), 4.85 (dd, 1H, *J* = 1.3, 0.6 Hz), 4.80 (dd, 1H, *J* = 1.3, 0.6 Hz), 2.18 (m, 4H), 2.06 (m, 2H), 1.92 (m, 4H), 1.68 (d, 3H, *J* = 1.4 Hz), 1.65 (m, 2H), 1.64 (s, 3H), 1.60 (s, 3H), 1.57 (d, 3H, *J* = 1.2 Hz) ppm. ^{13}C-NMR (C_6D_6, 125 MHz): 206.01 (C_q), 149.05 (C_q), 135.19 (C_q), 131.19 (C_q), 124.94 (CH), 124.25 (CH, t, $^1J_{C,D}$ = 23.0 Hz), 109.91 (CH_2), 42.65 (CH_2), 40.14 (CH_2), 36.21 (CH_2), 35.72 (CH_2), 29.39 (CH_2), 27.20 (CH_2), 26.65 (CH_2), 25.88 (CH_3), 21.96 (CH_3), 17.77 (CH_3), 16.13 (CH_3) ppm.

Synthesis of ethyl (2*E*,10*E*)-(10-^{2}H)-3,11,15-trimethyl-7-methylenehexadeca-2,10,14-trienoate (S7)

NaH (105 mg, 2.64 mmol, 2.00 eq, 60% in mineral oil) dissolved in dry THF (5 mL) was cooled to 0 °C. Triethyl phosphonoacetate (648 mg, 2.89 mmol, 2.20 eq) was added. After stirring the reaction mixture for 1 h at 0 °C, the reaction was cooled to –78 °C and the methyl ketone **S6** (346 mg, 1.32 mmol, 1.00 eq) was added. The reaction mixture was allowed to warm to room temperature and stirred overnight. Water (20 mL) was added to quench the reaction. The aqueous phase was extracted with Et_2O (50 mL, 3 times), and the combined layers were dried with $MgSO_4$ and concentrated under reduced pressure. Purification by column chromatography (petroleum ether/EtOAc, 15:1) yielded pure (*E*)-**S7** and (*Z*)-**S7** as colourless oils.

(*E*)-**S7**: 145 mg (0.44 mmol, 33%) TLC (petroleum ether: Et_2O = 8:1): R_f = 0.74. GC (HP-5): *I* = 2317. ^{1}H-NMR (C_6D_6, 500 MHz): 5.82 (m, 1H), 5.24 (m, 1H), 4.84 (br s, 1H), 4.77 (br s, 1H), 4.06 (q, 2H, *J* = 7.1 Hz), 2.19 (d, 3H, *J* = 1.5 Hz), 2.17 (m, 2H), 2.09 (m, 2H), 2.01 (m, 2H), 1.84 (m, 4H), 1.68 (br s, 3H), 1.60 (s, 3H), 1.57 (br s, 3H), 1.42 (tt, 2H, *J* = 8.9, 6.9 Hz), 1.02 (t, 3H, *J* = 7.2 Hz) ppm. ^{13}C-NMR (C_6D_6, 125 MHz): 166.47 (C_q), 159.56 (C_q), 148.91 (C_q), 135.21 (C_q), 131.21 (C_q), 124.91 (CH), 124.23 (CH, t, $^1J_{C,D}$ = 22.8 Hz), 116.36 (CH), 109.82 (CH_2), 59.40 (CH_2), 40.54 (CH_2), 40.14 (CH_2), 36.31 (CH_2), 35.82 (CH_2), 27.19 (CH_2), 26.65 (CH_2), 25.88 (CH_3), 25.69 (CH_2), 18.73 (CH_3), 17.77 (CH_3), 16.12 (CH_3), 14.46 (CH_3) ppm.

(*Z*)-**S7**: 56 mg (0.17 mmol, 13%) TLC (petroleum ether: Et_2O = 8:1): R_f = 0.68. GC (HP-5): *I* = 2258. ^{1}H-NMR (C_6D_6, 500 MHz): 5.75 (m, 1H), 5.24 (m, 1H), 4.88 (m, 2H), 4.03 (q, 2H, *J* = 7.1Hz), 2.73 (m, 2H), 2.19 (m, 4H), 2.11 (m, 4H), 1.68 (br s, 3H), 1.64 (m, 2H), 1.60 (s, 3H), 1.56 (br s, 3H), 1.53 (d, 3H, *J* = 1.5 Hz), 1.01 (t, 3H, *J* = 7.2 Hz) ppm. ^{13}C-NMR (C_6D_6, 125 MHz): 166.02 (C_q), 160.08 (C_q), 149.40 (C_q), 135.04 (C_q), 131.12 (C_q), 125.00 (CH), 124.37 (CH, t, $^1J_{C,D}$ = 23.0 Hz), 116.91 (CH), 109.60 (CH_2), 59.37 (CH_2), 40.15 (CH_2), 36.58 (CH_2), 36.42 (CH_2), 33.40 (CH_2), 27.22 (CH_2), 26.82 (CH_2), 26.70 (CH_3), 25.88 (CH_2), 24.88 (CH_3), 17.77 (CH_3), 16.12 (CH_3), 14.43 (CH_3) ppm.

Synthesis of (2*E*,10*E*)-(10-^{2}H)-3,11,15-trimethyl-7-methylenehexadeca-2,10,14-trien-1-ol (S8)

To a cooled (0 °C) solution of the ester (*E*)-**S7** (145 mg, 0.44 mmol, 1.00 eq) in CH_2Cl_2 (2 mL) was added DIBAL-H (603 mg, 1.06 mmol, 25 wt-% in toluene, 2.40 eq) and the reaction mixture was stirred for 1 h at room temperature. The mixture was cooled to 0 °C and a saturated solution of Na-K-tartrate (10 mL) was added. The resulting slurry was stirred for 2 h to dissolve the precipitate and the aqueous phase was extracted with Et_2O (30 mL, 3 times). The organic layers were dried with $MgSO_4$ and concentrated under reduced pressure. The residue was purified by column chromatography (petroleum ether:Et_2O = 3:1) to yield the alcohol **S8** (123 mg, 0.42 mmol, 96%) as a colourless oil. TLC (petroleum ether:Et_2O = 2:1): R_f = 0.26. GC (HP-5): *I* = 2192. ^{1}H-NMR (C_6D_6, 500 MHz): 5.38 (tp, 1H, *J* = 6.7, 1.3 Hz), 5.24 (ddq, 1H, *J* = 8.6, 5.4, 1.4 Hz), 4.87 (m, 2H), 3.97 (m, 2H), 2.19 (m, 4H), 2.10 (m, 4H), 1.98 (m, 2H), 1.91 (m, 2H), 1.68 (d, 3H, *J* = 1.4 Hz), 1.60 (s, 3H), 1.56 (br s, 3H), 1.53 (m, 2H), 1.46 (br s, 3H) ppm. ^{13}C-NMR (C_6D_6, 125 MHz): 149.47 (C_q), 138.19 (C_q), 135.18 (C_q), 131.19 (C_q), 125.05 (CH), 124.95 (CH), 109.59 (CH_2), 59.38 (CH_2), 40.14 (CH_2), 39.48 (CH_2), 36.51 (CH_2), 36.11 (CH_2), 27.22 (CH_2), 26.76 (CH_2), 26.24 (CH_2), 25.86 (CH_3), 17.76 (CH_3), 16.13 (CH_3), 16.10 (CH_3) ppm.

Synthesis of *iso*-(10-^{2}H)GGPP I (S10)

To a cooled (0 °C) solution of **S8** (123 mg, 0.42 mmol, 1.00 eq) in Et_2O (2 mL) PBr_3 (15 µL, 0.17 mmol, 0.40 eq) was added dropwise. The mixture was stirred for 1 h at 0 °C and was transferred directly to an ice/water mixture. The aqueous layer was extracted with Et_2O (20 mL, three times), the organic layers were dried with $MgSO_4$ and concentrated under reduced pressure. The bromide **S9** (ca. 500 mg) was directly used for the next reaction without purification.

To a solution of tris(tetra-n-butylammonium)hydrogen diphosphate (450 mg, 0.5 mmol, 1.20 eq) in acetonitrile (3 mL) a solution of the allyl bromide **S9** was added and the mixture was stirred at room temperature overnight. Acetonitrile was removed under reduced pressure. The residue was dissolved in aqueous NH_4HCO_3 solution (1 mL, 0.25 M) and loaded onto a DOWEX 50WX8 ion-exchange column (NH_4^+ form, pH 7.0). The column was flushed slowly with 1.5 column volumes of NH_4HCO_3 buffer (25 mM, 5% iPrOH) and the eluate was lyophilised to yield the diphosphate **S10** as a colourless hygroscopic powder (200 mg, 0.40 mmol, 95%). ^{1}H-NMR (D_2O, 500 MHz): 5.50 (m, 1H), 5.13 (m, 2H), 4.51 (m, 2H), 4.39 (t, 1H, J = 7.7 Hz), 2.06 (m, 14H), 1.75 (s, 3H), 1.69 (s, 3H), 1.61 (m, 6H) ppm. ^{13}C-NMR (D_2O, 125 MHz): 142.26 (C_q), 134.96 (C_q), 134.61 (C_q), 130.57 (C_q), 124.60 (CH), 120.34 (CH, d, $^3J_{C,P}$ = 8.7 Hz), 109.00 (CH_2), 62.51 (CH_2, d, $^2J_{C,P}$ = 4.5 Hz), 39.58 (CH_2), 39.20 (CH_2), 36.13 (CH_2), 35.68 (CH_2), 26.66 (CH_2), 26.15 (CH_2), 25.78 (CH_2), 25.42 (CH_3), 17.44 (CH_3), 15.88 (CH_3), 15.80 (CH_3) ppm. ^{31}P-NMR (D_2O, 203 MHz): –9.87 (d, $^2J_{P,P}$ = 19.6 Hz), –9.93 (d, $^2J_{P,P}$ = 19.6 Hz) ppm.

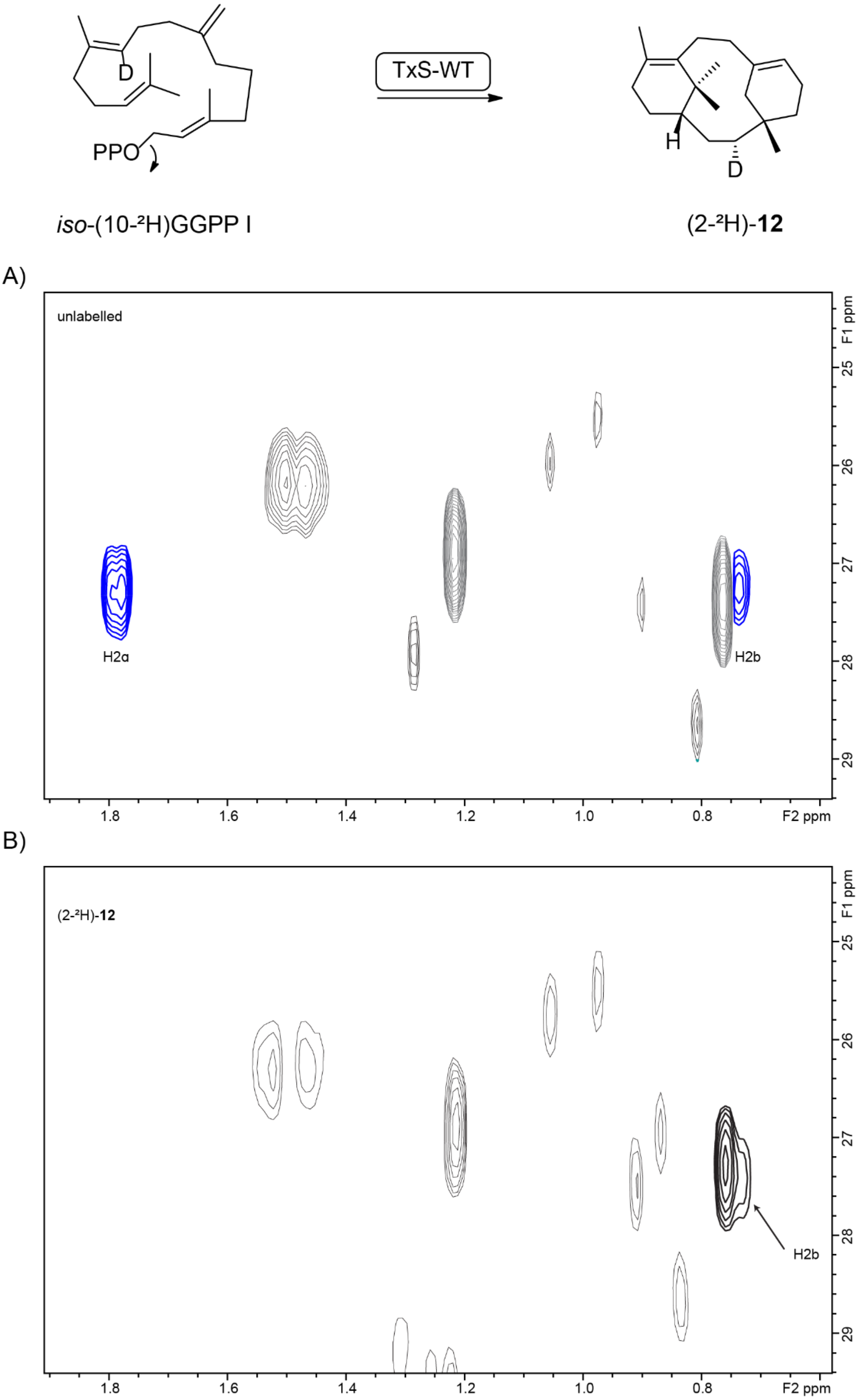

Figure S45. Proton shifts in the biosynthesis of taxaxenene (**12**). Partial HSQC spectra showing the signals for C2 of A) unlabelled **12** and B) (2-^{2}H)-**12** obtained from an incubation of (10-^{2}H)-*iso*-GGPP I with TxS.

Table S13. Results of DFT calculations for biosynthesis of **12 – 15** from *iso*-GGPP I by TxS and TxS-V610T (Scheme 5 of main text).

**Structure**	**Gibbs energy (298.15K) in Hartree**	**energy relative to *iso*-A in kcal/mol**	**reaction barrier in kcal/mol**	**Gibbs free energy in kcal/mol**
**A***	–781.656565	0.00		
**A*-B1*-TS**	–781.656087	0.30	0.30	
**B1***	–781.682987	–16.58		–16.58
**B2***	–781.678549	–13.80		
**B2*-B3*-TS**	–781.677298	–13.01	0.79	
**B3***	–781.704010	–29.77		–15.98
**B3***	–781.704011	–29.77		
**B3*-C1*-TS**	–781.705822	–30.91	–1.14	
**C1***	–781.714962	–36.64		–6.87
**C1***	–781.714959	–36.64		
**C1*-E*-TS**	–781.706368	–31.25	5.39	
**E***	–781.714888	–36.60		0.04
**E***	–781.714890	–36.60		
**E*-F*-TS**	–781.711380	–34.40	2.20	
**F***	–781.734640	–48.99		–12.39
**C2***	–781.715173	–36.78		
**C2*-D1*-TS**	–781.681589	–15.70	21.07	
**D1***	–781.718194	–38.67		–1.90
**D2***	–781.696824	–25.26		
**D2*-E*-TS**	–781.698028	–26.02	–0.76	
**E***	–781.714891	–36.60		–11.34

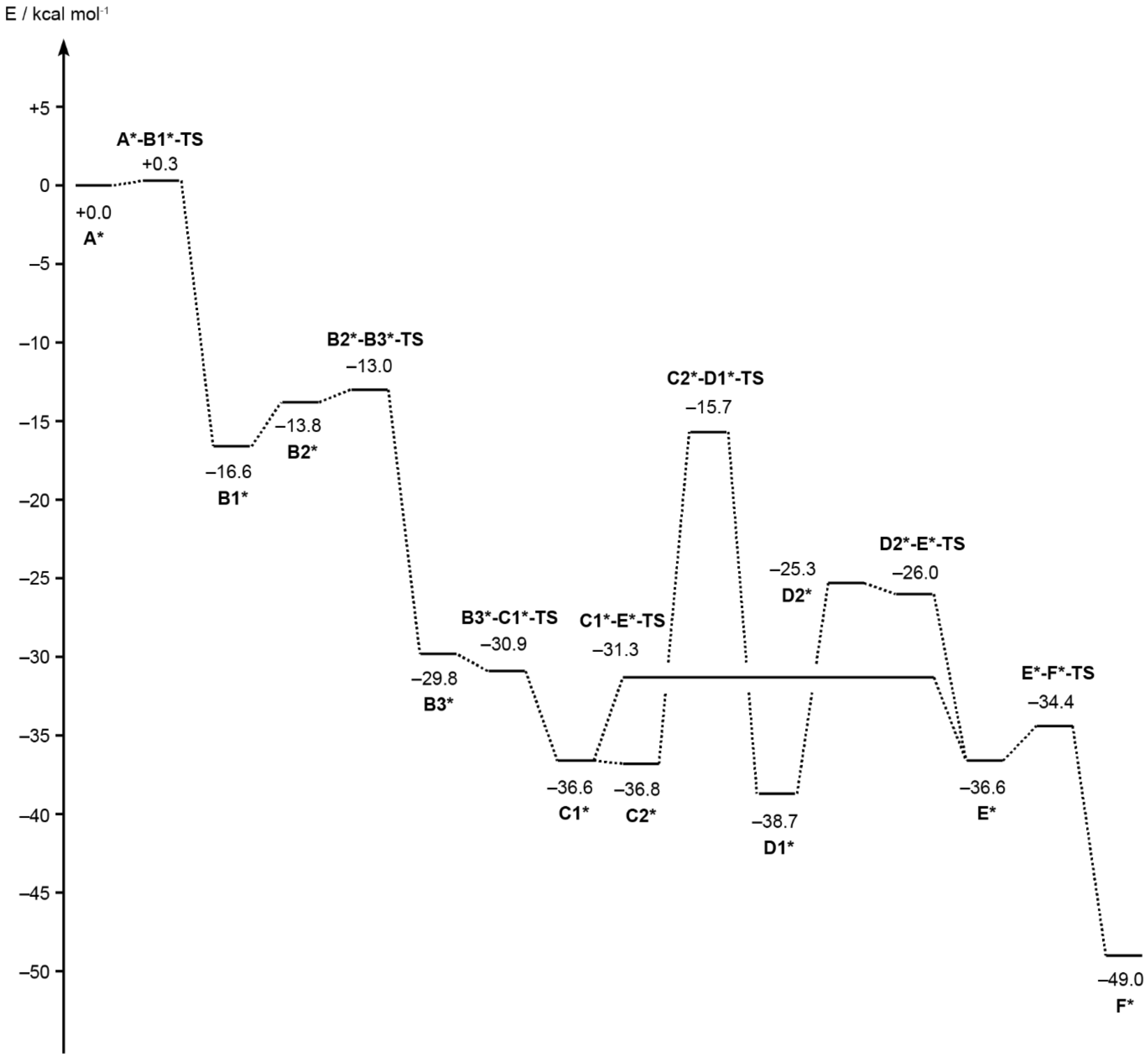

Figure S46. Computed energy profile for the transformations from *iso*-GGPP I to **12** to **16** (Scheme 5 of main text, mPW1PW91/6-311+G(d,p)//B97D3/6-31g(d,p), 298 K).

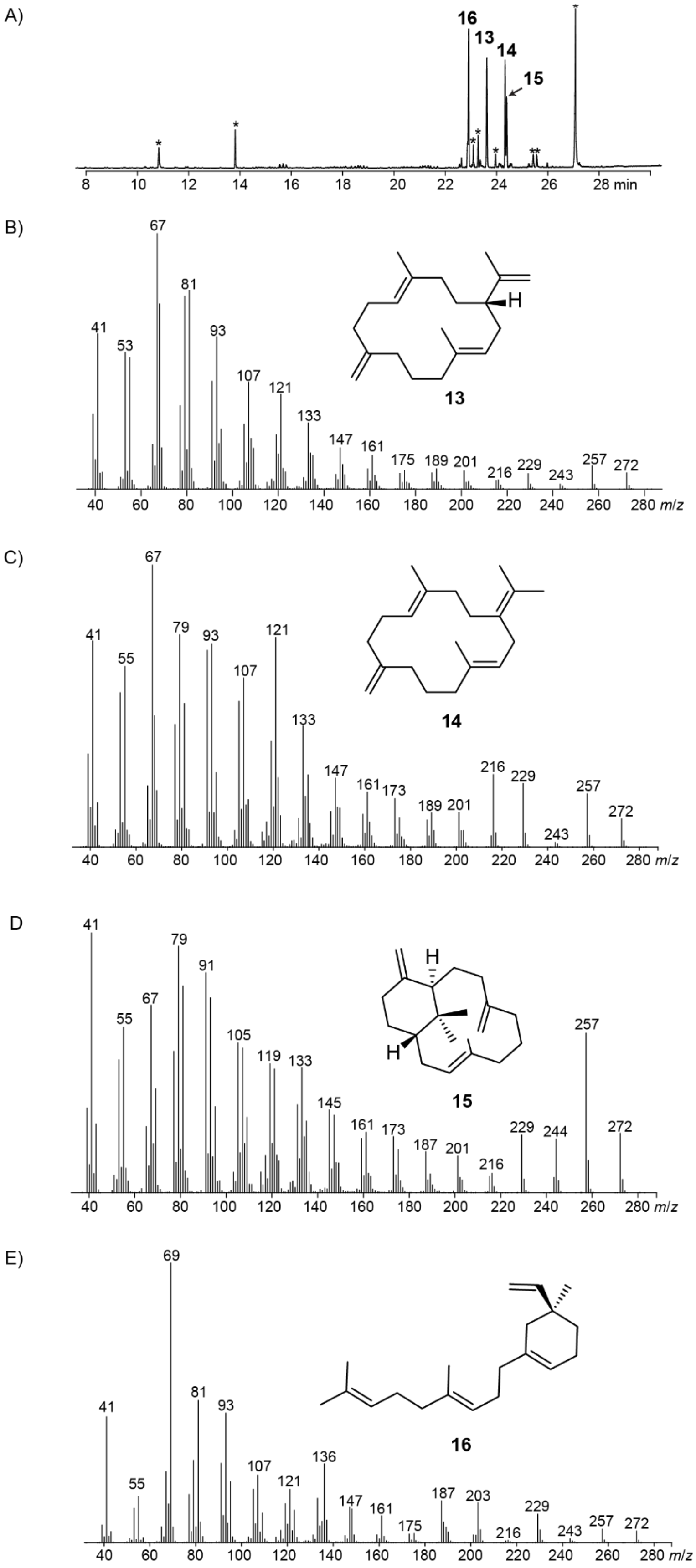

Figure S47. GC/MS analysis of the products obtained with TxS-V610T from *iso*-GGPP I. A) Total ion chromatogram of a crude extract from an enzyme incubation of *iso*-GGPP I with TxS-V610T. EI mass spectra of B) isocembrene A2 (**13**), C) isocembrene C (**14**), D) verticilla-3,8(19),12(18)-triene (**15**), and E) taxasimplene (**16**).

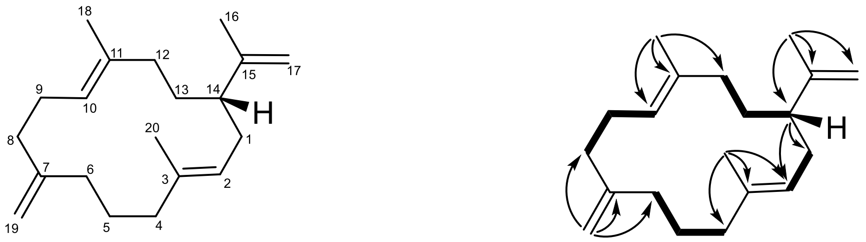

13

Figure S48. Structure elucidation of isocembrene A2 (**13**). Bold: ^{1}H,^{1}H-COSY, and single headed arrows: key HMBC correlations. Carbon numbering follows GGPP numbering to indicate the origin of each carbon.

Table S14. NMR data of isocembrene A2 (**13**) in C_6D_6 recorded at 298 K.

C[a]	type	^{13}C[b]	^{1}H[b]
1	CH_2	32.47	2.15 (m)<br>2.04 (m)
2	CH	125.39	5.22 (m)
3	C_q	135.09	–
4	CH_2	39.51	2.05 (m, 2H)
5	CH_2	24.64	1.56 (m, 2H)
6	CH_2	35.52	2.00 (m)<br>1.96 (m)
7	C_q	152.41	–
8	CH_2	37.32	2.14 (m)<br>2.08 (m)
9	CH_2	30.23	2.21 (m, 2H)
10	CH	125.19	5.26 (m)
11	C_q	135.85	–
12	CH_2	35.39	2.02 (m)<br>1.82 (m)
13	CH_2	30.22	1.62 (m)<br>1.49 (m)
14	CH	47.05	2.09 (m)
15	C_q	148.83	–
16	CH_2	110.09	4.85 (m, 2H)
17	CH_3	20.08	1.64 (m)
18	CH_3	17.63	1.58 (s)
19	CH_2	109.23	4.88 (m, 2H)
20	CH_3	15.68	1.53 (s)

[a] Carbon numbering as shown in Figure S48. [b] Chemical shifts δ in ppm, multiplicity: s = singlet, m = multiplet, coupling constants *J* are given in Hertz.

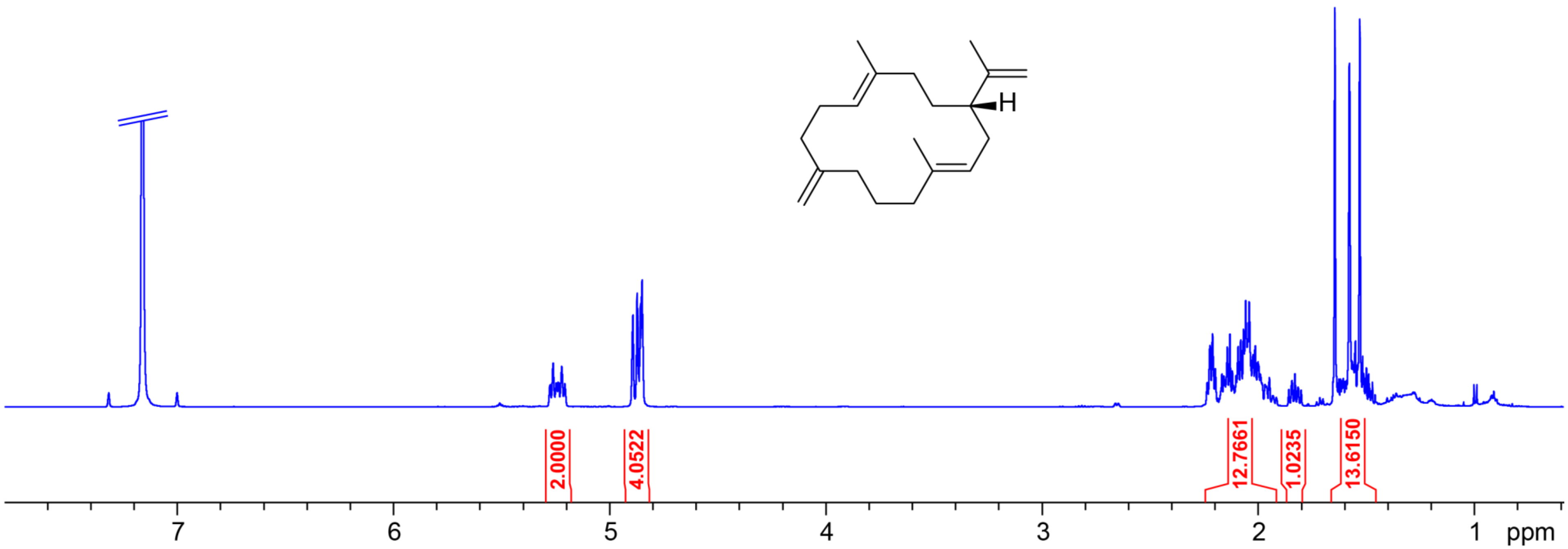

Figure S49. ^{1}H-NMR spectrum of **13** (700 MHz, C_6D_6).

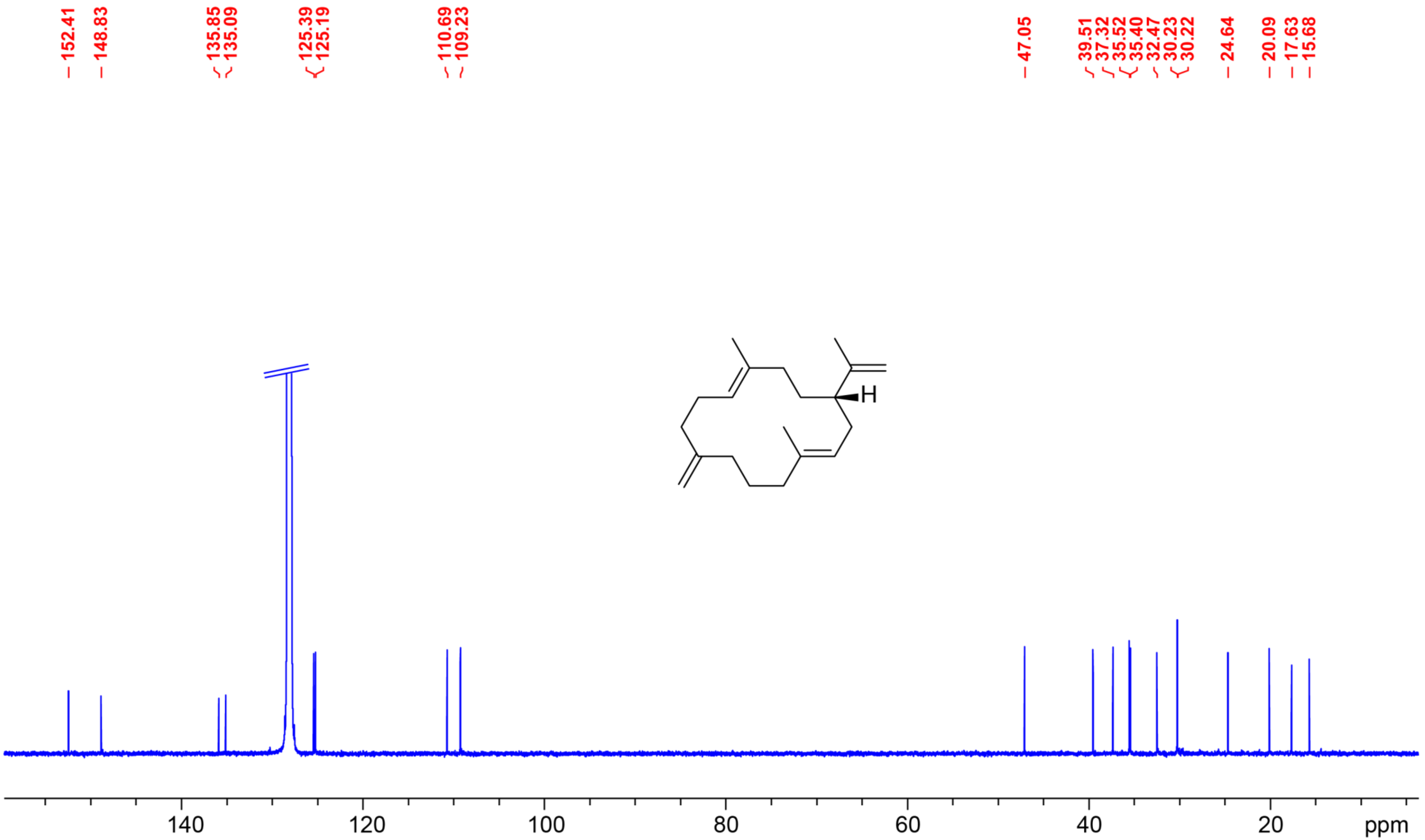

Figure S50. ^{13}C-NMR spectrum of **13** (176 MHz, C_6D_6).

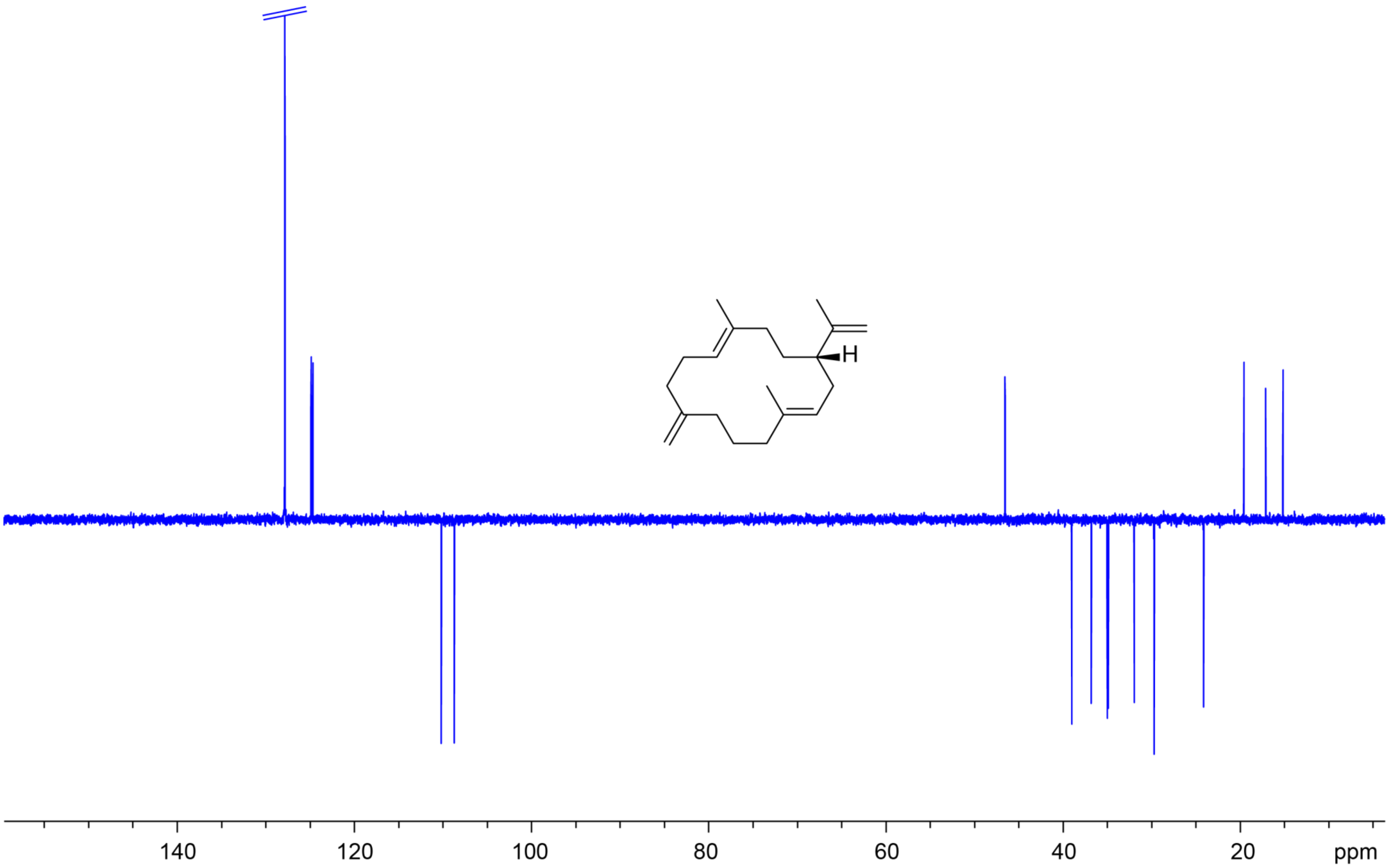

Figure S51. ^{13}C-DEPT135 spectrum of **13** (176 MHz, C_6D_6).

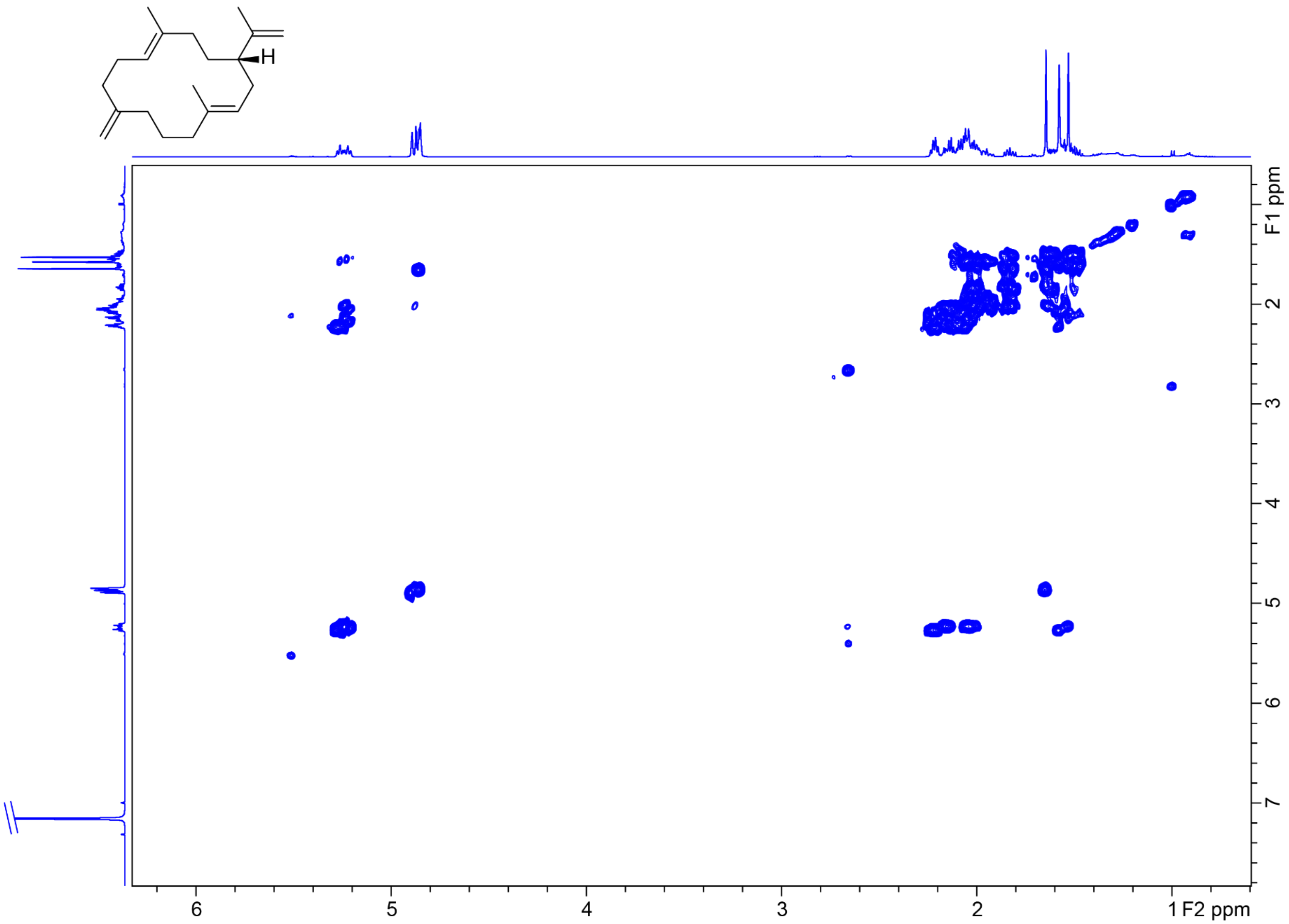

Figure S52. ^{1}H-^{1}H-COSY spectrum (C_6D_6) of **13**.

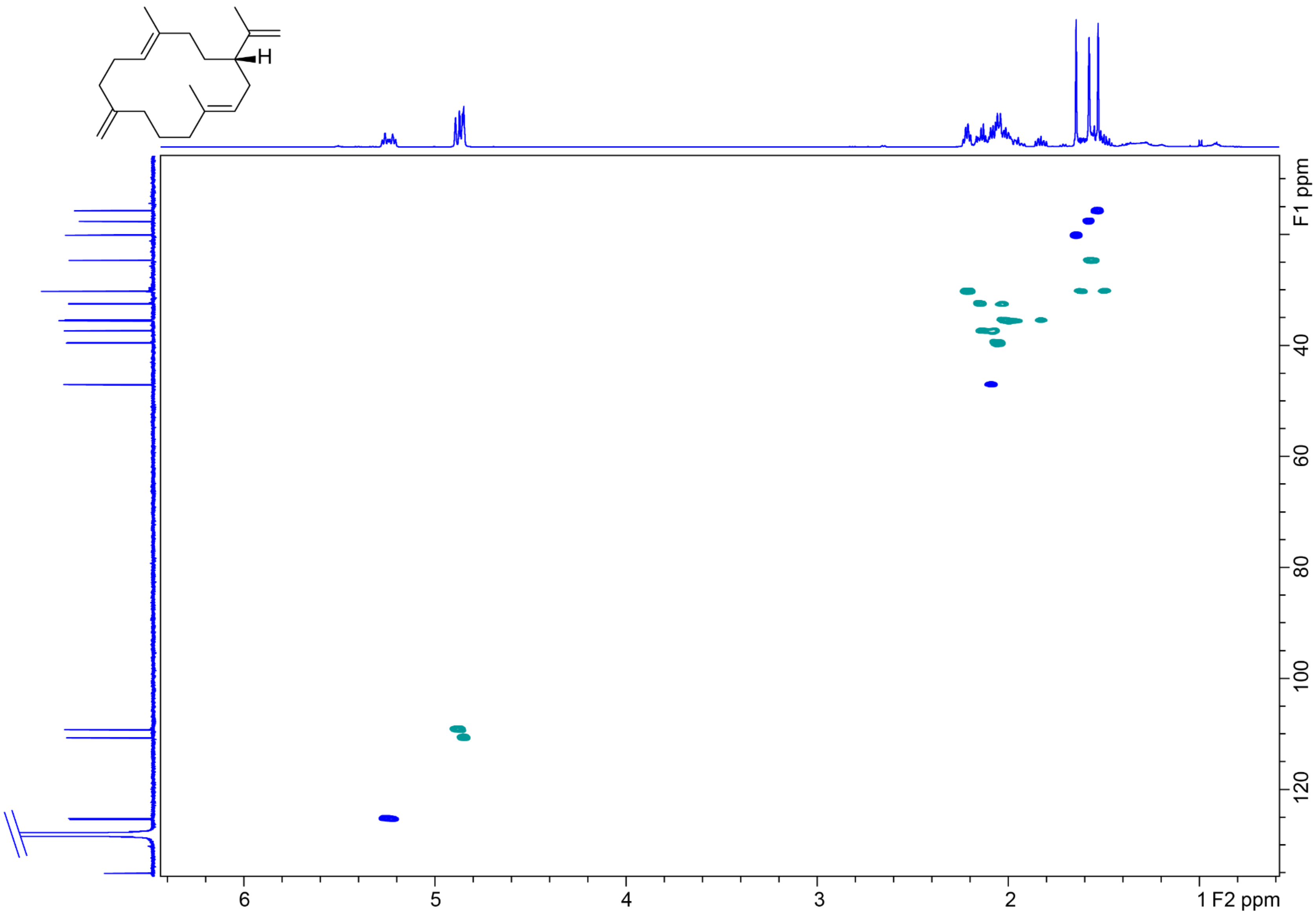

Figure S53. HSQC spectrum (C_6D_6) of **13**.

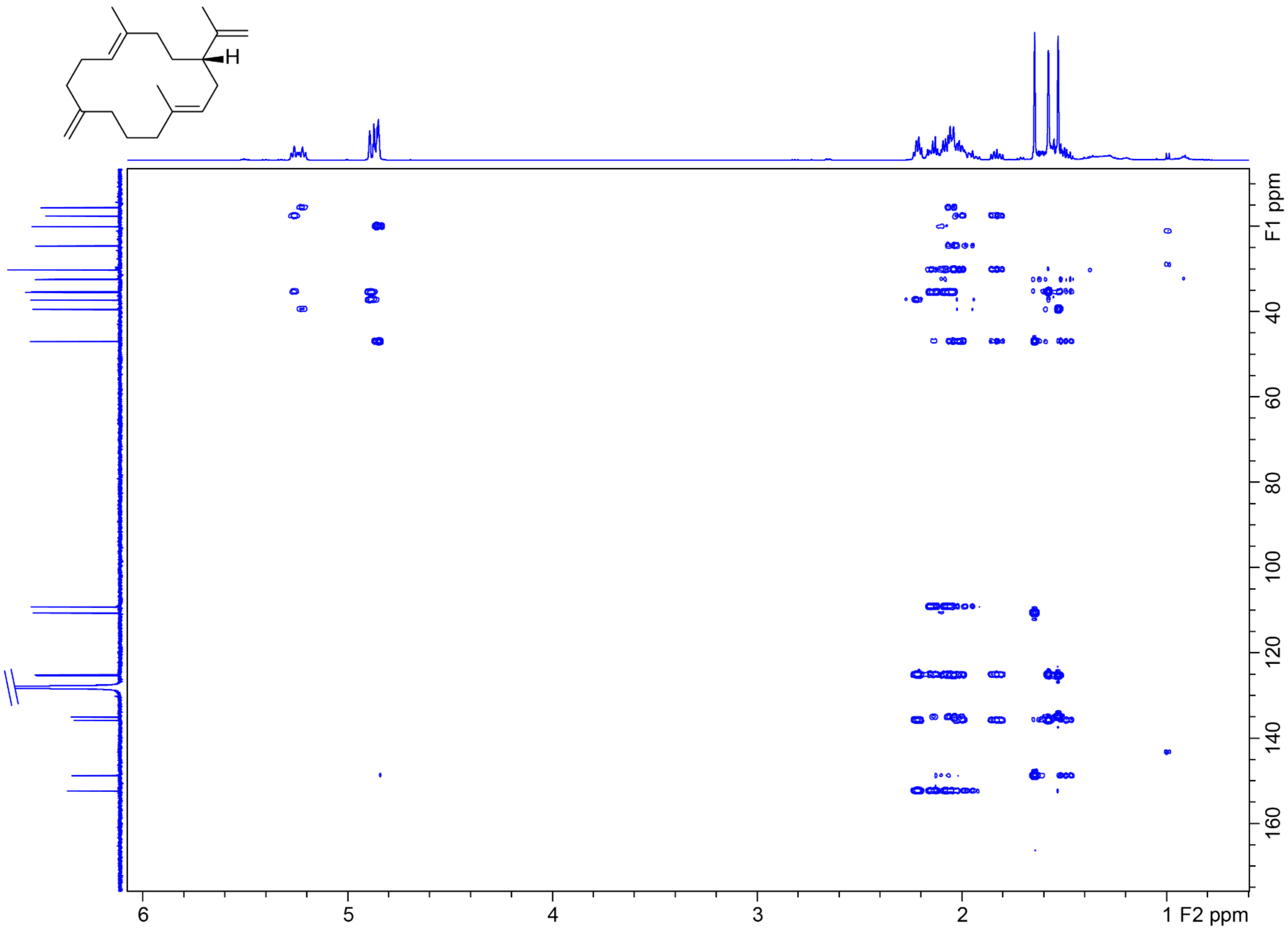

Figure S54. HMBC spectrum (C_6D_6) of **13**.

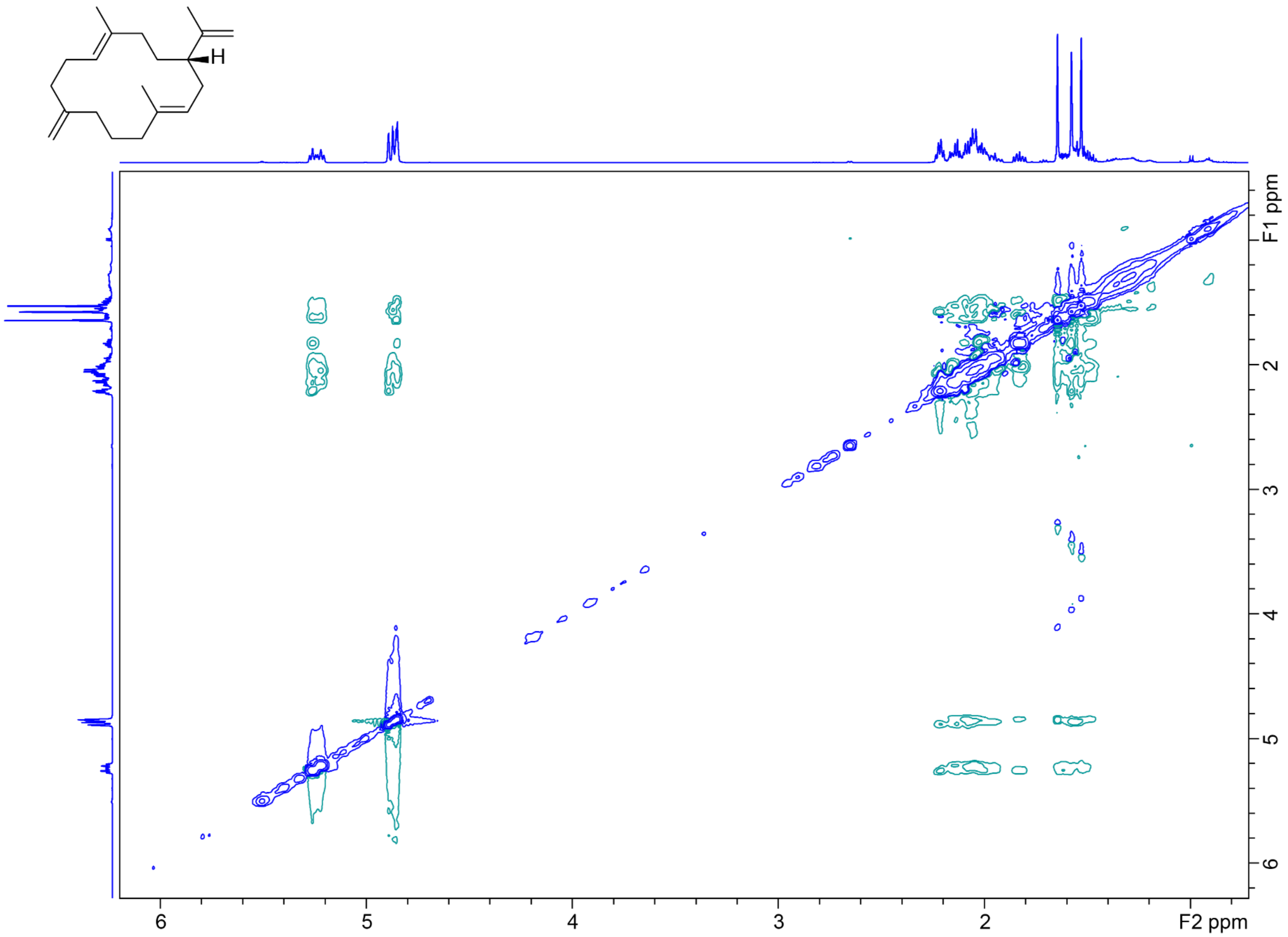

Figure S55. NOESY spectrum (C_6D_6) of **13**.

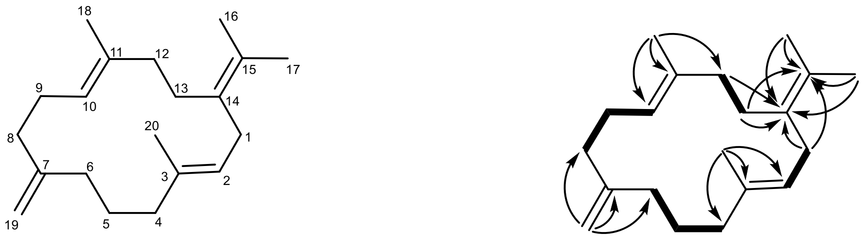

14

Figure S56. Structure elucidation of isocembrene C (**14**). Bold: $^{1}H,^{1}H$-COSY, single headed arrows: key HMBC. Carbon numbering follows GGPP numbering to indicate the origin of each carbon.

Table S15. NMR data of isocembrene C (**14**) in C_6D_6 recorded at 298 K.

C[a]	type	^{13}C[b]	^{1}H[b]
1	CH_2	30.48	2.95 (d, 2H, *J* = 7.2 Hz)
2	CH	125.43	5.16 (m)
3	C_q	134.43	–
4	CH_2	39.30	2.05 (m, 2H)
5	CH_2	24.65	1.56 (m, 2H)
6	CH_2	35.95	2.05 (m, 2H)
7	C_q	152.14	–
8	CH_2	36.64	2.11 (m, 2H)
9	CH_2	30.02	2.21 (m, 2H)
10	CH	125.04	5.26 (m)
11	C_q	135.50	–
12	CH_2	37.91	2.02 (m, 2H)
13	CH_2	30.15	2.29 (m, 2H)
14	C_q	131.29	–
15	C_q	124.50	–
16	CH_3	20.83	1.70 (s)
17	CH_3	20.52	1.65 (s)
18	CH_3	15.94	1.58 (s)
19	CH_2	109.69	4.87 (m)
20	CH_3	15.58	1.56 (m)

[a] Carbon numbering as shown in Figure S56. [b] Chemical shifts δ in ppm, multiplicity: s = singlet, d = doublet, m = multiplet, coupling constants *J* are given in Hertz.

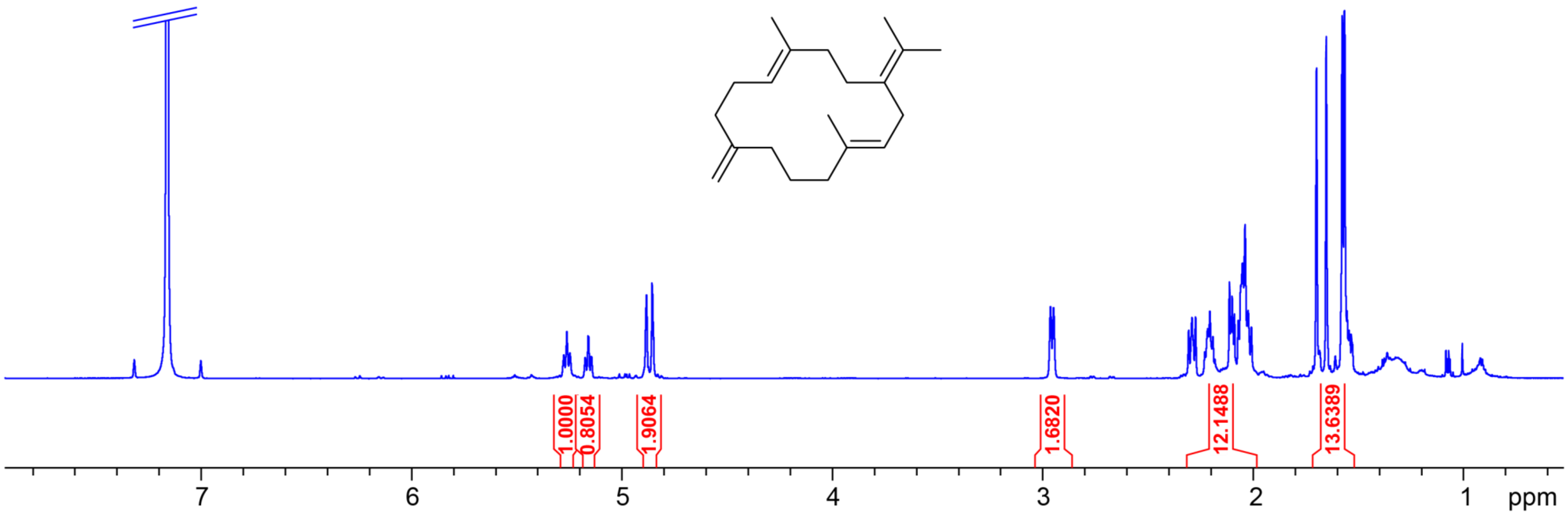

Figure S57. ^{1}H-NMR spectrum of **14** (700 MHz, C_6D_6).

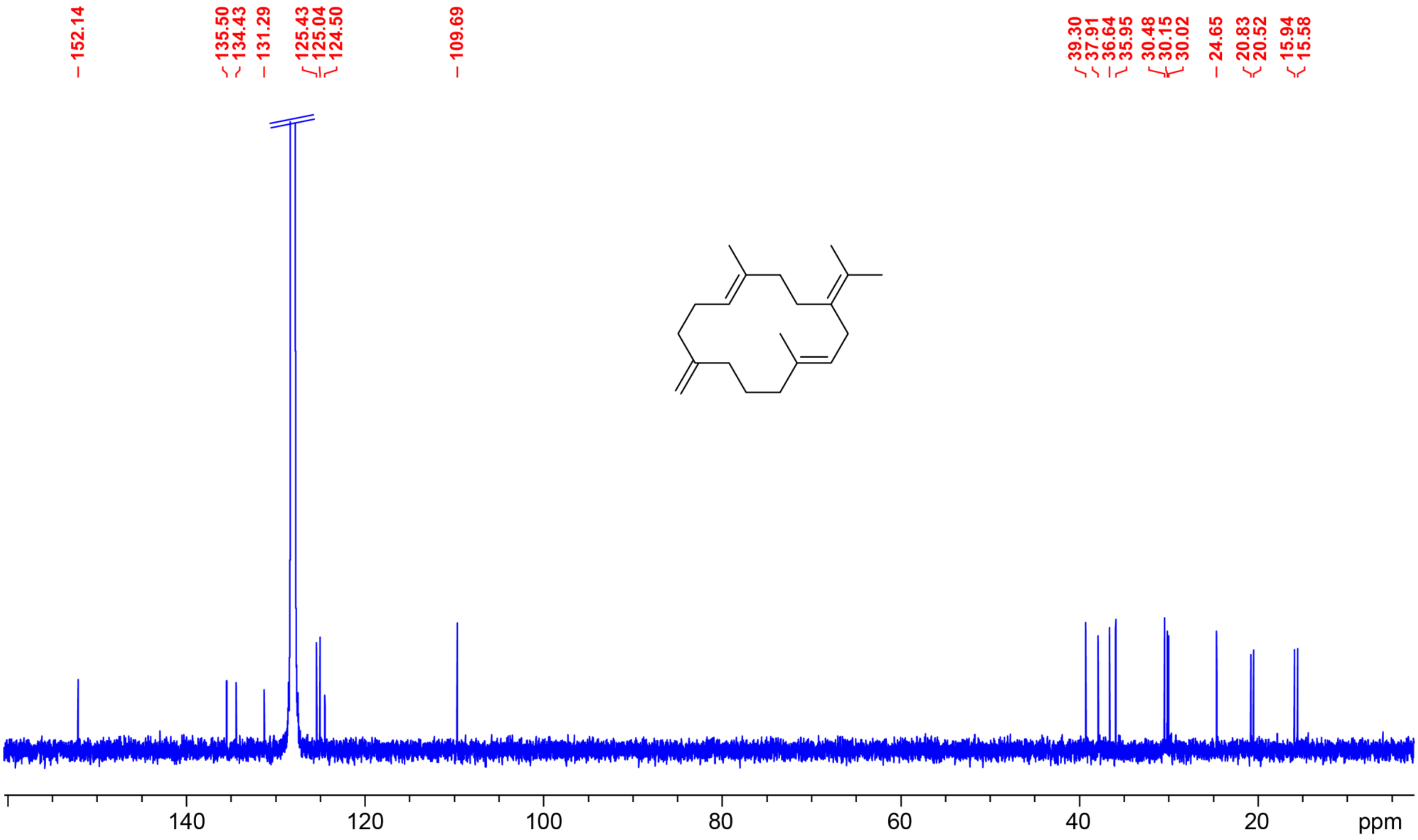

Figure S58. ^{13}C-NMR spectrum of **14** (176 MHz, C_6D_6).

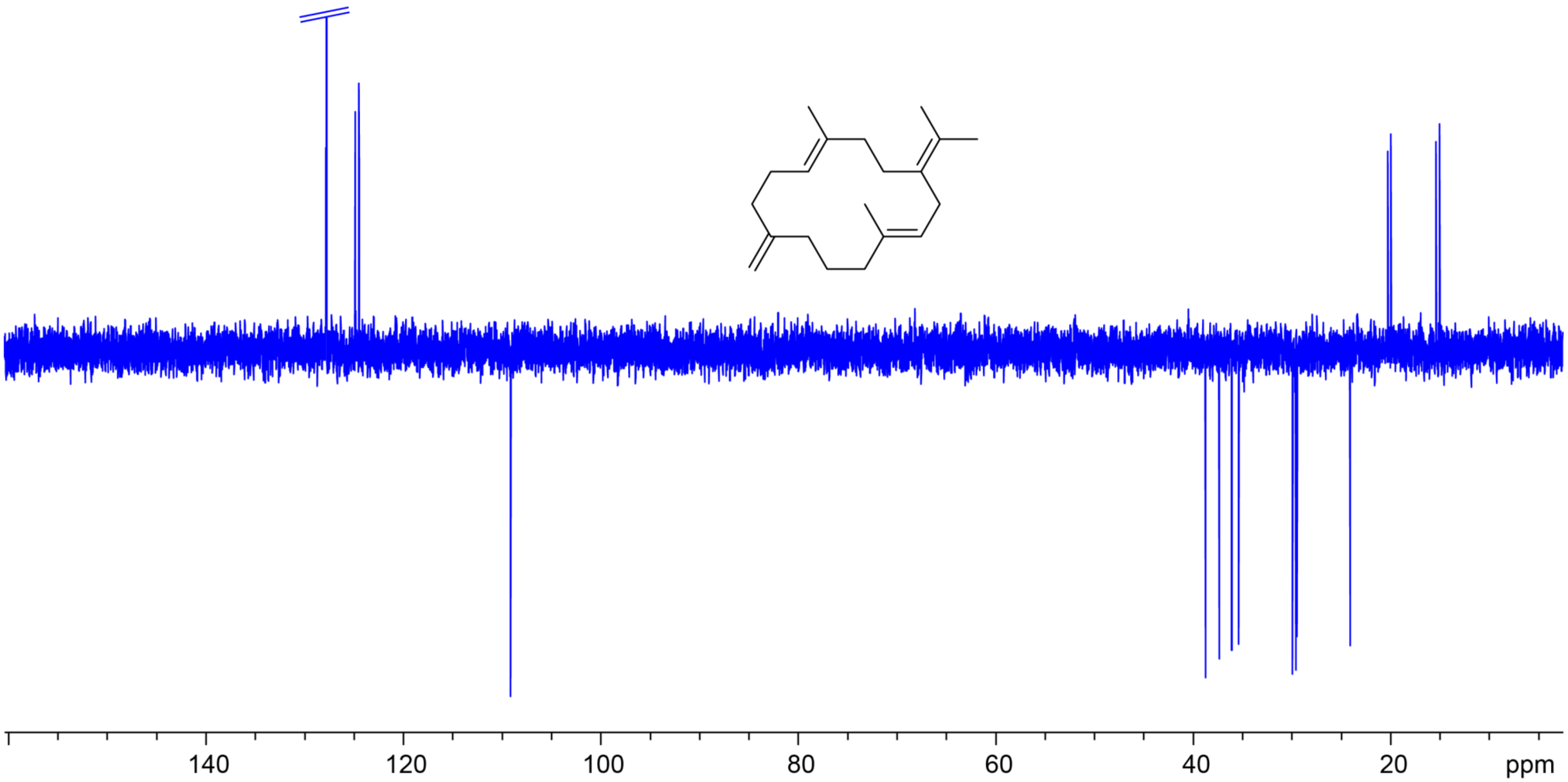

Figure S59. ^{13}C-DEPT135 spectrum of **14** (176 MHz, C_6D_6).

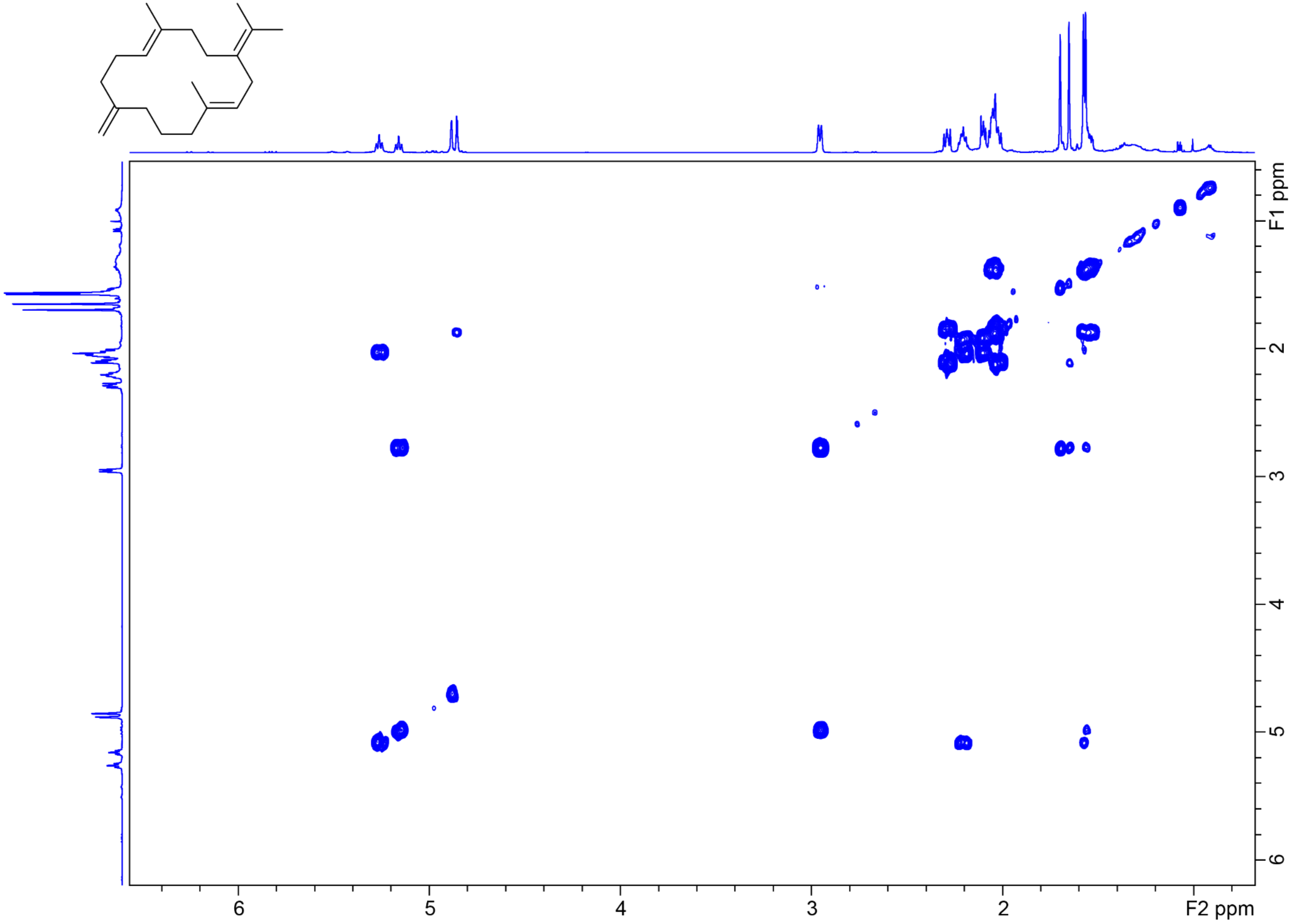

Figure S60. ^{1}H-^{1}H-COSY spectrum (C_6D_6) of **14**.

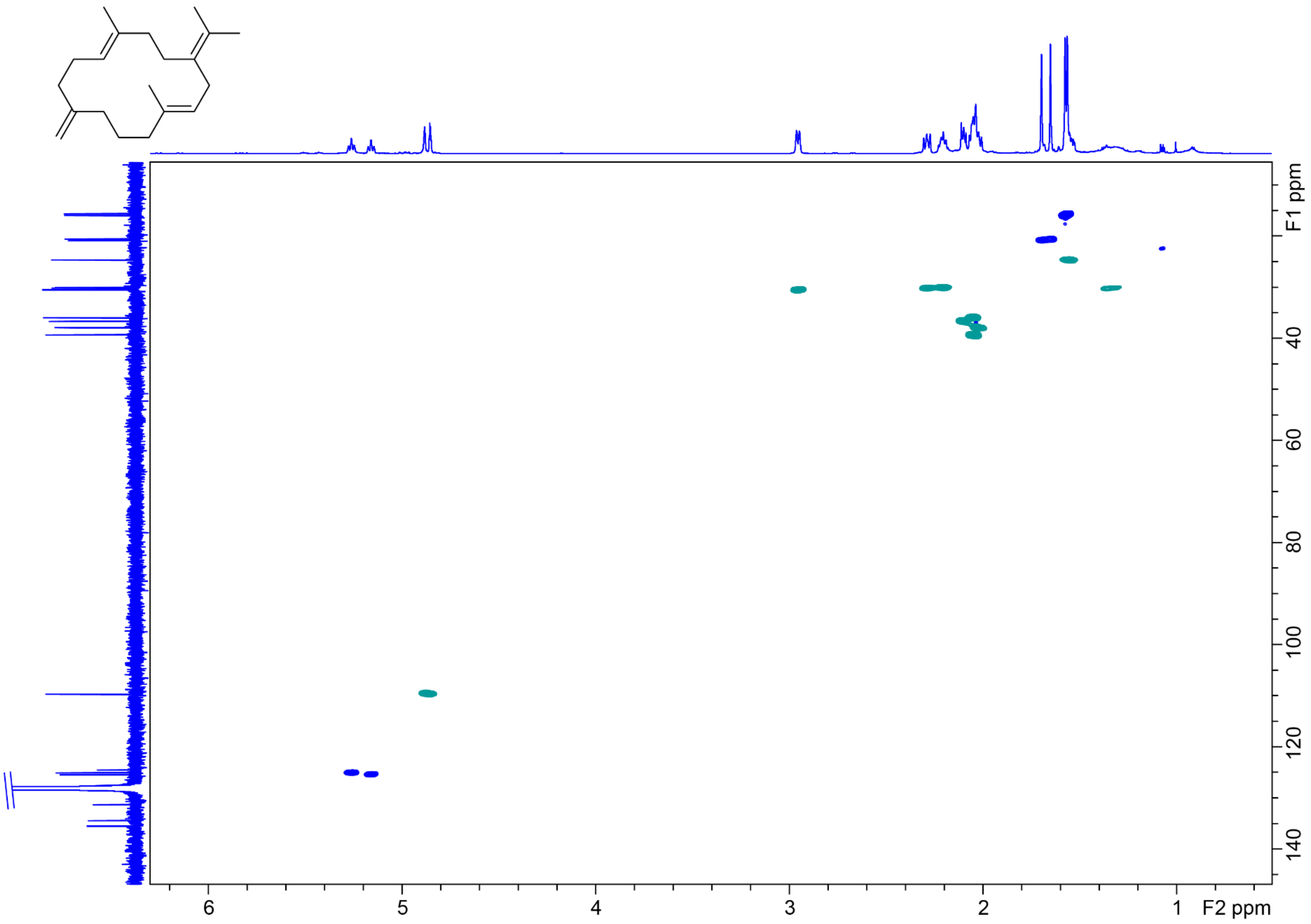

Figure S61. HSQC spectrum (C_6D_6) of **14**.

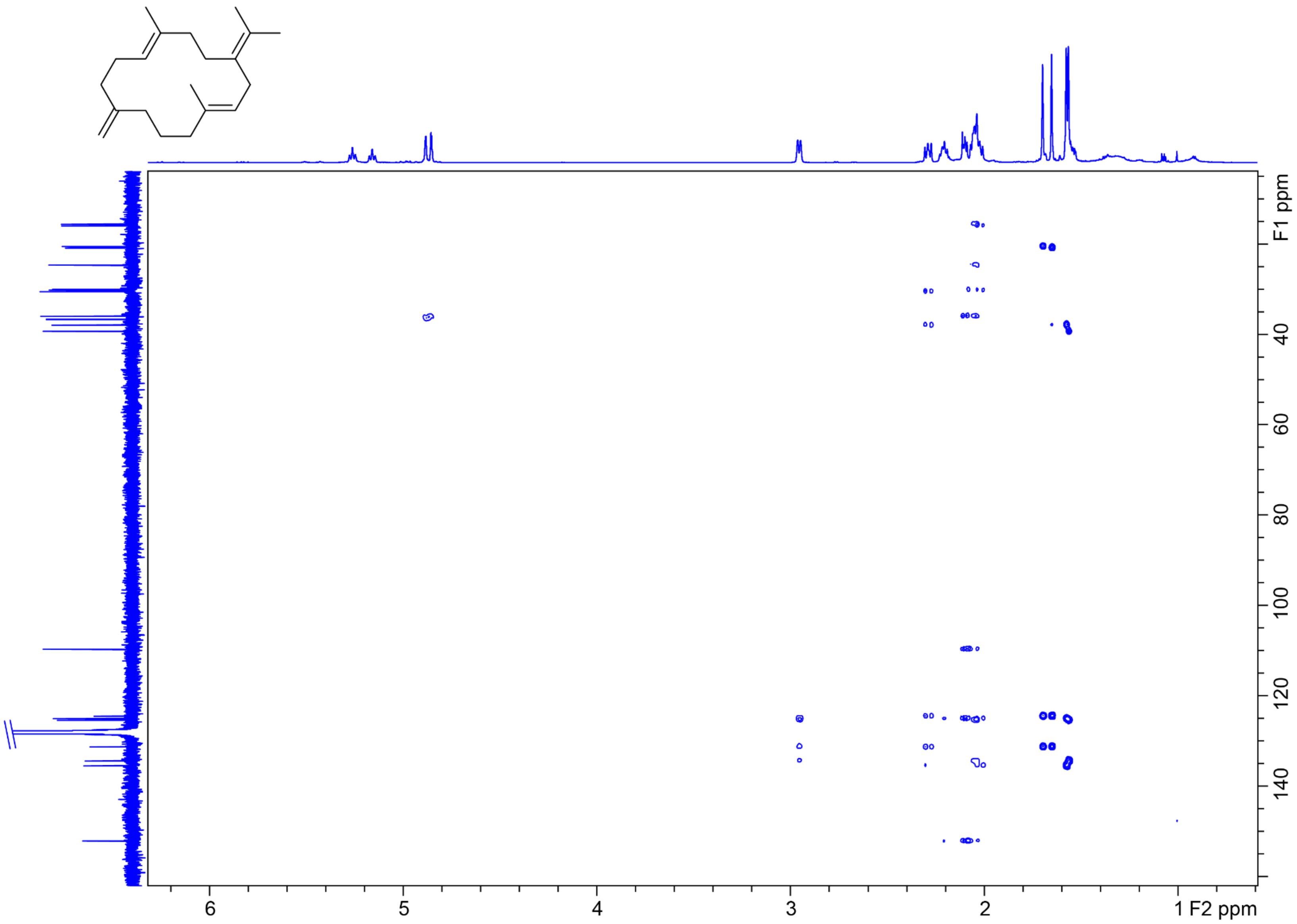

Figure S62. HMBC spectrum (C_6D_6) of **14**.

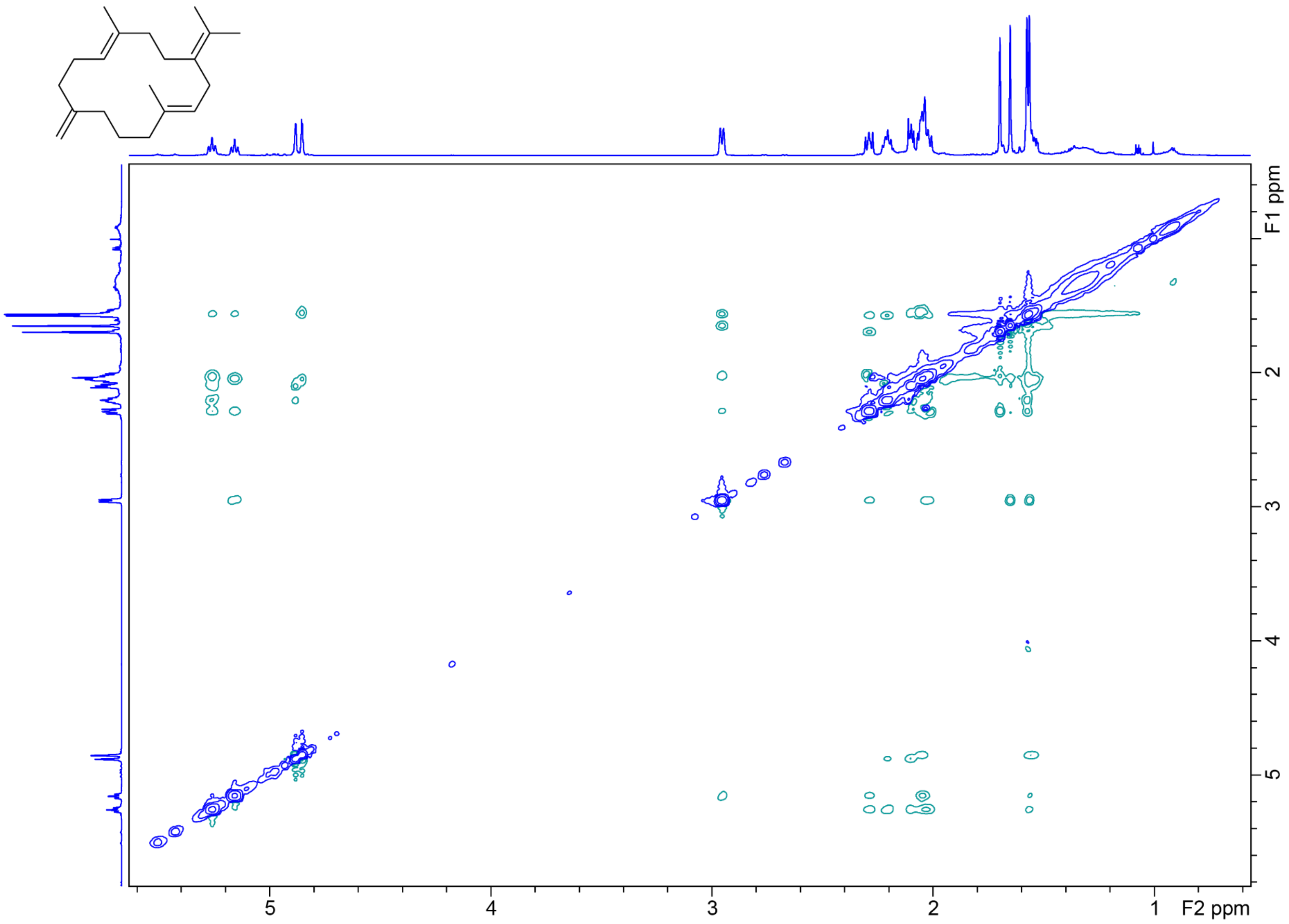

Figure S63. NOESY spectrum (C_6D_6) of **14**.

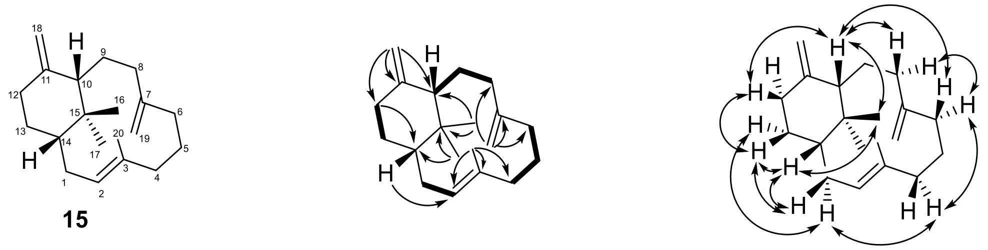

Figure S64. Structure elucidation of verticilla-3,8(19),12(18)-triene (**15**). Bold: ^{1}H,^{1}H-COSY, single headed arrows: key HMBC, and double headed arrows: NOESY correlations. Carbon numbering follows GGPP numbering to indicate the origin of each carbon.

Table S16. NMR data of verticilla-3,8(19),12(18)-triene (**15**) in C_6D_6 recorded at 298 K.

C[a]	type	^{13}C[b]	^{1}H[b]
1	CH_2	34.18	2.69 (m)<br>1.88 (m)
2	CH	128.75	5.76 (br d, J = 12.9)
3	C_q	131.73	–
4	CH_2	37.63	2.46 (m)<br>2.03 (m)
5	CH_2	21.82	1.79 (m)<br>1.67 (m)
6	CH_2	27.71	1.83 (m)<br>1.63 (m)
7	C_q	146.69	–
8	CH_2	35.11	2.42 (m)<br>2.03 (m)
9	CH_2	20.47	1.59 (m)<br>1.39 (m)
10	CH	43.03	2.44 (m)
11	C_q	148.98	–
12	CH_2	36.50	2.40 (m)<br>2.23 (m)
13	CH_2	31.52	2.05 (m)<br>1.50 (m)
14	CH	45.06	1.36 (m)
15	C_q	38.38	–
16	CH_3	26.39	0.93 (s)
17	CH_3	24.65	0.86 (s)
18	CH_2	106.00	5.00 (dt, J = 4.7, 2.1)<br>4.60 (dt, J = 4.7, 2.1)
19	CH_2	110.04	4.94 (m)<br>4.80 (m)
20	CH_3	14.32	1.50 (s)

[a] Carbon numbering as shown in Figure S64. [b] Chemical shifts δ in ppm, multiplicity: s = singlet, d = doublet, m = multiplet, t = triplet, br = broad coupling constants J are given in Hertz.

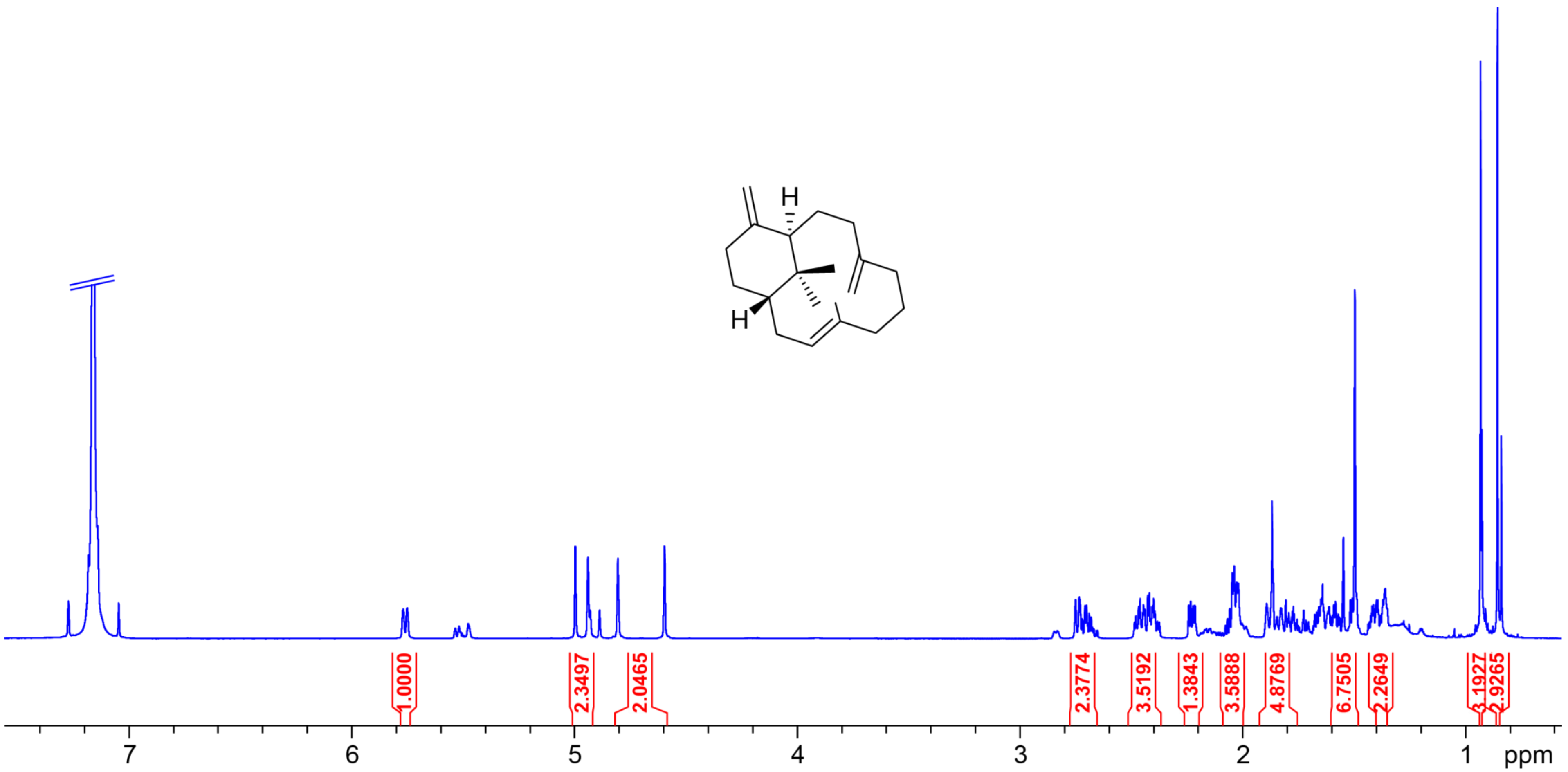

Figure S65. ^{1}H-NMR spectrum of **15** (700 MHz, C_6D_6).

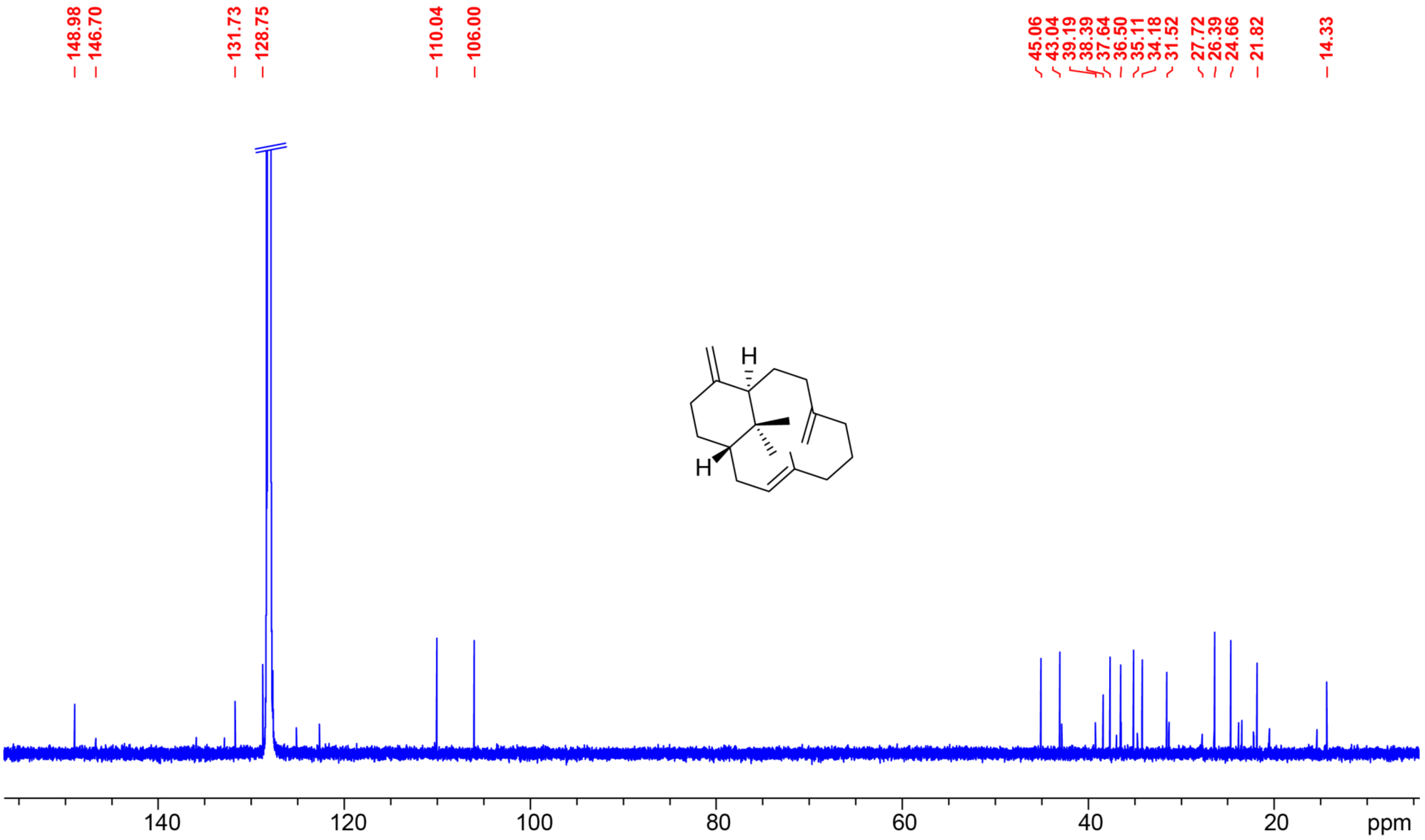

Figure S66. ^{13}C-NMR spectrum of **15** (176 MHz, C_6D_6).

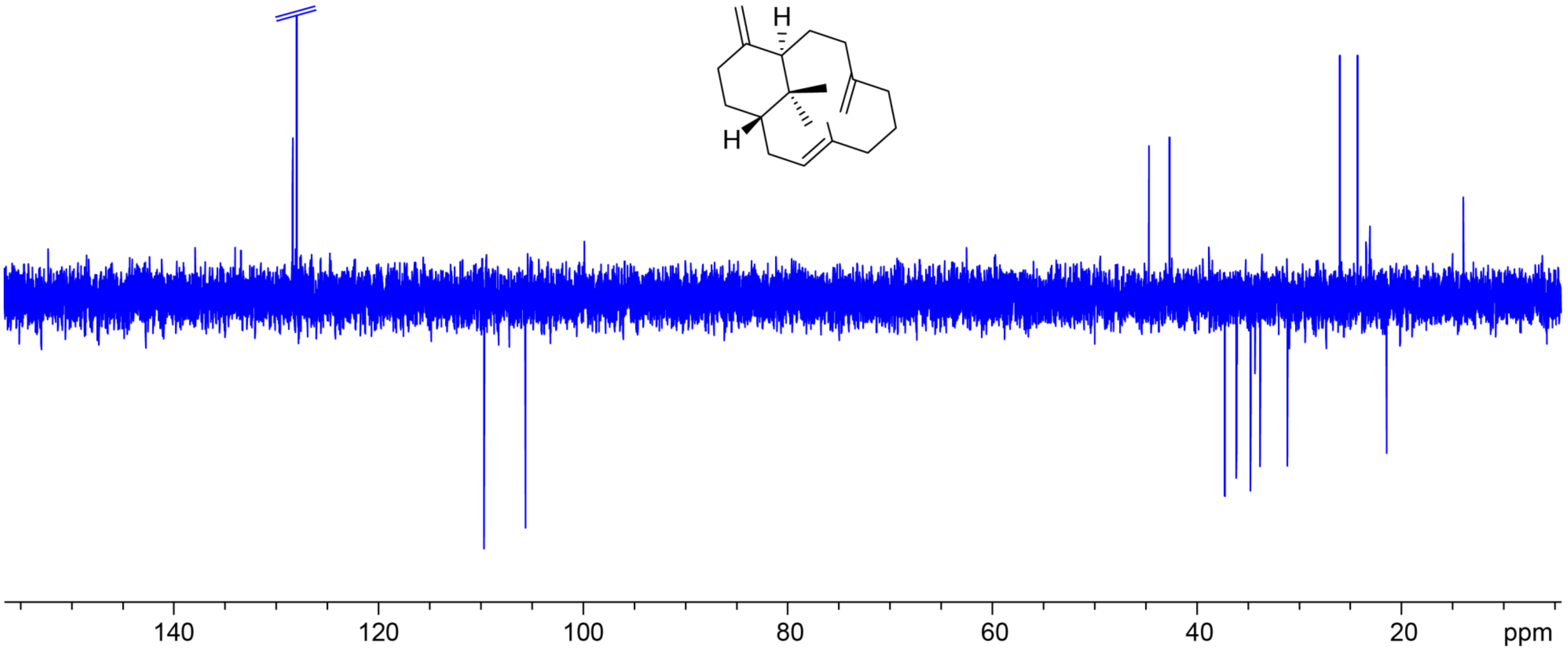

Figure S67. ^{13}C-DEPT135 spectrum of **15** (176 MHz, C_6D_6).

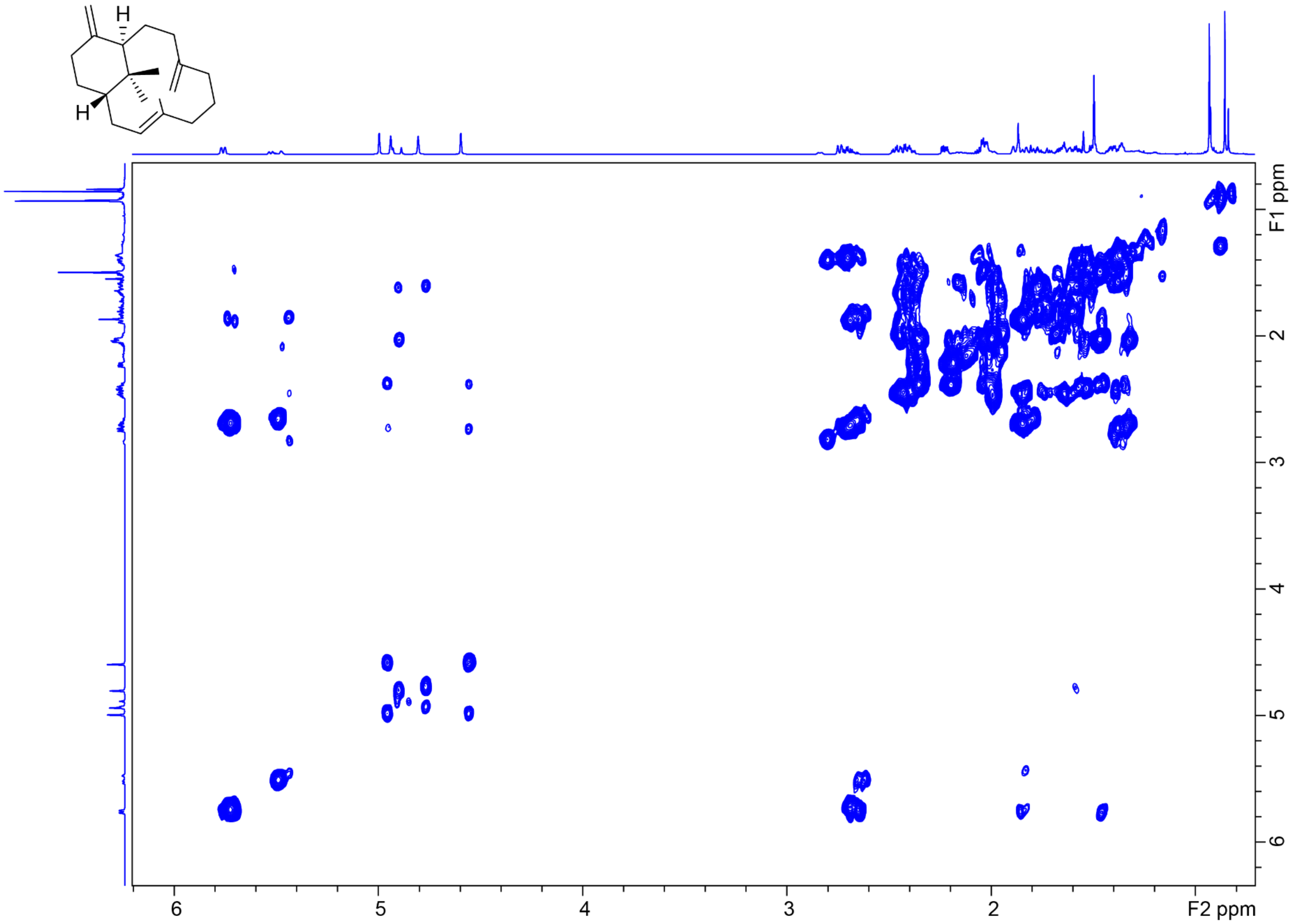

Figure S68. ^{1}H-^{1}H-COSY spectrum (C_6D_6) of **15**.

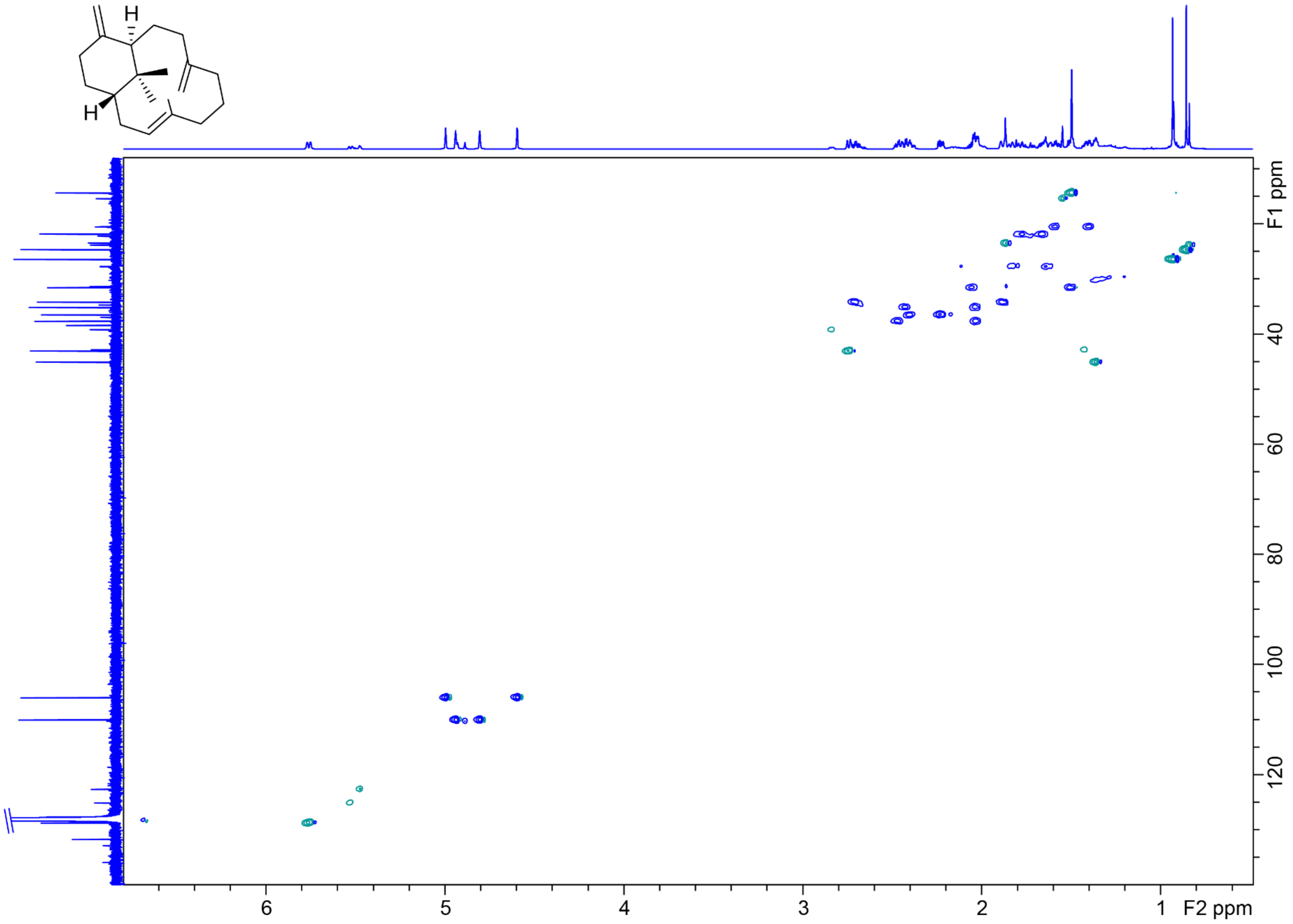

Figure S69. HSQC spectrum (C_6D_6) of **15**.

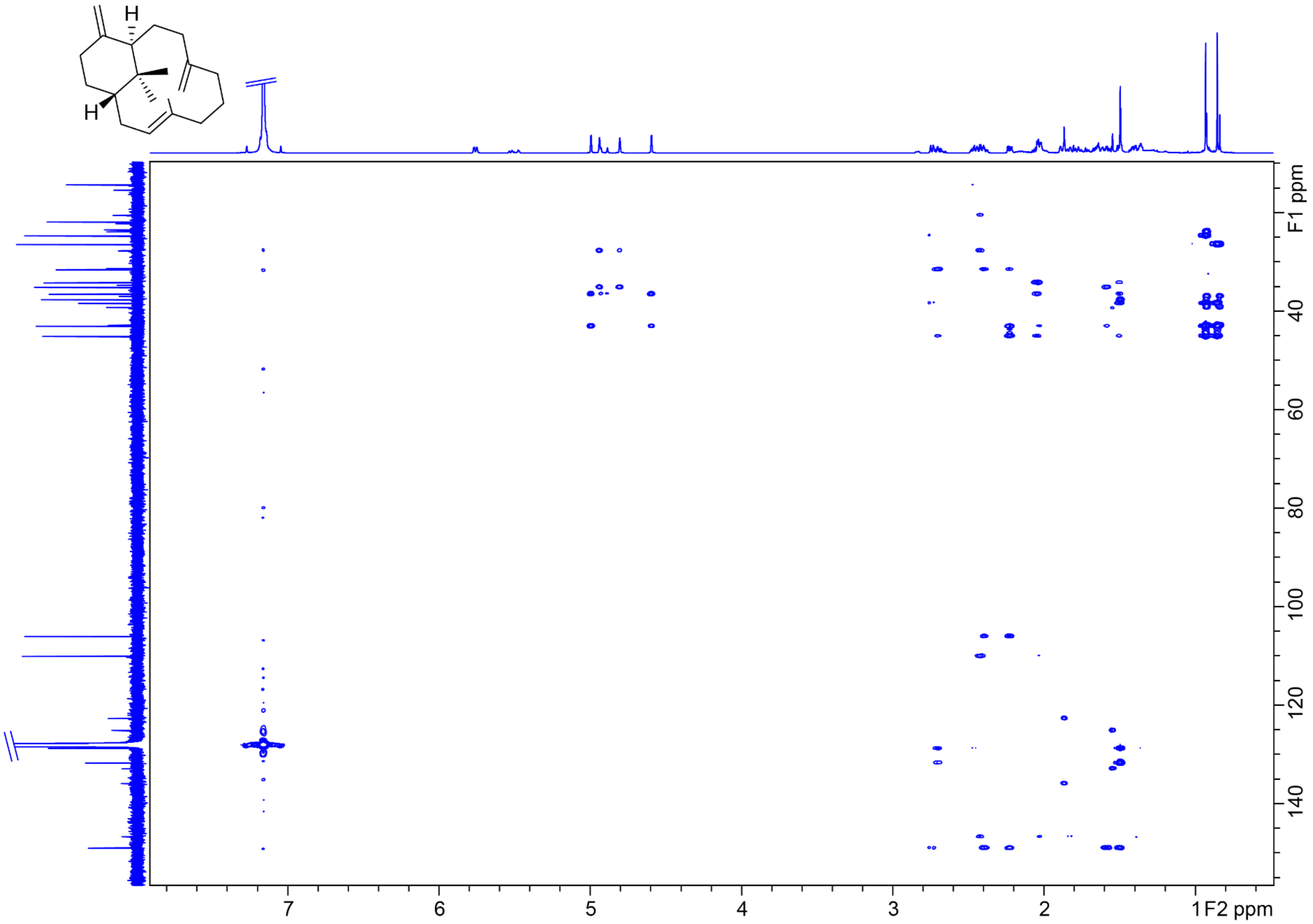

Figure S70. HMBC spectrum (C_6D_6) of **15**.

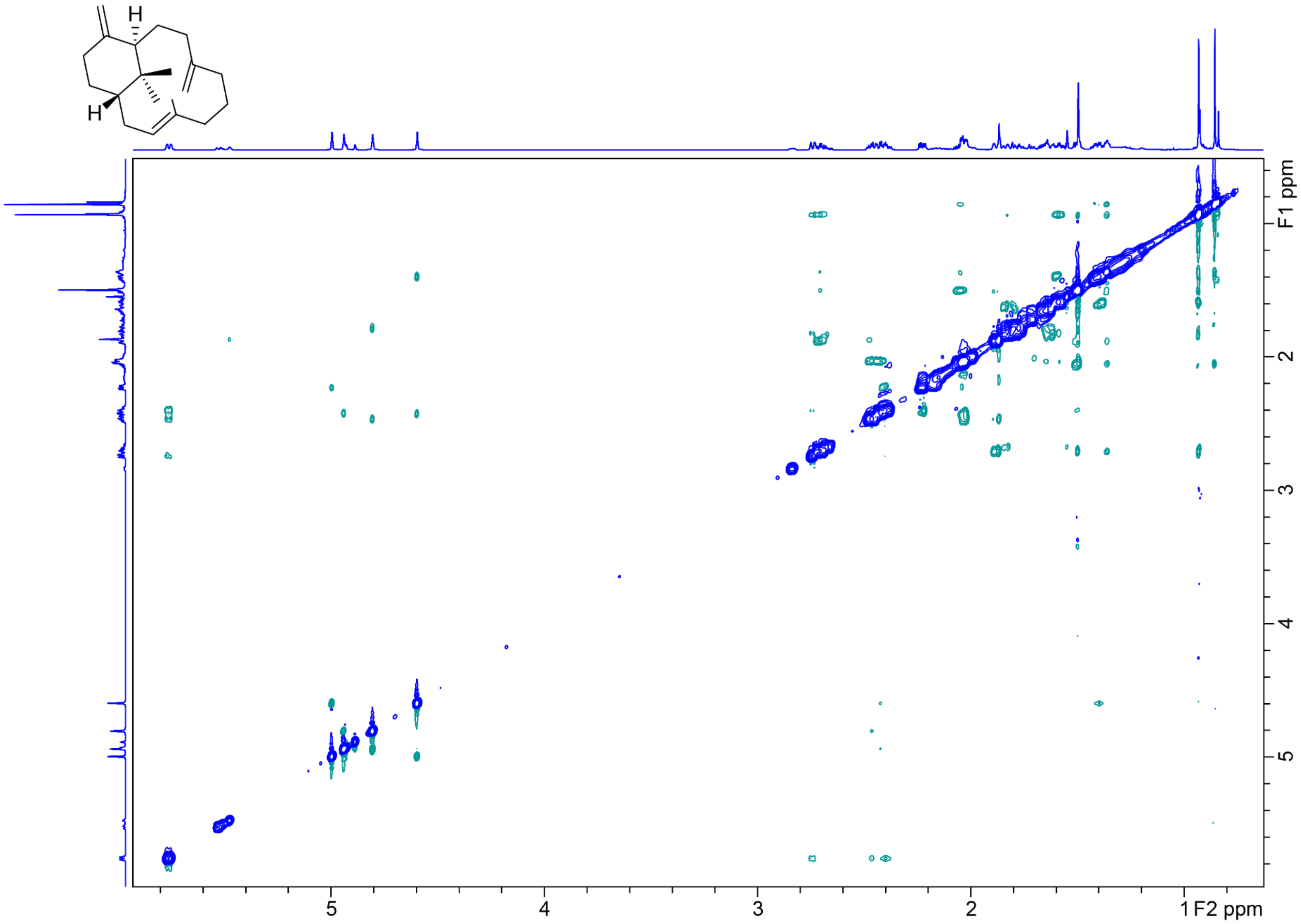

Figure S71. NOESY spectrum (C_6D_6) of **15**.

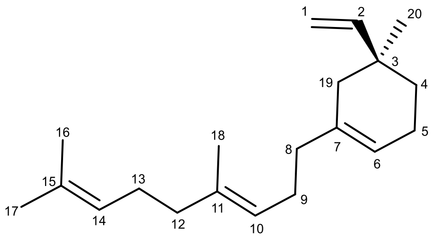

16

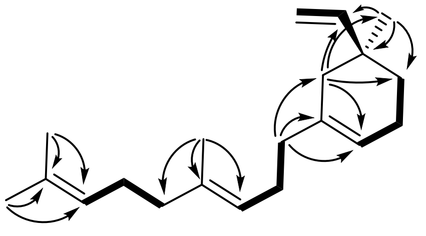

Figure S72. Structure elucidation of taxasimplene (**16**). Bold: ^{1}H,^{1}H-COSY, single headed arrows: key HMBC, and double headed arrows: NOESY correlations. Carbon numbering follows GGPP numbering to indicate the origin of each carbon.

Table S17. NMR data of taxasimplene (**16**) in C_6D_6 recorded at 298 K.

C[a]	type	^{13}C[b]	^{1}H[b]
1	CH_2	110.58	5.00 (dd, *J* = 1.4, 17.5)<br>4.98 (dd, *J* = 1.4, 17.5)
2	CH	147.82	5.83 (dd, *J* = 10.8, 17.5)
3	C_q	35.38	–
4	CH_2	33.91	1.43 (td, *J* = 12.6, 6.2)<br>1.34 (td, *J* = 12.6, 6.2)
5	CH_2	23.30	2.02 (m, 2H)
6	CH	120.31	5.43 (m)
7	C_q	136.06	–
8	CH_2	38.44	2.06 (m, 2H)
9	CH_2	26.86	2.20 (m, 2H)
10	CH	124.93	5.30 (dt, *J* = 1.3, 7.1)
11	C_q	135.01	–
12	CH_2	40.24	2.10 (m, 2H)
13	CH_2	27.25	2.19 (m, 2H)
14	CH	124.98	5.25 (dt, *J* = 1.3, 7.1)
15	C_q	131.15	–
16	CH_3	25.88	1.69 (s)
17	CH_3	17.77	1.57 (s)
18	CH_3	16.19	1.61 (s)
19	CH_2	40.10	1.97 (m)<br>1.75 (m)
20	CH_3	25.98	1.00 (s)

[a] Carbon numbering as shown in Figure S72. [b] Chemical shifts δ in ppm, multiplicity: s = singlet, d = doublet, m = multiplet, t = triplet, coupling constants *J* are given in Hertz.

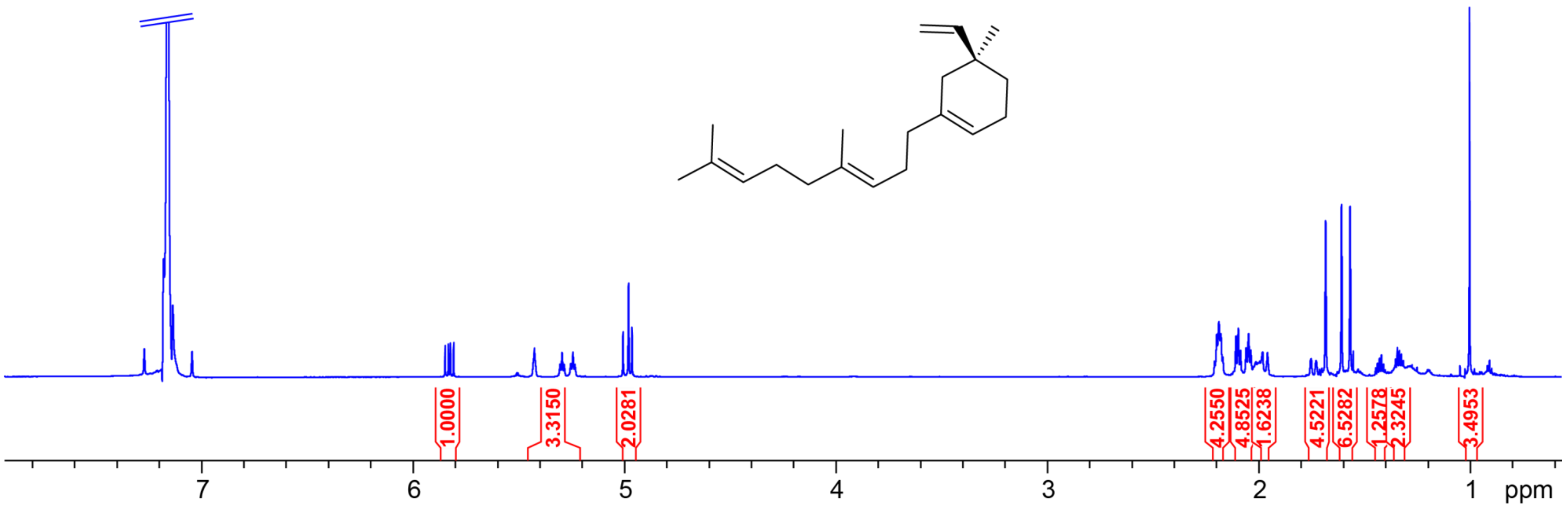

Figure S73. ^{1}H-NMR spectrum of **16** (700 MHz, C_6D_6).

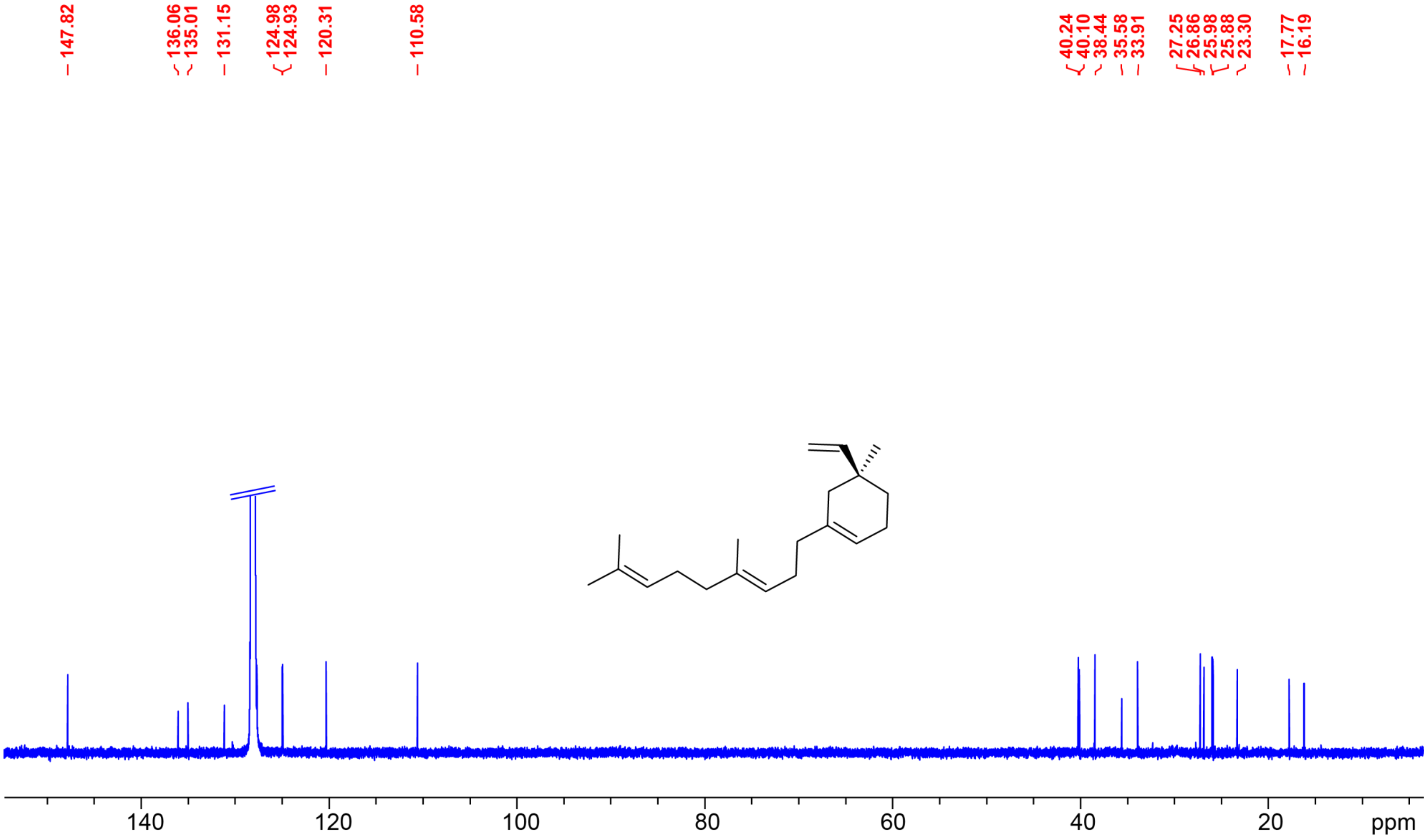

Figure S74. ^{13}C-NMR spectrum of **16** (176 MHz, C_6D_6).

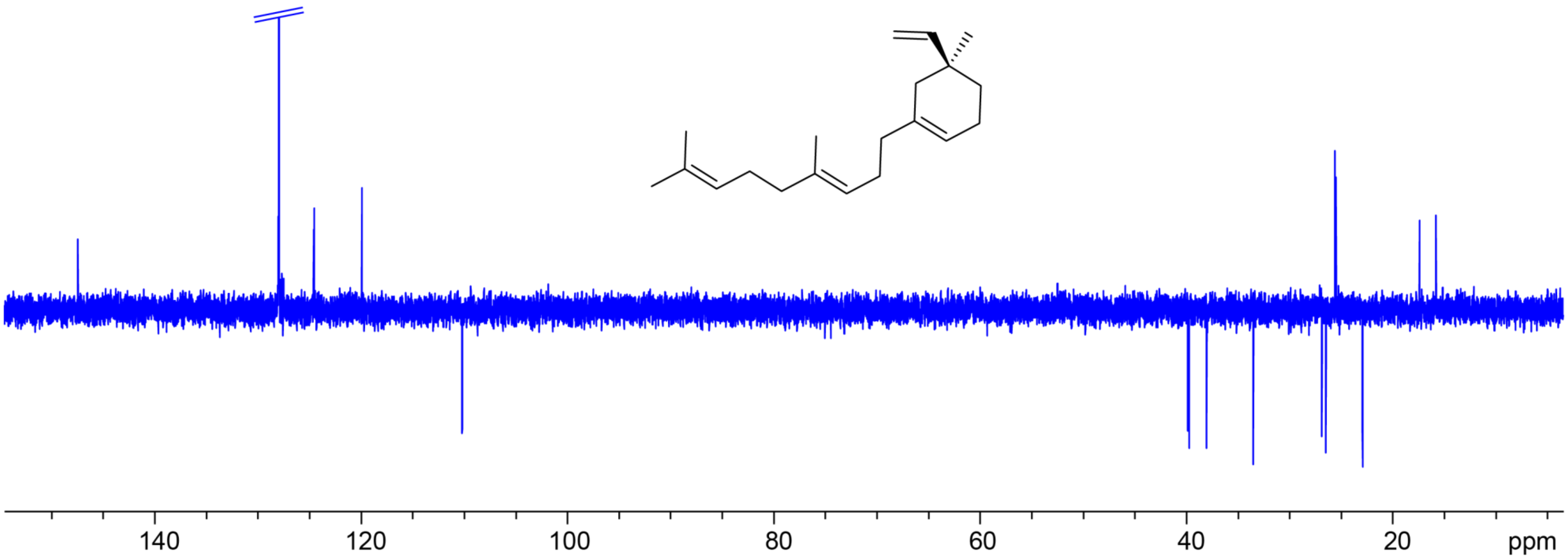

Figure S75. ^{13}C-DEPT135 spectrum of **16** (176 MHz, C_6D_6).

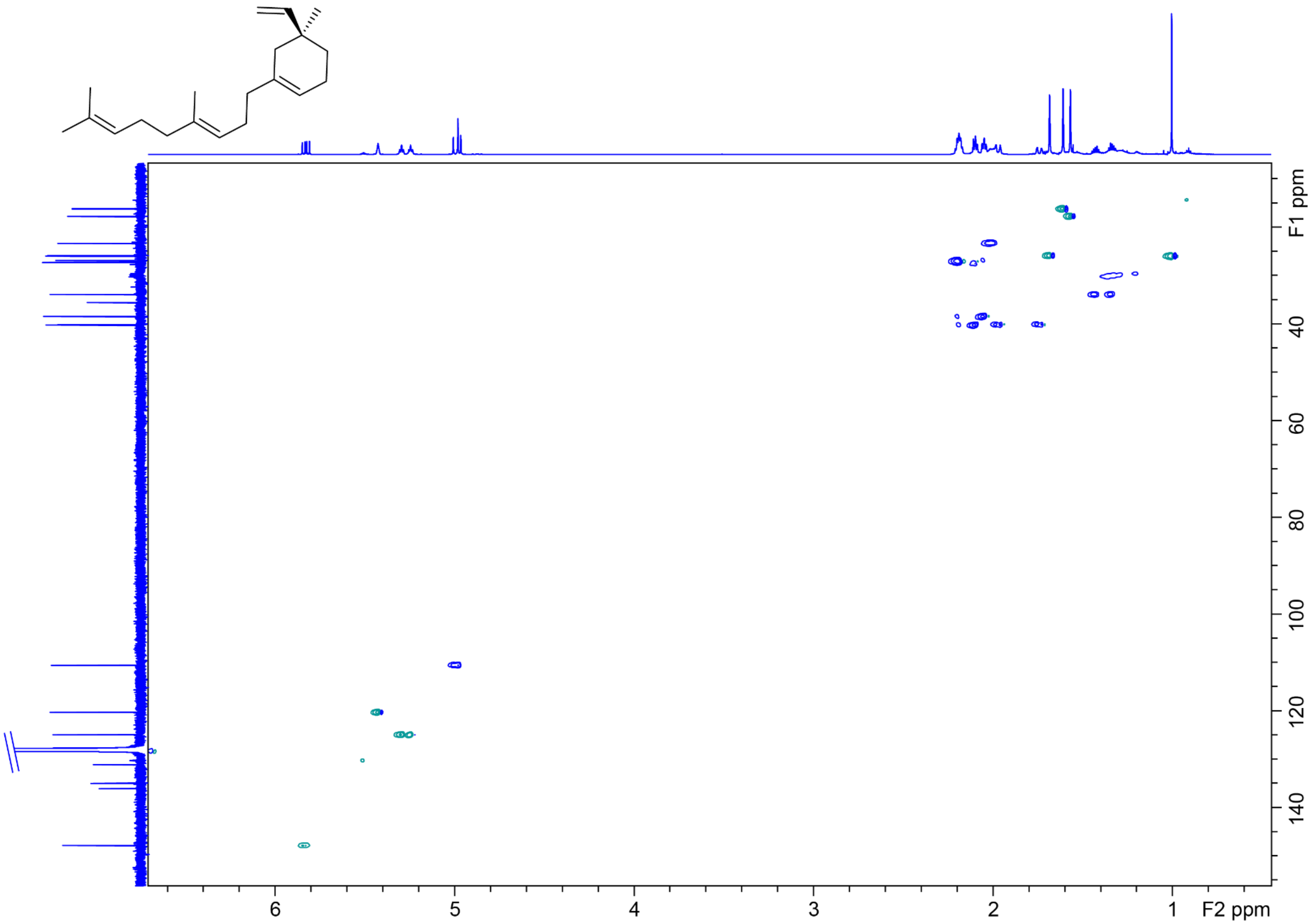

Figure S76. ^{1}H-^{1}H-COSY spectrum (C_6D_6) of **16**.

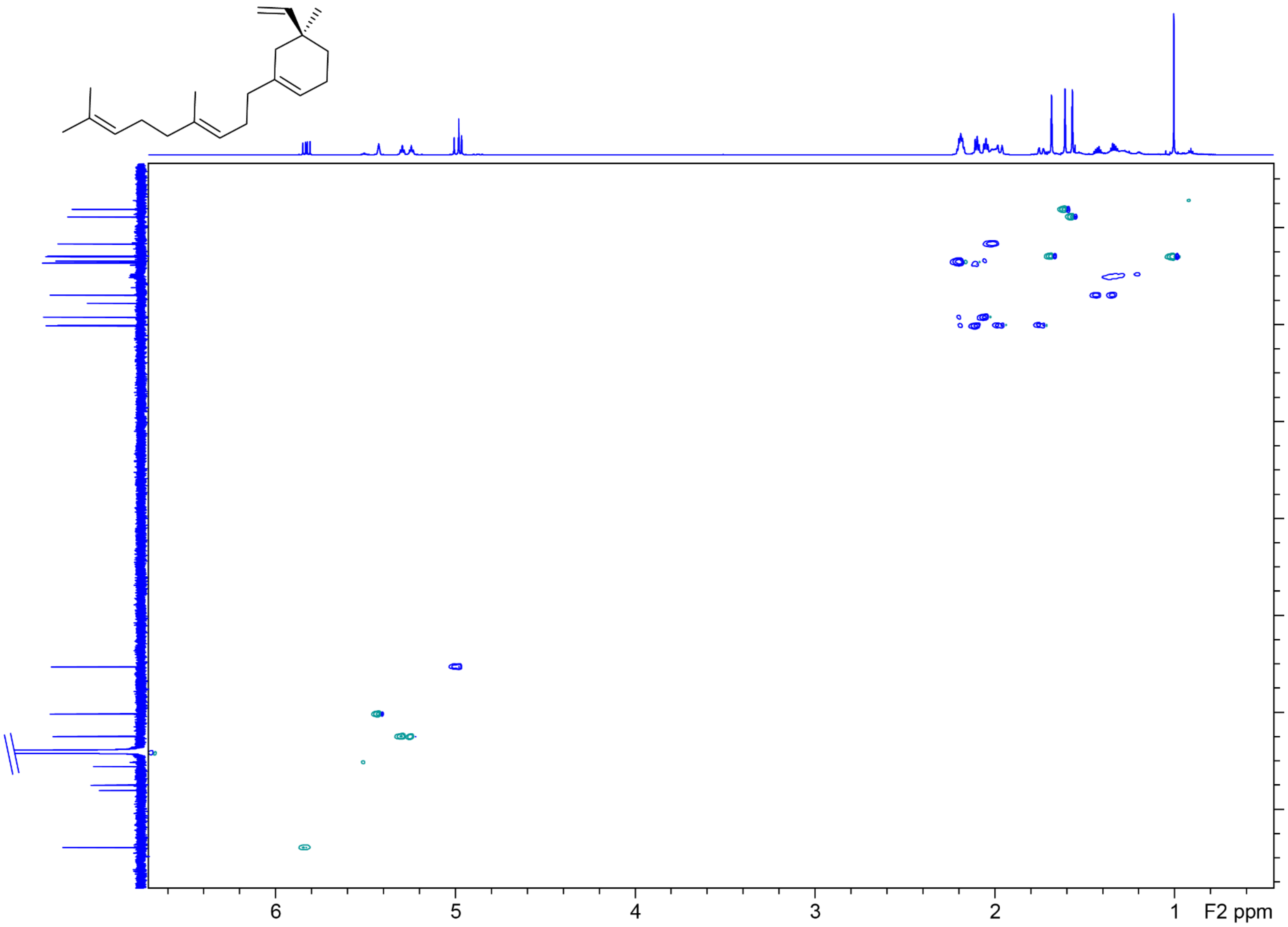

Figure S77. HSQC spectrum (C_6D_6) of **16**.

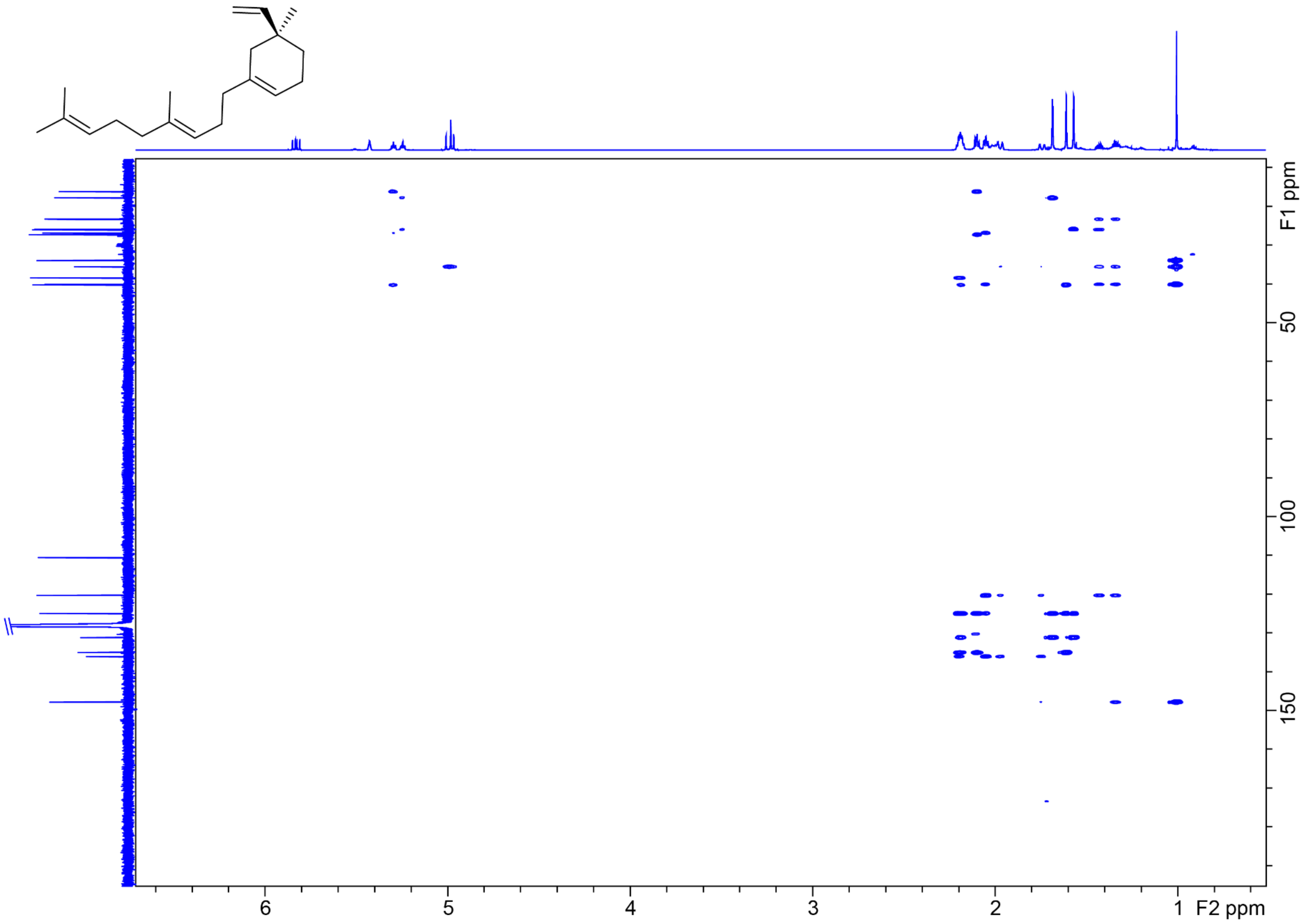

Figure S78. HMBC spectrum (C_6D_6) of **16**.

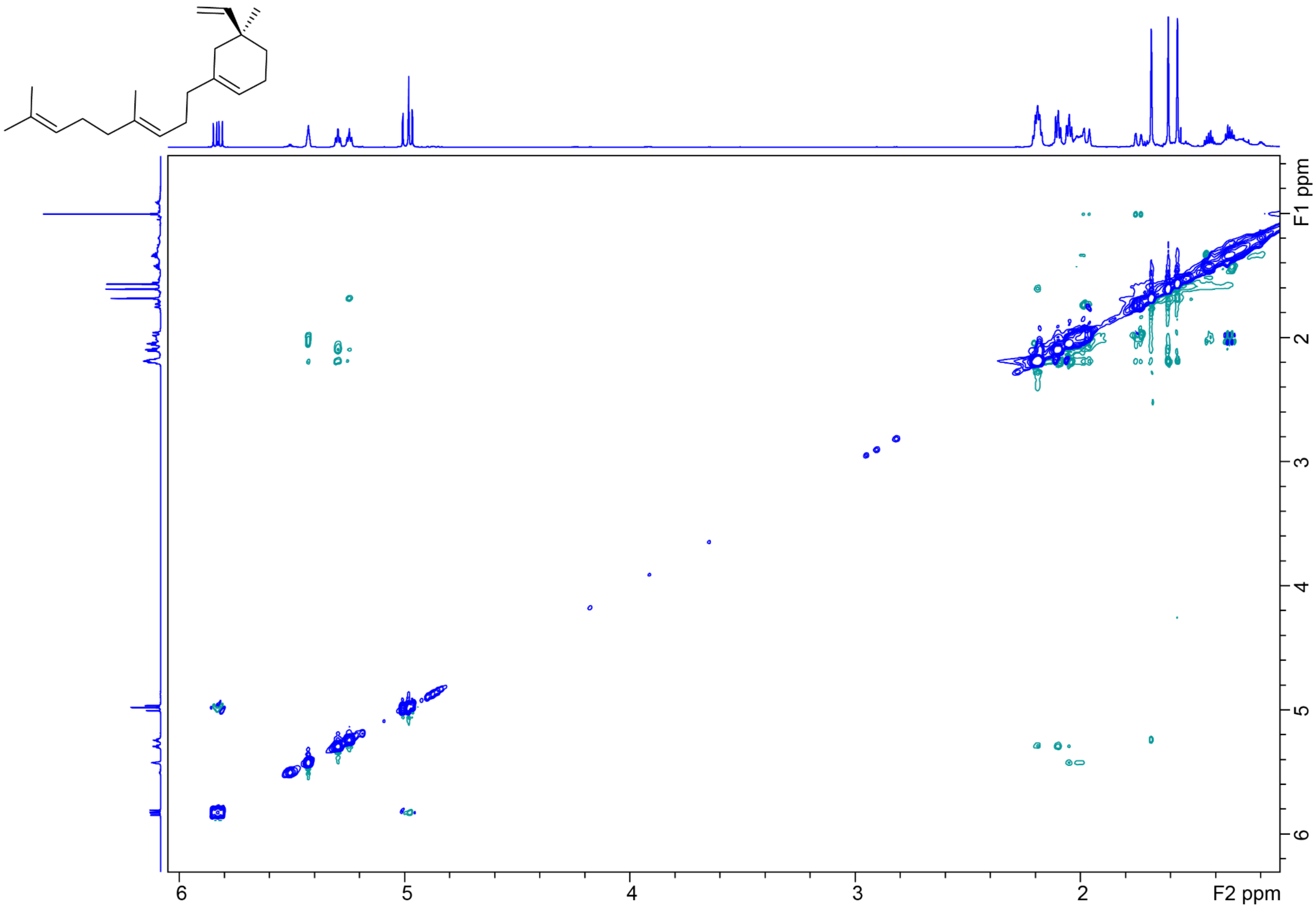

Figure S79. NOESY spectrum (C_6D_6) of **16**.

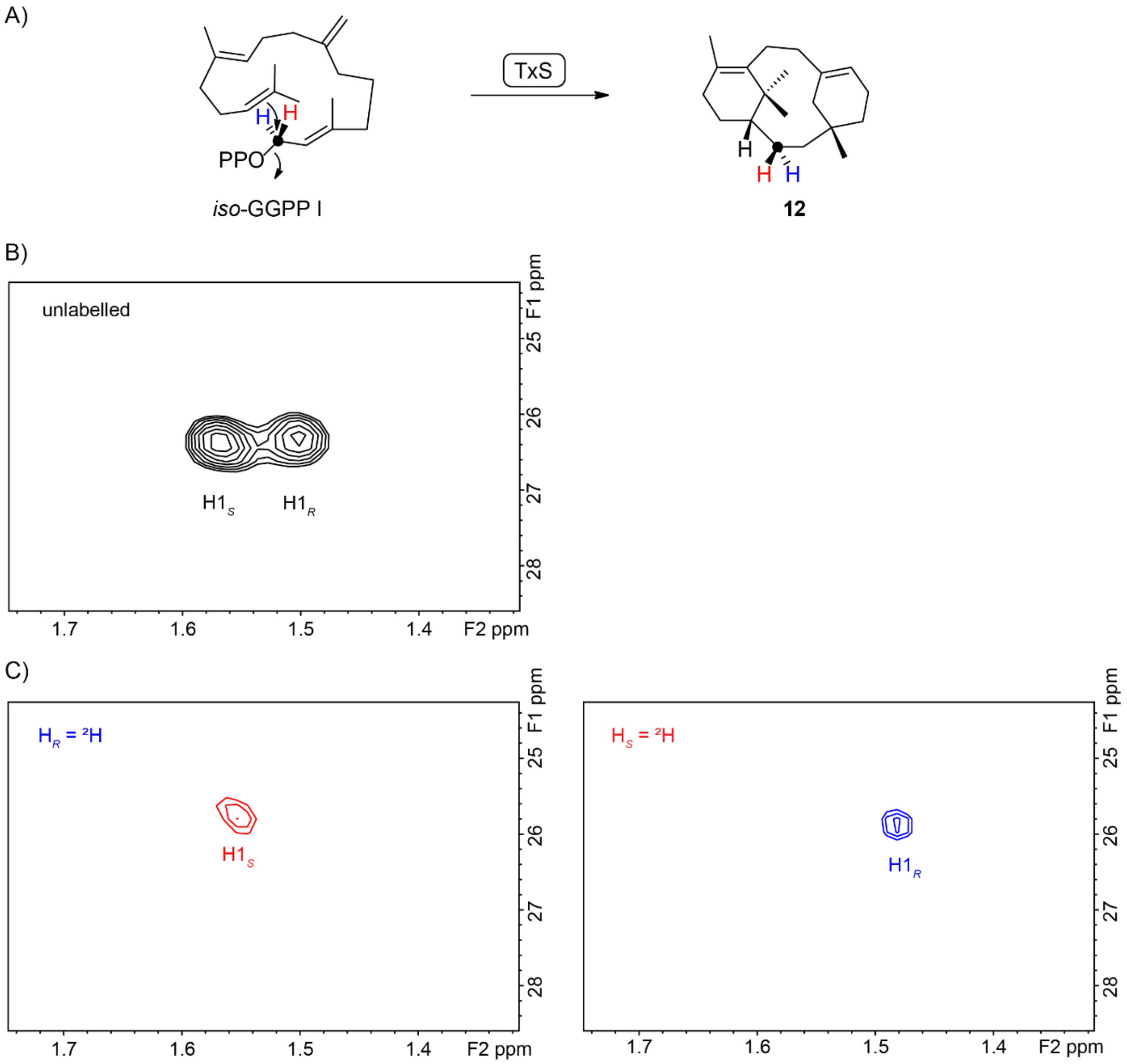

Figure S80. The absolute configuration of **12**. A) Cyclisation of (*R*)- (blue H = ^{2}H) and (*S*)-(1-^{13}C,1-^{2}H)-*iso*-GGPP I (red H = ^{2}H) into **12** with inversion of configuration at C1. B) Partial HSQC spectrum of unlabelled **12** showing the region for C1. C) HSQC spectra of labelled **12** obtained from (*R*)-(1-^{13}C,1-^{2}H)-*iso*-GGPP I (left) and from (*S*)-(1-^{13}C,1-^{2}H)-*iso*-GGPP I (right). Together with the NOESY based assignments for the hydrogens at C1 (Table S12) these data point to the shown absolute configuration of **12**.

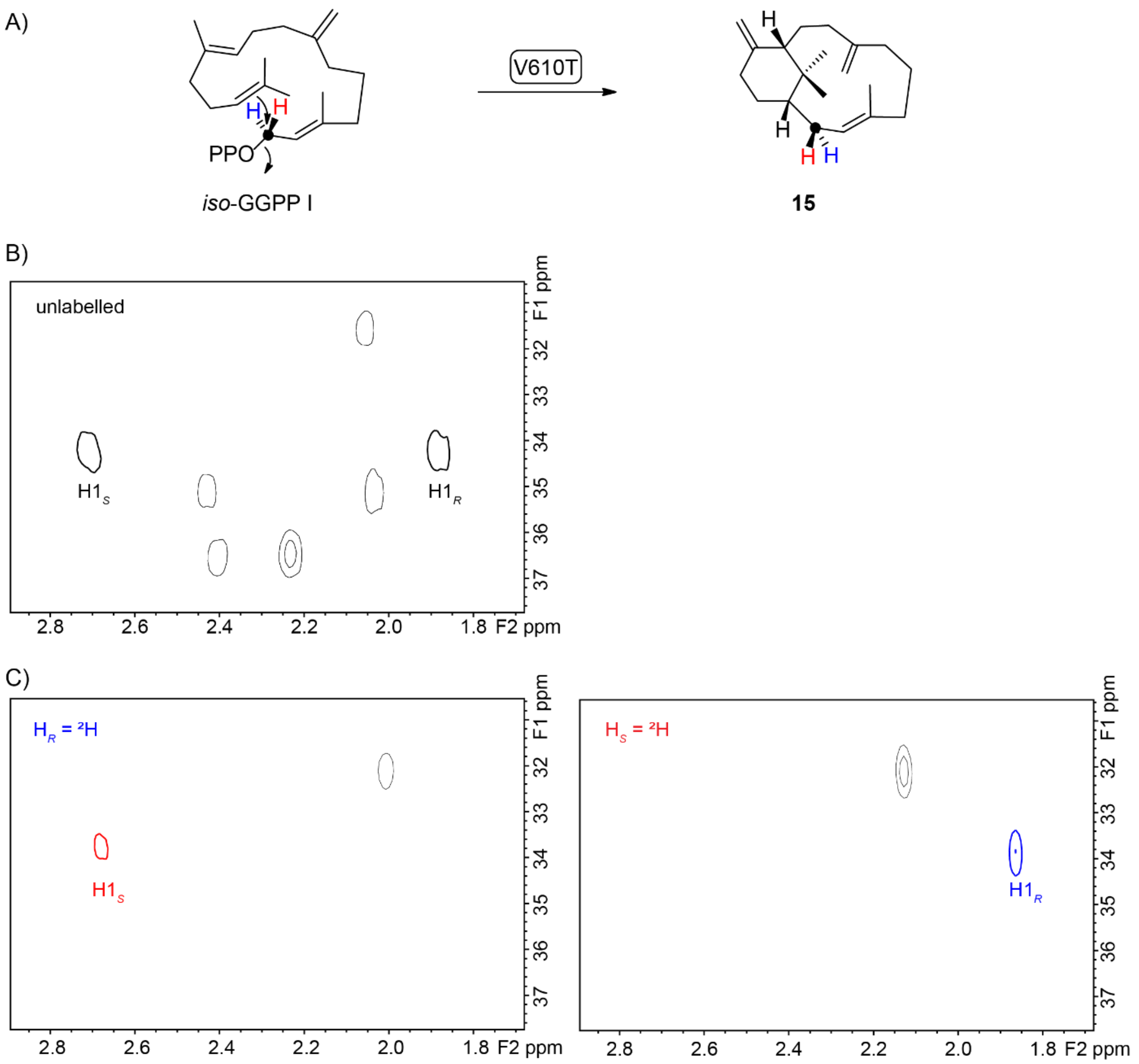

Figure S81. The absolute configuration of **15**. A) Cyclisation of (*R*)- (blue H = ^{2}H) and (*S*)-(1-^{13}C,1-^{2}H)-*iso*-GGPP I (red H = ^{2}H) into **15** with inversion of configuration at C1. B) Partial HSQC spectrum of unlabelled **15** showing the region for C1. C) HSQC spectra of labelled **15** obtained from (*R*)-(1-^{13}C,1-^{2}H)-*iso*-GGPP I (left) and from (*S*)-(1-^{13}C,1-^{2}H)-*iso*-GGPP I (right). Together with the NOESY based assignments for the hydrogens at C1 (Table S16) these data point to the shown absolute configuration of **15**.

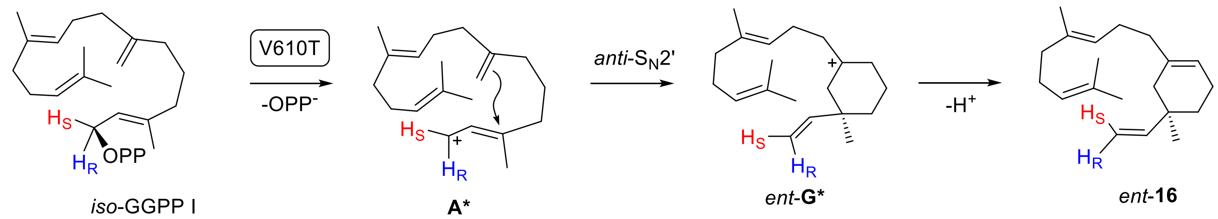

Scheme S3. Alternative cyclisation of *iso*-GGPP I leading to *ent*-**16**.

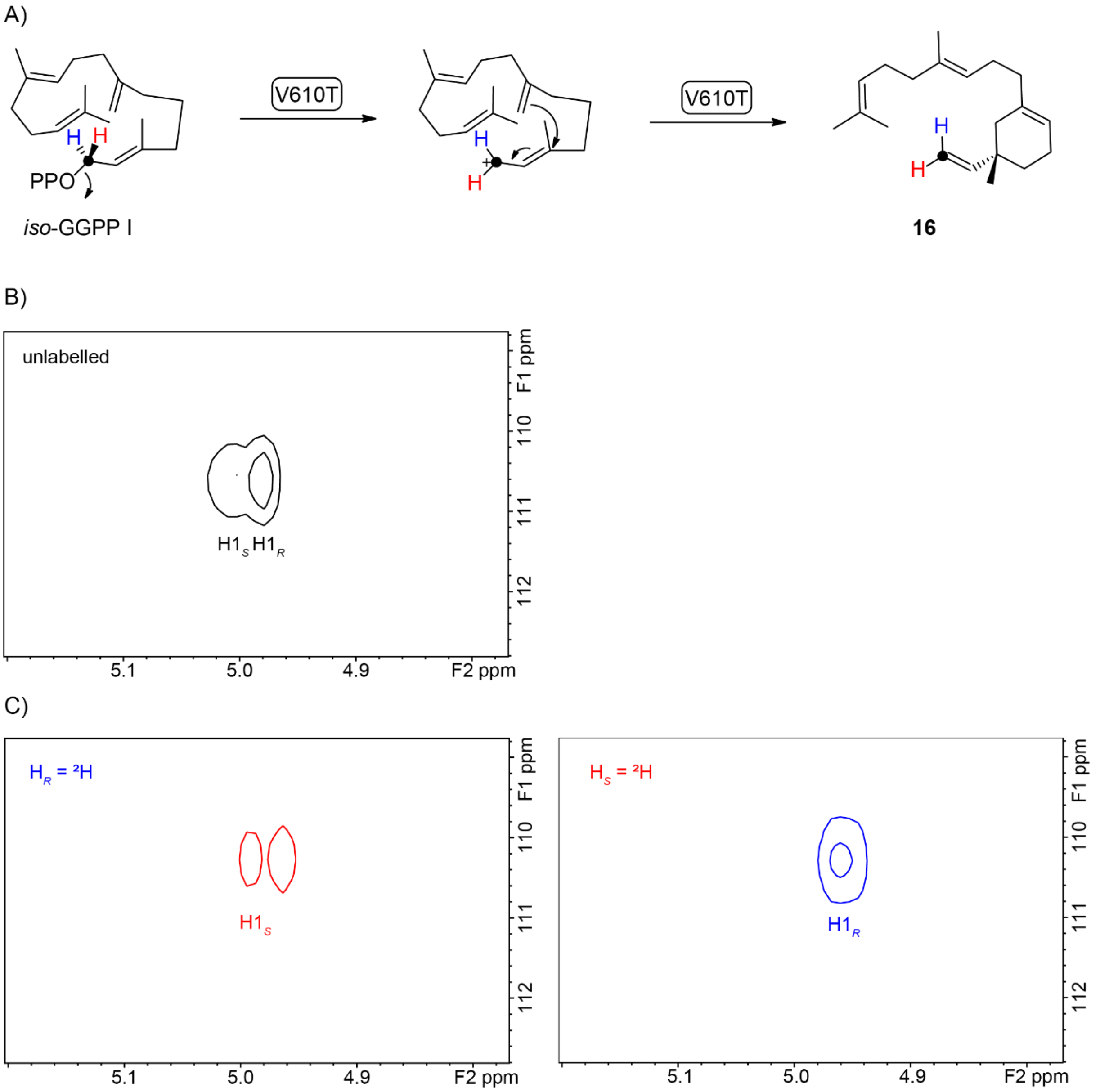

Figure S82. The absolute configuration of **16**. A) Cyclisation of (*R*)- (blue H = ^{2}H) and (*S*)-(1-^{13}C,1-^{2}H)-*iso*-GGPP I (red H = ^{2}H) into **15** with inversion of configuration at C1. B) Partial HSQC spectrum of unlabelled **15** showing the region for C1. C) HSQC spectra of labelled **15** obtained from (*R*)-(1-^{13}C,1-^{2}H)-*iso*-GGPP I (left) and from (*S*)-(1-^{13}C,1-^{2}H)-*iso*-GGPP I (right). Together with the NOESY based assignments for the hydrogens at C1 (Table S16) these data point to the shown absolute configuration of **15**.

Computational data

Cartesian coordinates, Gibbs energies (G"..." in Hartree) and imagininary frequencies of TS (T-"..." in cm^{-1}) (mPW1PW91/6-311+G(d,p)//B97D3/6-31G(d,p)-sp-density-fitting, 1 bar, 298.15 K).

Structures from Scheme 3 of main text (Table S5):

A$^{+\bullet}$ (G781.109516)

C	-1.230243	0.418372	1.595198
C	-1.461372	-0.664845	0.735771
C	-2.295145	-0.402083	-0.509170
C	-1.585497	0.810811	-1.215320
C	-1.753288	2.074215	-0.341691
C	-1.533090	1.837378	1.178000
H	-1.082220	2.868037	-0.692870
H	-2.767884	2.461339	-0.494104
H	-0.698536	2.456022	1.551081
H	-2.402068	2.193431	1.759015
C	-0.656654	0.275439	2.977505
H	-0.587875	-0.758604	3.327019
H	0.343129	0.735162	3.038782
H	-1.284742	0.837283	3.686335
C	-0.125680	0.493658	-1.659212
C	-0.764545	-1.979188	0.947618
C	0.965453	0.278013	-0.558247
H	-0.155447	-0.375090	-2.323520
C	0.791920	-1.875480	0.909891
C	1.490986	-1.220456	-0.324526
H	1.173888	-2.902742	1.003524
H	-2.144228	0.984063	-2.150822
C	-2.444769	-1.593979	-1.476571
H	-2.945914	-2.442770	-0.993765
H	-3.075620	-1.282283	-2.319043
H	-1.501531	-1.954038	-1.896493
C	-3.752864	-0.023337	-0.087114
H	-4.307138	0.324038	-0.969214
H	-4.261082	-0.914189	0.303024
H	-3.818756	0.751010	0.683392
H	-1.024358	-2.386689	1.938725
H	-1.105728	-2.715648	0.214457
H	1.120784	-1.344935	1.813994
C	3.010331	-1.224171	0.010073
H	3.575851	-1.105478	-0.927707
C	3.442511	-0.103826	0.966128
H	2.926293	-0.193520	1.939833
H	4.515785	-0.167632	1.191654
C	3.123530	1.198272	0.311535
H	3.827921	2.029504	0.397599
C	2.019493	1.362451	-0.490368
H	3.282931	-2.206795	0.422464
H	0.420051	0.485786	0.407849
C	1.336678	-2.122955	-1.567221

H	1.884162	-3.060540	-1.398145
H	0.300940	-2.402125	-1.784612
H	1.760354	-1.650652	-2.463364
C	1.835738	2.681403	-1.198099
H	2.626946	3.386328	-0.919195
H	1.874320	2.552567	-2.291388
H	0.870746	3.155228	-0.975218
H	0.178053	1.325794	-2.304381

$A^{+\bullet}$-$B^{+\bullet}$-TS (G781.052547, T-467)

C	-1.850589	0.593277	1.282347
C	-2.038588	-0.536240	0.404827
C	-3.073233	-0.573446	-0.545601
C	-0.143910	2.212807	-1.009883
C	-0.387922	2.741852	0.273555
C	-2.079190	1.937975	0.843568
H	0.249981	2.423346	1.099003
H	-0.674927	3.793820	0.322357
H	-2.267473	2.676929	1.625978
H	-2.660132	2.105969	-0.058247
C	-1.279035	0.421492	2.667019
H	-1.363630	-0.604110	3.037851
H	-0.214908	0.704421	2.716667
H	-1.804960	1.088012	3.365123
C	0.349689	0.837076	-1.262600
C	-1.218486	-1.813461	0.637463
C	1.356435	0.258292	-0.249612
H	-0.574565	0.225053	-1.222481
C	0.309423	-1.805261	0.901173
C	1.340791	-1.330049	-0.174153
H	0.532714	-2.865421	1.097212
H	-0.522251	2.772236	-1.870478
C	-3.154091	-1.651266	-1.596861
H	-3.626392	-2.560389	-1.186323
H	-3.789067	-1.321352	-2.429191
H	-2.176886	-1.944503	-1.997539
C	-4.286667	0.321770	-0.526707
H	-4.306259	0.993633	-1.400730
H	-5.180901	-0.313234	-0.630183
H	-4.399795	0.912660	0.386045
H	-1.673170	-2.303622	1.517482
H	-1.412493	-2.509929	-0.184317
H	0.540683	-1.288714	1.843535
C	2.737896	-1.842799	0.304384
H	3.369130	-2.028594	-0.577118
C	3.492098	-0.839800	1.197002
H	2.902991	-0.615960	2.107014
H	4.438280	-1.275476	1.544808
C	3.734470	0.403888	0.382606
H	4.728651	0.856119	0.357920
C	2.748585	0.891321	-0.394601
H	2.615551	-2.810595	0.812861
H	1.008703	0.567423	0.753553
C	1.057251	-1.979979	-1.543698

H	1.125932	-3.073994	-1.465365
H	0.060506	-1.744291	-1.940992
H	1.795432	-1.655649	-2.289941
C	2.967207	2.067774	-1.314321
H	3.993366	2.444229	-1.225037
H	2.802376	1.804222	-2.371160
H	2.289384	2.911579	-1.094570
H	0.701331	0.744856	-2.299171

B$^{+\bullet}$ (G781.052066)

C	-2.011486	0.246650	1.471069
C	-2.133419	-0.580338	0.266870
C	-3.086329	-0.310909	-0.726403
C	-0.008744	2.424568	-0.695888
C	0.308415	3.068979	0.449793
C	-2.159858	1.610313	1.402916
H	0.940827	2.622595	1.216225
H	-0.032469	4.088211	0.629404
H	-2.044684	2.229659	2.292810
H	-2.405757	2.128209	0.482628
C	-1.666868	-0.379292	2.811721
H	-1.883946	-1.451923	2.847867
H	-0.607914	-0.241797	3.070959
H	-2.250894	0.113398	3.600396
C	0.343650	1.015760	-1.074501
C	-1.317718	-1.862939	0.182754
C	1.348265	0.277873	-0.175266
H	-0.613196	0.461814	-1.089727
C	0.172959	-1.915478	0.595729
C	1.302330	-1.309161	-0.302290
H	0.382332	-2.993807	0.657896
H	-0.623882	2.954250	-1.431135
C	-3.106509	-1.064568	-2.032989
H	-3.601118	-2.043100	-1.906159
H	-3.688142	-0.514814	-2.783231
H	-2.106663	-1.257020	-2.439605
C	-4.239213	0.641722	-0.565411
H	-4.112956	1.520929	-1.220000
H	-5.156476	0.145895	-0.920966
H	-4.402202	0.983422	0.459567
H	-1.835977	-2.579420	0.847985
H	-1.418271	-2.304082	-0.813832
H	0.311630	-1.537666	1.618206
C	2.641696	-1.915593	0.248526
H	3.333797	-2.084370	-0.588910
C	3.367243	-0.999799	1.258697
H	2.710628	-0.794710	2.125725
H	4.264940	-1.489218	1.657375
C	3.710871	0.263264	0.527791
H	4.735134	0.643347	0.527340
C	2.767906	0.840583	-0.269491
H	2.447039	-2.900716	0.694788
H	1.033544	0.469223	0.871044
C	1.152919	-1.754239	-1.770580

H	1.188595	-2.850372	-1.843448
H	0.210076	-1.425078	-2.226929
H	1.973187	-1.354959	-2.383029
C	3.113503	1.974027	-1.186828
H	4.180682	2.218200	-1.133866
H	2.869035	1.727040	-2.232921
H	2.536093	2.885181	-0.956209
H	0.669319	1.003142	-2.127546

B$^{+\bullet}$ (G781.051526)

C	2.075327	-1.033060	-1.254210
C	2.087045	-0.623550	0.166746
C	2.859238	0.447889	0.608660
C	0.112599	2.572530	-0.026377
C	-0.029258	2.778792	-1.346221
C	1.791018	-0.131308	-2.237269
H	-0.627012	2.125243	-1.982859
H	0.433002	3.637407	-1.832922
H	1.714186	-0.447296	-3.278346
H	1.600918	0.916909	-2.013740
C	2.352660	-2.485360	-1.586834
H	3.367942	-2.749877	-1.253448
H	1.670040	-3.183170	-1.085069
H	2.288125	-2.661788	-2.666415
C	-0.451052	1.442808	0.801543
C	1.285265	-1.505160	1.106679
C	-1.274802	0.367189	0.072330
H	0.394156	0.967958	1.313179
C	-0.127015	-1.926991	0.621895
C	-1.379179	-1.020676	0.860204
H	-0.351077	-2.876425	1.131170
H	0.702051	3.293225	0.550350
C	2.950573	0.868119	2.051069
H	3.930639	0.567587	2.457825
H	2.923193	1.966305	2.120452
H	2.179008	0.459129	2.708275
C	3.733128	1.271037	-0.299498
H	3.223515	2.224560	-0.523428
H	4.666375	1.541601	0.215381
H	3.978402	0.786454	-1.247559
H	1.862634	-2.438193	1.243298
H	1.224359	-1.070038	2.109130
H	-0.089861	-2.165559	-0.451113
C	-2.587268	-1.856913	0.306157
H	-3.469415	-1.694045	0.941558
C	-2.984873	-1.477224	-1.138833
H	-2.122065	-1.617579	-1.819203
H	-3.788157	-2.122187	-1.516431
C	-3.404278	-0.043557	-1.102614
H	-4.365092	0.269833	-1.517982
C	-2.636596	0.853757	-0.396173
H	-2.354122	-2.928953	0.366359
H	-0.723584	0.110964	-0.858321
C	-1.612947	-0.782961	2.366007

H	-1.778906	-1.742472	2.875556
H	-0.764988	-0.292007	2.860387
H	-2.503070	-0.160617	2.533009
C	-3.150044	2.223902	-0.083835
H	-4.139144	2.394533	-0.523297
H	-3.234224	2.356489	1.008520
H	-2.463344	3.013042	-0.424298
H	-1.041716	1.883386	1.623420

B$^{+•}$-C$^{+•}$-TS (G781.031695, T-1583)

C	1.468895	-1.019008	-1.316852
C	2.018975	-0.774774	0.035039
C	2.977533	0.158603	0.284701
C	0.410903	2.585629	0.369612
C	0.270061	3.098077	-0.862826
C	0.654772	-0.008548	-1.860864
H	-0.422871	2.695973	-1.601843
H	0.850944	3.967136	-1.169954
H	0.148602	-0.175140	-2.816952
H	0.957925	1.021384	-1.658874
C	1.607555	-2.361426	-1.963541
H	2.523053	-2.327455	-2.582018
H	1.756731	-3.173410	-1.242209
H	0.774632	-2.598788	-2.638166
C	-0.272403	1.409110	1.034842
C	1.322644	-1.634037	1.094425
C	-1.172032	0.435898	0.267041
H	0.513775	0.840573	1.544600
C	-0.148531	-2.002001	0.780417
C	-1.334730	-0.988656	0.937272
H	-0.408399	-2.835977	1.449325
H	1.118350	3.077881	1.044404
C	3.504068	0.469135	1.664123
H	4.542648	0.117030	1.763508
H	3.536253	1.559591	1.813608
H	2.926235	0.027358	2.482062
C	3.667789	0.930239	-0.817806
H	3.472052	2.008032	-0.707208
H	4.758333	0.803067	-0.740967
H	3.368318	0.626235	-1.826850
H	1.870779	-2.585224	1.205928
H	1.392418	-1.146568	2.074971
H	-0.205824	-2.432405	-0.228833
C	-2.576787	-1.725838	0.338810
H	-3.474502	-1.463468	0.917989
C	-2.841674	-1.345137	-1.130891
H	-1.958060	-1.575391	-1.755534
H	-3.676795	-1.920617	-1.549606
C	-3.144158	0.121156	-1.131287
H	-4.025677	0.503772	-1.650731
C	-2.386875	0.978804	-0.364255
H	-2.441499	-2.811294	0.442829
H	-0.364343	0.100110	-0.899206
C	-1.604629	-0.763148	2.452322

H	-1.906109	-1.718775	2.902598
H	-0.721557	-0.405486	2.995339
H	-2.421540	-0.046107	2.611189
C	-2.856404	2.395452	-0.128721
H	-3.700860	2.641062	-0.783117
H	-3.206472	2.483288	0.913082
H	-2.081631	3.157300	-0.256889
H	-0.884753	1.829138	1.858168

$C^{+•}$ (G781.063400)

C	-2.544050	1.425299	-0.596267
C	-2.242214	0.319322	0.243035
C	-3.094892	-0.793599	0.357653
C	0.498113	-2.583567	-0.333034
C	-0.129346	-1.952838	-1.335317
C	-3.235213	1.306488	-1.922897
H	0.104387	-0.925432	-1.612486
H	-0.889090	-2.461668	-1.927765
H	-4.218490	1.806212	-1.922001
H	-3.355889	0.276689	-2.267685
C	-2.109199	2.817932	-0.244476
H	-2.897186	3.526588	-0.540498
H	-1.902293	2.953175	0.821653
H	-1.211077	3.119811	-0.811663
C	1.574692	-2.010642	0.578734
C	-1.002315	0.367285	1.154452
C	2.063825	-0.622758	0.247991
H	1.214234	-2.075601	1.615401
C	0.241139	1.103294	0.607657
C	1.644391	0.577149	1.103989
H	0.198863	2.163442	0.891424
H	0.244460	-3.625763	-0.115995
C	-2.742264	-1.968289	1.234233
H	-2.753957	-1.680149	2.299098
H	-3.469590	-2.778715	1.110070
H	-1.742275	-2.360911	1.012570
C	-4.488867	-0.860696	-0.204938
H	-4.536001	-1.595951	-1.026391
H	-5.166393	-1.246178	0.572186
H	-4.893077	0.088466	-0.560913
H	-1.281400	0.792724	2.132728
H	-0.727934	-0.669549	1.362438
H	0.226467	1.083130	-0.491817
C	2.680357	1.720505	0.922032
H	3.632574	1.399413	1.373212
C	2.933450	2.065893	-0.547119
H	2.057495	2.579188	-0.993275
H	3.762280	2.783653	-0.650086
C	3.231478	0.833859	-1.329177
H	3.768023	0.932136	-2.276357
C	2.867002	-0.444268	-0.910838
H	2.344171	2.606348	1.479622
H	-2.637142	1.852360	-2.672468
C	1.607961	0.247374	2.616251

H	1.385472	1.162587	3.181930
H	0.849186	-0.494028	2.889674
H	2.582278	-0.127970	2.956214
C	3.329809	-1.636066	-1.731841
H	3.927572	-1.308376	-2.590085
H	3.961519	-2.305536	-1.129815
H	2.488501	-2.231296	-2.106808
H	2.431175	-2.708860	0.537148

$C^{+\bullet}$ (G781.072985)

C	3.239831	-1.129915	-0.444191
C	2.576649	-0.088519	0.255411
C	2.946657	1.271237	0.181645
C	-2.169408	2.631432	-0.529523
C	-1.490598	2.333675	-1.645411
C	4.631660	-1.057161	-1.005299
H	-1.285132	1.306253	-1.946626
H	-1.134447	3.121917	-2.308261
H	5.255771	-0.279310	-0.559783
H	4.617266	-0.906962	-2.099260
C	2.596674	-2.482950	-0.584767
H	3.020161	-3.020958	-1.442946
H	2.797689	-3.106858	0.304574
H	1.506248	-2.433884	-0.702420
C	-2.758868	1.654966	0.483039
C	1.341751	-0.456208	1.083485
C	-2.429296	0.196828	0.284100
H	-2.457538	1.992758	1.483935
C	0.038422	-0.036268	0.368936
C	-1.292666	-0.438418	1.093123
H	0.039212	-0.451189	-0.651012
H	-2.365862	3.680069	-0.286320
C	2.305082	2.322594	1.044737
H	1.797198	1.943100	1.935137
H	3.066587	3.052730	1.358119
H	1.570143	2.894951	0.452173
C	3.921345	1.852697	-0.807688
H	4.016700	1.277571	-1.731914
H	3.611648	2.874775	-1.066797
H	4.925272	1.949260	-0.359884
H	1.331840	-1.529827	1.296162
H	1.405591	0.030612	2.064463
H	0.039655	1.052658	0.237498
C	-1.479341	-1.976371	1.144509
H	-2.360643	-2.191307	1.769475
C	-1.702720	-2.596667	-0.236627
H	-0.763517	-2.601774	-0.828985
H	-1.983440	-3.658592	-0.154801
C	-2.742288	-1.853642	-1.001007
H	-3.232146	-2.357089	-1.838340
C	-3.116391	-0.541959	-0.711318
H	-0.622243	-2.446468	1.646917
H	5.128220	-2.026505	-0.848893
C	-1.272775	0.083395	2.553954

H	-0.605795	-0.539117	3.163672
H	-0.913561	1.118283	2.624528
H	-2.271198	0.032131	3.007753
C	-4.248512	0.090590	-1.502932
H	-4.605735	-0.588359	-2.285847
H	-5.104447	0.317787	-0.850663
H	-3.939425	1.029511	-1.978855
H	-3.855311	1.787824	0.452135

$C^{+\bullet}$-$D^{+\bullet}$-TS (G781.022278, T-658)

C	3.499635	-0.821880	-0.265331
C	2.448681	-0.106860	0.251560
C	2.348081	1.354967	0.126418
C	-0.907026	2.385905	0.549465
C	-1.030641	2.581946	-0.837876
C	4.749433	-0.195564	-0.840942
H	-1.255370	1.758803	-1.514922
H	-0.831815	3.557393	-1.280235
H	4.834784	0.881492	-0.670563
H	4.826952	-0.391859	-1.922367
C	3.565348	-2.328327	-0.196857
H	3.955064	-2.723621	-1.147132
H	4.280869	-2.646389	0.579083
H	2.607249	-2.816247	0.005944
C	-1.622173	1.205281	1.304473
C	1.287152	-0.777614	0.977432
C	-2.054134	0.033274	0.479511
H	-0.990153	0.910813	2.149306
C	0.144082	-1.120088	-0.012350
C	-1.302892	-1.300174	0.590298
H	0.420470	-2.049013	-0.533725
H	-0.938352	3.319102	1.131751
C	1.824090	2.137588	1.152235
H	1.741290	1.749643	2.168047
H	1.896775	3.224046	1.065006
H	0.327200	2.119072	0.709326
C	2.553345	2.025613	-1.207770
H	2.984907	1.368866	-1.966046
H	1.545248	2.318057	-1.570654
H	3.136924	2.953194	-1.134446
H	1.609325	-1.683430	1.503924
H	0.917800	-0.104466	1.757545
H	0.110293	-0.351980	-0.800587
C	-2.051848	-2.406731	-0.200925
H	-2.954718	-2.692159	0.361460
C	-2.492279	-1.955333	-1.596399
H	-1.617586	-1.766017	-2.248919
H	-3.069906	-2.742806	-2.102290
C	-3.311801	-0.712538	-1.474325
H	-4.089392	-0.509721	-2.214971
C	-3.122363	0.205794	-0.448383
H	-1.413888	-3.300283	-0.255667
H	5.631355	-0.671776	-0.385650
C	-1.259149	-1.775968	2.068094

H	-0.757010	-2.751216	2.128763
H	-0.728399	-1.092361	2.740112
H	-2.278507	-1.898809	2.455856
C	-4.027377	1.424262	-0.370073
H	-4.797638	1.387426	-1.149238
H	-4.540490	1.471920	0.601417
H	-3.478853	2.369311	-0.495808
H	-2.513408	1.681712	1.746142

D$^{+\bullet}$ (G781.045626)

C	3.499635	-0.821880	-0.265331
C	2.448681	-0.106860	0.251560
C	2.348081	1.354967	0.126418
C	-0.907026	2.385905	0.549465
C	-1.030641	2.581946	-0.837876
C	4.749433	-0.195564	-0.840942
H	-1.255370	1.758803	-1.514922
H	-0.831815	3.557393	-1.280235
H	4.834784	0.881492	-0.670563
H	4.826952	-0.391859	-1.922367
C	3.565348	-2.328327	-0.196857
H	3.955064	-2.723621	-1.147132
H	4.280869	-2.646389	0.579083
H	2.607249	-2.816247	0.005944
C	-1.622173	1.205281	1.304473
C	1.287152	-0.777614	0.977432
C	-2.054134	0.033274	0.479511
H	-0.990153	0.910813	2.149306
C	0.144082	-1.120088	-0.012350
C	-1.302892	-1.300174	0.590298
H	0.420470	-2.049013	-0.533725
H	-0.938352	3.319102	1.131751
C	1.824090	2.137588	1.152235
H	1.741290	1.749643	2.168047
H	1.896775	3.224046	1.065006
H	0.327200	2.119072	0.709326
C	2.553345	2.025613	-1.207770
H	2.984907	1.368866	-1.966046
H	1.545248	2.318057	-1.570654
H	3.136924	2.953194	-1.134446
H	1.609325	-1.683430	1.503924
H	0.917800	-0.104466	1.757545
H	0.110293	-0.351980	-0.800587
C	-2.051848	-2.406731	-0.200925
H	-2.954718	-2.692159	0.361460
C	-2.492279	-1.955333	-1.596399
H	-1.617586	-1.766017	-2.248919
H	-3.069906	-2.742806	-2.102290
C	-3.311801	-0.712538	-1.474325
H	-4.089392	-0.509721	-2.214971
C	-3.122363	0.205794	-0.448383
H	-1.413888	-3.300283	-0.255667
H	5.631355	-0.671776	-0.385650
C	-1.259149	-1.775968	2.068094

H	-0.757010	-2.751216	2.128763
H	-0.728399	-1.092361	2.740112
H	-2.278507	-1.898809	2.455856
C	-4.027377	1.424262	-0.370073
H	-4.797638	1.387426	-1.149238
H	-4.540490	1.471920	0.601417
H	-3.478853	2.369311	-0.495808
H	-2.513408	1.681712	1.746142

$D^{+\bullet}$ (G781.045624)

C	3.249206	-0.998302	-0.599215
C	2.536182	-0.163576	0.212985
C	2.838814	1.305323	0.329310
C	-1.930642	2.675607	-0.125823
C	-1.707205	2.639411	-1.578253
C	4.527203	-0.558640	-1.277025
H	-1.023812	1.916337	-2.023074
H	-2.238245	3.311146	-2.250315
H	4.913397	0.389518	-0.889299
H	4.385250	-0.464603	-2.366436
C	2.887605	-2.441460	-0.861902
H	2.901627	-2.635451	-1.946054
H	3.639244	-3.117470	-0.423586
H	1.903417	-2.738690	-0.483277
C	-2.875553	1.493185	0.456616
C	1.350563	-0.628539	1.036282
C	-2.381680	0.106567	0.279903
H	-2.964928	1.725743	1.524837
C	0.043656	-0.153294	0.351786
C	-1.285985	-0.448849	1.164961
H	-0.021967	-0.601849	-0.649246
H	-2.437112	3.601636	0.173523
C	3.164940	1.854570	1.516646
H	3.279761	1.248753	2.416938
H	3.362321	2.922840	1.613305
H	-0.983502	2.612526	0.428831
C	2.677517	2.153399	-0.917769
H	3.318465	1.806676	-1.739310
H	1.643090	2.094893	-1.294919
H	2.911411	3.207270	-0.721645
H	1.342830	-1.716640	1.168988
H	1.419385	-0.178033	2.034982
H	0.128136	0.929848	0.200697
C	-1.497225	-1.970822	1.366168
H	-2.416369	-2.115371	1.955796
C	-1.631040	-2.725692	0.041665
H	-0.643307	-2.889474	-0.434059
H	-2.029008	-3.742168	0.194240
C	-2.485956	-2.004651	-0.938070
H	-2.831919	-2.559800	-1.814861
C	-2.874418	-0.681405	-0.812530
H	-0.673722	-2.381462	1.964407
H	5.305370	-1.324293	-1.136706

C	-1.218048	0.235256	2.548269
H	-0.488673	-0.281370	3.182135
H	-0.907443	1.285875	2.483569
H	-2.186886	0.185391	3.063071
C	-3.814416	-0.079221	-1.841675
H	-3.957402	-0.764133	-2.685320
H	-4.805417	0.124658	-1.410828
H	-3.428113	0.869520	-2.240695
H	-3.856983	1.624977	-0.009655

$D^{+\bullet}$-$E1^{+\bullet}$-TS (G781.035988, T-547)

C	3.293177	-0.941837	-0.618619
C	2.540946	-0.152788	0.205943
C	2.789206	1.321026	0.360817
C	-1.884347	2.805683	-0.142881
C	-1.829273	2.785179	-1.550213
C	4.565112	-0.444739	-1.267272
H	-1.086559	2.188215	-2.079082
H	-2.569030	3.311219	-2.152766
H	4.917972	0.504281	-0.850914
H	4.435551	-0.328638	-2.356083
C	2.985549	-2.390454	-0.916767
H	3.066242	-2.570874	-2.000081
H	3.728998	-3.052066	-0.443270
H	1.989869	-2.716598	-0.597422
C	-2.943387	1.353359	0.572320
C	1.363218	-0.677673	1.006937
C	-2.403354	0.050291	0.318003
H	-2.884057	1.698594	1.604515
C	0.045190	-0.180128	0.367579
C	-1.270917	-0.521664	1.165108
H	-0.025764	-0.569466	-0.658299
H	-2.477165	3.607145	0.304272
C	3.057099	1.858370	1.569437
H	3.166756	1.239889	2.461466
H	3.215304	2.930618	1.691067
H	-0.955368	2.603272	0.392367
C	2.633363	2.191629	-0.871244
H	3.303053	1.884448	-1.685151
H	1.610043	2.105971	-1.273460
H	2.827853	3.247838	-0.646557
H	1.371176	-1.771367	1.078411
H	1.438712	-0.287629	2.031177
H	0.120425	0.911492	0.274408
C	-1.470112	-2.053438	1.297657
H	-2.394184	-2.233058	1.869735
C	-1.580449	-2.750938	-0.061137
H	-0.584704	-2.878853	-0.529406
H	-1.965336	-3.777788	0.048266
C	-2.443737	-1.999862	-1.016234
H	-2.778424	-2.522398	-1.916838
C	-2.859923	-0.697742	-0.830237
H	-0.648789	-2.482289	1.886808
H	5.364343	-1.189874	-1.134677

C	-1.206315	0.098182	2.577706
H	-0.468792	-0.436289	3.187231
H	-0.904963	1.154261	2.558412
H	-2.172666	0.017458	3.093184
C	-3.807690	-0.064682	-1.830012
H	-3.957343	-0.719240	-2.696033
H	-4.795708	0.126478	-1.386047
H	-3.426946	0.898495	-2.200423
H	-3.900632	1.580102	0.103840

E1$^{+\bullet}$ (G781.042329)

C	2.870661	-1.652754	-0.379516
C	2.288922	-0.558178	0.253417
C	2.838965	0.808865	0.124066
C	0.013594	3.551047	-1.096830
C	0.524089	3.792664	0.117614
C	4.258899	-1.612438	-0.960089
H	0.021406	3.469808	1.030685
H	1.451861	4.350762	0.249169
H	4.813221	-0.700144	-0.724304
H	4.228053	-1.742806	-2.054564
C	2.220161	-3.008842	-0.427546
H	2.424218	-3.483621	-1.398382
H	2.667458	-3.673005	0.331848
H	1.137891	-2.991352	-0.271770
C	-2.878228	1.740964	0.209393
C	1.038909	-0.706256	1.093138
C	-2.567705	0.410232	0.114434
H	-2.445233	2.380075	0.975270
C	-0.180575	-0.153885	0.306505
C	-1.548560	-0.240748	1.066153
H	-0.258897	-0.671838	-0.661955
H	0.507018	3.897709	-2.006233
C	2.987253	1.600581	1.224415
H	2.756789	1.252862	2.230577
H	3.411233	2.600350	1.135679
H	-0.927944	3.014010	-1.227325
C	3.132793	1.364445	-1.254359
H	3.382821	0.595046	-1.990617
H	2.218583	1.865847	-1.610840
H	3.932334	2.114551	-1.232074
H	0.875673	-1.746402	1.395759
H	1.156134	-0.121282	2.014724
H	0.019495	0.901156	0.070478
C	-1.985168	-1.701647	1.342723
H	-2.933251	-1.668437	1.902571
C	-2.195534	-2.520664	0.065823
H	-1.229756	-2.841924	-0.370458
H	-2.722297	-3.462666	0.289615
C	-2.950365	-1.764190	-0.978377
H	-3.364685	-2.332093	-1.815942
C	-3.155131	-0.406638	-0.954032
H	-1.256677	-2.195534	2.001395
H	4.832387	-2.471265	-0.576969

C	-1.448026	0.499706	2.418055
H	-0.845246	-0.082979	3.125934
H	-0.974057	1.485721	2.321144
H	-2.439827	0.636727	2.867020
C	-3.974930	0.247918	-2.044465
H	-4.288809	-0.486191	-2.794911
H	-4.884539	0.713306	-1.636528
H	-3.407963	1.039103	-2.555868
H	-3.597545	2.213412	-0.456249

E1$^{+\bullet}$ (G781.045729)

C	2.872536	1.315724	0.098882
C	2.332198	0.073356	-0.232231
C	2.936399	-1.199182	0.209630
C	4.270270	1.492867	0.628873
H	4.935235	0.653892	0.405935
H	4.254940	1.640490	1.721959
C	2.139033	2.608038	-0.137366
H	2.348341	3.305458	0.687119
H	2.519748	3.099590	-1.049453
H	1.055072	2.500925	-0.232138
C	-2.746824	-1.971147	0.965261
C	1.042997	-0.015653	-1.021661
C	-2.526922	-0.762776	0.358030
H	-2.236186	-2.876127	0.643356
C	-0.149789	-0.300510	-0.068169
C	-1.504752	-0.627641	-0.784725
H	-0.280345	0.552503	0.615687
C	2.893706	-2.306042	-0.588529
H	2.497248	-2.289623	-1.601957
H	3.320665	-3.249717	-0.247765
C	3.557214	-1.317187	1.594581
H	3.157449	-0.569094	2.288314
H	3.349980	-2.312758	2.007131
H	4.649774	-1.204050	1.570934
H	0.862632	0.900196	-1.595525
H	1.115557	-0.830716	-1.751969
H	0.113095	-1.160767	0.565789
C	-1.961261	0.498617	-1.744675
H	-2.892702	0.168902	-2.231635
C	-2.223651	1.827181	-1.031028
H	-1.276183	2.336651	-0.766999
H	-2.739186	2.535411	-1.699793
C	-3.024745	1.646379	0.216722
H	-3.499714	2.531080	0.649532
C	-3.201643	0.441665	0.854447
H	-1.223358	0.631175	-2.548322
H	4.710523	2.407041	0.206265
C	-1.363035	-1.931630	-1.603756
H	-0.767269	-1.750497	-2.507522
H	-0.863994	-2.729419	-1.037905
H	-2.344115	-2.300559	-1.928428
C	-4.073886	0.369823	2.088779
H	-4.472148	1.357576	2.346100

H	-4.930062	-0.303240	1.933975
H	-3.515935	-0.009772	2.956750
H	-3.456148	-2.085834	1.782180

E1$^{+\bullet}$-E2$^{+\bullet}$-TS (G781.037931, T-82)

C	3.200438	-0.865755	-0.175717
C	2.353907	0.133986	0.302537
C	2.574643	1.565889	0.021713
C	4.564749	-0.589165	-0.746943
H	4.980690	0.376688	-0.446935
H	4.538782	-0.626643	-1.849222
C	2.859929	-2.328157	-0.067250
H	3.272724	-2.870876	-0.929613
H	3.346718	-2.762937	0.823374
H	1.788201	-2.539988	-0.002769
C	-2.695288	1.365835	1.527356
C	1.107149	-0.235254	1.082721
C	-2.500001	0.434081	0.541451
H	-2.176555	1.309572	2.481697
C	-0.113019	-0.220075	0.125050
C	-1.497542	-0.718939	0.728975
H	0.136620	-0.830926	-0.755808
C	2.296916	2.508589	0.969026
H	1.980377	2.253768	1.979083
H	2.447928	3.567648	0.757627
C	3.063789	2.017241	-1.347166
H	2.847598	1.275614	-2.124308
H	2.569910	2.957849	-1.622683
H	4.145242	2.210844	-1.358440
H	1.218406	-1.213139	1.561700
H	0.937318	0.491297	1.888238
H	-0.213913	0.807595	-0.251834
C	-1.992988	-1.960217	-0.061408
H	-2.906279	-2.336231	0.426838
C	-2.309070	-1.664544	-1.532157
H	-1.384167	-1.589507	-2.137353
H	-2.866402	-2.499409	-1.986464
C	-3.079765	-0.398575	-1.703548
H	-3.580159	-0.235684	-2.662116
C	-3.199255	0.574392	-0.740385
H	-1.241423	-2.760429	0.014909
H	5.261378	-1.379817	-0.434074
C	-1.390000	-1.144052	2.211384
H	-0.775781	-2.048880	2.305270
H	-0.945149	-0.379624	2.859434
H	-2.385612	-1.379482	2.608010
C	-4.044227	1.799012	-1.017301
H	-4.461040	1.766844	-2.030085
H	-4.887178	1.871255	-0.314221
H	-3.459650	2.725019	-0.918757
H	-3.390759	2.193834	1.408476

E2$^{+\bullet}$ (G781.049131)

C	3.348435	0.552596	-0.130090

C	2.069872	-0.013196	-0.211983
C	1.808904	-1.443227	0.014589
C	4.622697	-0.243983	-0.153366
H	4.513524	-1.252037	-0.560537
H	5.052231	-0.321099	0.859939
C	3.554658	2.043714	-0.100837
H	4.494180	2.282963	0.414919
H	3.660955	2.433703	-1.128722
H	2.738659	2.592321	0.381589
C	-2.174052	0.616325	-2.131964
C	0.871782	0.861949	-0.515431
C	-2.105667	0.302031	-0.803920
H	-1.925069	1.608404	-2.501070
C	-0.134676	0.912408	0.656236
C	-1.594760	1.296790	0.252486
H	0.213855	1.632653	1.413292
C	0.722142	-2.043792	-0.567266
H	0.088840	-1.528263	-1.282071
H	0.501118	-3.093978	-0.377646
C	2.658094	-2.265555	0.971664
H	3.145622	-1.645923	1.731864
H	2.024692	-2.999628	1.485674
H	3.439121	-2.834336	0.448094
H	1.180443	1.869837	-0.807419
H	0.352798	0.452982	-1.402241
H	-0.138005	-0.070665	1.140644
C	-2.501649	1.163358	1.506699
H	-3.523921	1.464339	1.227952
C	-2.529410	-0.267577	2.064899
H	-1.641400	-0.468832	2.694484
H	-3.381823	-0.405060	2.751148
C	-2.602875	-1.307348	0.995396
H	-2.828341	-2.332891	1.301514
C	-2.469618	-1.046700	-0.350515
H	-2.162414	1.866107	2.281293
H	5.371095	0.294097	-0.753249
C	-1.636592	2.758978	-0.233807
H	-1.323137	3.424725	0.581401
H	-0.963835	2.943349	-1.081435
H	-2.650583	3.050453	-0.537813
C	-2.766621	-2.140788	-1.353502
H	-2.893827	-3.108105	-0.854272
H	-3.699158	-1.925506	-1.896498
H	-1.975968	-2.244897	-2.109329
H	-2.513637	-0.100604	-2.877230

E2$^{+\bullet}$ (G781.047669)

C	-3.370194	0.500210	0.161550
C	-2.059655	0.023462	0.134666
C	-1.734048	-1.402199	-0.096895
C	-4.568528	-0.400736	0.274437
H	-4.325154	-1.447058	0.477055
H	-5.190564	-0.339749	-0.634029
C	-3.686514	1.971936	0.207184

H	-4.701727	2.155563	-0.167500
H	-3.669937	2.334769	1.249884
H	-2.983240	2.584944	-0.367643
C	2.082300	0.834601	2.109526
C	-0.883514	0.939386	0.374594
C	2.049685	0.385460	0.818317
H	1.857598	1.867589	2.364183
C	0.152687	0.894796	-0.770160
C	1.608917	1.279901	-0.352651
H	-0.162961	1.565544	-1.584330
C	-0.784235	-2.019484	0.664054
H	-0.303719	-1.515990	1.499048
H	-0.533311	-3.067802	0.504631
C	-2.345285	-2.125548	-1.280226
H	-3.389717	-1.858803	-1.467728
H	-1.789482	-1.836129	-2.187138
H	-2.271180	-3.214054	-1.173604
H	-1.204298	1.965373	0.580115
H	-0.377576	0.602859	1.303223
H	0.155728	-0.119643	-1.184669
C	2.555534	0.994124	-1.550527
H	3.575569	1.297357	-1.266252
C	2.562328	-0.487792	-1.956767
H	1.698829	-0.730399	-2.605684
H	3.440209	-0.719157	-2.583319
C	2.555207	-1.414049	-0.785610
H	2.754676	-2.472289	-0.976998
C	2.382637	-1.012215	0.521456
H	2.264133	1.620117	-2.406142
H	-5.208834	-0.032939	1.092149
C	1.676213	2.782302	-0.013826
H	1.417612	3.369796	-0.904721
H	0.973400	3.068410	0.779587
H	2.684787	3.077183	0.304902
C	2.615350	-2.003703	1.641450
H	2.709488	-3.023234	1.250613
H	3.548149	-1.769044	2.176036
H	1.809746	-1.994116	2.387711
H	2.368469	0.188197	2.937182

E2$^{+\bullet}$-F$^{+\bullet}$-TS (G781.037719, T-1389)

C	-3.273000	0.452597	0.252923
C	-2.000617	0.058461	-0.162263
C	-1.710038	-1.381180	-0.501726
C	-4.392154	-0.534182	0.404509
H	-4.085335	-1.574449	0.263044
H	-5.204471	-0.296583	-0.302865
C	-3.600905	1.883770	0.606502
H	-4.673821	2.005469	0.791885
H	-3.072035	2.197385	1.521252
H	-3.312941	2.585355	-0.189109
C	0.839032	0.981623	1.831949
C	-0.902809	1.003451	-0.223125
C	1.739687	0.479198	0.857759

H	0.739605	2.061578	1.954053
C	0.300317	0.741303	-1.107544
C	1.697806	1.177981	-0.492059
H	0.177314	1.311598	-2.041644
C	-1.055216	-2.167101	0.371026
H	-0.757827	-1.801459	1.354320
H	-0.823011	-3.206514	0.137364
C	-2.146614	-1.849254	-1.872315
H	-3.229111	-1.716378	-2.014156
H	-1.658430	-1.253841	-2.659923
H	-1.902836	-2.906482	-2.032940
H	-1.183172	2.059131	-0.146114
H	-0.314631	0.866020	1.000797
H	0.340529	-0.323021	-1.363294
C	2.850442	0.712084	-1.410157
H	3.782180	1.166175	-1.037431
C	3.027458	-0.809918	-1.444472
H	2.260098	-1.292453	-2.080939
H	3.985385	-1.079448	-1.915974
C	2.948595	-1.419996	-0.085138
H	3.361491	-2.423774	0.048388
C	2.358982	-0.813854	1.004047
H	2.691950	1.103244	-2.425188
H	-4.837778	-0.433965	1.407612
C	1.761757	2.719612	-0.379281
H	1.799688	3.154683	-1.386631
H	0.894227	3.157191	0.128946
H	2.664400	3.035906	0.159626
C	2.341706	-1.517346	2.346926
H	2.936278	-2.437191	2.315087
H	2.751134	-0.876643	3.139623
H	1.320884	-1.796498	2.647562
H	0.643648	0.411473	2.741275

$F^{+\bullet}$ (G781.052350)

C	-3.295558	0.396212	0.226323
C	-2.006065	0.078777	-0.257231
C	-1.645755	-1.355493	-0.572440
C	-4.334599	-0.659799	0.433972
H	-4.016081	-1.662724	0.136833
H	-5.257779	-0.389909	-0.105558
C	-3.710291	1.807264	0.556356
H	-4.665686	1.819684	1.092501
H	-2.965897	2.329955	1.173309
H	-3.841145	2.401347	-0.362763
C	0.811483	0.468705	2.098025
C	-1.064431	1.086441	-0.506982
C	1.667834	0.246043	0.902090
H	1.146487	1.352268	2.664625
C	0.270730	0.860971	-1.095317
C	1.569678	1.260533	-0.202370
H	0.370489	1.528283	-1.967658
C	-1.111461	-2.153363	0.367805
H	-0.954608	-1.803847	1.388300

H	-0.846093	-3.188355	0.149449
C	-1.924465	-1.812062	-1.986386
H	-2.998825	-1.733760	-2.214227
H	-1.411221	-1.171125	-2.720120
H	-1.611713	-2.850675	-2.148006
H	-1.340809	2.124062	-0.312708
H	-0.213564	0.717738	1.758329
H	0.391621	-0.175148	-1.427434
C	2.814950	1.203956	-1.121135
H	3.661926	1.619933	-0.552612
C	3.179003	-0.215075	-1.569091
H	2.510413	-0.573829	-2.375761
H	4.185355	-0.236617	-2.016329
C	3.113230	-1.184413	-0.437715
H	3.616149	-2.147621	-0.558587
C	2.431695	-0.948080	0.746576
H	2.663299	1.860819	-1.989375
H	-4.620777	-0.690588	1.499673
C	1.403617	2.710249	0.306137
H	1.324215	3.393135	-0.550388
H	0.514236	2.851236	0.929789
H	2.280073	3.012511	0.894473
C	2.473438	-1.991519	1.848488
H	3.160292	-2.804728	1.587871
H	2.814301	-1.560408	2.799497
H	1.483332	-2.435791	2.022508
H	0.763805	-0.381926	2.781744

F$^{+\bullet}$ (G781.052453)

C	-3.294857	0.395075	0.222606
C	-2.003158	0.087025	-0.259657
C	-1.638903	-1.346641	-0.576022
C	-4.328819	-0.672063	0.412346
H	-4.032398	-1.653032	0.031171
H	-5.280315	-0.365673	-0.051312
C	-3.742694	1.789195	0.567173
H	-4.504393	1.762372	1.357155
H	-2.932916	2.451503	0.892404
H	-4.220797	2.255578	-0.311332
C	0.808542	0.461747	2.100920
C	-1.060741	1.094645	-0.511428
C	1.666137	0.242977	0.904832
H	1.146910	1.340300	2.673415
C	0.275413	0.866250	-1.095424
C	1.571591	1.262449	-0.195077
H	0.380837	1.534231	-1.966481
C	-1.122082	-2.148774	0.370262
H	-0.982055	-1.803173	1.394565
H	-0.853706	-3.183089	0.152398
C	-1.893552	-1.797114	-1.996610
H	-2.963237	-1.714275	-2.243947
H	-1.365539	-1.155772	-2.719350
H	-1.581304	-2.836189	-2.156053
H	-1.335600	2.133036	-0.322603

H	-0.215038	0.716798	1.762599
H	0.397287	-0.169685	-1.427397
C	2.819742	1.209650	-1.110265
H	3.665006	1.622106	-0.536636
C	3.184419	-0.207316	-1.564324
H	2.518404	-0.561214	-2.375246
H	4.192325	-0.226998	-2.008122
C	3.114113	-1.182401	-0.437976
H	3.616536	-2.145439	-0.562110
C	2.429042	-0.951221	0.745281
H	2.671486	1.871178	-1.975558
H	-4.549407	-0.780900	1.488711
C	1.403635	2.710015	0.318626
H	1.326104	3.396386	-0.535315
H	0.512739	2.848154	0.940737
H	2.278607	3.010142	0.910213
C	2.465878	-2.000403	1.841889
H	3.151846	-2.813615	1.578948
H	2.804918	-1.574934	2.796092
H	1.474321	-2.443463	2.010779
H	0.757150	-0.392887	2.779372

$F^{+\bullet}$-$G^{+\bullet}$-TS (G781.042215, T-398)

C	-3.242952	0.449126	0.208164
C	-1.999331	0.107476	-0.340230
C	-1.661986	-1.338646	-0.621063
C	-4.282104	-0.595165	0.497996
H	-4.064102	-1.571390	0.056041
H	-5.271719	-0.248937	0.162652
C	-3.657656	1.853966	0.553281
H	-4.199969	1.857489	1.509832
H	-2.834616	2.570945	0.626846
H	-4.370878	2.227110	-0.200556
C	0.705382	0.333856	2.096507
C	-1.072410	1.106962	-0.742211
C	1.631732	0.195610	0.928274
H	1.050856	1.130417	2.774704
C	0.214854	0.846002	-1.283423
C	1.715274	1.292403	-0.011738
H	0.569097	1.582789	-2.007735
C	-1.242143	-2.156823	0.359164
H	-1.157131	-1.812975	1.389758
H	-1.005602	-3.202889	0.161394
C	-1.865300	-1.800478	-2.047739
H	-2.930783	-1.737137	-2.319172
H	-1.331398	-1.157834	-2.763516
H	-1.535617	-2.836688	-2.189566
H	-1.360708	2.154543	-0.650136
H	-0.291325	0.648246	1.744217
H	0.451740	-0.187143	-1.535289
C	2.943071	1.287345	-0.922572
H	3.780729	1.662395	-0.306895
C	3.308169	-0.099531	-1.463923
H	2.682396	-0.364343	-2.337510

H	4.339462	-0.103822	-1.848337
C	3.148897	-1.163026	-0.425587
H	3.650688	-2.119471	-0.592496
C	2.379171	-1.019012	0.709540
H	2.817634	2.010561	-1.739852
H	-4.369560	-0.735796	1.589297
C	1.355679	2.690502	0.476774
H	1.289047	3.387191	-0.368392
H	0.411769	2.729606	1.028768
H	2.150435	3.058832	1.143676
C	2.302497	-2.155810	1.709020
H	2.978405	-2.969927	1.423976
H	2.584016	-1.826680	2.718868
H	1.286515	-2.570737	1.768629
H	0.595556	-0.582897	2.679377

G$^{+\bullet}$ (G781.042248)

C	3.126682	0.661866	-0.240993
C	2.055310	0.105416	0.448790
C	1.912999	-1.388213	0.590896
C	4.210144	-0.190048	-0.843212
H	4.193603	-1.228447	-0.501363
H	5.195680	0.251822	-0.630830
C	3.321545	2.141094	-0.452978
H	3.607347	2.329870	-1.498575
H	2.449274	2.760104	-0.224803
H	4.166614	2.497379	0.158656
C	-0.608415	0.372432	-1.984145
C	1.087339	0.941534	1.131131
C	-1.671867	0.215042	-0.928209
H	-0.827792	1.222191	-2.645814
C	-0.076650	0.478565	1.678409
C	-2.025955	1.313872	-0.134256
H	-0.697877	1.134446	2.285343
C	1.563093	-2.160346	-0.453908
H	1.396078	-1.736694	-1.444039
H	1.470829	-3.242291	-0.352532
C	2.228919	-1.961891	1.956957
H	3.294082	-1.808620	2.190968
H	1.662834	-1.458876	2.753278
H	2.020275	-3.037572	1.999833
H	1.302190	2.006488	1.226232
H	0.365881	0.578951	-1.512574
H	-0.354230	-0.572268	1.624460
C	-3.210549	1.234868	0.793634
H	-4.079269	1.598167	0.208076
C	-3.510233	-0.175580	1.317140
H	-2.891880	-0.388286	2.211549
H	-4.549991	-0.248017	1.667457
C	-3.228351	-1.233896	0.302669
H	-3.696683	-2.212061	0.435222
C	-2.364064	-1.059256	-0.751951
H	-3.098892	1.949211	1.621078
H	4.116098	-0.195861	-1.942424

C	-1.456099	2.683879	-0.369325
H	-1.425702	3.263362	0.561995
H	-0.455647	2.671142	-0.810847
H	-2.116413	3.236256	-1.060966
C	-2.099621	-2.202988	-1.703295
H	-2.736169	-3.063234	-1.468062
H	-2.290074	-1.916884	-2.747531
H	-1.051763	-2.531892	-1.640170
H	-0.493765	-0.516477	-2.607751

Structures from Scheme 1 of main text (Table S11):

A (G781.676118)

C	-2.641579	-0.599311	-0.438297
C	-2.263527	0.564039	-1.018977
C	0.650467	-2.369851	0.875468
C	-0.103572	-3.041231	-0.068115
C	-1.608909	-3.018574	-0.203995
C	-2.315536	-1.935502	-1.088220
H	-1.885656	-3.998960	-0.615281
H	-2.045131	-2.975111	0.804921
H	-1.770217	-1.791116	-2.032358
H	-3.278603	-2.388130	-1.378615
C	-3.576251	-0.682580	0.751079
H	-3.755486	0.285785	1.227901
H	-4.553150	-1.085967	0.440190
H	-3.196235	-1.363076	1.527876
C	1.094304	-1.335820	-1.320071
C	-2.730743	1.954766	-0.664821
C	1.498969	-0.104378	-0.766909
H	1.816342	-2.080245	-1.649545
C	-1.621333	3.036974	-0.662755
C	-0.488768	2.761139	0.307693
H	-2.101279	3.996183	-0.401796
C	2.064588	-2.773661	1.180956
H	2.102355	-3.198500	2.196216
H	2.461530	-3.519982	0.482985
H	2.721607	-1.891519	1.191984
C	0.063711	-1.310530	1.762259
H	-0.180174	-1.750891	2.742619
H	0.799936	-0.516793	1.949612
H	-0.837996	-0.859081	1.341061
H	-3.481776	2.258916	-1.415056
H	-3.258281	1.965277	0.298006
H	-1.218042	3.154654	-1.680451
C	0.790302	2.793054	-0.123275
H	0.960512	3.051079	-1.175492
C	2.038484	2.486234	0.669607
H	2.513578	3.424578	1.004010
H	1.801961	1.920769	1.582749
C	3.112999	1.718222	-0.134581
H	3.352971	2.287908	-1.054829
H	4.058849	1.703363	0.430873
C	2.809096	0.307314	-0.580163
H	-1.627407	0.503537	-1.912135
H	0.706137	0.606801	-0.537006
C	-0.896816	2.469395	1.733668
H	-1.570706	3.257717	2.102873
H	-1.459421	1.525744	1.801983
H	-0.050787	2.410666	2.426931
C	4.017209	-0.520879	-0.918778
H	4.595844	-0.014105	-1.707962
H	4.689296	-0.582909	-0.050206
H	3.793905	-1.536119	-1.258454

H	0.121093	-1.374430	-1.797451
H	0.415849	-3.818961	-0.636782

A-B1-TS (G781.675917, T-34)

C	-2.605164	-0.826845	-0.514130
C	-2.363533	0.443201	-0.903842
C	0.677560	-2.113882	1.068009
C	0.164625	-2.954821	0.094781
C	-1.252759	-3.093800	-0.436520
C	-1.965855	-1.981258	-1.263475
H	-1.247472	-3.997430	-1.060879
H	-1.888275	-3.343469	0.429879
H	-1.294789	-1.594925	-2.044394
H	-2.772528	-2.509364	-1.800787
C	-3.597189	-1.208700	0.563321
H	-4.123719	-0.345801	0.982650
H	-4.356801	-1.894367	0.156009
H	-3.118376	-1.741827	1.400721
C	1.490594	-1.609107	-1.286600
C	-2.974127	1.708518	-0.357873
C	1.631300	-0.217862	-1.076528
H	2.347294	-2.272637	-1.195258
C	-1.993887	2.906112	-0.414142
C	-0.698789	2.689036	0.348328
H	-2.510524	3.790522	-0.004429
C	2.000983	-2.425553	1.712130
H	1.821923	-2.731732	2.755073
H	2.548452	-3.233434	1.213101
H	2.630802	-1.526518	1.761362
C	-0.001257	-0.889164	1.573948
H	-0.041035	-0.900232	2.673098
H	0.616030	-0.015251	1.299890
H	-0.994077	-0.725021	1.155146
H	-3.864607	1.976163	-0.953269
H	-3.333314	1.564369	0.670906
H	-1.767400	3.133063	-1.467139
C	0.481342	2.731844	-0.308943
H	0.442145	2.935921	-1.386131
C	1.871720	2.637047	0.261725
H	2.270997	3.656213	0.404092
H	1.883225	2.165926	1.255611
C	2.892531	1.914913	-0.671158
H	2.733327	2.284227	-1.699092
H	3.910984	2.212374	-0.388610
C	2.806784	0.407371	-0.687199
H	-1.666249	0.588005	-1.738900
H	0.767758	0.412600	-1.293271
C	-0.840102	2.504073	1.840993
H	-1.278441	3.409050	2.290357
H	-1.528245	1.679612	2.079056
H	0.109242	2.311038	2.352901
C	4.084719	-0.309822	-0.362427
H	4.850364	-0.027789	-1.103843
H	4.470855	0.041008	0.606585

H	4.014838	-1.400646	-0.337019
H	0.716430	-1.943444	-1.972001
H	0.794219	-3.824006	-0.123801

B1 (G781.691726)

C	0.218923	-2.512199	-0.628233
C	-1.044457	-2.054549	-0.793457
C	2.078232	0.024520	1.208123
C	2.752094	-0.157833	-0.096800
C	2.628905	-1.518882	-0.848809
C	1.327655	-1.998159	-1.531723
H	3.393576	-1.449559	-1.637242
H	2.986353	-2.297707	-0.158894
H	0.938604	-1.222266	-2.206063
H	1.640910	-2.831322	-2.185326
C	0.601311	-3.576788	0.373901
H	-0.266279	-4.016922	0.875991
H	1.152727	-4.390560	-0.121413
H	1.273108	-3.180462	1.154597
C	2.407590	1.131428	-1.028533
C	-2.276107	-2.432436	-0.009860
C	0.988840	1.498578	-1.301281
H	2.953284	1.976538	-0.592994
C	-3.353596	-1.322252	-0.092541
C	-2.868742	0.032289	0.402448
H	-4.230348	-1.639316	0.494577
C	2.905985	0.437738	2.372772
H	3.424374	-0.463555	2.753044
H	3.711513	1.125604	2.074396
H	2.320682	0.859979	3.196439
C	0.648268	-0.062915	1.318044
H	0.241941	0.089101	2.320380
H	0.245699	0.716213	0.601197
H	0.214635	-0.939761	0.792131
H	-2.705907	-3.370331	-0.402164
H	-2.022576	-2.635394	1.042324
H	-3.685416	-1.234545	-1.137842
C	-2.607836	1.018176	-0.485090
H	-2.776671	0.785094	-1.543840
C	-2.212850	2.447162	-0.227090
H	-3.086355	3.090226	-0.428185
H	-1.945261	2.622007	0.825209
C	-1.065023	2.936239	-1.164444
H	-1.255906	2.565465	-2.181936
H	-1.098538	4.036092	-1.208915
C	0.317554	2.532571	-0.709498
H	-1.199962	-1.303027	-1.574791
H	0.466774	0.946346	-2.085685
C	-2.761697	0.176045	1.902494
H	-3.765600	0.177293	2.355462
H	-2.229799	-0.679843	2.346577
H	-2.256136	1.095444	2.220660
C	0.916959	3.378921	0.389597
H	1.389928	4.275226	-0.042780

H	0.139038	3.735391	1.077235
H	1.682217	2.858690	0.978678
H	2.903980	0.876548	-1.976767
H	3.833360	-0.042617	0.075822

B2 (G781.714954)

C	-1.958298	0.841462	-1.070901
C	-0.874836	0.978557	-0.194460
C	-1.718610	-0.382489	1.165188
C	-1.836369	-1.692911	0.384714
C	-2.643838	-1.553133	-0.920421
C	-2.091786	-0.425481	-1.851992
H	-2.632565	-2.502379	-1.472545
H	-3.697371	-1.334130	-0.697352
H	-1.115627	-0.729096	-2.245456
H	-2.786493	-0.294088	-2.691949
C	-3.047312	1.854197	-1.214596
H	-3.132303	2.543650	-0.369731
H	-2.825418	2.460202	-2.111415
H	-4.016219	1.372934	-1.404591
C	-0.545186	-2.565805	0.254846
C	-0.471018	2.282726	0.457516
C	0.590801	-2.049425	-0.593322
H	-0.192363	-2.810884	1.264574
C	0.822570	2.832330	-0.222296
C	2.016725	1.915388	-0.008460
H	1.020804	3.828718	0.202556
C	-0.638791	-0.340902	2.226071
H	-0.685347	0.576360	2.821311
H	-0.832046	-1.179009	2.916240
H	0.367184	-0.470588	1.821381
C	-3.020364	0.234013	1.640498
H	-3.333201	-0.297279	2.553839
H	-2.882029	1.285331	1.920709
H	-3.839074	0.164160	0.918175
H	-1.266029	3.036356	0.394181
H	-0.261089	2.138580	1.525322
H	0.631040	2.970450	-1.296784
C	2.407041	1.083980	-0.998445
H	1.853498	1.146906	-1.944897
C	3.482541	0.033345	-0.981218
H	4.220630	0.246864	-1.771636
H	4.038920	0.028931	-0.034451
C	2.892129	-1.378636	-1.260388
H	2.433718	-1.381217	-2.261394
H	3.725024	-2.100901	-1.288327
C	1.875666	-1.830914	-0.227315
H	-0.035187	0.294095	-0.378703
H	0.373095	-1.935770	-1.659838
C	2.665297	2.001802	1.354270
H	3.056980	3.016652	1.525707
H	1.940711	1.812418	2.162652
H	3.496925	1.301463	1.482783
C	2.423164	-2.029122	1.167635

H	3.320371	-2.664923	1.130800
H	2.743369	-1.073255	1.609451
H	1.714373	-2.497529	1.858804
H	-0.888490	-3.521458	-0.176879
H	-2.492936	-2.285459	1.055911

B2-C-TS (G781.713618, T-104)

C	-2.053019	0.792759	-1.038001
C	-0.940119	0.980330	-0.211402
C	-1.646496	-0.453640	1.169037
C	-1.750892	-1.752857	0.369372
C	-2.624969	-1.625063	-0.893557
C	-2.153562	-0.468843	-1.832638
H	-2.609622	-2.567152	-1.457563
H	-3.673022	-1.443435	-0.616941
H	-1.180488	-0.726412	-2.264742
H	-2.882621	-0.359433	-2.646322
C	-3.205957	1.741282	-1.109158
H	-3.296956	2.399421	-0.240148
H	-3.057903	2.385492	-1.994364
H	-4.151508	1.207502	-1.274544
C	-0.433700	-2.571479	0.167181
C	-0.581095	2.295195	0.445254
C	0.686388	-1.963276	-0.641106
H	-0.067727	-2.876063	1.155869
C	0.692106	2.885617	-0.238552
C	1.906070	2.005370	-0.010199
H	0.857839	3.891003	0.178768
C	-0.505934	-0.381864	2.160552
H	-0.542258	0.529394	2.765245
H	-0.629030	-1.230728	2.853862
H	0.474603	-0.477854	1.689311
C	-2.942257	0.101709	1.725824
H	-3.181233	-0.455378	2.646169
H	-2.831167	1.153569	2.016072
H	-3.796731	0.008221	1.049044
H	-1.402267	3.020545	0.384450
H	-0.361842	2.158254	1.513062
H	0.498779	3.007941	-1.314760
C	2.361277	1.208284	-0.999085
H	1.853729	1.264098	-1.970663
C	3.479879	0.207380	-0.921528
H	4.261891	0.468264	-1.653901
H	3.963725	0.219076	0.064891
C	2.984594	-1.229001	-1.260260
H	2.550476	-1.222075	-2.271941
H	3.861172	-1.896678	-1.293198
C	1.975584	-1.782137	-0.268900
H	-0.071822	0.347040	-0.439399
H	0.456000	-1.738986	-1.687143
C	2.519436	2.039252	1.381493
H	3.585236	2.303471	1.343519
H	2.024043	2.783575	2.018589
H	2.449517	1.070309	1.897189

C	2.540637	-2.135570	1.088227
H	3.318917	-2.906038	0.975238
H	3.035024	-1.269766	1.553041
H	1.798423	-2.518854	1.796082
H	-0.748311	-3.506238	-0.327798
H	-2.352697	-2.382776	1.057685

C (G781.726425)

C	-1.733554	0.904811	-1.127392
C	-0.970843	0.784773	0.105559
C	-1.657298	-0.277684	1.105677
C	-1.892773	-1.636597	0.378038
C	-2.690842	-1.457948	-0.924552
C	-2.151404	-0.326708	-1.835265
H	-2.702164	-2.394452	-1.498404
H	-3.739616	-1.220835	-0.697780
H	-1.212792	-0.676831	-2.314073
H	-2.844620	-0.098629	-2.656106
C	-2.122593	2.191362	-1.750461
H	-1.631650	3.078278	-1.349298
H	-2.014565	2.148129	-2.844725
H	-3.213057	2.295014	-1.580133
C	-0.633682	-2.544119	0.197494
C	-0.523464	2.113025	0.754549
C	0.479981	-2.013271	-0.663686
H	-0.256962	-2.821019	1.188845
C	0.718507	2.733610	0.065917
C	1.926643	1.818004	0.158411
H	0.930466	3.699173	0.551357
C	-0.758662	-0.489168	2.341494
H	-0.797616	0.368400	3.022962
H	-1.117824	-1.359081	2.907904
H	0.286800	-0.666161	2.069939
C	-3.006001	0.323966	1.568988
H	-3.498020	-0.349104	2.283775
H	-2.854852	1.286383	2.074352
H	-3.699251	0.493453	0.732878
H	-1.350886	2.836491	0.769362
H	-0.277331	1.929881	1.805520
H	0.503215	2.950258	-0.990886
C	2.300606	1.098935	-0.925133
H	1.724051	1.255299	-1.846933
C	3.377261	0.059779	-1.035490
H	4.067484	0.321764	-1.853309
H	3.984158	-0.004673	-0.123173
C	2.768110	-1.336322	-1.370462
H	2.280103	-1.285511	-2.355799
H	3.597421	-2.057599	-1.456974
C	1.785154	-1.825894	-0.329097
H	-0.037509	0.213626	-0.220975
H	0.242166	-1.877667	-1.724750
C	2.613596	1.774488	1.503311
H	3.491963	1.121556	1.520122
H	2.945361	2.784804	1.788470

H	1.929936	1.438712	2.298316
C	2.367172	-2.098453	1.035971
H	3.262276	-2.731040	0.940649
H	2.699794	-1.166679	1.516857
H	1.673486	-2.598973	1.718403
H	-1.001094	-3.480070	-0.257676
H	-2.544349	-2.212434	1.058074

C (G781.726430)

C	-1.733869	0.904567	-1.127435
C	-0.971028	0.784772	0.105485
C	-1.656913	-0.277685	1.105798
C	-1.892526	-1.636674	0.378324
C	-2.691042	-1.458205	-0.924018
C	-2.151965	-0.327041	-1.834999
H	-2.702499	-2.394778	-1.497752
H	-3.739754	-1.221124	-0.696922
H	-1.213521	-0.677156	-2.314160
H	-2.845448	-0.099029	-2.655642
C	-2.122803	2.191014	-1.750775
H	-1.631372	3.077916	-1.350182
H	-2.015479	2.147393	-2.845088
H	-3.213134	2.295062	-1.579816
C	-0.633430	-2.544144	0.197460
C	-0.523834	2.113208	0.754270
C	0.480098	-2.013148	-0.663780
H	-0.256568	-2.821135	1.188730
C	0.718236	2.733760	0.065789
C	1.926369	1.818161	0.158417
H	0.930121	3.699324	0.551255
C	-0.757816	-0.489057	2.341310
H	-0.796660	0.368495	3.022808
H	-1.116627	-1.359025	2.907850
C	-3.005553	0.323786	1.569582
H	-2.854369	1.286227	2.074892
H	-3.699093	0.493181	0.733691
H	-3.497238	-0.349354	2.284528
H	-1.351284	2.836645	0.768755
H	-0.277927	1.930313	1.805341
H	0.503093	2.950400	-0.991047
C	2.300375	1.099039	-0.925088
H	1.723811	1.255330	-1.846894
C	3.377161	0.060017	-1.035463
H	4.067443	0.322221	-1.853165
H	3.983975	-0.004482	-0.123096
C	2.768175	-1.336090	-1.370655
H	2.280129	-1.285171	-2.355968
H	3.597551	-2.057276	-1.457307
C	1.785309	-1.825864	-0.329311
H	-0.037556	0.213922	-0.221239
H	0.242150	-1.877317	-1.724787
C	2.613329	1.774823	1.503303
H	2.945830	2.785051	1.787926
H	1.929422	1.439973	2.298479

H	3.491210	1.121252	1.520428
C	2.367463	-2.098704	1.035657
H	3.262729	-2.731024	0.940106
H	2.699850	-1.167007	1.516863
H	1.673928	-2.599620	1.717952
H	-1.000892	-3.480055	-0.257751
H	-2.543835	-2.212507	1.058615
H	0.287582	-0.665862	2.069379

C-D-TS (G781.712686, T-1390)

C	-1.488954	0.927814	-1.245079
C	-1.023404	0.858946	0.091952
C	-1.787083	-0.096017	1.071817
C	-2.126922	-1.433472	0.346715
C	-2.951160	-1.187498	-0.926562
C	-2.314891	-0.154174	-1.866937
H	-3.097679	-2.132313	-1.468345
H	-3.954451	-0.838705	-0.647696
H	-1.656850	-0.641027	-2.615378
H	-3.079557	0.335896	-2.493266
C	-1.080409	2.027502	-2.177571
H	-1.176852	3.017142	-1.712156
H	-0.019316	1.911133	-2.448342
H	-1.662215	2.004934	-3.105073
C	-0.881143	-2.330087	0.100126
C	-0.425545	2.130196	0.734177
C	0.280440	-1.708817	-0.649193
H	-0.536491	-2.729625	1.060425
C	0.958162	2.645096	0.258716
C	2.041399	1.593993	0.356736
H	1.205470	3.505120	0.901451
C	-0.951808	-0.407342	2.337329
H	-0.927275	0.439711	3.032860
H	-1.408052	-1.246246	2.879392
C	-3.087144	0.624545	1.519115
H	-2.854203	1.536583	2.083317
H	-3.716698	0.913454	0.667891
H	-3.675946	-0.030140	2.176049
H	-1.159291	2.941350	0.600498
H	-0.366001	1.969054	1.815577
H	0.907179	3.032167	-0.765490
C	2.507960	1.018805	-0.773728
H	2.108453	1.387734	-1.726766
C	3.451798	-0.137944	-0.896013
H	4.201974	0.057072	-1.676791
H	4.003961	-0.324425	0.033861
C	2.672450	-1.426868	-1.317858
H	2.222469	-1.260724	-2.307506
H	3.399862	-2.248483	-1.421043
C	1.614706	-1.838246	-0.325913
H	0.008681	-0.050655	-0.318641
H	0.093525	-1.471830	-1.703517
C	2.524119	1.273250	1.751740
H	2.894709	2.189832	2.235977

H	1.710933	0.896345	2.388915
H	3.333472	0.537107	1.774547
C	2.114618	-2.399009	0.975666
H	2.532213	-3.402426	0.790206
H	2.938525	-1.796422	1.379178
H	1.345996	-2.499085	1.747022
H	-1.215555	-3.203216	-0.484861
H	-2.766433	-2.006548	1.037784
H	0.082302	-0.676796	2.098514

D (G781.725740)

C	1.636337	0.644002	1.313989
C	1.389822	0.840913	-0.019563
C	1.756988	-0.235598	-1.066950
C	1.768050	-1.670605	-0.442730
C	2.586466	-1.708836	0.856943
C	2.120552	-0.669512	1.883622
H	2.548721	-2.716557	1.296739
H	3.642274	-1.522242	0.617440
H	1.314884	-1.081484	2.526473
H	2.934976	-0.461065	2.596586
C	1.439038	1.717748	2.362005
H	1.559331	2.735117	1.973649
H	0.436382	1.654145	2.817478
H	2.156676	1.587757	3.182984
C	0.365167	-2.313379	-0.294719
C	0.983605	2.216180	-0.539147
C	-0.663501	-1.542371	0.542720
H	-0.040288	-2.494629	-1.297679
C	-0.395131	2.799699	-0.080111
C	-1.582524	1.904251	-0.333280
H	-0.530850	3.746326	-0.627233
C	0.814477	-0.235752	-2.298149
H	0.947284	0.657452	-2.919491
H	1.049382	-1.098106	-2.937233
C	3.182631	0.111899	-1.588287
H	3.192264	1.107211	-2.052256
H	3.922205	0.120478	-0.779521
H	3.503697	-0.614322	-2.348872
H	1.738941	2.951881	-0.220095
H	1.014447	2.228239	-1.634768
H	-0.353853	3.053879	0.984656
C	-2.178598	1.280526	0.717393
H	-1.766877	1.470276	1.714364
C	-3.357677	0.372886	0.677167
H	-4.147798	0.735286	1.352047
H	-3.797549	0.279974	-0.322790
C	-3.028325	-1.097018	1.246104
H	-2.619197	-0.976414	2.257144
H	-3.982716	-1.637225	1.289993
C	-2.081787	-1.749782	0.329986
H	-0.564518	-0.407755	0.354677
H	-0.438321	-1.554411	1.621126
C	-2.073152	1.825060	-1.759009

H	-2.675732	2.718893	-1.986454
H	-1.236328	1.826425	-2.467327
H	-2.697423	0.948578	-1.969519
C	-2.601648	-2.410861	-0.894673
H	-2.312372	-3.476559	-0.880883
H	-3.690369	-2.342802	-0.984872
H	-2.125821	-2.000625	-1.799836
H	0.492652	-3.307333	0.162416
H	2.287841	-2.314251	-1.172614
H	-0.246677	-0.292835	-2.026201

D (G781.725746)

C	1.636854	0.644572	1.313570
C	1.389682	0.841040	-0.019893
C	1.756450	-0.235707	-1.067197
C	1.768295	-1.670543	-0.442591
C	2.587298	-1.708130	0.856731
C	2.121300	-0.668824	1.883383
H	2.550200	-2.715779	1.296744
H	3.642917	-1.521071	0.616765
H	1.315724	-1.080919	2.526252
H	2.935741	-0.460232	2.596276
C	1.440089	1.718561	2.361418
H	1.559187	2.735910	1.972669
H	0.438062	1.654326	2.818180
H	2.158844	1.589325	3.181554
C	0.365672	-2.313761	-0.293845
C	0.983134	2.216121	-0.539679
C	-0.663171	-1.542655	0.543265
H	-0.039847	-2.495779	-1.296641
C	-0.395580	2.799524	-0.080250
C	-1.582976	1.904007	-0.333075
H	-0.531518	3.746147	-0.627308
C	0.813142	-0.236448	-2.297804
H	0.945333	0.656633	-2.919448
H	1.047894	-1.098898	-2.936812
C	3.181641	0.111985	-1.589600
H	3.190788	1.107189	-2.053814
H	3.921768	0.120908	-0.781349
H	3.502330	-0.614350	-2.350233
H	1.738397	2.952038	-0.220985
H	1.013575	2.227988	-1.635307
H	-0.353954	3.053621	0.984522
C	-2.178551	1.280055	0.717761
H	-1.766500	1.469765	1.714597
C	-3.357692	0.372472	0.677880
H	-4.147513	0.734689	1.353195
H	-3.797955	0.279670	-0.321904
C	-3.028068	-1.097595	1.246517
H	-2.619086	-0.977088	2.257621
H	-3.982378	-1.637972	1.290163
C	-2.081360	-1.749985	0.330334
H	-0.564344	-0.408028	0.354708
H	-0.438039	-1.554090	1.621677

C	-2.074059	1.824960	-1.758651
H	-2.675671	2.719420	-1.986180
H	-1.237415	1.825193	-2.467190
H	-2.699356	0.949120	-1.968784
C	-2.601093	-2.410391	-0.894772
H	-2.312517	-3.476267	-0.881189
H	-3.689739	-2.341619	-0.985395
H	-2.124697	-2.000060	-1.799597
H	0.493583	-3.307376	0.163893
H	2.287972	-2.314221	-1.172528
H	-0.247828	-0.293743	-2.025169

D-E1-TS (G781.712802, T-1213)

C	1.762668	0.518157	1.284249
C	1.483792	0.753897	-0.028336
C	1.626709	-0.339186	-1.111893
C	1.576873	-1.778112	-0.503171
C	2.464038	-1.889179	0.747095
C	2.118031	-0.852568	1.820400
H	2.397857	-2.904456	1.165223
H	3.513051	-1.749679	0.450011
H	1.291865	-1.208205	2.466684
H	2.967111	-0.746227	2.515014
C	1.748079	1.594879	2.349756
H	1.875293	2.611266	1.961343
H	0.809630	1.573942	2.931260
H	2.553290	1.421231	3.076875
C	0.152087	-2.362817	-0.298661
C	1.219075	2.168158	-0.522002
C	-0.855103	-1.633076	0.558694
H	-0.286650	-2.569911	-1.282280
C	-0.111830	2.859216	-0.030034
C	-1.339052	2.050182	-0.277866
H	-0.191021	3.813677	-0.571267
C	0.556213	-0.228912	-2.229337
H	0.674418	0.680715	-2.830193
H	0.664565	-1.074408	-2.922215
C	3.012660	-0.125773	-1.790414
H	3.079885	0.872580	-2.243573
H	3.833952	-0.213417	-1.069812
H	3.169242	-0.863351	-2.590554
H	2.021443	2.843094	-0.190598
H	1.232537	2.205911	-1.617001
H	-0.024886	3.077810	1.039324
C	-1.794138	1.180187	0.723904
H	-1.321676	1.321344	1.704003
C	-3.184119	0.576344	0.787203
H	-3.799450	1.163048	1.484446
H	-3.684731	0.610299	-0.189201
C	-3.112359	-0.902695	1.292031
H	-2.711702	-0.913400	2.315595
H	-4.126047	-1.323172	1.312902
C	-2.222365	-1.654923	0.345973
H	-1.041883	0.027044	0.302723

H	-0.532413	-1.359578	1.568290
C	-1.993555	2.162304	-1.618201
H	-2.695863	3.013828	-1.590065
H	-1.258790	2.387357	-2.400476
H	-2.569945	1.274653	-1.900405
C	-2.864517	-2.221012	-0.890828
H	-3.090801	-3.287351	-0.722961
H	-3.818117	-1.725322	-1.112464
H	-2.221285	-2.171303	-1.777231
H	0.283359	-3.352869	0.174152
H	2.019311	-2.441473	-1.265811
H	-0.470689	-0.244106	-1.843104

E1 (G781.723491)

C	-1.764215	0.615436	-1.232051
C	-1.385936	0.813885	0.067794
C	-1.505197	-0.290933	1.148093
C	-1.668776	-1.727552	0.536518
C	-2.646118	-1.712438	-0.650715
C	-2.247935	-0.720529	-1.746809
H	-2.736191	-2.723237	-1.073556
H	-3.648274	-1.442025	-0.287179
H	-1.475545	-1.147375	-2.413834
H	-3.107525	-0.541737	-2.412795
C	-1.732244	1.707700	-2.279266
H	-1.880630	2.715895	-1.875039
H	-0.773178	1.706295	-2.827030
H	-2.510161	1.540535	-3.035110
C	-0.355949	-2.518947	0.237640
C	-1.056782	2.206231	0.561393
C	0.694017	-1.885838	-0.632342
H	0.090360	-2.825986	1.191690
C	0.262018	2.950395	0.000989
C	1.456901	2.128844	0.221209
H	0.318598	3.896275	0.554830
C	-0.312708	-0.282309	2.139312
H	-0.262051	0.647571	2.720142
H	-0.440924	-1.094981	2.866428
C	-2.788032	0.034034	1.974224
H	-2.722777	1.022920	2.448254
H	-3.686328	0.030848	1.345496
H	-2.922045	-0.705224	2.776137
H	-1.854585	2.907530	0.279255
H	-0.991748	2.232095	1.653726
H	0.111928	3.152565	-1.063729
C	1.754518	1.104214	-0.752560
H	1.385281	1.375352	-1.752748
C	3.110184	0.391633	-0.804606
H	3.790806	0.960429	-1.454569
H	3.578159	0.358640	0.188585
C	2.930827	-1.055462	-1.334898
H	2.497606	-1.019940	-2.345793
H	3.920245	-1.528271	-1.415757
C	2.031777	-1.825326	-0.389275

H	0.964718	0.319591	-0.434764
H	0.361406	-1.503715	-1.602139
C	2.202905	2.246207	1.499072
H	3.116980	2.837763	1.302360
H	1.630114	2.775229	2.268642
H	2.548696	1.275845	1.877148
C	2.709679	-2.399218	0.831456
H	3.237100	-3.327101	0.557732
H	3.477112	-1.716109	1.224480
H	2.019877	-2.642627	1.646115
H	-0.673728	-3.457876	-0.250528
H	-2.150170	-2.326060	1.328012
H	0.653271	-0.433272	1.644461

C (G781.726424)

C	-1.733164	0.904918	-1.127533
C	-0.970682	0.784810	0.105613
C	-1.657499	-0.277330	1.105606
C	-1.893047	-1.636307	0.378079
C	-2.690883	-1.457686	-0.924674
C	-2.151191	-0.326555	-1.835315
H	-2.702173	-2.394236	-1.498455
H	-3.739685	-1.220485	-0.698113
H	-1.212477	-0.676788	-2.313912
H	-2.844171	-0.098426	-2.656344
C	-2.121743	2.191511	-1.750789
H	-1.630383	3.078294	-1.349845
H	-2.014064	2.148061	-2.845069
H	-3.212128	2.295621	-1.580148
C	-0.634077	-2.544060	0.197772
C	-0.523137	2.113081	0.754514
C	0.479647	-2.013609	-0.663562
H	-0.257405	-2.820764	1.189197
C	0.718976	2.733459	0.065945
C	1.926968	1.817663	0.158418
H	0.931047	3.698971	0.551430
C	-0.759212	-0.488869	2.341680
H	-0.798140	0.368798	3.023033
H	-1.118690	-1.358631	2.908119
C	-3.006237	0.324530	1.568534
H	-2.855099	1.287001	2.073801
H	-3.699283	0.493986	0.732244
H	-3.498500	-0.348389	2.283289
H	-1.350442	2.836687	0.769206
H	-0.277123	1.929959	1.805523
H	0.503770	2.950168	-0.990861
C	2.300771	1.098532	-0.925149
H	1.724191	1.255032	-1.846904
C	3.377237	0.059182	-1.035556
H	4.067421	0.321004	-1.853461
H	3.984205	-0.005303	-0.123289
C	2.767827	-1.336837	-1.370367
H	2.279836	-1.286079	-2.355718
H	3.597023	-2.058259	-1.456768

C	1.784783	-1.826062	-0.328938
H	-0.037351	0.213626	-0.220802
H	0.241874	-1.878427	-1.724696
C	2.613942	1.774091	1.503309
H	2.945545	2.784437	1.788541
H	1.930350	1.438135	2.298294
H	3.492410	1.121292	1.520021
C	2.366675	-2.098111	1.036298
H	3.262236	-2.730087	0.941246
H	2.698535	-1.166082	1.517213
H	1.673153	-2.599057	1.718585
H	-1.001673	-3.480058	-0.257151
H	-2.544802	-2.211973	1.058081
H	0.286280	-0.666124	2.070406

C-E1-TS (G781.704421, T-364)

C	1.596929	0.672585	1.258182
C	1.115082	0.848529	-0.041630
C	1.456209	-0.199810	-1.148706
C	1.775871	-1.625431	-0.575727
C	2.734252	-1.537367	0.625201
C	2.231710	-0.603334	1.731180
H	2.906421	-2.539509	1.041410
H	3.715081	-1.175147	0.285937
H	1.509057	-1.111192	2.394994
H	3.055629	-0.325748	2.410361
C	1.556285	1.748071	2.306518
H	0.839259	2.551637	2.127336
H	1.367043	1.316496	3.299296
H	2.555824	2.212540	2.360883
C	0.556321	-2.555822	-0.308132
C	0.866742	2.281111	-0.545450
C	-0.506283	-2.014022	0.600227
H	0.119002	-2.852035	-1.269856
C	-0.472597	2.981328	-0.114023
C	-1.604021	2.014832	-0.225131
H	-0.622661	3.843860	-0.776110
C	0.325614	-0.296754	-2.199762
H	0.198991	0.642083	-2.753289
H	0.577312	-1.067283	-2.939863
C	2.739940	0.311907	-1.873717
H	2.574842	1.271433	-2.379024
H	3.579805	0.445157	-1.180206
H	3.042010	-0.409874	-2.644016
H	1.687916	2.933925	-0.220409
H	0.894267	2.277770	-1.639409
H	-0.392250	3.359186	0.911688
C	-1.817798	1.128497	0.823822
H	-1.363459	1.403715	1.785257
C	-3.019204	0.213494	0.934821
H	-3.697102	0.682894	1.667063
H	-3.574443	0.180882	-0.011125
C	-2.702318	-1.227852	1.397198
H	-2.197878	-1.195008	2.374954

H	-3.656186	-1.757847	1.539880
C	-1.835498	-1.926837	0.373812
H	-0.398295	0.531351	0.411308
H	-0.179905	-1.681684	1.589351
C	-2.339813	1.955552	-1.529972
H	-3.184257	2.664667	-1.486545
H	-1.701097	2.276296	-2.361886
H	-2.754091	0.968072	-1.755284
C	-2.541829	-2.428974	-0.862616
H	-3.213649	-3.259181	-0.593575
H	-3.180976	-1.652879	-1.311030
H	-1.861442	-2.792771	-1.639157
H	0.965257	-3.483088	0.130068
H	2.331758	-2.136988	-1.378603
H	-0.635337	-0.559948	-1.746956

E1 (G781.723487)

C	-1.764024	0.615314	-1.232253
C	-1.386036	0.813889	0.067649
C	-1.505341	-0.290875	1.148013
C	-1.668757	-1.727557	0.536505
C	-2.645927	-1.712596	-0.650884
C	-2.247744	-0.720670	-1.746987
H	-2.735787	-2.723419	-1.073719
H	-3.648167	-1.442317	-0.287498
H	-1.475401	-1.147534	-2.414040
H	-3.107365	-0.541809	-2.412915
C	-1.731589	1.707480	-2.279568
H	-1.880243	2.715696	-1.875456
H	-0.772226	1.706191	-2.826798
H	-2.509091	1.540214	-3.035810
C	-0.355840	-2.518837	0.237940
C	-1.057053	2.206268	0.561187
C	0.694054	-1.885736	-0.632121
H	0.090483	-2.825552	1.192096
C	0.262098	2.950358	0.001064
C	1.456803	2.128641	0.221323
H	0.318636	3.896076	0.555176
C	-0.312887	-0.282072	2.139271
H	-0.262360	0.647847	2.720047
H	-0.441045	-1.094713	2.866430
C	-2.788247	0.034042	1.974042
H	-2.723131	1.022952	2.448034
H	-3.686502	0.030747	1.345256
H	-2.922263	-0.705184	2.775985
H	-1.854639	2.907619	0.278625
H	-0.992289	2.232274	1.653520
H	0.112073	3.152730	-1.063615
C	1.754562	1.104160	-0.752516
H	1.384981	1.375155	-1.752623
C	3.110184	0.391694	-0.804839
H	3.790571	0.960508	-1.455022
H	3.578461	0.358795	0.188214
C	2.930824	-1.055485	-1.334898

H	2.497575	-1.020175	-2.345781
H	3.920253	-1.528283	-1.415683
C	2.031807	-1.825210	-0.389105
H	0.964665	0.319681	-0.434422
H	0.361430	-1.503612	-1.601908
C	2.202404	2.245830	1.499475
H	3.114595	2.840644	1.303842
H	1.627979	2.772093	2.269758
H	2.550890	1.275932	1.876126
C	2.709749	-2.398842	0.831744
H	3.236974	-3.326933	0.558405
H	3.477295	-1.715665	1.224444
H	2.019982	-2.641775	1.646596
H	-0.673415	-3.457983	-0.249947
H	-2.150276	-2.326015	1.327963
H	0.653133	-0.432960	1.644465

E2 (G781.718190)

C	-1.756724	1.275713	-0.859626
C	-1.375823	0.857435	0.384404
C	-1.985580	-0.427220	0.975586
C	-2.107551	-1.491442	-0.149565
C	-2.969644	-0.975546	-1.312273
C	-2.530716	0.405644	-1.827514
H	-2.958555	-1.704477	-2.136100
H	-4.012160	-0.923552	-0.971437
H	-1.889235	0.293778	-2.723994
H	-3.405545	0.965155	-2.198535
C	-1.418022	2.628279	-1.451334
H	-1.050335	3.360006	-0.724137
H	-0.675219	2.547922	-2.263138
H	-2.318332	3.056370	-1.916792
C	-0.712656	-2.027664	-0.577438
C	-0.455946	1.722579	1.233077
C	0.380193	-1.080362	-0.982435
H	-0.345108	-2.716586	0.191268
C	0.933557	1.943798	0.580502
C	2.070503	0.995465	0.736184
H	1.366996	2.863350	1.034236
H	-2.630004	-2.365720	0.274507
C	-1.185019	-1.029195	2.152314
H	-1.210748	-0.387145	3.042098
H	-1.631098	-1.990131	2.442108
H	-0.136695	-1.218255	1.895651
C	-3.391335	-0.058728	1.536001
H	-3.897058	-0.952773	1.928604
H	-3.291682	0.661282	2.359977
H	-4.033502	0.401429	0.777252
H	-0.898470	2.719748	1.370639
H	-0.334726	1.314956	2.242351
H	0.830262	2.190442	-0.482207
C	3.247985	1.279513	-0.149521
H	4.006814	1.749435	0.504375
C	3.887504	0.057461	-0.832202

H	4.678592	0.397616	-1.513406
H	4.371041	-0.592499	-0.089461
C	2.784393	-0.693300	-1.588853
H	2.333922	-0.028954	-2.342295
H	3.232860	-1.538177	-2.139167
C	1.716696	-1.255816	-0.672285
H	2.971826	2.018916	-0.914083
H	0.149548	-0.348951	-1.759832
C	2.311577	0.308782	2.034711
H	2.547739	1.098243	2.773696
H	1.428395	-0.207220	2.421894
H	3.164391	-0.375384	2.006948
C	2.164642	-2.367869	0.257496
H	3.212269	-2.256879	0.557567
H	1.561041	-2.446950	1.168915
H	2.088150	-3.334620	-0.264529
H	-0.873576	-2.655531	-1.476347

E2-F-TS (G781.717813, T-396)

C	-1.778823	1.029216	-1.149927
C	-1.748414	0.848425	0.198796
C	-2.296905	-0.473655	0.760993
C	-1.544672	-1.589231	-0.010095
C	-1.858259	-1.531214	-1.527497
C	-2.155801	-0.105388	-2.082983
H	-1.021854	-1.976157	-2.089286
H	-2.723961	-2.174687	-1.728739
H	-1.616202	0.043935	-3.033209
H	-3.219946	-0.025411	-2.359517
C	-1.451477	2.327022	-1.855414
H	-1.391745	3.192140	-1.184909
H	-0.511096	2.269475	-2.430799
H	-2.237528	2.543688	-2.595644
C	-0.023815	-1.638782	0.306900
C	-1.027288	1.822932	1.108805
C	0.957868	-0.615571	-0.313994
H	0.123249	-1.694759	1.393230
C	0.456057	1.992477	0.712499
C	1.523625	0.880148	0.782567
H	0.880689	2.771653	1.372702
H	-1.916050	-2.559819	0.364875
C	-2.124476	-0.652298	2.287737
H	-2.712827	0.091356	2.841205
H	-2.497697	-1.643680	2.578591
H	-1.090791	-0.579293	2.645847
C	-3.830489	-0.575530	0.517532
H	-4.204518	-1.548777	0.866818
H	-4.348167	0.207598	1.086623
H	-4.126166	-0.461709	-0.529577
H	-1.467565	2.830767	1.046243
H	-1.111985	1.533955	2.161695
H	0.492757	2.410449	-0.299451
C	2.794187	1.492165	0.144329
H	3.227277	2.154321	0.911625

C	3.871952	0.547939	-0.365554
H	4.687691	1.105864	-0.844984
H	4.319641	-0.050571	0.440725
C	3.212664	-0.385585	-1.441447
H	2.736939	0.239944	-2.207177
H	3.969956	-1.033915	-1.899835
C	2.241439	-1.153540	-0.635318
H	2.495829	2.144419	-0.689567
H	0.531733	0.018944	-1.102657
C	1.764460	0.304072	2.169863
H	2.043968	1.128447	2.842625
H	0.868464	-0.157098	2.595014
H	2.578696	-0.430408	2.190015
C	2.745137	-2.421774	-0.031486
H	3.840378	-2.463594	-0.022728
H	2.341702	-2.623410	0.966855
H	2.402187	-3.253248	-0.675812
H	0.295133	-2.621377	-0.068636

F (G781.725497)

C	-1.453867	0.480500	1.478705
C	-1.567589	-0.651838	0.701195
C	-2.265132	-0.513461	-0.660043
C	-1.550127	0.694243	-1.340133
C	-1.930493	1.986394	-0.588967
C	-1.851697	1.852744	0.959262
H	-1.323725	2.832467	-0.941551
H	-2.958514	2.242892	-0.871149
H	-1.131995	2.576961	1.378398
H	-2.813949	2.144187	1.412295
C	-0.976543	0.479420	2.913133
H	-0.902674	-0.521235	3.350871
H	0.002275	0.975830	3.021214
H	-1.679694	1.061885	3.527766
C	-0.025721	0.478720	-1.582926
C	-0.875608	-1.943613	1.073333
C	0.992083	0.290175	-0.352223
H	0.112704	-0.401026	-2.220679
C	0.659610	-1.787901	1.197149
C	1.500434	-1.174082	0.027363
H	1.081729	-2.783733	1.402625
H	-1.958844	0.777890	-2.363194
C	-2.217408	-1.756507	-1.574368
H	-2.714623	-2.618156	-1.111324
H	-2.762393	-1.529389	-2.500348
H	-1.210276	-2.068834	-1.864728
C	-3.781364	-0.235488	-0.434725
H	-4.262846	0.027482	-1.387423
H	-4.264801	-1.145292	-0.055812
H	-3.991828	0.561418	0.285664
H	-1.236367	-2.308146	2.048718
H	-1.118828	-2.731478	0.353440
H	0.868554	-1.184334	2.091694
C	2.942786	-1.121469	0.625343

H	3.309216	-2.153397	0.709516
C	3.905185	-0.257492	-0.180839
H	4.929587	-0.338942	0.204200
H	3.934054	-0.565545	-1.236434
C	3.420888	1.183475	-0.065368
H	3.616582	1.569179	0.957147
H	3.964642	1.890724	-0.717541
C	1.964777	1.366468	-0.316494
H	2.906866	-0.722832	1.653775
H	0.279489	0.559719	0.509451
C	1.535879	-2.124487	-1.188211
H	2.024259	-3.065089	-0.901941
H	0.540719	-2.390230	-1.555135
H	2.098290	-1.699492	-2.030499
C	1.556966	2.785229	-0.538698
H	2.153757	3.461900	0.088711
H	1.797436	3.058966	-1.582301
H	0.493791	2.972650	-0.385126
H	0.319043	1.326808	-2.185224

C (G781.726433)

C	-1.734059	0.904468	-1.127455
C	-0.971061	0.784805	0.105346
C	-1.656721	-0.277683	1.105882
C	-1.892481	-1.636680	0.378501
C	-2.691266	-1.458254	-0.923672
C	-2.152349	-0.327184	-1.834843
H	-2.702910	-2.394862	-1.497346
H	-3.739915	-1.221105	-0.696351
H	-1.214013	-0.677353	-2.314177
H	-2.845980	-0.099205	-2.655366
C	-2.122999	2.190890	-1.750853
H	-2.016413	2.147034	-2.845214
H	-3.213170	2.295337	-1.579078
H	-1.631044	3.077726	-1.350732
C	-0.633411	-2.544142	0.197424
C	-0.523911	2.113305	0.754066
C	0.480074	-2.013091	-0.663839
H	-0.256481	-2.821229	1.188643
C	0.718243	2.733834	0.065734
C	1.926342	1.818197	0.158394
H	0.930141	3.699360	0.551280
C	-0.757291	-0.489006	2.341155
H	-0.795950	0.368561	3.022645
H	-1.115961	-1.358954	2.907817
H	0.288023	-0.665843	2.068936
C	-3.005231	0.323824	1.569966
H	-3.496743	-0.349288	2.285061
H	-2.853909	1.286275	2.075212
H	-3.698983	0.493197	0.734250
H	-1.351373	2.836730	0.768388
H	-0.278158	1.930468	1.805179
H	0.503209	2.950572	-0.991102
C	2.300388	1.099097	-0.925113

H	1.723865	1.255431	-1.846939
C	3.377128	0.060039	-1.035471
H	4.067443	0.322254	-1.853142
H	3.983911	-0.004519	-0.123086
C	2.768125	-1.336046	-1.370780
H	2.280045	-1.285029	-2.356069
H	3.597513	-2.057202	-1.457529
C	1.785317	-1.825930	-0.329440
H	-0.037587	0.213974	-0.221457
H	0.242048	-1.877088	-1.724804
C	2.613286	1.774879	1.503284
H	3.490652	1.120623	1.520694
H	2.946601	2.784973	1.787445
H	1.929127	1.440938	2.298622
C	2.367530	-2.098860	1.035491
H	3.263269	-2.730482	0.939859
H	2.699151	-1.167069	1.517059
H	1.674252	-2.600484	1.717530
H	-1.000932	-3.480006	-0.257841
H	-2.543651	-2.212501	1.058941

C-G-TS (G781.715535, T-861)

C	1.397570	0.811936	1.308135
C	1.170694	0.980494	-0.081001
C	1.693947	-0.053011	-1.093245
C	2.058155	-1.429069	-0.435598
C	2.907020	-1.232821	0.829409
C	2.230826	-0.328004	1.866140
H	3.128886	-2.207074	1.285129
H	3.877090	-0.794469	0.557112
H	1.607475	-0.894747	2.575671
H	2.993262	0.156284	2.496560
C	1.034042	1.877961	2.331358
H	-0.027007	1.841685	2.603131
H	1.606513	1.713573	3.249512
H	1.250956	2.887118	1.961536
C	0.867182	-2.412490	-0.215461
C	0.572266	2.275342	-0.613401
C	-0.299776	-1.944264	0.619371
H	0.513847	-2.753343	-1.195621
C	-0.840195	2.721354	-0.117695
C	-1.917412	1.669478	-0.289373
H	-1.086970	3.615799	-0.709652
C	0.701081	-0.281362	-2.258977
H	0.623127	0.593566	-2.914094
H	1.071425	-1.107453	-2.878930
H	-0.299094	-0.540386	-1.902194
C	2.987881	0.602237	-1.677609
H	3.431793	-0.087807	-2.406851
H	2.763301	1.539452	-2.201311
H	3.736363	0.815732	-0.905354
H	1.287415	3.072611	-0.350318
H	0.553437	2.225632	-1.706027
H	-0.794965	3.048363	0.926252

C	-2.342727	0.974414	0.789403
H	-1.904805	1.238595	1.761082
C	-3.343518	-0.143188	0.853628
H	-4.108430	0.092614	1.610200
H	-3.876130	-0.271507	-0.097395
C	-2.658878	-1.479592	1.266078
H	-2.207863	-1.356329	2.263081
H	-3.439174	-2.252153	1.358956
C	-1.610284	-1.933254	0.270944
H	0.250239	0.284053	0.772197
H	-0.089306	-1.721889	1.671990
C	-2.473822	1.525776	-1.686682
H	-3.184485	0.700205	-1.789449
H	-2.997951	2.451639	-1.971683
H	-1.679173	1.379943	-2.432062
C	-2.145200	-2.366203	-1.073720
H	-2.913027	-3.141916	-0.932960
H	-2.642428	-1.535277	-1.595288
H	-1.382255	-2.771101	-1.745344
H	1.302498	-3.304455	0.267397
H	2.702316	-1.931351	-1.175496

G (G781.718708)

C	-1.507610	0.834997	-1.249815
C	-1.164803	0.781796	0.202482
C	-1.623702	-0.319314	1.086341
C	-1.870721	-1.670476	0.347188
C	-2.700953	-1.471174	-0.931930
C	-2.112639	-0.437703	-1.904530
H	-2.805474	-2.434867	-1.449675
H	-3.722615	-1.173902	-0.653492
H	-1.338569	-0.907917	-2.523744
H	-2.895670	-0.124783	-2.608058
C	-2.356543	2.105291	-1.600032
H	-1.890238	3.037781	-1.267066
H	-2.467326	2.152214	-2.688736
H	-3.357300	2.030029	-1.156201
C	-0.554706	-2.478131	0.136076
C	-0.553191	2.002771	0.810123
C	0.548209	-1.820150	-0.643716
H	-0.188059	-2.820742	1.111222
C	0.658496	2.666666	0.076053
C	1.905020	1.813800	0.171861
H	0.810218	3.637575	0.572517
C	-0.778833	-0.533448	2.365375
H	-0.869177	0.297755	3.073206
H	-1.141930	-1.432192	2.878869
H	0.278845	-0.676131	2.123507
C	-3.035575	0.274196	1.550378
H	-3.500281	-0.506226	2.167043
H	-2.899398	1.171733	2.162734
H	-3.707429	0.506512	0.719680
H	-1.375348	2.744368	0.817949
H	-0.291064	1.830617	1.856860

H	0.402820	2.876747	-0.970602
C	2.373421	1.195978	-0.931219
H	1.849678	1.387887	-1.876850
C	3.473324	0.176586	-1.020049
H	4.173359	0.436652	-1.829041
H	4.064513	0.125157	-0.096343
C	2.862320	-1.215370	-1.342686
H	2.380069	-1.168817	-2.330944
H	3.681390	-1.949119	-1.422753
C	1.862647	-1.707240	-0.308231
H	-0.516867	1.025390	-1.709970
H	0.308268	-1.547505	-1.674094
C	2.524095	1.708724	1.548502
H	3.512739	1.240100	1.532739
H	2.639111	2.708773	1.993747
H	1.897244	1.127859	2.243612
C	2.451294	-2.116074	1.018609
H	3.193661	-2.914881	0.865880
H	2.995346	-1.280208	1.482172
H	1.713724	-2.480945	1.740832
H	-0.850345	-3.393174	-0.406793
H	-2.481193	-2.276079	1.037104

G (G781.718913)

C	-2.051769	0.671609	-1.229153
C	-1.406241	0.786609	0.115476
C	-1.403356	-0.335935	1.088001
C	-1.506599	-1.779484	0.471946
C	-2.396705	-1.815806	-0.788707
C	-2.036689	-0.749964	-1.827686
H	-2.331807	-2.813809	-1.243453
H	-3.451379	-1.693150	-0.496875
H	-1.044095	-0.937638	-2.254954
H	-2.746327	-0.783199	-2.665738
C	-3.516071	1.243565	-1.189258
H	-3.578049	2.216148	-0.688792
H	-3.847304	1.366925	-2.226513
H	-4.197594	0.547440	-0.690619
C	-0.132150	-2.500909	0.291586
C	-0.929934	2.138534	0.503230
C	0.915760	-1.856826	-0.569015
H	0.275236	-2.726874	1.284149
C	0.299559	2.708483	-0.324823
C	1.622847	2.030335	-0.064466
H	0.348540	3.767975	-0.030545
C	-0.291372	-0.245433	2.158949
H	-0.412868	0.624957	2.812868
H	-0.351771	-1.132842	2.800124
H	0.698695	-0.209217	1.696958
C	-2.785810	-0.048506	1.850968
H	-2.780350	-0.719183	2.719261
H	-2.853946	0.983093	2.213970
H	-3.658425	-0.273917	1.234432
H	-1.752556	2.838937	0.284728

H	-0.698895	2.208913	1.569760
H	0.047474	2.682057	-1.393020
C	2.157205	1.260153	-1.035086
H	1.574916	1.150722	-1.959471
C	3.441411	0.480942	-1.020690
H	4.107902	0.849538	-1.817519
H	3.987677	0.607551	-0.076649
C	3.154019	-1.022469	-1.279840
H	2.721247	-1.133197	-2.285778
H	4.106974	-1.574633	-1.275216
C	2.210797	-1.607877	-0.243529
H	-1.503962	1.362518	-1.892444
H	0.644584	-1.679459	-1.613234
C	2.274210	2.333486	1.265265
H	3.183366	1.749071	1.438455
H	2.547364	3.398839	1.324138
H	1.593103	2.140875	2.108124
C	2.831928	-1.877184	1.105188
H	3.648160	-2.608382	0.998228
H	3.291948	-0.964993	1.515148
H	2.131844	-2.264460	1.851998
H	-0.382535	-3.481341	-0.150576
H	-2.023416	-2.379204	1.238921

G-H-TS (G781.713750, T-306)

C	-2.266483	0.352597	-1.120041
C	-1.590165	0.640533	0.230569
C	-1.030632	-0.405916	1.023825
C	-1.152011	-1.886769	0.629933
C	-2.102838	-2.125928	-0.563655
C	-1.961401	-1.060602	-1.652953
H	-1.907731	-3.124450	-0.976027
H	-3.148056	-2.148469	-0.213824
H	-0.942847	-1.070830	-2.062297
H	-2.639700	-1.280453	-2.488547
C	-3.774916	0.685151	-1.164469
H	-4.000398	1.678226	-0.753182
H	-4.109410	0.682265	-2.208427
H	-4.374886	-0.059022	-0.624985
C	0.254911	-2.568512	0.464695
C	-1.288840	2.114406	0.486776
C	1.201968	-1.911133	-0.499179
H	0.714129	-2.667464	1.456000
C	-0.159429	2.695856	-0.415104
C	1.239025	2.157862	-0.172697
H	-0.170085	3.782356	-0.229684
C	-0.010427	-0.128375	2.105120
H	-0.123878	0.850985	2.573108
H	-0.045408	-0.902379	2.880180
H	0.974466	-0.157073	1.624653
C	-2.718365	0.100393	1.656033
H	-2.397583	0.289716	2.680532
H	-3.360599	0.929586	1.346277
H	-3.260861	-0.833105	1.523661

H	-2.206440	2.681398	0.271135
H	-1.047748	2.300131	1.540443
H	-0.434279	2.566263	-1.471271
C	1.817689	1.366214	-1.099894
H	1.217739	1.109570	-1.982812
C	3.211982	0.801531	-1.107441
H	3.782043	1.271098	-1.927010
H	3.753889	1.046168	-0.183650
C	3.216750	-0.735434	-1.338955
H	2.768678	-0.947528	-2.321796
H	4.261298	-1.082088	-1.376803
C	2.452629	-1.464536	-0.250596
H	-1.780104	1.075896	-1.794515
H	0.843741	-1.807340	-1.527094
C	1.928059	2.666794	1.075087
H	2.813757	2.082196	1.346443
H	2.251500	3.710418	0.933771
H	1.253532	2.671725	1.944250
C	3.178190	-1.592487	1.069512
H	4.093255	-2.188778	0.931837
H	3.508221	-0.610511	1.442581
H	2.589586	-2.072849	1.858707
H	0.033053	-3.597011	0.134919
H	-1.583048	-2.388527	1.514030

H (G781.717552)

C	-2.489931	0.095685	-1.007704
C	-1.884031	0.497221	0.401149
C	-0.834784	-0.403603	0.937298
C	-0.818850	-1.860806	0.611339
C	-1.642768	-2.258882	-0.634838
C	-1.739294	-1.095775	-1.621142
H	-1.175509	-3.134259	-1.103830
H	-2.652607	-2.575818	-0.334315
H	-0.722767	-0.775443	-1.902333
H	-2.243650	-1.412986	-2.543791
C	-4.015397	-0.111289	-1.040390
H	-4.559905	0.768174	-0.673823
H	-4.328512	-0.272776	-2.079706
H	-4.347808	-0.981824	-0.460226
C	0.621822	-2.513911	0.638778
C	-1.580554	2.025316	0.431553
C	1.532258	-1.880834	-0.369430
H	1.039190	-2.473709	1.651692
C	-0.520420	2.529145	-0.578727
C	0.919898	2.154074	-0.304172
H	-0.596960	3.629820	-0.571362
C	0.090627	0.031653	2.012201
H	-0.106917	1.030890	2.398837
H	0.118581	-0.700039	2.831494
H	1.097433	0.024301	1.565617
C	-2.934744	0.242446	1.595886
H	-2.516114	0.511227	2.571372
H	-3.784635	0.904711	1.397337

H	-3.292316	-0.790597	1.622434
H	-2.537487	2.516452	0.199168
H	-1.322271	2.356756	1.447583
H	-0.795561	2.214772	-1.594943
C	1.598190	1.353324	-1.162250
H	1.046724	0.948750	-2.019559
C	3.069706	1.029058	-1.133519
H	3.555230	1.554533	-1.974290
H	3.548361	1.417377	-0.223705
C	3.362785	-0.489453	-1.278414
H	2.976938	-0.838209	-2.248337
H	4.453145	-0.639069	-1.287378
C	2.715612	-1.267984	-0.149025
H	-2.296931	0.960898	-1.658005
H	1.157307	-1.875539	-1.397768
C	1.573927	2.844259	0.876888
H	2.361619	2.241752	1.346157
H	2.040158	3.789170	0.556336
H	0.843159	3.107770	1.653320
C	3.454148	-1.243022	1.169669
H	4.409488	-1.780027	1.064166
H	3.714983	-0.215875	1.467027
H	2.908535	-1.710272	1.997517
H	0.450317	-3.578039	0.415553
H	-1.310406	-2.282604	1.516864

H (G781.717611)

C	-2.487234	0.096199	-1.008782
C	-1.882745	0.497012	0.400840
C	-0.834326	-0.404065	0.937900
C	-0.819484	-1.861560	0.612872
C	-1.645854	-2.259881	-0.631460
C	-1.740033	-1.098529	-1.620108
H	-1.181865	-3.137735	-1.099059
H	-2.656390	-2.572937	-0.329121
H	-0.722935	-0.781322	-1.902658
H	-2.245773	-1.416315	-2.541798
C	-4.013415	-0.104875	-1.043719
H	-4.555034	0.776662	-0.677799
H	-4.325699	-0.264998	-2.083493
H	-4.349922	-0.974235	-0.464147
C	0.621381	-2.514625	0.638148
C	-1.580225	2.025189	0.432923
C	1.531132	-1.881319	-0.370522
H	1.039690	-2.474869	1.650691
C	-0.519871	2.530939	-0.576223
C	0.920377	2.154806	-0.302808
H	-0.596137	3.631606	-0.566399
C	0.091370	0.031122	2.012538
H	-0.105924	1.030462	2.399082
H	0.119603	-0.700455	2.831902
H	1.097996	0.023803	1.565541
C	-2.934529	0.240454	1.594479
H	-2.516856	0.508052	2.570709

H	-3.784236	0.902972	1.395953
H	-3.292162	-0.792598	1.619384
H	-2.537263	2.516082	0.200436
H	-1.322745	2.355724	1.449458
H	-0.795177	2.218976	-1.593127
C	1.597572	1.354090	-1.161771
H	1.045171	0.950122	-2.018772
C	3.068927	1.029009	-1.134256
H	3.554103	1.554653	-1.975122
H	3.548426	1.416693	-0.224608
C	3.361171	-0.489575	-1.280084
H	2.974414	-0.837690	-2.249874
H	4.451454	-0.639713	-1.289943
C	2.714505	-1.268341	-0.150563
H	-2.289778	0.960095	-1.659471
H	1.155810	-1.876131	-1.398704
C	1.575551	2.843671	0.878399
H	2.042984	3.788125	0.558280
H	0.845213	3.107688	1.655079
H	2.362539	2.239900	1.347266
C	3.453706	-1.243545	1.167777
H	4.408971	-1.780567	1.061674
H	3.714735	-0.216458	1.465151
H	2.908540	-1.710894	1.995861
H	0.449564	-3.578648	0.414664
H	-1.309163	-2.283098	1.519474

H-J1-TS (G781.695492, T-1201)

C	-2.770846	0.025925	-0.753376
C	-1.975563	0.485863	0.538380
C	-0.765965	-0.425480	0.845305
C	-0.777311	-1.879220	0.395107
C	-1.427144	-2.109776	-1.006783
C	-1.868282	-0.810409	-1.678245
H	-0.711915	-2.633414	-1.655098
H	-2.285565	-2.787048	-0.905925
H	-0.973125	-0.230961	-1.964585
H	-2.400932	-1.032384	-2.613840
C	-4.096062	-0.716921	-0.488355
H	-4.826131	-0.085789	0.030942
H	-4.544039	-1.016540	-1.445211
H	-3.961548	-1.627689	0.109402
C	0.606134	-2.604501	0.490058
C	-1.582826	1.996541	0.378418
C	1.625167	-1.928241	-0.386206
H	0.939389	-2.670325	1.533772
C	-0.633385	2.424660	-0.789204
C	0.833614	2.317342	-0.458493
H	-0.834378	3.487406	-0.995465
C	0.269292	0.059055	1.598147
H	0.204618	1.042238	2.064750
H	1.056010	-0.585226	1.976639
H	0.961134	0.611494	0.188280
C	-2.856875	0.426758	1.822470

H	-2.292374	0.794453	2.688562
H	-3.750026	1.054581	1.705606
H	-3.189668	-0.592639	2.048769
H	-2.530470	2.527026	0.213921
H	-1.194485	2.394548	1.327487
H	-0.869302	1.866445	-1.702841
C	1.572007	1.209554	-0.879914
H	1.105057	0.606735	-1.666888
C	3.077155	1.032989	-0.753258
H	3.571344	1.701526	-1.476818
H	3.422723	1.348297	0.242194
C	3.498847	-0.438338	-1.016919
H	3.234125	-0.703794	-2.051552
H	4.593025	-0.508165	-0.931523
C	2.800338	-1.360297	-0.033422
H	-3.041831	0.947137	-1.295770
H	1.320957	-1.806596	-1.432331
C	1.416013	3.347693	0.456201
H	1.953858	4.086168	-0.163390
H	0.648803	3.888748	1.021219
H	2.159814	2.925384	1.143311
C	3.442003	-1.470690	1.328533
H	4.448916	-1.904384	1.230015
H	3.580701	-0.481664	1.795489
H	2.883216	-2.102810	2.027487
H	0.432989	-3.640213	0.156833
H	-1.428763	-2.367595	1.145879

J1 (G781.738751)

C	-3.006472	-0.091165	-0.668223
C	-2.054575	0.578392	0.418028
C	-0.760720	-0.156835	0.583045
C	-0.758553	-1.627636	0.399243
C	-1.419208	-2.055741	-0.974210
C	-2.156545	-0.907140	-1.661886
H	-0.617343	-2.433079	-1.620780
H	-2.091020	-2.900088	-0.777117
H	-1.420362	-0.247476	-2.148973
H	-2.799574	-1.305573	-2.458769
C	-4.176846	-0.935773	-0.130804
H	-4.862190	-0.351432	0.493502
H	-4.757448	-1.317785	-0.980751
H	-3.850190	-1.806145	0.454133
C	0.555295	-2.397972	0.680937
C	-1.757980	2.083862	0.097567
C	1.648444	-1.930929	-0.242639
H	0.838216	-2.273546	1.734554
C	-0.537153	2.250333	-0.814642
C	0.774252	1.782679	-0.126189
H	-0.430316	3.304361	-1.107315
C	0.391601	0.656031	0.977920
H	0.063742	1.255126	1.845301
H	1.274907	0.079413	1.244840
H	1.283805	0.347021	-1.672939

C	-2.717207	0.547955	1.853546
H	-2.051803	0.986135	2.606093
H	-3.626825	1.158260	1.806253
H	-2.988685	-0.465021	2.165895
H	-2.658859	2.519839	-0.353424
H	-1.589853	2.641355	1.031879
H	-0.695229	1.685021	-1.745995
C	1.746581	1.230227	-1.205997
H	1.760851	2.004474	-1.989846
C	3.212640	0.912223	-0.807043
H	3.892285	1.495765	-1.443479
H	3.419863	1.246462	0.219763
C	3.605401	-0.588553	-0.928177
H	3.382482	-0.938204	-1.947408
H	4.691514	-0.681298	-0.777897
C	2.841378	-1.395809	0.097765
H	-3.452317	0.752709	-1.216994
H	1.393779	-1.924713	-1.307565
C	1.402564	2.955230	0.654260
H	1.806743	3.693604	-0.051427
H	0.655812	3.460230	1.282624
H	2.222315	2.629706	1.306531
C	3.426508	-1.406584	1.489967
H	4.375753	-1.964154	1.479800
H	3.677260	-0.389757	1.831812
H	2.782818	-1.874393	2.243637
H	0.327546	-3.468119	0.541277
H	-1.514096	-1.935476	1.157976

J2 (G781.740568)

C	-2.657070	-0.063685	-0.951623
C	-2.003995	0.461651	0.417355
C	-0.705800	-0.220461	0.699773
C	-0.680381	-1.708122	0.721272
C	-1.649985	-2.329185	-0.341987
C	-1.949322	-1.331319	-1.463189
H	-1.169923	-3.232149	-0.740896
H	-2.586026	-2.658774	0.130892
H	-0.995634	-1.046972	-1.940655
H	-2.564874	-1.807028	-2.238894
C	-4.181897	-0.224238	-0.892133
H	-4.680482	0.684688	-0.531986
H	-4.555809	-0.419386	-1.905800
H	-4.499930	-1.062888	-0.259182
C	0.712492	-2.391040	0.791567
C	-1.821034	1.998643	0.366735
C	1.615234	-1.881141	-0.300506
H	1.155016	-2.214567	1.780240
C	-0.620344	2.376747	-0.507241
C	0.729765	1.831378	0.044532
H	-0.555798	3.468187	-0.612880
C	0.426345	0.632541	1.070905
H	0.070004	1.158936	1.981296
H	1.336281	0.087753	1.318239

H	1.069235	0.529633	-1.664267
C	-2.908734	0.113515	1.662850
H	-2.392074	0.364178	2.598225
H	-3.814034	0.726961	1.605224
H	-3.199504	-0.940765	1.687170
H	-2.747275	2.443083	-0.023431
H	-1.693157	2.403104	1.384245
H	-0.786687	1.980877	-1.522189
C	1.587452	1.369548	-1.174132
H	1.522138	2.209519	-1.883942
C	3.086831	1.021327	-0.987837
H	3.679793	1.625154	-1.689096
H	3.438047	1.313075	0.012453
C	3.433025	-0.474938	-1.230882
H	3.047458	-0.778056	-2.216077
H	4.527950	-0.583410	-1.258261
C	2.831916	-1.319971	-0.131498
H	-2.449441	0.739093	-1.675463
H	1.184617	-1.868158	-1.307802
C	1.447265	2.926969	0.858271
H	1.796921	3.721752	0.185759
H	0.768286	3.380407	1.594363
H	2.318700	2.538894	1.401137
C	3.615551	-1.345661	1.160970
H	4.582421	-1.842102	0.986677
H	3.856884	-0.329368	1.510867
H	3.120511	-1.881003	1.978685
H	0.534348	-3.476534	0.724126
H	-1.161839	-1.889472	1.712018

J2-K1-TS (G781.727272, T-787)

C	-2.769246	-0.110880	-0.885779
C	-2.055687	0.431909	0.429423
C	-0.686285	-0.254783	0.677774
C	-0.549752	-1.656872	0.424897
C	-1.736556	-2.356802	-0.191311
C	-2.241111	-1.484409	-1.358686
H	-1.433140	-3.357757	-0.520457
H	-2.540585	-2.502018	0.547225
H	-1.405702	-1.350330	-2.063752
H	-3.025083	-2.029262	-1.901093
C	-4.304574	-0.111795	-0.778933
H	-4.696425	0.862793	-0.461161
H	-4.737623	-0.331654	-1.763025
H	-4.676583	-0.871731	-0.077766
C	0.757368	-2.421422	0.611576
C	-1.809410	1.959046	0.267807
C	1.753977	-1.885793	-0.392103
H	1.131845	-2.281640	1.634214
C	-0.609264	2.242892	-0.638144
C	0.741032	1.786965	-0.005237
H	-0.553707	3.314141	-0.876322
C	0.458436	0.660855	1.066906
H	0.160538	1.170152	1.996180

H	1.373114	0.105492	1.282182
H	1.208096	0.446770	-1.652352
C	-2.909611	0.218814	1.713690
H	-2.354284	0.562421	2.597978
H	-3.833779	0.804626	1.667360
H	-3.193251	-0.830825	1.870101
H	-2.727287	2.404490	-0.141173
H	-1.659023	2.426944	1.253269
H	-0.756180	1.728352	-1.602558
C	1.665408	1.327182	-1.172364
H	1.600731	2.130533	-1.923024
C	3.166129	1.050340	-0.892083
H	3.779211	1.689394	-1.542759
H	3.433056	1.341117	0.134146
C	3.605484	-0.424753	-1.127264
H	3.323457	-0.727573	-2.146538
H	4.701233	-0.487864	-1.052845
C	2.935661	-1.302250	-0.096308
H	-2.511841	0.607969	-1.678209
H	1.407724	-1.859026	-1.429342
C	1.371953	2.971123	0.759944
H	1.704450	3.745294	0.055362
H	0.645695	3.428507	1.446796
H	2.241040	2.663371	1.356541
C	3.601034	-1.341044	1.258492
H	4.589446	-1.814550	1.158231
H	3.786929	-0.328458	1.648268
H	3.049080	-1.904169	2.020313
H	0.545405	-3.493117	0.488266
H	-0.886491	-1.052325	1.642156

K1 (G781.738444)

C	-3.056406	-0.336497	-0.325689
C	-2.090838	0.545800	0.525488
C	-0.735701	-0.240495	0.781573
C	-0.472007	-1.337053	-0.196273
C	-1.028353	-1.294938	-1.584663
C	-2.440303	-0.629308	-1.700764
H	-0.333932	-0.707479	-2.204975
H	-1.017643	-2.308255	-2.008254
H	-2.366620	0.290064	-2.293060
H	-3.084666	-1.316608	-2.264678
C	-3.479659	-1.648235	0.361404
H	-4.040479	-1.473323	1.285963
H	-4.119219	-2.236672	-0.309183
H	-2.620967	-2.290006	0.624616
C	0.297308	-2.555497	0.194730
C	-1.816367	1.920945	-0.176381
C	1.578476	-2.009135	-0.421638
H	0.370381	-2.701882	1.276335
C	-0.524660	2.020374	-1.004989
C	0.757850	1.762148	-0.161663
H	-0.466619	3.022745	-1.453847
C	0.415234	0.806775	1.010336

H	0.061840	1.403287	1.863311
H	1.305290	0.282698	1.363167
H	1.499910	0.341971	-1.647626
C	-2.716155	0.837596	1.908442
H	-2.063215	1.480977	2.512993
H	-3.669771	1.368935	1.781526
H	-2.913059	-0.074481	2.484819
H	-2.680435	2.176196	-0.806748
H	-1.779286	2.702711	0.597779
H	-0.559365	1.328287	-1.858644
C	1.875950	1.220822	-1.103546
H	2.029251	1.989310	-1.876673
C	3.246810	0.872305	-0.465841
H	4.047847	1.420152	-0.980461
H	3.287152	1.203116	0.582034
C	3.637317	-0.643414	-0.530834
H	3.661171	-0.957237	-1.584288
H	4.648506	-0.764446	-0.116470
C	2.632732	-1.447746	0.249122
H	-3.967381	0.262348	-0.493203
H	1.606182	-1.995089	-1.511963
C	1.229547	3.095772	0.468377
H	1.571691	3.789349	-0.311908
H	0.413909	3.582646	1.020469
H	2.058378	2.947883	1.174443
C	2.868277	-1.561296	1.730656
H	3.652806	-2.320633	1.886936
H	3.263354	-0.627197	2.151542
H	1.991434	-1.866947	2.310368
H	-0.051298	-3.471514	-0.297966
H	-0.809458	-0.754428	1.756570

K2 (G781.730615)

C	-3.120935	-0.265906	-0.213510
C	-1.990187	0.667045	0.335672
C	-0.751916	-0.219828	0.732114
C	-0.527187	-1.360874	-0.217915
C	-1.163121	-1.346201	-1.567533
C	-2.693816	-0.979528	-1.514211
H	-0.684025	-0.570097	-2.179753
H	-1.003613	-2.300312	-2.084335
H	-2.904687	-0.359602	-2.393520
H	-3.268249	-1.907602	-1.631561
C	-3.613803	-1.309406	0.809528
H	-3.938446	-0.861309	1.755184
H	-4.468800	-1.858870	0.395532
H	-2.839771	-2.058580	1.042957
C	0.240267	-2.589511	0.157527
C	-1.585533	1.730026	-0.728822
C	1.497784	-1.959352	-0.423158
H	0.314908	-2.755906	1.235959
C	-0.350145	2.568957	-0.351266
C	0.912965	1.734854	0.009442
H	-0.591361	3.209034	0.510869

C	0.477195	0.705744	1.079064
H	0.179824	1.263137	1.978565
H	1.303956	0.064051	1.384328
H	0.831394	0.333384	-1.648852
C	-2.500795	1.399276	1.600335
H	-2.628091	0.719841	2.452051
H	-1.828435	2.201564	1.921366
H	-3.476316	1.858065	1.390583
H	-1.411886	1.264761	-1.708884
H	-2.443143	2.403985	-0.876166
H	-0.113574	3.256379	-1.177244
C	1.511595	1.106456	-1.282544
H	1.484012	1.891620	-2.055194
C	2.961015	0.550672	-1.339118
H	3.063552	0.086980	-2.332547
H	3.668263	1.392895	-1.340151
C	3.492930	-0.440381	-0.266593
H	4.359182	-0.979728	-0.690853
H	3.915846	0.121136	0.580924
C	2.525582	-1.435887	0.325906
H	-3.974017	0.387580	-0.461325
H	1.509905	-1.795139	-1.501055
C	1.951809	2.677180	0.657991
H	2.840044	2.133768	1.007625
H	2.286430	3.432296	-0.066886
H	1.524825	3.206402	1.520651
C	2.816954	-1.848824	1.745586
H	2.865909	-0.989333	2.429203
H	2.120673	-2.587708	2.153189
H	3.824443	-2.296713	1.759395
H	-0.099604	-3.495122	-0.359012
H	-0.963088	-0.717945	1.697980

K2-L-TS (G781.733502, T-207)

C	-3.069671	-0.220311	-0.223552
C	-1.933537	0.697614	0.336878
C	-0.691715	-0.196724	0.728238
C	-0.476450	-1.322060	-0.266848
C	-1.097097	-1.229069	-1.630904
C	-2.639446	-0.969010	-1.511902
H	-0.667527	-0.401178	-2.204471
H	-0.911512	-2.145321	-2.205228
H	-2.927304	-0.406085	-2.408844
H	-3.160047	-1.934352	-1.562738
C	-3.610105	-1.231580	0.808477
H	-3.976061	-0.751118	1.722543
H	-4.448968	-1.791109	0.374832
H	-2.848857	-1.972501	1.099311
C	0.081253	-2.647995	0.110609
C	-1.533155	1.778692	-0.712923
C	1.338795	-1.987272	-0.427144
H	0.113800	-2.866795	1.179813
C	-0.279203	2.597617	-0.349131
C	0.975684	1.748715	0.006662

H	-0.502993	3.242504	0.514337
C	0.523555	0.724305	1.071178
H	0.238998	1.286466	1.972511
H	1.351378	0.082987	1.385413
H	0.846693	0.395371	-1.689485
C	-2.438868	1.419088	1.610691
H	-2.547846	0.736200	2.462220
H	-1.771786	2.228836	1.924734
H	-3.422118	1.867003	1.413466
H	-1.395530	1.336009	-1.708022
H	-2.384663	2.467890	-0.821491
H	-0.040214	3.280623	-1.178429
C	1.564120	1.111774	-1.284371
H	1.613709	1.914266	-2.037863
C	2.970232	0.454906	-1.306252
H	3.065368	-0.028943	-2.290499
H	3.739154	1.240931	-1.295064
C	3.400445	-0.553694	-0.205364
H	4.239933	-1.157805	-0.598086
H	3.835593	-0.012263	0.647710
C	2.368267	-1.492571	0.362450
H	-3.902758	0.449693	-0.494680
H	1.434578	-1.876935	-1.507215
C	2.030229	2.677419	0.648498
H	2.916656	2.123340	0.987279
H	2.366823	3.433135	-0.074818
H	1.617700	3.205822	1.518690
C	2.611546	-1.944728	1.776737
H	2.878148	-1.113799	2.441841
H	1.791737	-2.514342	2.221499
H	3.495661	-2.606148	1.749853
H	-0.285682	-3.497748	-0.473154
H	-0.921331	-0.705337	1.681875

L (G781.736541)

C	-3.017000	-0.124425	-0.232417
C	-1.858996	0.755457	0.340692
C	-0.636868	-0.168034	0.722767
C	-0.424497	-1.290638	-0.309472
C	-1.080103	-1.143964	-1.673197
C	-2.605976	-0.883027	-1.526494
H	-0.632639	-0.323327	-2.242865
H	-0.913337	-2.053244	-2.266912
H	-2.921683	-0.326103	-2.418761
H	-3.135120	-1.845422	-1.558022
C	-3.600772	-1.117756	0.793715
H	-3.973441	-0.622697	1.697405
H	-4.445480	-1.657824	0.346521
H	-2.863526	-1.874468	1.102275
C	-0.119091	-2.690193	0.086837
C	-1.436219	1.840679	-0.697049
C	1.113198	-1.961602	-0.429665
H	-0.113785	-2.943419	1.146799
C	-0.159781	2.622944	-0.331886

C	1.072807	1.736403	0.009833
H	-0.360441	3.266015	0.538655
C	0.590869	0.711783	1.058225
H	0.362288	1.262040	1.983462
H	1.416385	0.042404	1.350592
H	0.869303	0.452304	-1.735847
C	-2.347090	1.478435	1.621045
H	-2.483495	0.789427	2.463640
H	-1.652936	2.260182	1.948810
H	-3.313018	1.963378	1.425709
H	-1.318882	1.408989	-1.698888
H	-2.269842	2.553794	-0.789034
H	0.096887	3.306294	-1.156025
C	1.633011	1.092211	-1.289168
H	1.772361	1.909676	-2.014599
C	2.975416	0.315179	-1.287402
H	3.045733	-0.192744	-2.260896
H	3.813834	1.026013	-1.268138
C	3.291088	-0.706049	-0.158586
H	4.061443	-1.410978	-0.531388
H	3.776540	-0.202670	0.689179
C	2.179801	-1.541334	0.400667
H	-3.826156	0.575089	-0.502728
H	1.334099	-1.973809	-1.497967
C	2.160209	2.626407	0.650538
H	3.037813	2.045755	0.969021
H	2.507318	3.385184	-0.064455
H	1.773517	3.150517	1.535074
C	2.353422	-2.066045	1.795426
H	3.021947	-1.443032	2.397694
H	1.411640	-2.217256	2.331447
H	2.820780	-3.063547	1.699244
H	-0.500954	-3.488303	-0.552139
H	-0.897725	-0.676807	1.666671

Structures from Scheme 5 of main text (Table S13):

A* (G781.656565)

C	-2.332337	0.187746	-1.320258
C	-1.351193	1.119458	-1.270277
C	-1.177032	-1.775578	1.611449
C	-1.305334	-2.482682	0.450153
C	-2.347237	-2.302429	-0.622456
C	-2.044955	-1.235403	-1.732827
H	-2.481023	-3.270877	-1.124656
H	-3.317637	-2.054922	-0.166150
H	-0.999570	-1.340061	-2.064310
H	-2.685507	-1.491667	-2.593765
C	-3.778454	0.544804	-1.036976
H	-3.894291	1.106254	-0.099069
H	-4.177939	1.187505	-1.836759
H	-4.423086	-0.340946	-0.983519
C	2.827086	-1.197977	-2.870569
C	-1.543271	2.556655	-0.859121
C	3.288782	-0.545800	-1.759625
H	2.139920	-2.041785	-2.817070
C	-0.247345	3.273532	-0.403150
C	0.460328	2.541083	0.729215
H	-0.509557	4.293534	-0.088461
C	-0.198341	-2.209301	2.675207
H	0.458811	-1.381212	2.988240
H	-0.746110	-2.510639	3.582326
C	-2.006732	-0.567168	1.949247
H	-1.438874	0.354758	1.730545
H	-2.938562	-0.517381	1.378554
H	-2.248418	-0.544001	3.021435
H	-1.978343	3.134638	-1.695462
H	-2.282809	2.611877	-0.044089
H	0.432918	3.370732	-1.264108
C	1.668164	1.742240	0.303070
H	2.415768	2.439438	-0.118474
C	2.333424	0.845842	1.347885
H	2.775657	1.435663	2.162314
H	1.588522	0.176182	1.800084
C	3.463667	-0.014943	0.698242
H	4.265688	0.642057	0.336225
H	3.881571	-0.674363	1.476540
C	2.921600	-0.855318	-0.413452
H	-0.348567	0.817164	-1.591550
H	3.983883	0.286248	-1.894599
C	0.009660	2.593853	1.998172
H	-0.853471	3.206983	2.260894
H	0.488300	2.057903	2.817070
C	1.961011	-1.921036	-0.069098
H	1.883142	-2.715128	-0.817672
H	2.181878	-2.355547	0.912642
H	0.930736	-1.492240	0.036188
H	3.139554	-0.884165	-3.865980
H	-0.642967	-3.346744	0.329189

H	0.420008	-3.059623	2.362820
H	1.371417	1.114905	-0.558368

A*-B1*-TS (G781.656087, T-36)

C	-1.058390	2.275596	-0.688536
C	0.278307	2.070540	-0.794236
C	-2.676196	-0.753988	1.213758
C	-3.101161	-0.510983	-0.061337
C	-3.248778	0.834602	-0.729293
C	-2.031321	1.447011	-1.500656
H	-4.068206	0.735726	-1.454858
H	-3.591601	1.571737	0.013034
H	-1.497399	0.646772	-2.036479
H	-2.466118	2.114768	-2.265000
C	-1.647152	3.429556	0.091408
H	-0.884620	4.071811	0.542716
H	-2.265066	4.054884	-0.572186
H	-2.312844	3.088527	0.898288
C	-0.937671	-2.201030	-2.114250
C	1.355078	2.898865	-0.130413
C	0.408685	-2.307072	-1.871484
H	-1.685187	-2.417554	-1.354948
C	2.794581	2.338564	-0.258236
C	3.023635	1.050540	0.512926
H	3.490091	3.107039	0.105822
C	-2.958756	-2.083223	1.875139
H	-2.094639	-2.467610	2.435728
H	-3.763937	-1.951492	2.616858
C	-2.076041	0.325334	2.085600
H	-1.413888	0.986363	1.513904
H	-2.869311	0.949875	2.529218
H	-1.506512	-0.104345	2.919766
H	1.357006	3.910832	-0.573134
H	1.125758	3.037184	0.938829
H	3.021692	2.181706	-1.326498
C	2.514852	-0.209474	-0.145222
H	2.787990	-0.178883	-1.215193
C	2.965365	-1.545202	0.450139
H	4.060938	-1.616634	0.492042
H	2.594340	-1.658240	1.478537
C	2.431508	-2.728137	-0.424274
H	2.944126	-2.731453	-1.395263
H	2.668262	-3.669683	0.099719
C	0.956883	-2.629576	-0.598449
H	0.610045	1.289139	-1.485469
H	1.112958	-2.123593	-2.686266
C	3.624723	1.053756	1.720182
H	3.996301	1.981672	2.156341
H	3.780823	0.148290	2.305644
C	0.077557	-2.787784	0.588785
H	-0.640735	-3.609536	0.443286
H	0.640792	-2.976844	1.506964
H	-0.545063	-1.881570	0.710671
H	-1.305321	-1.914309	-3.098965

H	-3.565979	-1.351968	-0.588164
H	-3.294897	-2.846852	1.161496
H	1.405955	-0.183136	-0.145747

B1* (G781.682987)

C	-0.510959	-2.426976	-0.571000
C	-1.663341	-1.765090	-0.839233
C	1.969246	-0.570409	1.211194
C	2.591966	-0.921528	-0.088696
C	2.077832	-2.171752	-0.869342
C	0.676401	-2.267579	-1.511268
H	2.809473	-2.291460	-1.682762
H	2.229695	-3.036777	-0.206914
H	0.504107	-1.423670	-2.194783
H	0.723479	-3.166081	-2.152259
C	-0.351655	-3.418350	0.557152
H	-1.276900	-3.575106	1.120065
H	-0.031060	-4.394325	0.160679
H	0.427650	-3.108008	1.274304
C	2.698842	0.420333	-1.004023
C	-2.943393	-1.764972	-0.047486
C	1.472083	1.215835	-1.292416
H	3.473931	1.043098	-0.542941
C	-3.692635	-0.405246	-0.095388
C	-2.905695	0.777194	0.458848
H	-4.627526	-0.519040	0.470601
C	2.845858	-0.503320	2.410353
H	2.394682	0.031402	3.252517
H	3.051850	-1.541202	2.735346
C	0.593794	-0.160885	1.284983
H	-0.104895	-0.802035	0.707424
H	0.218803	0.088400	2.280264
H	0.550854	0.740253	0.601203
H	-3.623768	-2.537338	-0.448509
H	-2.752566	-2.031129	1.002501
H	-3.978196	-0.200294	-1.140559
C	-2.002915	1.483970	-0.536887
H	-2.588311	1.697864	-1.448570
C	-1.302299	2.775290	-0.097464
H	-2.026647	3.600606	-0.065321
H	-0.905935	2.676630	0.925300
C	-0.131134	3.141104	-1.057509
H	-0.425940	2.918497	-2.093351
H	0.065635	4.221725	-0.997739
C	1.134103	2.396345	-0.687917
H	-1.677885	-1.152113	-1.747572
H	0.808839	0.843529	-2.076619
C	-3.021707	1.137519	1.749491
H	-3.694973	0.602041	2.420697
H	-2.488122	1.985283	2.178404
C	1.969548	3.040768	0.393249
H	2.613230	2.338297	0.936595
H	2.622739	3.808470	-0.051495
H	1.331752	3.557122	1.122853

H	3.111870	0.030571	-1.946068
H	3.649533	-1.153825	0.111282
H	3.833003	-0.082522	2.164816
H	-1.242056	0.757625	-0.869235

B2* (G781.678549)

C	-1.167765	2.235694	-0.651012
C	0.182872	2.204828	-0.585545
C	-2.174445	-0.865169	1.108068
C	-2.661409	-0.911257	-0.281326
C	-2.976074	0.390635	-1.078078
C	-1.899036	1.349726	-1.642854
H	-3.555562	0.030887	-1.942003
H	-3.687473	0.966679	-0.467298
H	-1.179641	0.796163	-2.260540
H	-2.448848	2.004339	-2.341517
C	-2.021345	3.162149	0.186076
H	-1.426722	3.852362	0.793695
H	-2.679766	3.763137	-0.459843
H	-2.685938	2.606371	0.868877
C	-1.670880	-1.955665	-1.045978
C	1.069736	2.988878	0.355687
C	-0.233916	-1.615407	-1.250020
H	-1.788759	-2.924984	-0.546946
C	2.513187	2.442064	0.367123
C	2.606892	0.941192	0.653446
H	3.099901	2.997175	1.114952
C	-2.850486	-1.723208	2.116088
H	-2.211728	-1.975969	2.969743
H	-3.708249	-1.143212	2.510120
C	-0.984919	-0.133940	1.463774
H	-0.149722	-0.595818	0.862464
H	-0.933032	0.872544	1.012127
H	-0.727900	-0.154854	2.525103
H	1.093055	4.054726	0.071094
H	0.656338	2.960582	1.376873
H	2.978580	2.652252	-0.607855
C	3.371699	0.149272	-0.389102
H	4.420844	0.490771	-0.318986
C	3.348966	-1.390871	-0.351632
H	4.298884	-1.747781	-0.773298
H	3.331490	-1.767279	0.681493
C	2.225239	-2.038699	-1.202776
H	2.162059	-1.504749	-2.162748
H	2.549459	-3.066396	-1.445646
C	0.833948	-2.191980	-0.621297
H	0.695162	1.515544	-1.264951
H	-0.009072	-0.941791	-2.078863
C	2.115420	0.417562	1.791108
H	1.639068	1.048645	2.542590
H	2.212820	-0.637930	2.041765
C	0.704191	-3.159291	0.530644
H	0.888973	-4.187163	0.180506
H	1.461825	-2.954112	1.298897

H	-0.280115	-3.143647	1.011974
H	-2.154551	-2.039542	-2.031509
H	-3.612722	-1.465897	-0.272652
H	-3.288279	-2.626643	1.668750
H	3.048370	0.486508	-1.390725

B2*-B3*-TS (G781.677298, T-136)

C	-1.550214	1.673835	-0.884470
C	-0.244884	2.001334	-0.783899
C	-2.185821	-0.864414	1.110756
C	-2.297238	-1.513552	-0.213007
C	-2.863088	-0.532847	-1.331985
C	-1.995284	0.616598	-1.878527
H	-3.112473	-1.204338	-2.166433
H	-3.818290	-0.133788	-0.961332
H	-1.128471	0.218239	-2.420962
H	-2.628096	1.099660	-2.645682
C	-2.642695	2.365527	-0.103064
H	-2.259977	3.130320	0.580593
H	-3.351666	2.854870	-0.789949
H	-3.244099	1.657987	0.489823
C	-1.084010	-2.403908	-0.643879
C	0.363879	3.046315	0.126955
C	0.251913	-1.793036	-0.958493
H	-0.980050	-3.186738	0.119809
C	1.895938	2.887509	0.233218
C	2.336719	1.475521	0.616026
H	2.279966	3.607946	0.971805
C	-3.416088	-0.709565	1.923969
H	-3.378628	0.171383	2.576710
H	-4.339633	-0.733019	1.334411
C	-0.912329	-0.450023	1.677931
H	-0.121123	-1.182803	1.468423
H	-0.534730	0.413984	1.079405
H	-0.961474	-0.170104	2.734039
H	0.136139	4.060820	-0.242252
H	-0.082053	2.986846	1.133216
H	2.349663	3.159661	-0.731498
C	3.317296	0.836163	-0.343846
H	4.250270	1.422315	-0.242503
C	3.664499	-0.659092	-0.227553
H	4.677974	-0.790261	-0.632025
H	3.729773	-0.973171	0.824621
C	2.733223	-1.602503	-1.036622
H	2.503490	-1.124960	-2.001150
H	3.315640	-2.509407	-1.276307
C	1.450218	-2.091865	-0.391633
H	0.453581	1.466529	-1.435381
H	0.278680	-1.129582	-1.825099
C	1.924270	0.914061	1.770354
H	1.303801	1.468262	2.476027
H	2.234129	-0.083674	2.077314
C	1.641272	-3.027458	0.781338
H	2.204790	-3.918064	0.463179

H	2.244123	-2.551985	1.568929
H	0.707979	-3.375165	1.239079
H	-1.439266	-2.919628	-1.553501
H	-3.155737	-2.204669	-0.101949
H	-3.443399	-1.586153	2.603216
H	2.983710	1.047090	-1.375979

B3* (G781.704010)

C	-2.003589	0.830356	-1.064043
C	-0.938199	0.987503	-0.173984
C	-1.747370	-0.506716	1.117133
C	-1.759348	-1.766367	0.255718
C	-2.555329	-1.605950	-1.053839
C	-2.060949	-0.399088	-1.914105
H	-2.477933	-2.522779	-1.653372
H	-3.623260	-1.464116	-0.837113
H	-1.068824	-0.625679	-2.319104
H	-2.756491	-0.258604	-2.751961
C	-3.151589	1.783462	-1.163338
H	-3.279453	2.423725	-0.285776
H	-2.969975	2.443987	-2.029731
H	-4.089531	1.252949	-1.376684
C	-0.404902	-2.530240	0.103171
C	-0.625927	2.266437	0.570054
C	0.720865	-1.868317	-0.653039
H	-0.067493	-2.834899	1.101692
C	0.722031	2.858724	0.074547
C	1.902086	1.908950	0.288496
H	0.891553	3.798940	0.619122
C	-3.091116	0.012818	1.583392
H	-3.020102	1.056163	1.914086
H	-3.889316	-0.072517	0.840481
C	-0.691002	-0.449449	2.197233
H	-0.814519	-1.356159	2.812997
H	0.331704	-0.444728	1.809979
H	-0.832766	0.407902	2.862535
H	-1.422723	3.011752	0.448693
H	-0.531572	2.078233	1.647324
H	0.634219	3.120745	-0.990678
C	2.576626	1.425532	-0.983514
H	3.055481	2.314666	-1.432949
C	3.606771	0.280965	-0.902090
H	4.393249	0.466814	-1.647378
H	4.122565	0.280941	0.068699
C	3.025151	-1.120865	-1.210674
H	2.593472	-1.098509	-2.223692
H	3.867833	-1.832355	-1.246758
C	1.994071	-1.664511	-0.237414
H	-0.070792	0.339504	-0.353671
H	0.520608	-1.642689	-1.704781
C	2.303269	1.610296	1.536251
H	1.811119	2.046059	2.407688
H	3.154601	0.965895	1.741393
C	2.528296	-2.056251	1.120533

H	3.240206	-2.888659	1.006377
H	3.086532	-1.232935	1.584584
H	1.757108	-2.378071	1.828320
H	-0.660276	-3.468914	-0.418014
H	-2.382497	-2.444180	0.877023
H	-3.389729	-0.575647	2.466316
H	1.787934	1.149438	-1.708588

B3* (G781.704011)

C	-2.003620	0.830182	-1.064079
C	-0.938355	0.987329	-0.173826
C	-1.747407	-0.506900	1.117047
C	-1.759136	-1.766503	0.255525
C	-2.555114	-1.606083	-1.054041
C	-2.060739	-0.399167	-1.914235
H	-2.477638	-2.522864	-1.653639
H	-3.623051	-1.464332	-0.837303
H	-1.068541	-0.625694	-2.319082
H	-2.756175	-0.258698	-2.752181
C	-3.151565	1.783363	-1.163342
H	-3.280467	2.422185	-0.284859
H	-2.968689	2.445501	-2.028253
H	-4.089241	1.253279	-1.378772
C	-0.404554	-2.530118	0.102953
C	-0.626281	2.266336	0.570221
C	0.721188	-1.868013	-0.653146
H	-0.067130	-2.834808	1.101460
C	0.721620	2.858734	0.074653
C	1.901735	1.909033	0.288615
H	0.891081	3.798999	0.619162
C	-3.091289	0.012384	1.583273
H	-3.020385	1.055640	1.914271
H	-3.889396	-0.072788	0.840236
C	-0.691174	-0.449679	2.197365
H	-0.814499	-1.356603	2.812830
H	0.331553	-0.444569	1.810173
H	-0.833253	0.407480	2.862842
H	-1.423172	3.011539	0.448740
H	-0.531971	2.078205	1.647501
H	0.633727	3.120675	-0.990585
C	2.576425	1.425813	-0.983388
H	3.055094	2.315058	-1.432793
C	3.606805	0.281476	-0.901932
H	4.393298	0.467486	-1.647165
H	4.122531	0.281552	0.068892
C	3.025512	-1.120491	-1.210566
H	2.593991	-1.098244	-2.223652
H	3.868362	-1.831786	-1.246496
C	1.994384	-1.664277	-0.237440
H	-0.070836	0.339483	-0.353504
H	0.520996	-1.642240	-1.704867
C	2.302773	1.610201	1.536368
H	1.810503	2.045811	2.407812
H	3.154073	0.965761	1.741511

C	2.528568	-2.056271	1.120453
H	3.239729	-2.889317	1.006234
H	3.087641	-1.233379	1.584250
H	1.757275	-2.377341	1.828463
H	-0.659750	-3.468799	-0.418313
H	-2.382186	-2.444494	0.876718
H	-3.389956	-0.576365	2.465986
H	1.787806	1.149559	-1.708489

B3*-C1*-TS (G781.705822, T-309)

C	-1.939781	0.852485	-1.074863
C	-0.930667	0.925660	-0.096181
C	-1.762018	-0.464202	1.100784
C	-1.796456	-1.739932	0.249045
C	-2.556379	-1.561908	-1.077891
C	-2.014124	-0.364788	-1.931850
H	-2.484073	-2.476590	-1.681468
H	-3.625684	-1.393760	-0.889924
H	-1.016939	-0.619956	-2.307469
H	-2.685738	-0.206503	-2.785270
C	-3.012664	1.876198	-1.236758
H	-3.176075	2.497321	-0.351306
H	-2.707154	2.552159	-2.056453
H	-3.957437	1.416731	-1.556500
C	-0.457400	-2.536838	0.117862
C	-0.590007	2.204705	0.644725
C	0.683134	-1.910447	-0.646198
H	-0.130971	-2.830895	1.122850
C	0.722655	2.815121	0.083986
C	1.930438	1.895177	0.276005
H	0.892848	3.769984	0.602178
C	-3.115541	0.072152	1.539519
H	-3.032003	1.103263	1.904471
H	-3.884491	0.036121	0.761436
C	-0.760572	-0.479451	2.243140
H	-0.951786	-1.390564	2.832843
H	0.279636	-0.505800	1.907564
H	-0.900699	0.371334	2.917515
H	-1.399497	2.943301	0.577967
H	-0.433828	2.003848	1.710999
H	0.590270	3.052788	-0.982830
C	2.562367	1.376968	-1.005052
H	3.034023	2.250853	-1.490529
C	3.586494	0.225945	-0.923745
H	4.365468	0.395388	-1.680646
H	4.113507	0.230926	0.040723
C	2.988823	-1.174591	-1.206450
H	2.556305	-1.166264	-2.219334
H	3.823236	-1.896350	-1.229353
C	1.951481	-1.686923	-0.222951
H	-0.064778	0.274034	-0.285439
H	0.499266	-1.730944	-1.710038
C	2.393769	1.655256	1.514834
H	1.930206	2.113560	2.390422

H	3.271169	1.041750	1.706028
C	2.467051	-2.014063	1.158458
H	3.287859	-2.743408	1.081522
H	2.885241	-1.125051	1.648829
H	1.709771	-2.438251	1.826106
H	-0.730143	-3.477777	-0.390156
H	-2.444752	-2.402181	0.858643
H	-3.467365	-0.536447	2.387248
H	1.750982	1.088232	-1.699153

C1* (G781.714962)

C	-1.641723	1.013732	-1.099368
C	-0.960332	0.764533	0.161149
C	-1.791179	-0.322364	1.030334
C	-1.974240	-1.618560	0.188169
C	-2.658953	-1.333964	-1.159120
C	-2.038393	-0.141691	-1.934955
H	-2.626801	-2.224390	-1.801532
H	-3.721575	-1.101868	-1.003432
H	-1.077532	-0.477051	-2.378416
H	-2.672163	0.170533	-2.775715
C	-1.987917	2.359451	-1.612968
H	-1.514913	3.196202	-1.098300
H	-1.816041	2.422614	-2.698098
H	-3.085862	2.458336	-1.497382
C	-0.699259	-2.509632	0.042262
C	-0.486800	2.008218	0.936844
C	0.463697	-1.926362	-0.715583
H	-0.385980	-2.841266	1.038588
C	0.793566	2.642764	0.351256
C	2.002682	1.709722	0.429730
H	1.000884	3.566266	0.911399
C	-3.160527	0.297319	1.391670
H	-3.032395	1.226259	1.961947
H	-3.765858	0.531559	0.504682
C	-1.026004	-0.633565	2.332919
H	-1.444453	-1.540306	2.790726
H	0.043347	-0.794576	2.162265
H	-1.135775	0.172811	3.067011
H	-1.292555	2.754548	0.997929
H	-0.268293	1.717719	1.969067
H	0.633905	2.945919	-0.695032
C	2.574618	1.265713	-0.907247
H	3.097580	2.143286	-1.329050
C	3.516726	0.045171	-0.940430
H	4.277082	0.202687	-1.718252
H	4.075274	-0.051559	0.000905
C	2.799438	-1.283886	-1.279097
H	2.346582	-1.190299	-2.278650
H	3.568023	-2.072513	-1.354843
C	1.749663	-1.756996	-0.293587
H	-0.062485	0.130319	-0.126926
H	0.295225	-1.756564	-1.785002
C	2.540211	1.413599	1.626770

H	2.125811	1.824255	2.549048
H	3.433313	0.801529	1.736139
C	2.241142	-2.106317	1.087510
H	3.173832	-2.684573	1.018334
H	2.477660	-1.195270	1.655267
H	1.523381	-2.689903	1.672671
H	-1.025905	-3.418117	-0.492314
H	-2.679455	-2.240351	0.766647
H	-3.737435	-0.396035	2.018340
H	1.735082	1.088499	-1.605575

C1* (G781.714959)

C	-1.641824	1.013678	-1.099374
C	-0.960479	0.764517	0.161158
C	-1.791226	-0.322512	1.030279
C	-1.974110	-1.618689	0.188041
C	-2.658852	-1.334110	-1.159235
C	-2.038417	-0.141738	-1.935022
H	-2.626597	-2.224506	-1.801683
H	-3.721498	-1.102141	-1.003534
H	-1.077580	-0.476993	-2.378597
H	-2.672273	0.170510	-2.775712
C	-1.988145	2.359410	-1.612869
H	-1.514630	3.196094	-1.098543
H	-1.817139	2.422553	-2.698120
H	-3.085966	2.458487	-1.496254
C	-0.698994	-2.509542	0.042097
C	-0.487042	2.008273	0.936819
C	0.463921	-1.926078	-0.715680
H	-0.385695	-2.841220	1.038402
C	0.793435	2.642744	0.351389
C	2.002504	1.709639	0.429922
H	1.000744	3.566216	0.911588
C	-1.026123	-0.633778	2.332897
H	-1.136140	0.172438	3.067124
H	-1.444445	-1.540687	2.790488
C	-3.160667	0.296971	1.391647
H	-3.032657	1.225864	1.962031
H	-3.766004	0.531239	0.504673
H	-3.737506	-0.396524	2.018222
H	-1.292789	2.754629	0.997712
H	-0.268691	1.717851	1.969091
H	0.633915	2.945947	-0.694906
C	2.574827	1.266055	-0.907028
H	3.097988	2.143735	-1.328359
C	3.516903	0.045504	-0.940311
H	4.277268	0.203085	-1.718115
H	4.075439	-0.051324	0.001027
C	2.799627	-1.283511	-1.279171
H	2.346740	-1.189759	-2.278695
H	3.568239	-2.072094	-1.355067
C	1.749923	-1.756833	-0.293713
H	-0.062523	0.130390	-0.126926
H	0.295418	-1.756074	-1.785061

C	2.539743	1.413150	1.627001
H	2.125137	1.823519	2.549312
H	3.432804	0.801016	1.736372
C	2.241439	-2.106261	1.087353
H	3.174696	-2.683584	1.018200
H	2.476935	-1.195177	1.655493
H	1.524077	-2.690736	1.672119
H	-1.025470	-3.418033	-0.492579
H	-2.679239	-2.240621	0.766468
H	0.043275	-0.794548	2.162335
H	1.735517	1.089119	-1.605700

C1*-E*-TS (G781.706368, T-980)

C	-1.706454	1.191798	-0.999385
C	-1.091554	0.851845	0.221251
C	-1.798554	-0.228023	1.107865
C	-2.250463	-1.407516	0.193457
C	-3.192217	-0.920581	-0.919013
C	-2.585753	0.225441	-1.741090
H	-3.446878	-1.754963	-1.587720
H	-4.139484	-0.584145	-0.477002
H	-1.950278	-0.181971	-2.554754
H	-3.363508	0.789952	-2.279552
C	-1.479373	2.486696	-1.720621
H	-0.706597	3.128906	-1.296477
H	-1.264210	2.314223	-2.786053
H	-2.431006	3.046311	-1.698061
C	-1.044191	-2.235110	-0.351587
C	-0.197885	1.899053	0.908062
C	0.106577	-1.437278	-0.954962
H	-0.669646	-2.869928	0.458600
C	1.175707	2.098126	0.221760
C	2.351300	1.255695	0.694583
H	1.480007	3.154878	0.325327
C	-0.872670	-0.764066	2.225027
H	-0.767542	-0.040306	3.042141
H	-1.313024	-1.671383	2.659317
C	-3.015603	0.451120	1.789114
H	-2.682167	1.266965	2.444202
H	-3.717908	0.877291	1.062434
H	-3.557412	-0.275010	2.410939
H	-0.734835	2.858143	0.923008
H	-0.055575	1.633008	1.959621
H	1.064042	1.945126	-0.863211
C	3.486341	1.121984	-0.318245
H	4.424981	1.474234	0.134998
C	3.727304	-0.308653	-0.846599
H	4.538622	-0.265713	-1.587450
H	4.094702	-0.950191	-0.033018
C	2.501199	-0.963271	-1.526153
H	2.028982	-0.260908	-2.230752
H	2.872997	-1.803966	-2.143519
C	1.456184	-1.587241	-0.632952
H	-0.196037	-0.079848	-0.418154

H	-0.064640	-1.056101	-1.971857
C	2.467511	0.747017	1.933604
H	1.697040	0.850607	2.695493
H	3.379037	0.232619	2.243209
C	1.955963	-2.419530	0.509107
H	2.794800	-3.043428	0.166760
H	2.356506	-1.770943	1.300858
H	1.194447	-3.067870	0.948756
H	-1.424941	-2.919162	-1.126610
H	-2.825497	-2.101390	0.827944
H	0.132180	-1.004867	1.867657
H	3.292627	1.784795	-1.176398

E* (G781.714888)

C	-1.467029	1.013082	-1.192667
C	-1.267010	0.913116	0.166748
C	-1.918799	-0.240719	0.965842
C	-2.044727	-1.529842	0.087752
C	-2.757759	-1.232581	-1.240247
C	-2.115945	-0.073618	-2.017893
H	-2.778882	-2.136446	-1.866801
H	-3.807179	-0.984912	-1.032363
H	-1.351587	-0.450076	-2.728215
H	-2.865939	0.396259	-2.674913
C	-1.044034	2.215853	-2.006085
H	-1.066696	3.151845	-1.436983
H	-0.022934	2.093842	-2.403835
H	-1.700454	2.335333	-2.877770
C	-0.702113	-2.281048	-0.101592
C	-0.672575	2.076485	0.962867
C	0.428775	-1.458104	-0.739830
H	-0.377964	-2.675648	0.868156
C	0.770725	2.536024	0.632253
C	1.862287	1.471014	0.722828
H	1.016535	3.345622	1.337630
C	-1.151652	-0.593841	2.265490
H	-1.287624	0.168252	3.041920
H	-1.543815	-1.534208	2.676368
C	-3.334272	0.250263	1.389078
H	-3.256142	1.139238	2.028936
H	-3.953692	0.519115	0.525912
H	-3.853772	-0.530399	1.963008
H	-1.328107	2.953943	0.829436
H	-0.716540	1.844490	2.032392
H	0.805824	2.994068	-0.363003
C	2.736993	1.359761	-0.514647
H	3.370631	2.264633	-0.546897
C	3.639562	0.129065	-0.659648
H	4.446193	0.346670	-1.372666
H	4.128814	-0.131516	0.288386
C	2.883168	-1.112134	-1.242869
H	2.482007	-0.843871	-2.229600
H	3.632220	-1.911211	-1.380482
C	1.809909	-1.654135	-0.368128

H	0.240746	-0.353934	-0.458775
H	0.338497	-1.379917	-1.835080
C	2.090602	0.793476	1.862568
H	1.488698	0.970116	2.753504
H	2.911522	0.088343	1.976007
C	2.205739	-2.422190	0.839782
H	3.255343	-2.272769	1.111818
H	1.557609	-2.226334	1.702060
H	2.068847	-3.494057	0.598614
H	-0.883228	-3.158054	-0.742112
H	-2.684951	-2.226313	0.654666
H	-0.073395	-0.704280	2.103432
H	2.098352	1.442619	-1.412346

E* (G781.714890)

C	-1.467347	1.013160	-1.192503
C	-1.267152	0.913029	0.166887
C	-1.918748	-0.241034	0.965852
C	-2.044383	-1.530125	0.087673
C	-2.757374	-1.232953	-1.240373
C	-2.115915	-0.073663	-2.017837
H	-2.778083	-2.136757	-1.867029
H	-3.806914	-0.985702	-1.032598
H	-1.351401	-0.449761	-2.728180
H	-2.866048	0.396040	-2.674825
C	-1.044951	2.216187	-2.005834
H	-1.702032	2.335886	-2.876999
H	-1.067163	3.152029	-1.436498
H	-0.024170	2.094232	-2.404448
C	-0.701671	-2.281126	-0.101598
C	-0.672791	2.076361	0.963145
C	0.429098	-1.458007	-0.739841
H	-0.377470	-2.675621	0.868172
C	0.770402	2.536050	0.632329
C	1.862149	1.471225	0.722848
H	1.016236	3.345793	1.337537
C	-1.151661	-0.594059	2.265544
H	-1.287863	0.167978	3.041990
H	-1.543668	-1.534520	2.676361
C	-3.334356	0.249645	1.388957
H	-3.256480	1.138658	2.028796
H	-3.953762	0.518322	0.525725
H	-3.853731	-0.531115	1.962867
H	-1.328468	2.953740	0.829931
H	-0.716575	1.844195	2.032636
H	0.805324	2.993937	-0.363008
C	2.736550	1.359992	-0.514862
H	3.369872	2.265066	-0.547444
C	3.639486	0.129567	-0.659835
H	4.446038	0.347339	-1.372892
H	4.128821	-0.130790	0.288213
C	2.883483	-1.111972	-1.242947
H	2.482329	-0.843939	-2.229742
H	3.632782	-1.910837	-1.380368

C	1.810256	-1.654040	-0.368218
H	0.240976	-0.353883	-0.458536
H	0.338730	-1.379669	-1.835061
C	2.090817	0.793883	1.862629
H	1.489053	0.970545	2.753653
H	2.911878	0.088910	1.976061
C	2.206084	-2.422161	0.839645
H	2.069231	-3.494018	0.598404
H	3.255673	-2.272731	1.111740
H	1.557925	-2.226354	1.701919
H	-0.882613	-3.158204	-0.742077
H	-2.684558	-2.226698	0.654524
H	-0.073359	-0.704302	2.103596
H	2.097619	1.442470	-1.412389

E*-F*-TS (G781.711380, T-103)

C	-1.604659	1.196961	-1.008992
C	-1.366958	0.914700	0.309127
C	-2.098910	-0.301679	0.932870
C	-2.030445	-1.519033	-0.051214
C	-2.681958	-1.157516	-1.399230
C	-2.232790	0.205575	-1.969246
H	-2.483565	-1.951011	-2.135569
H	-3.770566	-1.145251	-1.260835
H	-1.486866	0.054481	-2.774779
H	-3.078985	0.690132	-2.483662
C	-1.221173	2.494147	-1.687223
H	-2.038589	2.813578	-2.350374
H	-1.025473	3.317420	-0.992118
H	-0.338780	2.375216	-2.339700
C	-0.615363	-2.144581	-0.192243
C	-0.539920	1.867257	1.178566
C	0.440607	-1.253332	-0.870282
H	-0.265934	-2.441449	0.804871
C	0.888971	2.164407	0.663198
C	2.004504	1.168164	0.875585
H	1.251217	3.099775	1.134067
C	-1.603773	-0.739284	2.329591
H	-1.717042	0.057771	3.074765
H	-2.217202	-1.583456	2.672726
C	-3.583701	0.129874	1.136972
H	-3.640508	0.938136	1.879029
H	-4.044414	0.502452	0.215834
H	-4.183105	-0.712803	1.511342
H	-1.060229	2.836628	1.246777
H	-0.483768	1.502216	2.210667
H	0.866085	2.404575	-0.408833
C	3.291313	1.467429	0.133915
H	4.023261	1.911984	0.829314
C	3.917112	0.211927	-0.509229
H	4.725281	0.513205	-1.188573
H	4.378855	-0.432730	0.251769
C	2.856595	-0.585171	-1.308355
H	2.334661	0.087044	-2.005219

H	3.391094	-1.338510	-1.917435
C	1.861589	-1.364316	-0.496989
H	0.152236	-0.186435	-0.744994
H	0.407584	-1.354859	-1.974181
C	1.928000	0.063378	1.664034
H	1.026997	-0.178037	2.217844
H	2.818527	-0.511788	1.906837
C	2.363644	-2.545041	0.268130
H	2.271479	-3.409130	-0.418160
H	3.416169	-2.460062	0.555058
H	1.753214	-2.790586	1.142554
H	-0.714732	-3.081341	-0.762005
H	-2.645611	-2.315710	0.400835
H	-0.563090	-1.078717	2.350935
H	3.105689	2.224073	-0.642714

F* (G781.734640)

C	1.269579	1.254339	0.920400
C	1.367164	0.825871	-0.377201
C	2.154847	-0.465969	-0.672283
C	1.822346	-1.569890	0.386327
C	2.117210	-1.063951	1.814579
C	1.640011	0.380225	2.103847
H	1.671985	-1.752603	2.546751
H	3.200954	-1.110369	1.981563
H	0.770083	0.373690	2.787041
H	2.416626	0.913425	2.678673
C	0.904586	2.664926	1.332271
H	1.586078	2.993883	2.129438
H	0.985823	3.398835	0.524553
H	-0.106661	2.734286	1.771067
C	0.428823	-2.244351	0.254155
C	0.897843	1.685737	-1.557460
C	-0.792325	-1.534416	0.895830
H	0.229099	-2.472849	-0.801297
C	-0.535198	2.204982	-1.352581
C	-1.455719	1.225759	-0.707131
H	-1.013139	2.445564	-2.327508
C	1.973756	-1.036227	-2.100441
H	2.409313	-0.375493	-2.860706
H	2.507894	-1.993230	-2.168456
C	3.665625	-0.084214	-0.562148
H	3.926695	0.664437	-1.322623
H	3.923575	0.337675	0.415431
H	4.292988	-0.970498	-0.734610
H	1.572111	2.540938	-1.727195
H	0.933556	1.099641	-2.480633
H	-0.572306	3.146064	-0.790355
C	-2.495477	1.737853	0.209416
H	-2.801360	2.750936	-0.107150
C	-3.658792	0.766530	0.432939
H	-4.352582	1.186457	1.172051
H	-4.225037	0.663177	-0.507182
C	-3.133611	-0.597720	0.905678

H	-2.737489	-0.471614	1.925289
H	-3.969095	-1.305541	0.987062
C	-2.010855	-1.202761	-0.008924
H	-0.490619	-0.601313	1.383023
H	-1.189669	-2.172055	1.700838
C	-1.583249	-0.173645	-1.120820
H	-0.742972	-0.529353	-1.722494
H	-2.455086	-0.107585	-1.816354
C	-2.539994	-2.459055	-0.730462
H	-2.762516	-3.245369	0.002956
H	-3.465738	-2.237654	-1.280528
H	-1.813358	-2.865322	-1.446746
H	0.525150	-3.229515	0.732638
H	2.544809	-2.378656	0.181950
H	0.934056	-1.236914	-2.387275
H	-1.952590	1.922767	1.164838

C2* (G781.715173)

C	-1.725201	0.962749	-1.094438
C	-1.042722	0.741268	0.168524
C	-1.767865	-0.424818	1.016604
C	-1.851231	-1.713439	0.148201
C	-2.577360	-1.459067	-1.184317
C	-2.086088	-0.199746	-1.938467
H	-2.476367	-2.329427	-1.846698
H	-3.654056	-1.330342	-1.006492
H	-1.129509	-0.437189	-2.451318
H	-2.779182	0.095167	-2.738710
C	-2.096538	2.299955	-1.614341
H	-1.581496	3.142320	-1.150688
H	-2.012394	2.343141	-2.709412
H	-3.179624	2.412534	-1.403506
C	-0.503972	-2.480496	-0.030014
C	-0.659791	2.003848	0.967775
C	0.608584	-1.768167	-0.753606
H	-0.162300	-2.827341	0.951927
C	0.633824	2.672273	0.457130
C	1.840021	1.734098	0.522617
H	0.822066	3.568677	1.066656
C	-0.966016	-0.703189	2.305420
H	-1.156839	0.057510	3.071326
H	-1.279610	-1.666739	2.729236
C	-3.179265	0.074605	1.402564
H	-3.120202	0.997387	1.994105
H	-3.807953	0.281266	0.524823
H	-3.694017	-0.677007	2.016163
H	-1.490504	2.724576	0.977507
H	-0.500655	1.721900	2.012986
H	0.506583	3.026113	-0.577606
C	2.520116	1.466683	-0.808505
H	3.002010	2.414302	-1.111056
C	3.545012	0.321949	-0.898774
H	4.287225	0.572966	-1.669752
H	4.115027	0.228997	0.036447

C	2.927015	-1.039697	-1.303701
H	2.477389	-0.929143	-2.302991
H	3.750794	-1.766661	-1.407625
C	1.904307	-1.627938	-0.353666
H	-0.091169	0.188315	-0.136026
H	0.405367	-1.488324	-1.793302
C	2.264488	1.265476	1.709293
H	1.770412	1.549106	2.639695
H	3.133770	0.620994	1.809288
C	2.446226	-2.170709	0.944984
H	2.896772	-3.159171	0.757300
H	3.246334	-1.536837	1.344391
H	1.688926	-2.299599	1.724902
H	-0.746067	-3.393361	-0.601968
H	-2.491743	-2.407623	0.719352
H	0.113982	-0.734994	2.124313
H	1.735764	1.304660	-1.572959

C2*-D1*-TS* (G781.681589, T-456)

C	1.274946	0.930134	1.218685
C	1.196924	0.841519	-0.162983
C	1.965758	-0.329657	-0.854033
C	1.876744	-1.635281	0.010856
C	2.434068	-1.394390	1.423277
C	1.840409	-0.150466	2.104198
H	2.257521	-2.279159	2.050759
H	3.524272	-1.279420	1.363019
H	1.056959	-0.422876	2.835645
H	2.608926	0.336441	2.730241
C	0.844263	2.149935	2.003788
H	1.032633	3.091863	1.476033
H	-0.228149	2.111755	2.244160
H	1.370664	2.191438	2.964779
C	0.494032	-2.356624	0.040674
C	0.916854	2.141232	-0.944084
C	-0.663441	-1.620409	0.670174
H	0.227443	-2.690363	-0.971190
C	-0.532579	2.724869	-0.778631
C	-1.568900	1.636134	-0.774089
H	-0.730772	3.388393	-1.636072
C	1.505973	-0.667255	-2.290985
H	1.570749	0.194527	-2.968299
H	2.176190	-1.434925	-2.699525
C	3.449753	0.129585	-0.969401
H	3.533634	0.987037	-1.651552
H	3.866735	0.438058	-0.003312
H	4.074909	-0.678140	-1.375119
H	1.619911	2.928960	-0.638568
H	1.108118	1.972984	-2.009859
H	-0.628328	3.342606	0.120468
C	-2.626466	1.612072	0.292035
H	-3.208652	2.540724	0.136036
C	-3.577749	0.399664	0.378878
H	-4.455127	0.727303	0.954046

H	-3.962196	0.144317	-0.620733
C	-3.001510	-0.855832	1.091636
H	-2.575426	-0.539572	2.057405
H	-3.855222	-1.512849	1.325921
C	-1.970742	-1.659605	0.313891
H	-0.431912	0.277509	-0.535953
H	-0.450579	-1.057223	1.580123
C	-1.390303	0.597661	-1.667433
H	-0.697462	0.716982	-2.497008
H	-2.091342	-0.225431	-1.737689
C	-2.512345	-2.506575	-0.814914
H	-2.922407	-3.444437	-0.406886
H	-3.346795	-2.016615	-1.338698
H	-1.753314	-2.783833	-1.556230
H	0.658335	-3.286735	0.613722
H	2.554035	-2.354985	-0.478921
H	0.491736	-1.075499	-2.340081
H	-2.129221	1.770892	1.264103

D1* (G781.718194)

C	-1.756774	1.275132	-0.860641
C	-1.375966	0.857939	0.383761
C	-1.985402	-0.426405	0.975965
C	-2.107440	-1.491546	-0.148288
C	-2.969537	-0.976647	-1.311447
C	-2.531231	0.404459	-1.827593
H	-2.957983	-1.706120	-2.134783
H	-4.012139	-0.924868	-0.970823
H	-1.890600	0.292435	-2.724638
H	-3.406541	0.963677	-2.197938
C	-1.417750	2.627052	-1.453623
H	-1.050961	3.359658	-0.726834
H	-0.674020	2.545922	-2.264505
H	-2.317589	3.054465	-1.920583
C	-0.712676	-2.028547	-0.575813
C	-0.456179	1.723873	1.231814
C	0.380236	-1.081840	-0.982063
H	-0.345119	-2.716593	0.193681
C	0.933395	1.944574	0.579330
C	2.070516	0.996696	0.736022
H	1.366741	2.864580	1.032302
C	-1.184483	-1.027254	2.153051
H	-1.210486	-0.384606	3.042399
H	-1.630089	-1.988203	2.443513
C	-3.391132	-0.057820	1.536369
H	-3.291575	0.663022	2.359622
H	-4.033683	0.401334	0.777326
H	-3.896469	-0.951658	1.929946
H	-0.898820	2.721148	1.368379
H	-0.335165	1.317225	2.241498
H	0.830355	2.190401	-0.483597
C	3.247877	1.279731	-0.150053
H	4.007124	1.749619	0.503339
C	3.887111	0.057044	-0.832123

H	4.678263	0.396903	-1.513404
H	4.370574	-0.592519	-0.088986
C	2.784215	-0.694236	-1.588580
H	2.333539	-0.030222	-2.342188
H	3.233316	-1.538889	-2.138763
C	1.716727	-1.257209	-0.672169
H	3.164121	-0.373901	2.007121
H	0.149368	-0.350870	-1.759814
C	2.311529	0.310544	2.034735
H	1.428238	-0.204906	2.422403
H	2.548109	1.100259	2.773322
C	2.164887	-2.368464	0.258422
H	2.088257	-3.335706	-0.262690
H	3.212557	-2.257232	0.558221
H	1.561382	-2.446755	1.169987
H	-0.873956	-2.657510	-1.473862
H	-2.630034	-2.365373	0.276554
H	-0.136095	-1.215917	1.896377
H	2.971848	2.018746	-0.915038

D2* (G781.696824)

C	1.439173	1.077578	1.132562
C	1.316170	0.892636	-0.210313
C	1.994490	-0.327777	-0.888311
C	1.993328	-1.563336	0.068211
C	2.624170	-1.214352	1.426390
C	2.017645	0.039836	2.076634
H	2.542923	-2.071701	2.111142
H	3.699570	-1.053448	1.273627
H	1.226308	-0.239830	2.800372
H	2.779763	0.530104	2.704682
C	1.011889	2.343167	1.853201
H	1.092072	3.244280	1.233286
H	-0.024616	2.281040	2.226482
H	1.641956	2.503634	2.738241
C	0.611756	-2.268336	0.195605
C	0.768714	2.008888	-1.108441
C	-0.540286	-1.466480	0.715690
H	0.342597	-2.764394	-0.745785
C	-0.673057	2.511163	-0.814550
C	-1.765119	1.474566	-0.805978
H	-0.943766	3.228433	-1.611705
C	1.384158	-0.768814	-2.241523
H	1.468976	0.006750	-3.012939
H	1.942949	-1.638708	-2.612958
C	3.461169	0.098495	-1.196539
H	3.471850	0.925300	-1.920164
H	3.985940	0.444559	-0.299252
H	4.027575	-0.735681	-1.635923
H	1.418816	2.895476	-1.032324
H	0.823305	1.709411	-2.161305
H	-0.710955	3.077583	0.121177
C	-2.799662	1.565283	0.287163
H	-3.428078	2.444446	0.050723

C	-3.678471	0.323277	0.533893
H	-4.491734	0.621869	1.209126
H	-4.170934	-0.005129	-0.393337
C	-2.926338	-0.846454	1.214738
H	-2.457625	-0.466585	2.135388
H	-3.670010	-1.596513	1.526471
C	-1.875491	-1.586043	0.382912
H	-1.296165	-0.420094	-0.573592
H	-0.325149	-0.743588	1.503111
C	-1.790378	0.454330	-1.732047
H	-1.061740	0.436515	-2.538338
H	-2.693342	-0.134908	-1.889445
C	-2.402177	-2.609699	-0.599597
H	-2.628010	-3.541390	-0.059367
H	-3.339278	-2.273162	-1.061195
H	-1.687342	-2.851523	-1.393825
H	0.739174	-3.090536	0.925928
H	2.642446	-2.321960	-0.401132
H	0.332805	-1.069364	-2.176648
H	-2.280371	1.841553	1.221278

D2*-E*-TS (G781.698028, T-815)

C	-1.495272	1.008386	-1.163629
C	-1.376481	0.862823	0.186588
C	-1.982509	-0.378944	0.888839
C	-1.898076	-1.631323	-0.038472
C	-2.534619	-1.356590	-1.410672
C	-1.993348	-0.086866	-2.086913
H	-2.397235	-2.225724	-2.071497
H	-3.618868	-1.253793	-1.271593
H	-1.172295	-0.338291	-2.789043
H	-2.769435	0.337360	-2.745197
C	-1.141291	2.280736	-1.908824
H	-1.219900	3.183299	-1.292565
H	-0.120826	2.246826	-2.326706
H	-1.814319	2.412389	-2.767278
C	-0.477024	-2.248822	-0.134740
C	-0.891030	2.022089	1.059052
C	0.644521	-1.379963	-0.659859
H	-0.193794	-2.688714	0.829618
C	0.544753	2.546236	0.786893
C	1.652764	1.507219	0.803013
H	0.780518	3.295517	1.560739
C	-1.340427	-0.740875	2.250548
H	-1.520678	0.026408	3.013064
H	-1.795173	-1.668446	2.625204
C	-3.473390	-0.040671	1.184815
H	-3.537690	0.810157	1.876756
H	-4.020838	0.236298	0.277054
H	-3.982515	-0.893946	1.656667
H	-1.567986	2.883048	0.936311
H	-0.964749	1.751797	2.118694
H	0.581689	3.081553	-0.168104
C	2.620301	1.575200	-0.365309

H	3.153749	2.537311	-0.268772
C	3.640925	0.437333	-0.536088
H	4.449136	0.788847	-1.191260
H	4.118168	0.180617	0.420480
C	3.046258	-0.827400	-1.216488
H	2.618583	-0.534666	-2.186263
H	3.867613	-1.534062	-1.413256
C	2.011398	-1.569413	-0.405189
H	1.051253	-0.381687	0.316544
H	0.426595	-0.755554	-1.528205
C	1.798163	0.653049	1.846319
H	1.124251	0.696511	2.698855
H	2.643077	-0.026893	1.936404
C	2.511855	-2.490833	0.667478
H	2.612071	-3.496064	0.224448
H	3.503308	-2.194120	1.028416
H	1.822109	-2.582139	1.513497
H	-0.532863	-3.097095	-0.840257
H	-2.501136	-2.419515	0.443683
H	-0.258353	-0.903395	2.193059
H	2.031703	1.677938	-1.293670

E* (G781.714891)

C	-1.468120	1.013328	-1.192298
C	-1.267338	0.912756	0.166987
C	-1.918128	-0.241645	0.966024
C	-2.044128	-1.530465	0.087494
C	-2.757665	-1.232763	-1.240093
C	-2.116029	-0.073783	-2.017764
H	-2.779277	-2.136474	-1.866861
H	-3.806952	-0.984932	-1.031636
H	-1.350879	-0.450267	-2.727259
H	-2.865699	0.395272	-2.675699
C	-1.046662	2.216979	-2.005142
H	-1.067434	3.152331	-1.434970
H	-0.026646	2.094968	-2.405724
H	-1.705240	2.337749	-2.875043
C	-0.701360	-2.281345	-0.102339
C	-0.673158	2.076182	0.963305
C	0.429268	-1.457867	-0.740120
H	-0.377326	-2.676298	0.867328
C	0.769965	2.536150	0.632506
C	1.861978	1.471555	0.722781
H	1.015644	3.345751	1.337903
C	-1.150243	-0.594906	2.265210
H	-1.286776	0.166542	3.042179
H	-1.541397	-1.535919	2.675536
C	-3.333598	0.248628	1.390257
H	-3.255434	1.137620	2.030078
H	-3.953746	0.517203	0.527528
H	-3.852237	-0.532346	1.964523
H	-1.328861	2.953541	0.830159
H	-0.716869	1.843981	2.032801
H	0.804739	2.994142	-0.362804

C	2.735812	1.360276	-0.515302
H	3.368760	2.265610	-0.548417
C	3.639248	0.130198	-0.660235
H	4.445791	0.348161	-1.373238
H	4.128615	-0.129843	0.287879
C	2.883675	-1.111599	-1.243188
H	2.482433	-0.843810	-2.230020
H	3.633206	-1.910284	-1.380573
C	1.810629	-1.653979	-0.368488
H	0.241098	-0.353634	-0.459056
H	0.339261	-1.379702	-1.835397
C	2.091181	0.794376	1.862583
H	1.489716	0.971047	2.753808
H	2.912212	0.089293	1.975714
C	2.206518	-2.422066	0.839288
H	2.069855	-3.493948	0.598058
H	3.256024	-2.272319	1.111529
H	1.558044	-2.226466	1.701405
H	-0.882175	-3.158119	-0.743231
H	-2.684112	-2.227193	0.654383
H	-0.071882	-0.704028	2.102911
H	2.096466	1.442288	-1.412579

References

[1] G. R. Fulmer, A. J. M. Miller, N. H. Sherden, H. E. Gottlieb, A. Nudelman, B. M. Stoltz, J. E. Bercaw, K. I. Goldberg, *Organometallics* **2010**, *29*, 2176.

[2] M. M. Bradford, *Anal. Biochem.* **1976**, *72*, 248-254.

[3] J. Rinkel, J. S. Dickschat, *Org. Lett*. **2019**, *21*, 2426-2429.

[4] Q. Huang, H. J. Williams, C. A. Roessner, A. I. Scott, *Tetrahedron Lett.* **2000**, *41*, 9701-9704.

[5] F. M. Hahn, A. P. Hurlburt, C. D. Poulter, *J. Bacteriol.* **1999**, *181*, 4499-4504.

[6] P. Rabe, J. Rinkel, E. Dolja, T. Schmitz, B. Nubbemeyer, T. H. Luu, J. S. Dickschat, *Angew. Chem. Int. Ed.* **2017**, *56*, 2776-2779.

[7] L. Lauterbach, J. Rinkel, J. S. Dickschat, *Angew. Chem. Int. Ed*. **2018**, *57*, 8280-8283.

[8] Z. Quan, J. S. Dickschat, *Org. Biomol. Chem.* **2020**, *18*, 6072-6076.

[9] P. Rabe, L. Barra, J. Rinkel, R. Riclea, C. A. Citron, T. A. Klapschinski, A. Janusko, J. S. Dickschat, *Angew. Chem. Int. Ed.* **2015**, *54*, 13448-13451.

[10] G. Bian, J. Rinkel, Z. Wang, L. Lauterbach, A. Hou, Y. Yuan, Z. Deng, T. Liu, J. S. Dickschat, *Angew. Chem. Int. Ed.* **2018**, *57*, 15887-15890.

[11] Z. Quan, J. S. Dickschat, *Org. Lett.* **2020**, *22*, 7552-7555.

[12] H. Li, J. S. Dickschat, *Org. Chem. Front.* **2022**, *9*, 795-801.

[13] H. Li, J. S. Dickschat, *Angew. Chem. Int. Ed*. **2022**, *61*, e202211054.

[14] A. E. Koepp, M. Hezari, J. Zajicek, B. Stofer Vogel, R. E. LaFever, N. G. Lewis, R. Croteau, *J. Biol. Chem.* **1995**, *270*, 8686-8690.

[15] Y. Ishihara, A. Mendoza, P. S. Baran, Tetrahedron, **2013**, *69*, 5685-5701

[16] P. Schrepfer, A. Buettner, C. Goerner, M. Hertel, J. van Rijn, F. Wallrapp, W. Eisenreich, V. Sieber, R. Kourist, T. Brück, *Proc. Natl. Acad. Sci. USA* **2016**, *113*, E958.

[17] T. Kato, M. Suzuki, T. Kobayashi, B. P. Moore, *J. Org. Chem.* **1980**, *45*, 1126-1130.

[18] A. Meguro, T. Tomita, M. Nishiyama, T. Kuzuyama, *ChemBioChem* **2013**, *14*, 316.

[19] Q. Jin, D. C. Williams, M. Hezari, R. Croteau, R. B. Coates, *J. Org. Chem.* **2005**, *70*, 4667.

[20] S. Grimme, S. Ehrlich, L. Goerigk, *J. Comp. Chem.* **2011**, *32*, 1456-1465.

[21] Gaussian 16, Revision C.01, M. J. Frisch, G. W. Trucks, H. B. Schlegel, G. E. Scuseria, M. A. Robb, J. R. Cheeseman, G. Scalmani, V. Barone, G. A. Petersson, H. Nakatsuji, X. Li, M. Caricato, A. V. Marenich, J. Bloino, B. G. Janesko, R. Gomperts, B. Mennucci, H. P. Hratchian, J. V. Ortiz, A. F. Izmaylov, J. L. Sonnenberg, D. Williams-Young, F. Ding, F. Lipparini, F. Egidi, J. Goings, B. Peng, A. Petrone, T. Henderson, D. Ranasinghe, V. G. Zakrzewski, J. Gao, N. Rega, G. Zheng, W. Liang, M. Hada, M. Ehara, K. Toyota, R. Fukuda, J. Hasegawa, M. Ishida, T. Nakajima, Y. Honda, O. Kitao, H. Nakai, T. Vreven, K. Throssell, J. A. Montgomery, Jr., J. E. Peralta, F. Ogliaro, M. J. Bearpark, J. J. Heyd, E. N. Brothers, K. N. Kudin, V. N. Staroverov, T. A. Keith, R. Kobayashi, J. Normand, K. Raghavachari, A. P. Rendell, J. C. Burant, S. S. Iyengar, J. Tomasi, M. Cossi, J. M. Millam, M. Klene, C. Adamo, R. Cammi, J. W. Ochterski, R. L. Martin, K. Morokuma, O. Farkas, J. B. Foresman, and D. J. Fox, Gaussian, Inc., Wallingford CT, 2019.

[22] S. Grimme, *Chem. Eur. J.* **2012**, *18*, 9955-9964.

[23] GoodVibes v3.0.1, G. Luchini, J. V. Alegre-Requena, Y. Guan, I. Funes-Ardoiz, R. S. Paton, 2019.

[24] C. Adamo, V. Barone, *J. Chem. Phys.* **1998**, *108*, 664.

[25] S. P. T. Matsuda, W. K. Wilson, Q. Xiong, *Org. Biomol. Chem.* **2006**, *4*, 530-543.

[26] Y. J. Hong, D. J. Tantillo, *J. Org. Chem.* **2018**, *83*, 3780-3793.

[27] L. Lauterbach, B. Goldfuss, J. S. Dickschat, *Angew. Chem.* **2020**, *132*, 12041-12045; *Angew. Chem. Int. Ed.* **2020**, *59*, 11943-11947.

[28] H. Xu, B. Goldfuss, J. S. Dickschat, *Chem. Eur. J.* **2021**, *27*, 9758-9762.

[29] P. Pracht, F. Bohle, S. Grimme, *Phys. Chem. Chem. Phys.* **2020**, *22*, 7169-7192.

[30] S. Grimme, *J. Chem. Theory Comput.* **2019**, *155*, 2847-2862.

[31] P. Pracht, S. Grimme, *Chem. Sci.* **2021**, *12*, 6551-6568.
[32] P. Pracht, C.A. Bauer, S. Grimme, *J. Comput. Chem.* **2017**, *38*, 2618-2631.
[33] S. Spicher, C. Plett, P. Pracht, A. Hansen, S. Grimme, *J. Chem. Theory Comput.* **2022**, *18*, 3174-3189.
[34] J. Bigeleisen, M. G. Mayer, Calculation of Equilibrium Constants for Isotopic Exchange Reactions, *J. Chem. Phys.* **1947**, *15*, 261.
[35] S. Edgar, F.-S. Li, K. Qiao, J.-K. Weng, G. Stephanopoulos, *ACS Synth. Biol.* **2017**, *6*, 201.
[36] M. Köksal, Y. Jin, R. M. Coates, R. Croteau, D. W. Christianson, *Nature* **2011**, *469*, 116.
